# *N*‐Terminal Selective C−H Azidation of Proline‐Containing Peptides: a Platform for Late‐Stage Diversification

**DOI:** 10.1002/chem.202200368

**Published:** 2022-02-23

**Authors:** Emmanuelle M. D. Allouche, Raphaël Simonet‐Davin, Jerome Waser

**Affiliations:** ^1^ Laboratory of Catalysis and Organic Synthesis Ecole Polytechnique Fédérale de Lausanne EPFL, SB ISIC LCSO, BCH 4306 1015 Lausanne Switzerland

**Keywords:** amino acids, azidation, C−H functionalization, peptides, selectivity

## Abstract

A methodology for the C−H azidation of *N*‐terminal proline‐containing peptides was developed employing only commercially available reagents. Peptides bearing a broad range of functionalities and containing up to 6 amino acids were selectively azidated at the carbamate‐protected *N*‐terminal residue in presence of the numerous other functional groups present on the molecules. Post‐functionalizations of the obtained aminal compounds were achieved: cycloaddition reactions or C−C bond formations via a sequence of imine formation/nucleophilic addition were performed, offering an easy access to diversified peptides.

## Graphical abstract

**A selective C−H azidation** of up to 6 amino acids long proline‐containing peptides is reported. In each example, the *N*‐terminal residue was selectively functionalized even in presence of amino acids bearing tertiary aliphatic, benzylic and α to heteroatoms C−H bonds. The azidated products were used in cycloaddition reactions and as masked imines for new C−C bond formations, allowing an easy access to diversified peptide scaffolds.

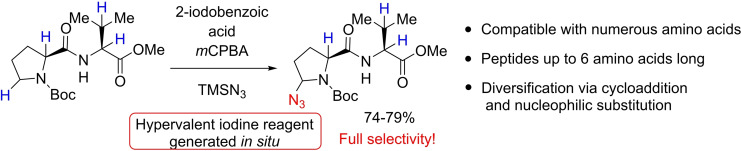

Numerous established pharmaceutical companies are conducting drug development on peptide‐based molecules.^[1]^ Methods to fine‐tune the structure of peptides are thus of interest to either improve their properties or to study their biological function.^[2]^ C−H functionalization is one of the most attractive strategies, as it is atom economic and targets the most prevalent chemical bonds. However, applying this approach to peptides represents a unique challenge, not only because of the range of functional groups present that can deactivate many catalysts, but also because of the low reactivity of C−H bonds and the difficulty of achieving selectivity.^[3]^ The introduction of an azide is of particular interest as it is one of the synthetically most useful functional groups and can undergo multiple transformations.^[4]^ However, despite impressive progress in the field of C−H azidation, most methods remain limited to less functionalized small organic molecules and terpene derivatives.^[5]^

As hypervalent iodine reagents are highly functional group tolerant and relatively non‐toxic, they have been used for the functionalization of amino acids‐containing biomolecules.^[6]^ The combination of hypervalent iodine/azide chemistry has demonstrated to be powerful for the azidation of small organic molecules.^[7]^ In 1994, Magnus and co‐workers reported the azidation of cyclic amines using a mixture of (PhIO)_n_/TMSN_3_ in dichloromethane at low temperature.^[8]^ This methodology was also applied on proline derivatives, generating δ‐azido amino acids as mixtures of diastereoisomers (Scheme 1a).^[9]^ A large amount of a mixture of PhIO (2.4 to 5 equivalents) and TMSN_3_ (4.8 to 10 equivalents) was used at −40 °C overnight, the in situ generated diazidated intermediate being highly explosive above −20 °C.^[10]^ In 2016, Chen and co‐workers described a visible‐light‐promoted azidation of tertiary C−H bonds and applied the strategy on two examples of leucine‐containing dipeptides (Scheme 1b).^[11]^ The Zhdankin reagent 1‐azido‐1,2‐benziodoxole‐3‐(1H)‐one (ABX, **1**)^[5a]^ was used as HAT as well as azide transfer reagent. To the best of our knowledge, this is the only example of C−H azidation performed on a peptide, despite the high potential of such a strategy for late‐stage peptide diversification.^[12]^

**Scheme 1:**
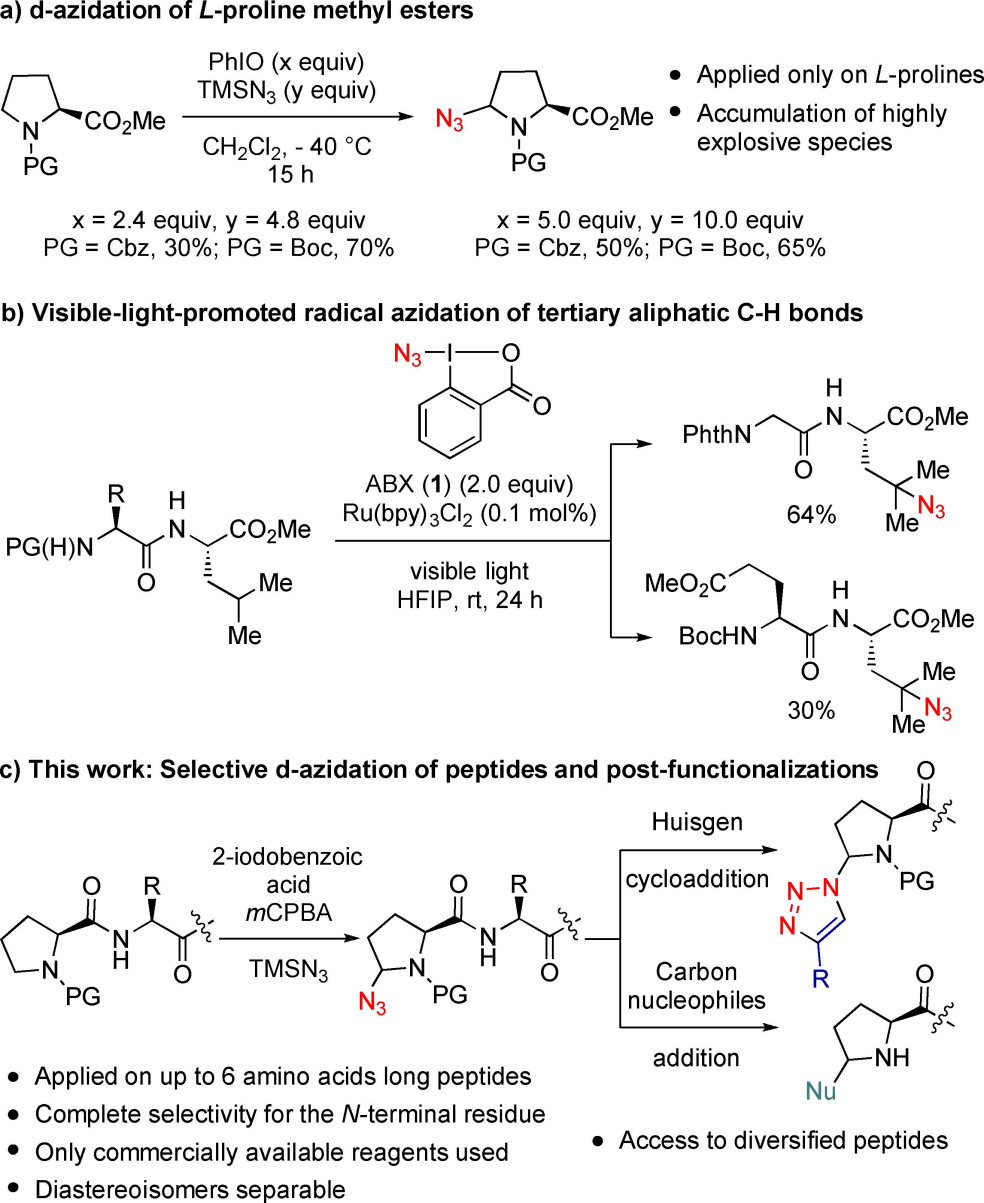
a) δ‐azidation of *L*‐proline methyl esters. b) Azidation of tertiary C−H bonds in leucine‐containing peptides. c) This work: *N*‐terminal selective δ‐azidation of *L*‐proline‐containing peptides as a platform for the formation of diversified scaffolds. PG=protecting group.

Herein, we describe a *N*‐terminal selective azidation of proline‐containing peptides using only stable and commercially available reagents (Scheme 1c). By generating the active hypervalent iodine compound in situ, we avoid the hazard associated with isolated reagents. This methodology, applied on up to 6 amino acids long peptides, allows the generation of azidated peptides that can undergo multiple transformations, providing an easy access to modified peptides. Beside classical cycloaddition reactions with alkynes, new C−C bonds were also generated via a sequence of imine formation/nucleophilic addition based on the leaving group ability of the azide.[^13^, ^14^]

Before moving to peptide substrates, we started our investigations by testing azidated cyclic hypervalent iodine reagents on prolines derivatives to develop safer and more convenient conditions for C−H azidation. In fact, ABX (**1**) is thermally stable up to 120 °C, even if care has to be used when handling highly pure crystalline compound, as it is sensitive to shock and friction.^[15]^ Cbz‐Pro‐OMe **3** was treated with two equivalents of ABX (**1**) using dichloromethane as the solvent. While low reactivity was observed at room temperature (Table S1, Entry 1), **4** was obtained in a 45 % ^1^H NMR yield as a mixture of diastereoisomers after overnight reaction at 45 °C (Table 1, Entry 2). The desired product **4** was generated in 50 % yield when the reaction was performed at 60 °C in dichloroethane (Table 1, Entry 3). Warming up the mixture at 80 °C led to the degradation of both starting material **3** and desired product **4** (Table 1, Entry 4). As we were also concerned about the explosivity of ABX (**1**) when manipulated as a solid,^[15]^ we tested the more stable azidobenziodazolone (ABZ, **2**) but less than 5 % of **4** were generated (Table 1, Entry 5). We thus envisaged the in situ generation of ABX from stable and commercially available reagents. With the mixture 2‐iodobenzoic acid/*m*‐CPBA/TMSN_3_ (2 equivalents of each), azidated compound **4** was formed in a 50 % yield, the same amount of starting material being recovered after the overnight reaction at 60 °C (Table 1, Entry 6). Interestingly, only two equivalents of TMSN_3_ were needed compared to ten equivalents in Magnus′ method to obtain a comparable yield (Scheme 1b).^[9]^ Despite an extensive optimization^[16]^ and similarly to Magnus′ work, further increase in conversion for this substrate was not possible. Interestingly, when the reaction was performed on the free acid proline **5**, azidated compound **6** was observed in a 35 % yield (Table 1, Entry 7) while only traces were generated when ABX (**1**) was used despite conversion of **5** (Table 1, Entry 8). A control experiment showed that all of the starting material **3** was recovered when the reaction was run without 2‐iodobenzoic acid (Table 1, Entry 9), supporting the hypothesis of an in situ formation of the ABX reagent. When a catalytic amount of 2‐iodobenzoic acid was used however, the reaction was not as efficient.[^16^, ^17^]

**Table 1:** Optimization of the reaction on Cbz‐Pro‐OMe **3**.

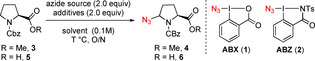						
Entry	R	Azide source	Additives	Solvent T °C	Yield^[a]^	Remaining SM^[a]^
1	Me	ABX (**1**)	–	DCM rt	<5 %	>95 %
2	Me	ABX (**1**)	–	DCM 40 °C	4 5%	51 %
3	Me	ABX (**1**)	–	DCE 60 °C	50 %	50 %
4	Me	ABX (**1**)	–	DCE 80 °C	40 %	36 %
5	Me	ABZ (**2**)	–	DCE 60 °C	<5%	>95 %
6	Me	TMSN_3_	2‐iodobenzoic acid *m*CPBA	DCE 60 °C	50 %	50 %
7^[b]^	H	TMSN_3_	2‐iodobenzoic acid *m*CPBA	DCE 60 °C	35 %	26 %
8	H	ABX (**1**)	–	DCE 60 °C	traces	42 %
9	Me	TMSN_3_	*m*CPBA	DCE 60 °C	0 %	>95 %

Reactions run on 0.1 mmol scale. 1 : 1 mixtures of diastereoisomers were obtained. O/N: overnight. [a] Determined by ^1^H NMR using mesitylene as internal standard. [b] Reaction run on 0.4 mmol scale.

We then studied the influence of both acid and amine protecting groups (Scheme 2). The variation of the ester part did not have any effect on the outcome of the reaction: methyl ester **4** and benzyl ester **7** were isolated in 36 % and 40 % yields, respectively, as mixtures of diastereoisomers. On the other hand, the nature of the carbamate had an important influence on the efficiency of the reaction.^[18]^ As observed by Magnus and co‐workers,^[9]^ the best result was obtained with a Boc protecting group,^[16]^ compound **8** being isolated in a 68 % yield. The transformation was very clean, and only traces of α‐ and δ‐diazidated proline were observed. When the reaction conditions were applied to an α‐methylated proline, compound **9** was obtained in a 72 % yield as the only observed product. Finally, azidated Boc‐Pro‐OH **10** was obtained in 50 % ^1^H NMR yield compared to 35 % for Cbz proline (compound **6**).

**Scheme 2:**
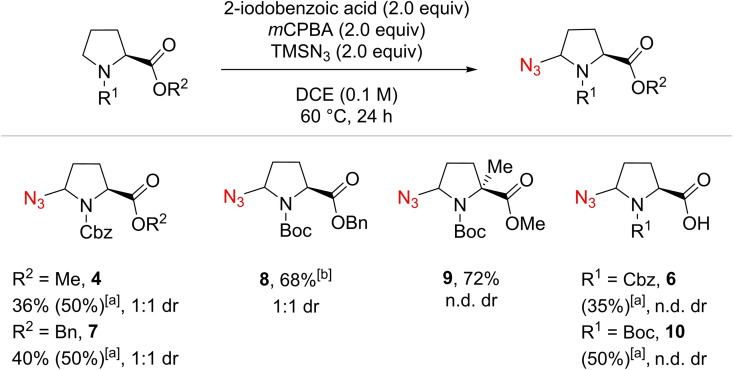
Preliminary evaluation of the scope of proline derivatives. Reactions run on 0.4 mmol scale. Isolated yields. [a] ^1^H NMR yield using mesitylene as internal standard. n.d. dr: dr not determined as complex mixtures of diastereoisomers and rotamers were obtained. [b] Traces of α‐ and δ‐diazidated product were observed.

We next examined a first simple dipeptide, Boc‐ProGly‐OMe (**11**) (Scheme 3a). In contrast to simple amino acids, dipeptide **11** has several C−H bonds activated by a neighboring nitrogen atom. However, peptide **12** with azidation on proline exclusively was isolated as the only product in 57 % yield. This result could be in principle rationalized by the electron‐withdrawing effect of the ester group, diminishing the electron density of the α C−H bond of the glycine residue. To test this hypothesis, the dipeptide Boc‐ProPro‐OMe (**13**) bearing two prolines was tested (Scheme 3b). To our surprise, only C−H azidation of the *N*‐terminal proline bearing the Boc group was obtained to give **14** in 55 % yield. Therefore, we speculated that the carbamate group was promoting C−H functionalization,^[19]^ and decided to investigate other carbamate protected amino acid derivatives (Scheme 3c). Azidated Boc‐Gly‐OMe **15** and Boc‐Gly‐OBn **16** were obtained in 34 % and 32 % yields, respectively.^[20]^ When a diphenyl urea was used instead of the carbamate, a slight increase of yield to 39 % was observed (compound **17**). In the case of the dipeptide Boc‐GlyPro‐OMe, exclusive azidation on the *N*‐terminal glycine was obtained to give **18** in 32 % yield despite the lower reactivity of the Gly residue. Finally, when the reaction was applied on α‐substituted amino acids, such as alanine, only traces of azidated compounds such as **19** were detected.

**Scheme 3:**
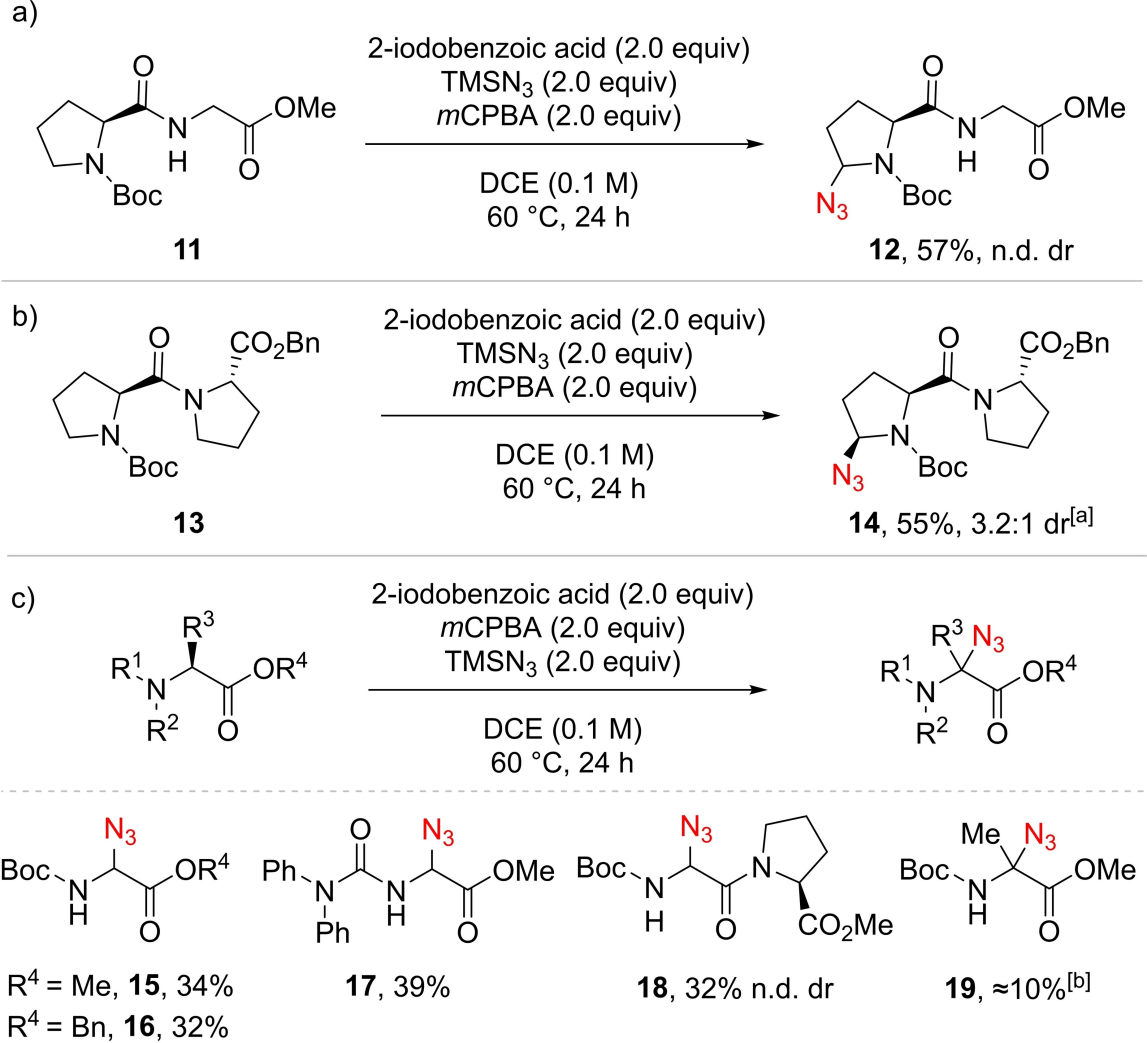
Activating effect of the *N*‐carbamate or urea. Reaction conditions: 0.4 mmol scale. Isolated yields. n.d. dr: dr not determined as complex mixtures of diastereoisomers and rotamers were obtained. [a] dr evaluated on the ^1^H NMR of the isolated mixture as the crude mixture was too complex, the major diastereoisomer is represented. [b] ^1^H NMR yield using mesitylene as internal standard, detected by HRMS.

The compatibility of the reaction with other amino acids was then studied using *N*‐terminal proline‐containing dipeptides (Scheme 4). Ala, Val, Leu, Phe along with protected functionalized amino acids such as Ser, Glu and Lys were tolerated, providing dipeptides **20**–**26** in 58 to 77 % yields. No side reactivity was observed even in presence of tertiary, α to heteroatom or benzylic C−H bonds. The case of protected lysine **26** is noteworthy, as no azidation was observed next to the primary Cbz protected amine. Interestingly, the presence of specific amino acids had a positive influence on the reaction. The transformation was particularly efficient in presence of Val and protected Ser (compounds **21** and **24**). The same trend was in part observed for glycine‐containing dipeptides: azidated Boc‐GlyVal‐OMe (**27**) and Boc‐GlyLeu‐O*t*‐Bu (**28**) were obtained in 41 % and 34 % yields, respectively. The reaction was scaled up to 1.0 mmol with no significant change in yield, allowing the isolation of **21** in 79 % yield. All the products were obtained with very low diastereoselectivity. We were however pleased to find that the two stereoisomers were easily separable by flash chromatography on silica gel in most cases (**20** to **25**), providing diastereomeric pure compounds. Access to different stereoisomers is essential in the context of medicinal chemistry.

**Scheme 4:**
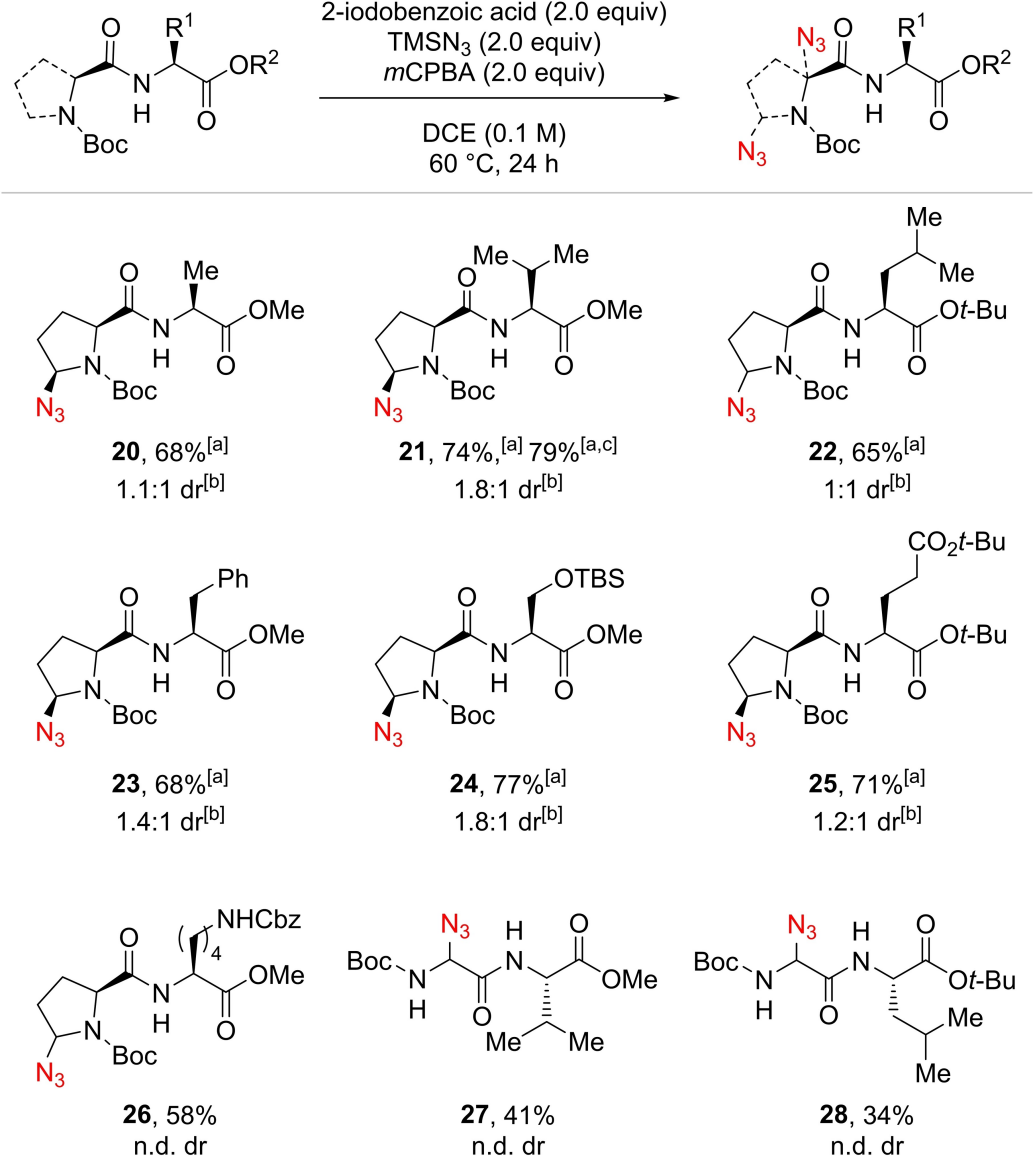
Scope of dipeptides. Reaction conditions: 0.4 mmol scale. Isolated yields. The major diastereoisomer is represented. n.d. dr: dr not determined as complex mixtures of diastereoisomers and rotamers were obtained. [a] Diastereoisomers separable by flash chromatography on silica gel. [b] Evaluation of the diastereomeric ratio according to isolated mass as crude compounds were obtained as complex mixture of diastereoisomers and rotamers. [c] Reaction done on 1.0 mmol scale.

Once we had demonstrated the compatibility of the reaction with numerous amino acids, we applied the conditions on longer peptides (Scheme 5). Azidated tetramers **29** and **30** were obtained in 33 % and 51 % yields respectively, the efficiency of the reaction was improved by the presence of the valine residue at the second position as previously observed on dipeptides. Pentamer **31** was formed in around 45 % yield while a decrease of the reaction efficiency to ±25 % yield was observed when hexamers were used as starting materials (compounds **32** and **33**). It is worthy to note that similar yields were obtained despite the presence of protected Glu and Ser in compound **33**. While 30 % non‐reacted starting material remained when the reaction was applied on the pentamer, only small amounts (<5 %) of unfunctionalized hexamers were observed. No other major peptidic product could be identified by HPLC (<2 %), highlighting the high selectivity of the azidation. It is worthy to note that compounds **12**, **14**, **18** and **32**, bearing several positions that could be azidated in the reaction (Pro or Gly), were functionalized at the *N*‐terminal residue only. We believe that this high selectivity is induced by the higher reactivity of carbamates when compared to amides.

**Scheme 5:**
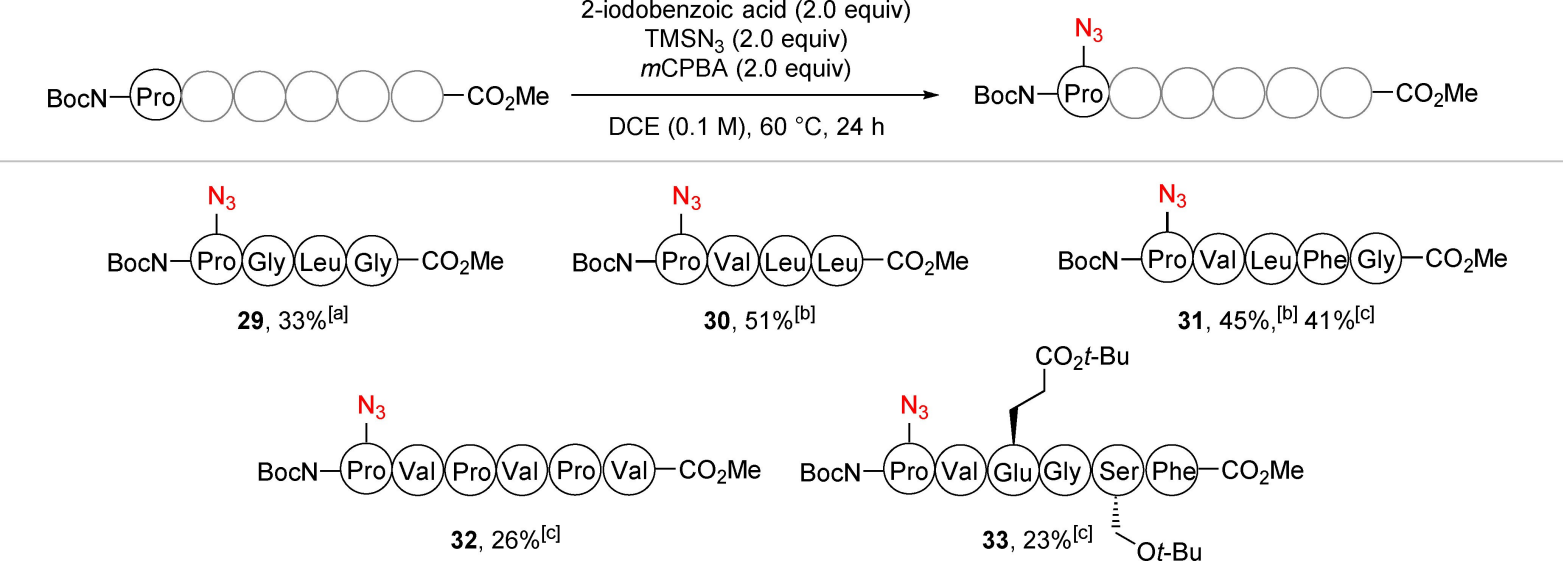
Scope of larger peptides. dr not determined as complex mixture of diastereoisomers and rotamers were obtained. [a] Isolated yield on 0.4 mmol scale. [b] Reactions done on 0.1 mmol scale, yields determined by ^1^H NMR using mesitylene as internal standard. [c] Reactions done on 0.1 mmol scale, calibrated yields estimated by HPLC‐UV (210 nm).^[16]^

Concerning the mechanism of the reaction and based on literature precedents,[^18^, ^21^, ^22^] a cationic species is most probably generated after the oxidation of the proline δ‐position or glycine α‐position. The *N*‐acyliminium formed could then be trapped by the nucleophilic azide to generate the azidated amino acid or peptide.

We next studied the derivatization of the azidated products (Scheme 6). We first performed a copper‐catalyzed Huisgen [3+2] cycloaddition using diastereomeric pure compounds **21 a** and **21 b** (Scheme 6a and 6b). Both substrates were converted into triazoles **34 a** and **34 b** in ≥96 % yield.^[23]^

**Scheme 6:**
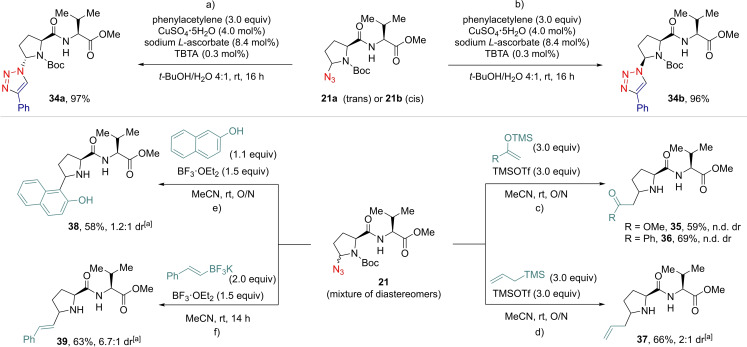
Post‐functionalization reactions: a) and b) cycloaddition reactions on single diastereoisomers **21 a** and **21 b**; c) to e) nucleophilic substitutions on crude **21**. O/N: overnight. n.d. dr: dr not determined as complex mixtures of diastereoisomers and rotamers were obtained. [a] Diastereomeric ratio evaluated by ^1^H NMR of the purified mixture as the crude was too complex.

In addition, we envisaged using these δ‐azidated proline‐containing peptides as masked imines to generate C−C bonds.^[13]^ To do so, the standard azidation conditions were used to synthesize **21** and triethylamine was added to the crude mixture after 24 h. Residual benzoic acid was removed using a filtration over a pad of silica.^[16]^ After evaporation, but no further purification, **21** (as a mixture of the two diastereoisomers) was dissolved in acetonitrile and treated with several nucleophiles in presence of a Lewis acid (Scheme 6c‐f). Enol ethers, TMS‐allyl, phenol and BF_3_K salts were added to generate compounds **35**–**39** as mixtures of diastereoisomers in good 58–69 % yields over two steps. The presence of TMSOTf or BF_3_ ⋅ OEt_2_ also triggered Boc deprotection.^[24]^ These two step procedures with a single purification at the end therefore resulted in a formal C−H alkylation, allylation, arylation and alkenylation of *N*‐terminal proline in a dipeptide. Two pathways can be envisaged for the addition of the C nucleophile: Boc deprotection could occur either before or after C−C bond formation, going either through an imine or an *N*‐acyliminium ion intermediate, respectively. When we attempted such reaction on a Cbz‐protected substrate, traces of C−C addition products were observed. This result indicated that the second process is possible, but the low yield observed does not allow to exclude cleavage of the carbamate first in the case of the Boc group.^[25]^

In summary, we have developed a new strategy for the C−H azidation of proline‐containing peptides using commercially available reagents. The reaction is compatible with numerous amino acids and up to 6 amino acids long peptides. Importantly, under the optimized reaction conditions, only the *N*‐terminal residue was functionalized. Diastereomeric pure azidated dipeptides could be obtained by flash chromatography separation of the two stereoisomers. Cycloaddition reactions were performed along with new C−C bond formations via an imine formation allowed by the donor property of the neighboring nitrogen and the leaving group ability of the azide. This methodology thus offers an easy access to diversified peptide scaffolds.^[26]^

## Conflict of interest

The authors declare no conflict of interest.

## Supporting information

As a service to our authors and readers, this journal provides supporting information supplied by the authors. Such materials are peer reviewed and may be re‐organized for online delivery, but are not copy‐edited or typeset. Technical support issues arising from supporting information (other than missing files) should be addressed to the authors.

Supporting Information

## Data Availability

The data that support the findings of this study are openly available in zenodo at 10.5281/zenodo.5975486, reference number 5975486.
