# Supplementary material for: N‐Terminal Selective C−H Azidation of Proline‐Containing Peptides: a Platform for Late‐Stage Diversification
Source: Chemistry. 2022 Feb 23;28(17):e202200368. doi: 10.1002/chem.202200368 (PMC9306896; doi:10.1002/chem.202200368)

Chemistry–A European Journal

Supporting Information

N-Terminal Selective C–H Azidation of Proline-Containing Peptides: a Platform for Late-Stage Diversification

Emmanuelle M. D. Allouche, Raphaël Simonet-Davin, and Jerome Waser*

Table of contents

1. General information ... 2

2. Starting materials preparation ... 3

2.1 General procedures for amino acids and peptides synthesis ... 3

2.2 Starting amino acids and peptides characterization data ... 6

2.3 Calibration curves for peptides 59, 60 and 61 ... 25

2.3 Procedures for the synthesis of ABX (1) and ABZ (2) ... 27

3. Optimization of the C-H azidation reaction ... 30

4. Scope of the azidation reaction ... 34

4.1 General procedures ... 34

4.2 Characterization data ... 35

4.3 Yields evalutation and characterization data for compounds 31, 32 and 33 ... 55

5. Post-functionalizations ... 59

5.1 Huisgen [3+2]-cycloadditions ... 59

5.2 Nucleophilic susbtitutions ... 61

6. Competitive Huisgen [3+2]-cycloaddition experiment ... 66

7. NMR spectra ... 67

1. General information

All reactions using anhydrous conditions were carried out in oven-dried glassware under an atmosphere of nitrogen using standard techniques for the manipulation of air-sensitive compounds. Anhydrous dichloromethane was taken from a commercial SPS solvent dispenser (H_2O content < 10 ppm, Karl-Fischer titration). Anhydrous 1,2-dichloroethane, dimethylformamide, acetonitrile, tetrahydrofuran and methanol were from chemical suppliers (Acros Organics). All reagent-grade chemicals were obtained from commercial suppliers (Acros, Aldrich, Fluka, VWR, Fluorochem, Combi-Blocks an Merck) and were used as received unless otherwise stated. Chromatographic purifications of products were accomplished using flash chromatography (FC) on SiliaFlash P60 silica gel (230 - 400 mesh) unless stated otherwise. For thin layer chromatography (TLC) analysis, pre-coated TLC sheets ALUGRAM® Xtra SIL G/UV_{254} were employed, using UV light as the visualizing agent and iodine, CAN or basic aqueous potassium permanganate stain solutions, and heat as developing agents.

The NMR spectra were recorded on Brucker DPX-400 and AV NEO-400 spectrometers (400 MHz for ^1H and 101 MHz for ^{13}C) at the specified temperature in $CDCl_3$, CD_2Cl_2 or MeOD-d_4. All signals are reported in ppm using the residual $CHCl_3$ (^1H: δ 7.26 ppm, ^{13}C: δ 77.16 ppm), CH_2Cl_2 (^1H: δ 5.32 ppm, ^{13}C: δ 53.84 ppm), MeOH (^1H: δ 3.31 ppm, ^{13}C: δ 49.00ppm) as references. The coupling constants (*J*) are reported in Hz. The following abbreviations were used to explain the multiplicities: app. = apparent, br = broad, s = singlet, d = doublet, t = triplet, td = triplet of doublet, q = quartet, dd = doublet of doublet, ddd = doublet of doublet of doublet, m = multiplet. When applicable, high temperatures ^1H NMR experiments were performed to determine if mixtures of rotamers or diastereoisomers were obtained. Melting points were measured on a Büchi B-540 melting point apparatus using open glass capillaries and are uncorrected. Infrared spectra were recorded on a JASCO FT-IR B4100 spectrophotometer with an ATR PRO410-S and a ZnSe prisma and are reported as cm^{-1} (w = weak, m = medium, s = strong, br = broad). High-resolution mass spectrometric measurements were performed by the mass spectrometry service of ISIC at the EPFL on LTQ Orbitrap ELITE ETD (Thermo fisher), Xevo G2-S QTOF (Waters), or LTQ Orbitrap ELITE ETD (Thermo fisher).

HPLC-MS measurements for azidated pentameres and hexamers were performed on an Agilent 1290 Infinity HPLC system with a G4226A 1290 Autosampler, a G4220A 1290 Bin Pump and a G4212A 1290 DAD detector, connected to a 6130 Quadrupole LC/MS, coupled with a Waters XBridge C18 column (250 x 4.6 mm, 5 µm). Water:acetonitrile 95:5 + 0.1% formic acid (solvent A), water:acetonitrile 5:95 + 0.1% formic acid (solvent B) were used as the mobile phase at a flow rate of 0.6 mL/min. The gradient was programmed as follow: Method 1: 100% A to 50% A in 2.5 min, then 50% A to 25% A in 20 min, then 25% A to 100% B in 2.5 min, then 100% B for 5 minutes; Method 2: 100% A to 70% A in 2.5 min, then 70% A to 60% A in 50 min, then 60% A to 100% B in 2.5 min, then 100% B for 5 minutes; Method 3: 100% A to 100% B in 20 minutes, then 100% B for 5 minutes.

The column temperature was set to 25 °C. Low resolution mass spectrometric measurements were acquired using the following parameters: positive electrospray ionization (ESI),

temperature of drying gas = 350 °C, flow rate of drying gas = 12 L min^{-1}, pressure of nebulizer gas = 60 psi, capillary voltage = 2500 V and fragmentor voltage = 70 V.

When the reaction was performed on pentameres and hexameres, as the products were not isolated, the regioselectivity of the azidation was confirmed using MS/MS analysis. The spectra were obtained by the mass spectrometry service of ISIC at the EPFL using Thermo Orbitrap Elite instrument. The desired ion was selected using mass filters and submitted to fragmentations. The obtained data was analyzed using fragment generation program on eln.epfl.ch.[1] For the calculations peak threshold for intensity was set to 0.01% for quantity, precision was set to 5 ppm and minimal similarity: 70%. The peaks were compared to theoretical peaks. The theoretical peak width was calculated from the mass of the ion by the formula provided in the script. The zone was set to -0.5 to 2.5 ppm. y and b fragments with and without the azide group were selected and reported.

2. Starting materials preparation

(Benzyloxy)carbonyl)-*L*-proline **5** and (*tert*-butoxycarbonyl)-*L*-proline were obtained from Combi-Blocks and were used as received.

2.1 General procedures for amino acids and peptides synthesis

General procedure A for the methylation of amino acids and peptides

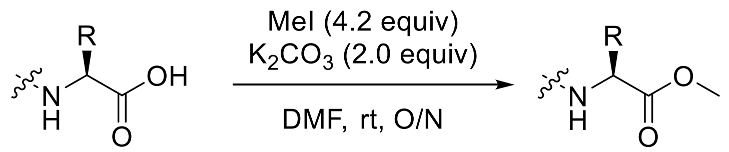

Using a slightly modified literature procedure,[2] potassium carbonate (2.0 equiv) was added to a stirred solution of the chosen free acid (1.0 equiv) in dry dimethylformamide (0.6 M). To this suspension, a solution of iodomethane (4.2 equiv) in dry dimethylformamide (2.5 M) was added dropwise using a syringe. The reaction was stirred at room temperature overnight under a nitrogen atmosphere. The solvent was then removed under reduced pressure and the crude mixture was dissolved in ethyl acetate, washed with water and brine several times and extracted twice. The combined organic layers were dried over anhydrous magnesium sulfate, filtered, and concentrated under reduced pressure to afford the desired compound which was used without any further purification.

[1] a) J. S. Desport, G. Frache, L. Patiny Ref Rapid Commun Mass Spectrom. 2020, e8652; b) D. Ortiz, N. Gasilova, F. Sepulveda, L. Patiny, P. J. Dyson, L. Menin, Ref Rapid Commun Mass Spectrom. 2020.
[2] C.-C. Chen, S.-F. Wang, Y.-Y. Su, Y. A. Lin, P-C. Lin, *Chem. Asian. J.* **2017**, *12*, 1326–1337.

General procedure B for the benzylation of amino acids

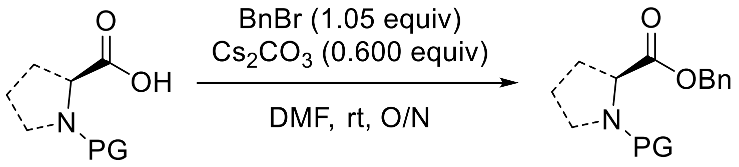

Cesium carbonate (0.600 equiv) was added to a stirred solution of the chosen free acid (1.00 equiv) in dry dimethylformamide (0.430 M). The suspension was stirred 15 minutes before the dropwise addition of benzyl bromide (1.05 equiv) via syringe. The reaction was stirred at room temperature overnight under a nitrogen atmosphere. The mixture was then diluted with water and extracted twice with ethyl acetate. The combined organic layers were washed successively with water and brine, dried over anhydrous magnesium sulfate, filtered, and concentrated under reduced pressure. The crude product was purified by flash chromatography on silica gel.

General procedure C for the Boc protection of amino acids

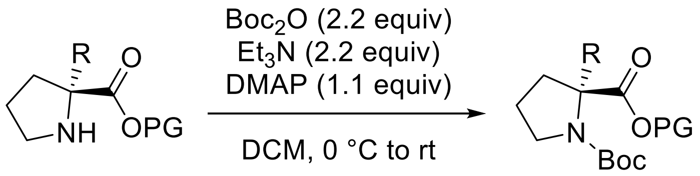

Using a slightly modified literature procedure,[3] to a solution of the chosen substrate (1.0 equiv) in dry dichloromethane (0.27 M) cooled down to 0 °C using an ice bath, were added di-*tert*-butyldicarbonate (2.2 equiv), triethylamine (2.2 equiv) and 4-(dimethylamino)pyridine (1.1 equiv). The cooling bath was removed and the reaction was stirred overnight under a nitrogen atmosphere. The reaction mixture was then diluted with dichloromethane, washed consecutively with aqueous 1 N hydrochloric acid and saturated sodium carbonate solutions, dried over magnesium sulfate and concentrated under reduced pressure. The crude product was purified by flash chromatography on silica gel.

[3] K. Jones, K.-C. Woo, *Tetrahedron* **1991**, *47*, 7179–7184.

General procedure D for the synthesis of dipeptides and tetramers

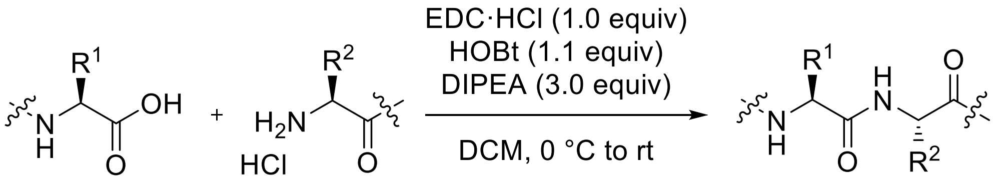

Using a slightly modified literature procedure,[4] to a solution of the chosen acide (1.0 equiv) in dry dichloromethane (0.16 M) cooled down to 0 °C using an ice bath, were added *N*,*N*-diispopropylethylamine (DIPEA, 3.0 equiv), the chosen amine hydrochloride salt (1.0 equiv), 1-hydroxybenzotriazole (HOBt, 1.1 equiv) and 1-ethyl-3-(3-dimethylaminopropyl)carbodiimide hydrochloride (EDC·HCl, 1.0 equiv). The cooling bath was removed and the reaction was stirred overnight under a nitrogen atmosphere. The reaction mixture was then quenched with a saturated sodium carbonate solution and extracted twice with ethyl acetate. The combined organic layers were washed consecutively with 10 wt% aqueous citric acid solution and brine, dried over magnesium sulfate and concentrated under reduced pressure. The crude product was purified by flash chromatography on silica gel.

General procedure E for the synthesis of pentamers and hexamers

Solid-Phase Peptide Synthesis (SPPS): Pentamers and hexamers were synthesized on an Advanced ChemTech 348-Ω parallel peptide synthesizer (AAPPTec) using standard Fmoc SPPS-chemistry and 2-chlorotrityl chloride resin (100-200 mesh, 1% DVB, 1.0-1.6 mmol Cl/g). Inside each SPPS syringe were manually added 70 mg of resin (c.a. 80 µmol, 1.0 equiv), followed by the chosen *C*-terminal Fmoc-protected monomer (0.32 mmol, 4.0 equiv), *N*,*N*-diispopropylethylamine (79 µL, 0.48 mmol, 6.0 equiv) and dichloromethane (0.04 M, 2.0 mL). The syringes were shacked for 2 hours and the resin was washed with dimethylformamide (5 x 3.0 mL) and dichloromethane (5 x 3.0 mL). Capping was performed using a mixture Ac_2O:2,6-lutidine:DMF (5:6:89) and the resin was washed with dimethylformamide (4 x 3.0 mL). Fmoc protecting group was then removed by shaking the resin with 20% v/v piperidine in dimethylformamide at 400 rpm 5 minutes. After dimethylformamide (5 x 3.0 mL) and dichloromethane (5 x 3.0 mL) washes, the next coupling was carried out by shaking the resin with the chosen Fmoc-protected monomer (0.32 mmol, 4.0 equiv), 1-[bis(dimethylamino)methylene]-1*H*-1,2,3-triazolo[4,5-*b*]pyridinium 3-oxide hexafluorophosphate (HATU, 0.32 mmol, 4.0 equiv) and 4-methylmorpholine (NMM, 0.48 mmol, 6.0 equiv) in dimethylformamide (6.0 mM, 1.3 mL) at 400 rpm 30 minutes. The capping, Fmoc removal and coupling steps were repeated 4 or 5 times to obtain pentamers and hexamers, respectively. As only *N*-terminal Boc-proline-containing peptides were synthesized, the final coupling was performed with Boc-Pro-OH each time.

[4] P. Guo, K. Wang, W.-J. Jin, H. Xie, L. Qi, X.-Y. Liu, X.-Z. Shu, *J. Am. Chem. Soc.* **2021**, *143*, 513–523.

Peptide cleavage: Peptides were cleaved from the resin by treatment with a 4:1 dichloromethane:hexafluoroisopropanol mixture (1.0 mL). The resulting suspension was shaken 3 hours at 400 rpm at room temperature. The resin was removed by filtration and peptides were precipitated in cold diethyl ether (20 mL). Peptides were pelleted by centrifugation at 4000 rpm for 5 minutes at 4 °C. Finally, the mother liquors were carefully removed and crude peptides were dried under vacuum. In absence of precipitation in diethyl ether, everything was evaporated and dried under vacuum.

Crude peptides were then directly methylated using general procedure **A**.

2.2 Starting amino acids and peptides characterization data

1-Benzyl 2-methyl (*S*)-pyrrolidine-1,2-dicarboxylate (5)

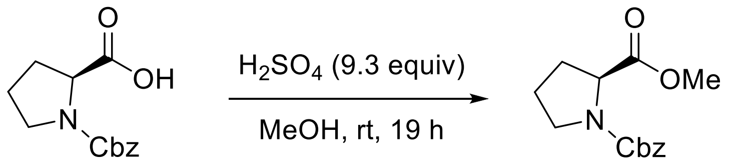

5 **3**

(Benzyloxy)carbonyl)-*L*-proline **5** (1.0 g, 4.0 mmol, 1.0 equiv) was weighed in an oven-dried 20 mL microwave vial equipped with a magnetic stir bar and dissolved with 8.0 mL of anhydrous methanol. Sulphuric acid (2.0 mL, 37 mmol, 9.3 equiv) was added dropwise and the reaction was stirred 19 hours at room temperature under a nitrogen atomosphere. The reaction mixture was then poured onto crushed ice and the mixture was extracted twice with diethylether. The combined organic layers were dried over magnesium sulfate and concentrated under reduced pressure to afford crude 1-benzyl 2-methyl (*S*)-pyrrolidine-1,2-dicarboxylate **3** as a clear oil (1.1 g, 4.0 mmol, 99%) which was used without further purification.

^{1}H NMR (400 MHz, 298 K, mixture of two rotamers) δ 7.39 – 7.27 (m, 5H, Ar*H*), 5.23 – 4.99 (m, 2H, O*CH$_2$*Ph), 4.39 (dd, *J* = 8.6, 3.5 Hz, 0.5H, NCH$_2$CH$_2$CH$_2$C*H*C(O)), 4.33 (dd, *J* = 8.6, 3.8 Hz, 0.5H, NCH$_2$CH$_2$CH$_2$C*H*C(O)), 3.73 (s, 1.5H, O*CH$_3$*), 3.66 – 3.59 (m, 1H, NC*H*HCH$_2$CH$_2$CHC(O)), 3.57 (s, 1.5H, O*CH$_3$*), 3.55 – 3.44 (m, 1H, NCH*H*CH$_2$CH$_2$CHC(O)), 2.31 – 2.12 (m, 1H, NCH$_2$CH$_2$C*H*HCHC(O)), 2.07 – 1.82 (m, 3H, NCH$_2$C*H$_2$*CH$_2$CHC(O) + NCH$_2$CH$_2$CH*H*CHC(O)). **^{13}C NMR** (101 MHz, CDCl$_3$, 298 K, mixture of two rotamers) δ 173.4 (Cq), 173.2 (Cq), 155.0 (Cq), 154.4 (Cq), 136.8 (Cq), 136.7 (Cq), 128.5 (CH), 128.5 (CH), 128.1 (CH), 128.0 (CH), 127.97 (CH), 127.9 (CH), 67.1 (CH$_2$), 67.0 (CH$_2$), 59.3 (CH), 58.9 (CH), 52.3 (CH$_3$), 52.1 (CH$_3$), 47.0 (CH$_2$), 46.5 (CH$_2$), 31.0 (CH$_2$), 30.0 (CH$_2$), 24.4 (CH$_2$), 23.6 (CH$_2$).

NMR spectra are in agreement with the reported data.[5]

[5] W. A. Loughlin, S. S. Schweiker, I. D. Jenkins, L. C. Henderson, *Tetrahedron* **2013,** *69*, 1576–1582.

Dibenzyl (*S*)-pyrrolidine-1,2-dicarboxylate (40)

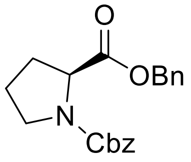

Prepared according to the general procedure **B** from ((benzyloxy)carbonyl)-*L*-proline (1.5 g, 6.0 mmol, 1.0 equiv), benzyl bromide (0.75 mL, 6.3 mmol, 1.1 equiv), cesium carbonate (1.2 g, 3.6 mmol, 0.60 equiv) in *N*,*N*-dimethylformamide (14 mL). The crude mixture was purified by flash chromatography on silica gel using dichloromethane as eluent to afford dibenzyl (*S*)-pyrrolidine-1,2-dicarboxylate **40** (1.8 g, 5.4 mmol, 90%) as a yellowish oil.

Rf (dichloromethane): 0.11. **^{1}H NMR** (400 MHz, $CDCl_3$, 298 K, mixture of two rotamers) δ 7.32 – 7.11 (m, 10H, Ar*H*), 5.19 – 4.89 (m, 4H, 2 x OC*H*$_2$Ph), 4.37 (dd, *J* = 8.6, 3.4 Hz, 0.5H, $NCH_2CH_2CH_2C$*H*C(O)), 4.30 (dd, *J* = 8.6, 3.8 Hz, 0.5H, $NCH_2CH_2CH_2C$*H*C(O)), 3.61 – 3.49 (m, 1H, NC*H*HCH_2CH_2CHC(O)), 3.49 – 3.31 (m, 1H, NCH*H*CH_2CH_2CHC(O)), 2.24 – 2.03 (m, 1H, NCH_2CH_2C*H*HCHC(O)), 2.01 – 1.71 (m, 3H, NCH_2C*H*$_2CH_2CHC$(O) + NCH_2CH_2CH*H*CHC(O)). **^{13}C NMR** (101 MHz, $CDCl_3$, 298 K, mixture of two rotamers, signals not fully resolved) δ 172.8 (Cq), 172.6 (Cq), 155.0 (Cq), 154.4 (Cq), 136.8 (Cq), 136.7 (Cq_j), 135.9 (Cq), 135.7 (Cq_j), 128.7 (CH), 128.6 (CH), 128.5 (CH), 128.4 (CH), 128.3 (CH), 128.22 (CH), 128.17 (CH), 128.1 (CH), 128.1 (CH), 128.0 (CH), 127.9 (CH), 67.1 (CH_2), 67.08 (CH_2), 66.9 (CH_2), 66.8 (CH_2), 59.4 (CH), 59.1 (C), 47.1 (CH_2), 46.6 (CH_2), 31.0 (CH_2), 30.0 (CH_2), 24.4 (CH_2), 23.7 (CH_2).

NMR spectra are in agreement with the reported data.[6]

2-Benzyl 1-(*tert*-butyl) (*S*)-pyrrolidine-1,2-dicarboxylate (41)

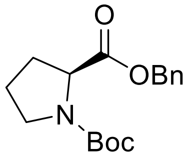

Prepared according to the general procedure **C** from benzyl *L*-prolinate hydrochloride (0.75 g, 3.0 mmol, 1.0 equiv), di-*tert*-butyldicarbonate (1.5 g, 6.6 mmol, 2.2 equiv), triethylamine (0.90 mL, 6.6 mmol, 2.2 equiv) and 4-(dimethylamino)pyridine (0.41 g, 3.3 mmol, 1.1 equiv) in dichloromethane (11 mL). The crude mixture was purified by flash chromatography on silica gel using a gradient from pentane to pentane/ethyl acetate 9:1 as eluent to afford 2-benzyl 1-(*tert*-butyl) (*S*)-pyrrolidine-1,2-dicarboxylate **41** as a yellow liquid (0.89 g, 2.9 mmol, 98%).

Rf (pentane/ethyl acetate 9:1): = 0.31. **^{1}H NMR** (400 MHz, $CDCl_3$ 298 K, mixture of two rotamers) δ 7.36 – 7.30 (m, 5H, Ar*H*), 5.32 – 5.02 (m, 2H, OC*H*$_2$Ph), 4.38 (dd, *J* = 8.7, 3.4 Hz, 0.4H, $NCH_2CH_2CH_2C$*H*$C(O)_{rotamermin}$), 4.26 (dd, *J* = 8.6, 3.9 Hz, 0.6H,

[6] K. Hattori, H. Sajiki, K. Hirota, *Tetrahedron Lett.* **2000**, *56*, 8433–8441.

$NCH_2CH_2CH_2CHC(O)_{rotamermaj}$), 3.64 – 3.28 (m, 2H, $NCH_2CH_2CH_2CHC(O)$), 2.27 – 2.12 (m, 1H, $NCH_2CH_2CHHCHC(O)$), 2.03 – 1.79 (m, 3H, $NCH_2CH_2CH_2CHC(O)$ + $NCH_2CH_2CHHCHC(O)$), 1.46 (s, 3.6H, $CH_{3Bocrotamermin}$), 1.34 (s, 5.4H, $CH_{3Bocrotamermaj}$). **^{13}C NMR** (101 MHz, $CDCl_3$, 298 K, mixture of two rotamers, signals not fully resolved) δ 173.2 ($Cq_{rotamermaj}$), 172.9 ($Cq_{rotamermin}$), 154.5 ($Cq_{rotamermin}$), 153.9 ($Cq_{rotamermaj}$), 136.0 ($Cq_{rotamermin}$), 135.8 ($Cq_{rotamermaj}$), 128.7 (CH), 128.6 (CH), 128.5 (CH), 128.4 (CH), 128.2 (CH), 128.1 (CH), 80.0 ($Cq_{rotamermaj}$), 79.9 ($Cq_{rotamermin}$), 66.8 (CH_2), 59.3 ($CH_{rotamermaj}$), 59.0 ($CH_{rotamermin}$), 46.7 ($CH_{2rotamermin}$), 46.5 ($CH_{2rotamermaj}$), 31.0 ($CH_{2rotamermaj}$), 30.0 ($CH_{2rotamermin}$), 28.6 ($CH_{3rotamermin}$), 28.4 ($CH_{3rotamermaj}$), 24.4 ($CH_{2rotamermin}$), 23.7 ($CH_{2rotamermal}$).

NMR spectra are in agreement with the reported data.[7]

1-(*tert*-Butyl) 2-methyl (*S*)-2-methylpyrrolidine-1,2-dicarboxylate (42)

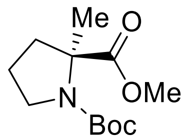

Prepared according to the general procedure **C** from methyl (*S*)-2-methylpyrrolidine-2-carboxylate hydrochloride (0.54 g, 3.0 mmol, 1.0 equiv), di-*tert*-butyldicarbonate (1.5 g, 6.6 mmol, 2.2 equiv), triethylamine (0.90 mL, 6.6 mmol, 2.2 equiv) and 4-(dimethylamino)pyridine (0.41 g, 3.3 mmol, 1.1 equiv) in dichloromethane (11 mL). The crude mixture was purified by flash chromatography on silica gel using a gradient from dichloromethane to dichloromethane/methanol 98:2 as eluent to afford 1-(*tert*-butyl) 2-methyl (*S*)-2-methylpyrrolidine-1,2-dicarboxylate **42** as a yellow liquid (0.26 g, 1.0 mmol, 35%).

Rf (dichloromethane/methanol 98:2): = 0.52. **^{1}H NMR** (400 MHz, $CDCl_3$, 298 K, mixture of two rotamers) δ 3.69 (s, 3H, OCH_3), 3.62 – 3.36 (m, 2H, $NCH_2CH_2CH_2CC(O)$), 2.20 – 2.08 (m, 1H, $NCH_2CH_2CHHCC(O)$), 1.96 – 1.75 (m, 3H, $NCH_2CH_2CH_2CC(O)$ + $NCH_2CH_2CHHCC(O)$), 1.54 (s, 0.9H, $CH_{3rotamermin}$), 1.49 (s, 2.1H, $CH_{3rotamermaj}$), 1.42 (s, 3H, $CH_{3Bocrotamermin}$), 1.39 (s, 6H, $CH_{3Bocrotamermaj}$). **^{13}C NMR** (101 MHz, $CDCl_3$, 298 K, mixture of two rotamers) δ 175.5 ($Cq_{rotamermaj}$), 175.3 ($Cq_{rotamermin}$), 154.0 ($Cq_{rotamermin}$), 153.7 ($Cq_{rotamermaj}$), 80.0 ($Cq_{rotamermaj}$), 79.6 ($Cq_{rotamermin}$), 65.3 ($Cq_{rotamermin}$), 64.9 ($Cq_{rotamermaj}$), 52.3 ($CH_{3rotamermin}$), 52.2 ($CH_{3rotamermaj}$), 48.0 ($CH_{2rotamermin}$), 47.8 ($CH_{2rotamermaj}$), 40.3 ($CH_{2rotamermaj}$), 39.3 ($CH_{2rotamermin}$), 28.5 ($CH_{3rotamermin}$), 28.4 ($CH_{3rotamermaj}$), 23.5 ($CH_{2rotamermin}$), 23.3 ($CH_{3rotamermaj}$), 22.9 ($CH_{2rotamermaj}$), 22.4 ($CH_{3rotamermin}$). **IR** (ν_{max}, cm^{-1}) 2980 (m), 2889 (w), 1743 (s), 1698 (s), 1390 (s), 1367 (s), 1165 (s). **HRMS** (ESI/QTOF) m/z: $[M + Na]^+$ Calcd for $C_{12}H_{21}NNaO_4^+$ 266.1363; Found 266.1366.

[7] Y. Wang, X. Wen, X. Cui, X. P. Zhang, *J. Am. Chem. Soc.* **2018,** *140*, 4792–4796.

tert-Butyl (S)-2-((2-methoxy-2-oxoethyl)carbamoyl)pyrrolidine-1-carboxylate (11)

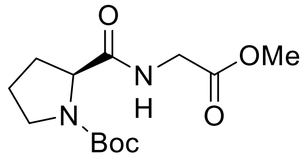

Prepared according to the general procedure **A** from (*tert*-butoxycarbonyl)-*L*-prolylglycine (0.82 g, 3.0 mmol, 1.0 equiv), iodomethane (0.75 mL, 12 mmol, 4.0 equiv), potassium carbonate (0.83 g, 6.0 mmol, 2.0 equiv) in *N*,*N*-dimethylformamide (2 x 4.7 mL). *tert*-butyl (*S*)-2-((2-methoxy-2-oxoethyl)carbamoyl)pyrrolidine-1-carboxylate **11** (0.63 g, 2.2 mmol,73%) was obtained as a yellow sticky oil.

^{1}H NMR (400 MHz, $CDCl_3$, 298 K, mixture of two rotamers) δ 7.29 (s, 0.5H, N*H*), 6.53 (s, 0.5H, N*H*), 4.33 – 4.26 (br m, 1H, $NCH_2CH_2CH_2C\mathit{H}_{Pro}C(O)$), 4.16 – 3.86 (m, 2H, $NHC\mathit{H}_{2Gly}C(O)$), 3.74 (s, 3H, $OC\mathit{H}_3$), 3.55 – 3.34 (m, 2H, $NC\mathit{H}_{2Pro}CH_2CH_2CHC(O)$), 2.41 – 2.05 (br m, 1H, $NCH_2CH_2C\mathit{H}H_{Pro}CHC(O)$), 1.94 – 1.76 (m, 3H, $NCH_2CH_2CH\mathit{H}_{Pro}CHC(O)$ + $NCH_2C\mathit{H}_{2Pro}CH_2CHC(O)$), 1.46 (s, 9H, $C\mathit{H}_{3Boc}$). **^{13}C NMR** (101 MHz, $CDCl_3$, 298 K, mixture of two rotamers, signals not fully resolved) δ 173.1 (Cq), 172.5 (Cq), 170.3 (Cq), 156.0 (Cq), 154.8 (Cq), 80.7 (Cq), 61.2 (CH), 60.1 (CH), 52.4 (CH_3), 47.3 (CH_2), 41.3 (CH_2), 31.1 (CH_2), 28.5 (CH_3), 24.6 (CH_2), 23.9 (CH_2).

NMR spectra are in agreement with the reported data.[8]

tert-Butyl (S)-2-((S)-2-((benzyloxy)carbonyl)pyrrolidine-1-carbonyl)pyrrolidine-1-carboxylate (13)

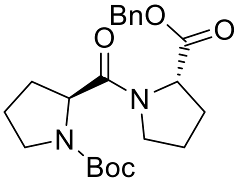

Prepared according to the general procedure **D** from (*tert*-butoxycarbonyl)-*L*-proline (0.65 g, 3.0 mmol, 1.0 equiv), benzyl *L*-prolinate hydrochloride (0.73 g, 3.0 mmol, 1.0 equiv), 1-(3-dimethylaminopropyl)-3-ethylcarbodiimide hydrochloride (0.58 g, 3.0 mmol, 1.0 equiv), 1-hydroxybenzotriazole hydrate (0.51 g, 3.3 mmol, 1.1 equiv) and *N*,*N*-diisopropylethylamine (1.6 mL, 9.0 mmol, 3.0 equiv) in dichloromethane (19.0 mL). The crude mixture was purified by flash chromatography on silica gel using a gradient from pentane to pentane/ethyl acetate 1:1 as eluent to afford *tert*-butyl (*S*)-2-((*S*)-2-((benzyloxy)carbonyl)pyrrolidine-1-carbonyl)pyrrolidine-1-carboxylate **13** (0.93 g, 2.3 mmol, 77%) as a yellowish sticky oil.

Rf (pentane/ethyl acetate 1:1): 0.26. **^{1}H NMR** (400 MHz, MeOD-d_4, 298 K, mixture of two rotamers) δ 7.40 – 7.28 (m, 5H, Ar*H*), 5.19 (dd, *J* = 12.2, 4.4 Hz, 1H, OC*H*HPh), 5.08 (dd, *J* =

[8] M. Inman, H. L. Dexter, C. J. Moody, *Org. Lett.* **2017**, *19*, 3454–3457.

12.2, 1.0 Hz, 1H, OCH*H*Ph), 4.60 – 4.44 (m, 2H, NC*H*C(O)), 3.81 – 3.57 (m, 2H, N$CH_2CH_2CH_2$CHC(O)), 3.52 – 3.45 (m, 1H, N$CH_2CH_2CH_2$CHC(O)), 3.42 – 3.36 (m, 1H, N$CH_2CH_2CH_2$CHC(O)), 2.32 – 2.12 (m, 2H, NCH_2CH_2C*H*HCHC(O)), 2.10 – 1.73 (m, 6H, NCH_2CH_2CH*H*CHC(O) + N$CH_2CH_2CH_2$CHC(O)), 1.45 (s, 4H, CH_3Boc), 1.39 (s, 5H, CH_3Boc). **^{13}C NMR** (101 MHz, MeOD-d_4, 298 K, mixture of two rotamers, signals not fully resolved) δ 173.9 (Cq), 173.4 (Cq), 173.4 (Cq), 173.2 (Cq), 156.2 (Cq), 155.6 (Cq), 137.2 (Cq), 137.2 (Cq), 129.6 (CH), 129.4 (CH), 129.4 (CH), 81.3 (Cq), 81.2 (Cq), 68.0 (CH_2), 67.9 (CH_2), 60.6 (CH), 59.3 (CH), 59.1 (CH), 48.2 (CH_2), 48.0 (CH_2), 47.9 (CH_2), 47.8 (CH_2), 30.7 (CH_2), 30.0 (CH_2), 29.9 (CH_2), 29.8 (CH_2), 28.7 (CH_3), 28.6 (CH_3), 25.9 (CH_2), 25.1 (CH_2), 24.6 (CH_2). **IR** (ν_{max}, cm^{-1}) 2974 (w), 2881 (w), 1743 (m), 1693 (s), 1658 (s), 1396 (s), 1165 (s), 1122 (m), 741 (m), 698 (m). **HRMS** (ESI/QTOF) m/z: $[M + Na]^+$ Calcd for $C_{22}H_{30}N_2NaO_5^+$ 425.2047; Found 425.2053.

Methyl (*tert*-butoxycarbonyl)glycinate (43)

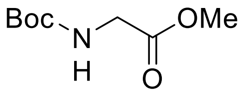

Prepared according to the general procedure **A** from (*tert*-butoxycarbonyl)glycine (1.0 g, 5.7 mmol, 1.0 equiv), iodomethane (1.4 mL, 23 mmol, 4.0 equiv), potassium carbonate (1.6 g, 11 mmol, 2.0 equiv) in *N*,*N*-dimethylformamide (2 x 9.0 mL). Methyl (*tert*-butoxycarbonyl)glycinate **43** (1.1 g, 5.7 mmol, quant.) was obtained as a yellowish liquid.

^{1}H NMR (400 MHz, $CDCl_3$, 298 K) δ 5.00 (br s, 1H, N*H*), 3.92 (d, *J* = 5.7 Hz, 2H, NHCH_2C(O)), 3.75 (s, 3H, OCH_3), 1.45 (s, 9H, CH_{3Boc}). **^{13}C NMR** (101 MHz, $CDCl_3$, 298 K) δ 171.0 (Cq), 155.8 (Cq), 80.1 (Cq), 52.3 (CH_3), 42.4 (CH_2), 28.4 (CH_3).

NMR spectra are in agreement with the reported data.[9]

Benzyl (*tert*-butoxycarbonyl)glycinate (44)

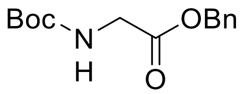

Prepared according to the general procedure **B** from (*tert*-butoxycarbonyl)glycine (0.53 g, 3.0 mmol, 1.0 equiv), benzyl bromide (0.38 mL, 3.2 mmol, 1.1 equiv), cesium carbonate (0.59 g, 1.8 mmol, 0.60 equiv) in *N*,*N*-dimethylformamide (7.0 mL). The crude mixture was purified by flash chromatography on silica gel using a gradient from dichloromethane to dichloromethane/methanol 98:2 as eluent to afford benzyl (*tert*-butoxycarbonyl)glycinate **44** (0.68 g, 2.6 mmol, 85%) as a yellowish solid.

Rf (dichloromethane/methanol 98:2): 0.34. **^{1}H NMR** (400 MHz, $CDCl_3$, 298 K) δ 7.39 – 7.31 (m, 5H, Ar*H*), 5.18 (s, 2H, OCH_2Ph), 5.01 (br s, 1 H, N*H*), 3.96 (d, *J* = 5.6 Hz, 2H, NHCH_2C(O)), 1.45

[9] K. K. H. Vong, S. Maeda, K. Tanaka, *Chem. Eur. J.* **2016**, *22*, 18865–18872.

(s, 9H, CH_{3Boc}). **^{13}C NMR** (101 MHz, $CDCl_3$, 298 K) δ 170.4 (Cq), 155.8 (Cq), 135.4 (Cq), 128.8 (CH), 128.6 (CH), 128.5 (CH), 80.2 (Cq), 67.2 (CH_2), 42.6 (CH_2), 28.4 (CH_3).

NMR spectra are in agreement with the reported data.[10]

Methyl (diphenylcarbamoyl)glycinate (45)

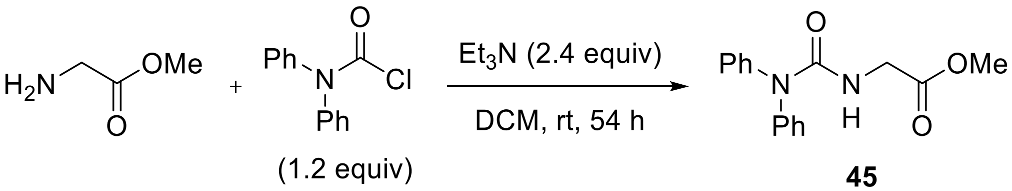

Glycine methyl ester hydrochloride (0.32 g, 2.6 mmol, 1.0 equiv) was weighed in an oven-dried 50 mL round-bottomed flask equipped with a magnetic stir bar and dissolved with 14 mL of anhydrous dichloromethane. Triethylamine (0.85 mL, 6.1 mmol, 2.4 equiv) was then added followed by diphenylcarbamoyl chloride (0.71 g, 3.1 mmol, 1.2 equiv) dissolved in 3.4 mL of anhydrous dichloromethane. The reaction was stirred under a nitrogen atmosphere over weekend. The reaction mixture was then quenched with water and extracted twice with dichloromethane. The combined organic layers were washed with brine, dried over anhydrous magnesium sulfate, filtered and concentrated under reduced pressure. The crude product was purified by flash chromatography on silica gel using a gradient from dichloromethane to dichloromethane/ethyl acetate 9:1 as eluent to afford methyl (diphenylcarbamoyl)glycinate **45** as a white solid (0.66 g, 2.3 mmol, 91%).

Rf (dichloromethane/ethyl acetate 9:1): = 0.36. **Mp:** 121.4 - 123.0 °C. **^{1}H NMR** (400 MHz, $CDCl_3$, 298 K) δ 7.38 – 7.34 (m, 4H, Ar*H*), 7.32 – 7.29 (m, 4H, Ar*H*), 7.25 – 7.21 (m, 2H, Ar*H*), 5.04 (br s, 1H, N*H*), 4.04 (d, *J* = 5.6 Hz, 2H, NHCH_2C(O)), 3.73 (s, 3H, OCH_3). **^{13}C NMR** (101 MHz, $CDCl_3$, 298 K) δ 171.4 (Cq), 156.1 (Cq), 142.6 (Cq), 129.6 (CH), 127.6 (CH), 126.6 (CH), 52.4 (CH_3), 42.6 (CH_2). **IR** (ν_{max}, cm^{-1}) 3365 (m), 3063 (m), 1758 (s), 1650 (s), 1508 (s), 1488 (s), 1332 (m), 1204 (s), 1183 (s), 760 (s), 700 (s). **HRMS** (ESI/QTOF) m/z: $[M + H]^+$ Calcd for $C_{16}H_{17}N_2O_3^+$ 285.1234; Found 285.1238.

Methyl (*tert*-butoxycarbonyl)glycyl-*L*-prolinate (46)

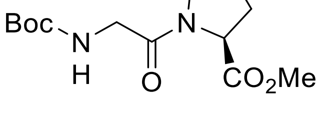

Prepared according to the general procedure **A** from (*tert*-butoxycarbonyl)glycyl-*L*-proline (0.82 g, 3.0 mmol, 1.0 equiv), iodomethane (0.75 mL, 12 mmol, 4.0 equiv), potassium carbonate (0.83 g, 6.0 mmol, 2.0 equiv) in *N*,*N*-dimethylformamide (2 x 4.7 mL). Methyl (*tert*-

[10] K. C. Nadimpally, K. Thalluri, N. B. Palakurthy, A. Saha, B. Mandal, *Tetrahedron Lett.* **2011**, *52*, 2579–2582.

butoxycarbonyl)glycyl-*L*-prolinate **46** (0.70 g, 2.4 mmol, 81%) was obtained as a yellow sticky oil.

^{1}H NMR (400 MHz, $CDCl_3$, 298 K, mixture of two rotamers) δ 5.40 (br s, 1H, N*H*), 4.50 (dd, *J* = 8.7, 3.5 Hz, 0.83H, $NCH_2CH_2CH_2C\mathit{H}_{rotamermaj}C(O)$), 4.37 (dd, *J* = 8.1, 2.8 Hz, 0.17H, $NCH_2CH_2CH_2C\mathit{H}_{rotamermin}C(O)$), 4.02 – 3.83 (m, 2H, $NHC\mathit{H_2}C(O)$), 3.75 (s, 0.55H, $OC\mathit{H}_{3rotamermin}$), 3.72 (s, 2.45H, $OC\mathit{H}_{3rotamermaj}$), 3.65 – 3.53 (m, 1H, $NC\mathit{H}HCH_2CH_2CHC(O)$), 3.48 – 3.42 (m, 1H, $NCH\mathit{H}CH_2CH_2CHC(O)$), 2.32 – 1.80 (m, 4H, $NCH_2C\mathit{H_2}C\mathit{H_2}CHC(O)$), 1.42 (s, 9H, CH_{3Boc}). **^{13}C NMR** (101 MHz, $CDCl_3$, 298 K, mixture two of rotamers, signals not fully resolved) δ 172.5 ($Cq_{rotamermaj}$), 172.0 ($Cq_{rotamermin}$), 167.7 ($Cq_{rotamermin}$), 167.5 ($Cq_{rotamermaj}$), 155.9 (Cq), 79.7 (Cq), 59.0 ($CH_{rotamermaj}$), 58.6 ($CH_{rotamermin}$), 52.9 ($CH_{3rotamermin}$), 52.5 ($CH_{3rotamermaj}$), 46.8 ($CH_{2rotamermin}$), 46.0 ($CH_{2rotamermaj}$), 43.1 ($CH_{2rotamermaj}$), 42.9 ($CH_{2rotamermin}$), 31.5 ($CH_{2rotamermin}$), 29.1 ($CH_{2rotamermaj}$), 28.5 (CH_3), 24.8 ($CH_{2rotamermaj}$), 22.3 ($CH_{2rotamermin}$).

NMR spectra are in agreement with the reported data.[11]

Methyl (tert-butoxycarbonyl)-*L*-alaninate (47)

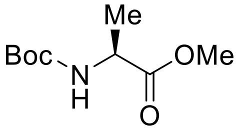

Prepared according to the general procedure **A** from (*tert*-butoxycarbonyl)-*L*-alanine (0.38 g, 2.0 mmol, 1.0 equiv), iodomethane (0.50 mL, 8.0 mmol, 4.0 equiv), potassium carbonate (0.55 g, 4.0 mmol, 2.0 equiv) in *N*,*N*-dimethylformamide (2 x 3.2 mL). Methyl (tert-butoxycarbonyl)-*L*-alaninate **47** (0.36 g, 1.8 mmol, 89%) was obtained as a yellow liquid.

^{1}H NMR (400 MHz, $CDCl_3$, 298 K) δ 5.04 (s, 1H, N*H*), 4.40 – 4.19 (m, 1H, NHC*H*C(O)), 3.74 (s, 3H, $OC\mathit{H_3}$), 1.44 (s, 9H, $C\mathit{H}_{3Boc}$), 1.37 (d, *J* = 7.2 Hz, 3H, $C\mathit{H}_{3Ala}$). **^{13}C NMR** (101 MHz $CDCl_3$, 298 K) δ 174.0 (Cq), 155.2 (Cq), 80.0 (Cq), 52.5 (CH_3), 49.3 (CH), 28.5 (CH_3), 18.8 (CH_3).

NMR spectra are in agreement with the reported data.[12]

tert-Butyl (*S*)-2-(((*S*)-1-methoxy-1-oxopropan-2-yl)carbamoyl)pyrrolidine-1-carboxylate (48)

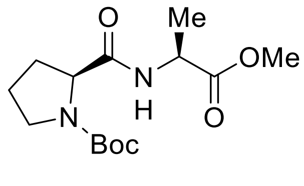

[11] E. Morisset, A. Chardon, J. Rouden, J. Blanchet, *Eur. J. Org. Chem.* **2020**, 388–392.
[12] M. Nappi, C. He, W. G. Whitehurst, B. G. N. Chappell, M. J. Gaunt, *Angew. Chem., Int. Ed.* **2018**, *57*, 3178–3182.

Prepared according to the general procedure **D** from *tert*-butoxycarbonyl)-*L*-proline (0.65 g, 3.0 mmol, 1.0 equiv), methyl *L*-alaninate hydrochloride (0.42 g, 3.0 mmol, 1.0 equiv), 1-(3-dimethylaminopropyl)-3-ethylcarbodiimide hydrochloride (0.58 g, 3.0 mmol, 1.0 equiv), 1-hydroxybenzotriazole hydrate (0.51 g, 3.3 mmol, 1.1 equiv) and *N*,*N*-diisopropylethylamine (1.6 mL, 9.0 mmol, 3.0 equiv) in dichloromethane (19.0 mL). The crude mixture was purified by flash chromatography on silica gel using a gradient from pentane to pentane/ethyl acetate 5:5 as eluent to afford *tert*-butyl (*S*)-2-(((*S*)-1-methoxy-1-oxopropan-2-yl)carbamoyl)pyrrolidine-1-carboxylate **48** (0.80 g, 2.7 mmol, 89%) as a white solid.

Rf (pentane/ethyl acetate 5:5): 0.22. **^{1}H NMR** (400 MHz, $CDCl_3$, 298 K, mixture of two rotamers) δ 7.31 (br s, 0.5H, N*H*CHC(O)), 6.55 (br s, 0.5H, N*H*CHC(O)), 4.54 (app. br s, 1H, NHC*H*C(O)), 4.29 – 4.21 (m, 1H, $NHCH_2CH_2CH_2CH(O)$), s (s, 3H, OCH_3), 3.45 – 3.33 (m, 2H, $NHCH_2CH_2CH_2CH(O)$), 2.21 (app. br m, 1H, $NHCH_2CHHCH_2CH(O)$), 1.87 (app. br s, 3H, $NHCH_2CH_2CH_2CH(O)$ + $NHCH_2CHHCH_2CH(O)$), 1.46 (s, 9H, CH_{3Boc}), 1.42 – 1.29 (m, 3H, CH_{3Ala}). **^{13}C NMR** (101 MHz, $CDCl_3$, 298 K, mixture of two rotamers, signals not fully resolved) δ 173.3 (Cq), 172.2 (Cq), 171.9 (Cq), 155.8 (Cq), 154.8 (Cq), 80.8 (Cq), 80.6 (Cq), 61.1 (CH), 60.0 (CH), 52.5 (CH_3), 48.1 (CH), 47.2 (CH_2), 31.0 (CH_2), 28.5 (CH_3), 24.7 (CH_2), 23.9 (CH_2), 18.8 (CH_3), 18.4 (CH_3).

NMR spectra are in agreement with the reported data.[13]

***tert*-Butyl (*S*)-2-(((*S*)-1-methoxy-3-methyl-1-oxobutan-2-yl)carbamoyl)pyrrolidine-1-carboxylate (49)**

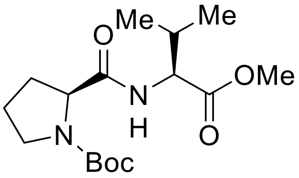

Prepared according to the general procedure **D** from *tert*-butoxycarbonyl)-*L*-proline (0.65 g, 3.0 mmol, 1.0 equiv), methyl *L*-valinate hydrochloride (0.50 g, 3.0 mmol, 1.0 equiv), 1-(3-dimethylaminopropyl)-3-ethylcarbodiimide hydrochloride (0.58 g, 3.0 mmol, 1.0 equiv), 1-hydroxybenzotriazole hydrate (0.51 g, 3.3 mmol, 1.1 equiv) and *N*,*N*-diisopropylethylamine (1.6 mL, 9.0 mmol, 3.0 equiv) in dichloromethane (19.0 mL). The crude mixture was purified by flash chromatography on silica gel using a gradient from pentane to pentane/ethyl acetate 7:3 as eluent to afford *tert*-butyl (*S*)-2-(((*S*)-1-methoxy-3-methyl-1-oxobutan-2-yl)carbamoyl)pyrrolidine-1-carboxylate **49** (0.79 g, 2.4 mmol, 80%) as a yellowish solid.

Rf (pentane/ethyl acetate 7:3): 0.25. **^{1}H NMR** (400 MHz, $CDCl_3$, 298 K, complex mixture of rotamers) δ 7.49 (s, 0.55H, N*H*), 6.52 (s, 0.45H, N*H*), 4.63 – 4.41 (m, 1H, NHC*H*C(O)), 4.48 – 4.40 (m, 1H, $NCH_2CH_2CH_2CHC(O)$), 3.72 (s, 3H, OCH_3), 3.57 – 3.21 (m, 2H, $NCH_2CH_2CH_2CHC(O)$), 2.48 – 2.04 (m, 2H, $CH_{Val}(CH_3)_2$ + CHH_{Pro}), 2.00 – 1.69 (m, 3H, CH_{2Pro}), 1.46 (s, 9H, CH_{3Boc}), 0.95

[13] R. M. de Figueiredo, J.-S. Suppo, C. Midrier, J.-M. Campagne, *Adv. Synth. Catal.* **2007**, *359*, 1963–1968.

– 0.81 (m, 6H, CH_{3Val}). **^{13}C NMR** (101 MHz, $CDCl_3$, 298 K, complex mixture of rotamers, signals not fully resolved) δ 172.7 (Cq), 172.3 (Cq), 172.0 (Cq), 156.0 (Cq), 154.8 (Cq), 81.0 (Cq), 80.5 (Cq), 61.4 (CH), 59.8 (CH), 57.4 (CH), 56.9 (CH), 52.2 (CH_3), 47.1 (CH_2), 31.6 (CH), 31.2 (CH), 28.5 (CH_3), 27.7 (CH_2), 24.8 (CH_2), 23.9 (CH_2), 19.2 (CH_3), 17.8 (CH_3).

NMR spectra are in agreement with the reported data.[14]

tert-Butyl (_S_)-2-(((_S_)-1-(_tert_-butoxy)-4-methyl-1-oxopentan-2-yl)carbamoyl)pyrrolidine-1-carboxylate (50)

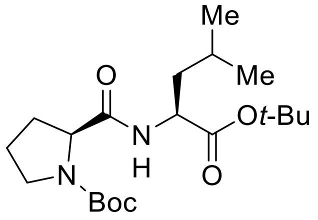

Prepared according to the general procedure **D** from *tert*-butoxycarbonyl)-*L*-proline (0.65 g, 3.0 mmol, 1.0 equiv), *tert*-butyl *L*-leucinate hydrochloride (0.67 g, 3.0 mmol, 1.0 equiv), 1-(3-dimethylaminopropyl)-3-ethylcarbodiimide hydrochloride (0.58 g, 3.0 mmol, 1.0 equiv), 1-hydroxybenzotriazole hydrate (0.51 g, 3.3 mmol, 1.1 equiv) and *N*,*N*-diisopropylethylamine (1.6 mL, 9.0 mmol, 3.0 equiv) in dichloromethane (19.0 mL). The crude mixture was purified by flash chromatography on silica gel using a gradient from pentane to pentane/ethyl acetate 8:2 as eluent to afford *tert*-butyl (*S*)-2-(((*S*)-1-(*tert*-butoxy)-4-methyl-1-oxopentan-2-yl)carbamoyl)pyrrolidine-1-carboxylate **50** (0.82 g, 2.1 mmol, 71%) as a yellowish solid.

Rf (pentane/ethyl acetate 8:2): 0.24. **Mp**: 90.3 – 97.5 °C. **^{1}H NMR** (400 MHz, MeOD-*d*$_4$, 298 K, mixture of rotamers) δ 4.29 (dd, *J* = 8.8, 6.2 Hz, 1H, NHC*H*$CH_2CH(CH_3)_3$), 4.23 (dd, *J* = 8.6, 3.9 Hz, 1H, $NCH_2CH_2CH_2$C*H*C(O)), 3.57 – 3.45 (m, 1H, NC*H*HCH_2CH_2CHC(O)), 3.45 – 3.34 (m, 1H, NCH*H*CH_2CH_2CHC(O)), 2.34 – 2.08 (m, 1H, NCH_2CH_2C*H*HCHC(O)), 2.07 – 1.81 (m, 3H, NCH_2C$H_2$$CH_2$CHC(O) + NCH_2CH_2CH*H*CHC(O)), 1.81 – 1.66 (m, 1H, NHCHCH_2C*H*$(CH_3)_2$), 1.66 – 1.52 (m, 2H, NHCHC$H_2$$CH(CH_3)_2$), 1.46 (s, 12H, CH_{3Boc} + $CH_{3OtBu rotamermaj}$), 1.42 (s, 6H, CH_{3Boc} + $CH_{3OtBu rotamermin}$), 0.97 (d, *J* = 6.6 Hz, 3H, CH_{3Val}), 0.93 (d, *J* = 6.5 Hz, 3H, CH_{3Val}). **^{13}C NMR** (101 MHz, MeOD-*d*$_4$, 298 K, mixture of rotamers, signals not fully resolved) δ 175.7 ($Cq_{rotamermaj}$), 175.2 ($Cq_{rotamermin}$), 173.4 ($Cq_{rotamermin}$), 173.3 ($Cq_{rotamermaj}$), 156.3 ($Cq_{rotamermin}$), 156.0 ($Cq_{rotamermaj}$), 82.6 (Cq), 81.4 ($Cq_{rotamermaj}$), 81.1 ($Cq_{rotamermin}$), 61.3 ($CH_{rotamermaj}$), 60.9 ($CH_{rotamermin}$), 53.1 ($CH_{rotamermin}$), 52.9 ($CH_{rotamermaj}$), 48.2 ($CH_{2rotamermin}$), 47.9 ($CH_{2rotamermaj}$), 41.5 (CH_2), 32.5 ($CH_{2rotamermaj}$), 31.3 ($CH_{2rotamermin}$), 28.7 ($CH_{3rotamermin}$), 28.6 ($CH_{3rotamermaj}$), 28.2 (CH_3), 26.0 (CH), 25.3 ($CH_{2rotamermin}$), 24.5 ($CH_{2rotamermaj}$), 23.3 (CH_3), 22.0 ($CH_{3rotamermin}$), 21.9 ($CH_{3rotamermaj}$). **IR** (ν_{max}, cm^{-1}) 2966 (w), 2873 (w), 1736 (m), 1693 (s), 1655 (s), 1396 (s), 1161 (s), 1126 (m). **HRMS** (ESI/QTOF) m/z: $[M + Na]^+$ Calcd for $C_{20}H_{36}N_2NaO_5^+$ 407.2516; Found 407.2512.

[14] W. Wu, Z. Zhang, L. S. Liebeskind, *J. Am. Chem. Soc.* **2011,** *133*, 14256–14259.

tert-Butyl (_S_)-2-(((_S_)-1-methoxy-1-oxo-3-phenylpropan-2-yl)carbamoyl)pyrrolidine-1-carboxylate (51)

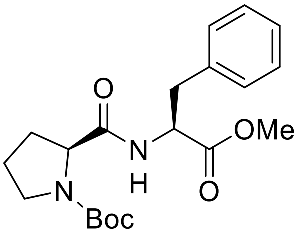

Prepared according to the general procedure **D** from *tert*-butoxycarbonyl)-*L*-proline (0.65 g, 3.0 mmol, 1.0 equiv), methyl *L*-phenylalaninate hydrochloride (0.65 g, 3.0 mmol, 1.0 equiv), 1-(3-dimethylaminopropyl)-3-ethylcarbodiimide hydrochloride (0.58 g, 3.0 mmol, 1.0 equiv), 1-hydroxybenzotriazole hydrate (0.51 g, 3.3 mmol, 1.1 equiv) and *N*,*N*-diisopropylethylamine (1.6 mL, 9.0 mmol, 3.0 equiv) in dichloromethane (19.0 mL). The crude mixture was purified by flash chromatography on silica gel using a gradient from pentane to pentane/ethyl acetate 1:1 as eluent to afford *tert*-butyl (*S*)-2-(((*S*)-1-methoxy-1-oxo-3-phenylpropan-2-yl)carbamoyl)pyrrolidine-1-carboxylate **51** (0.87 g, 2.3 mmol, 77%) as a yellow sticky oil.

Rf (pentane/ethyl acetate 1:1): 0.52. **^{1}H NMR** (400 MHz, $CDCl_3$, 298 K, mixture of two rotamers) δ 7.60 – 7.32 (m, 3H, Ar*H*), 7.24 (d, *J* = 6.5 Hz, 2H, Ar*H*), 6.60 (br s, 1H, N*H*CHC(O)), 5.01 (app. br s, 1H, NHC*H*C(O)), 4.39 (app. br d, 1H, $NCH_2CH_2CH_2CHC(O)$), 3.87 (s, 3H, OCH_3), 3.65 – 3.39 (m, 2H, $NCH_2CH_2CH_2CHC(O)$), 3.34 (dd, *J* = 13.9, 5.7 Hz, 1H, $CHH_{Phe}Ph$), 3.16 (dd, *J* = 13.9, 7.0 Hz, 1H, $CHH_{Phe}Ph$), 2.42 (app. br s, 1H, $NHCH_2CH_2CHHCH(O)$), 2.15 (app. br d, 1H, $NHCH_2CH_2CHHCH(O)$), 1.91 (app br s, 2H, $NHCH_2CH_2CH_2CH(O)$), 1.57 (s, 9H, CH_{3Boc}). **^{13}C NMR** (101 MHz, $CDCl_3$, 298 K, mixture of two rotamers, signals not fully resolved) δ 172.2 ($Cq_{rotamermin}$), 171.8 ($Cq_{rotamermaj}$), 155.8 (Cq), 154.7 (Cq), 136.3 (Cq), 136.0 (Cq), 129.3 (CH), 128.6 (CH), 127.2 (CH), 80.9 ($Cq_{rotamermaj}$), 80.5 ($Cq_{rotamermin}$), 61.2 (CH), 60.1 (CH), 53.4 (CH), 52.8 (CH), 52.4 (CH_3), 47.1 (CH_2), 38.2 (CH_2), 30.8 (CH_2), 28.4 (CH_3), 24.6 (CH_2), 23.5 (CH_2).

NMR spectra are in agreement with the reported data.[15]

tert-Butyl (_S_)-2-(((_S_)-3-((tert-butyldimethylsilyl)oxy)-1-methoxy-1-oxopropan-2-yl)carbamoyl)pyrrolidine-1-carboxylate (52)

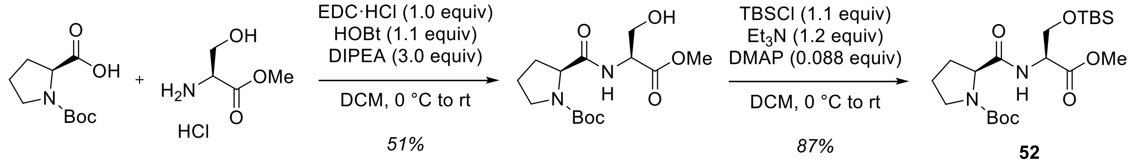

tert-butyl (S)-2-(((S)-3-hydroxy-1-methoxy-1-oxopropan-2-yl)carbamoyl)pyrrolidine-1-carboxylate was prepared according to the general procedure **D** from *tert*-butoxycarbonyl)-*L*-proline (0.65 g, 3.0 mmol, 1.0 equiv), methyl *L*-serinate hydrochloride (0.47 g, 3.0 mmol, 1.0

[15] G. K. Min, D. Hernández, A. T. Lindhardt, T. Skrydstrup, *Org. Lett.* **2010**, *12*, 4716–4719.

equiv), 1-(3-dimethylaminopropyl)-3-ethylcarbodiimide hydrochloride (0.58 g, 3.0 mmol, 1.0 equiv), 1-hydroxybenzotriazole hydrate (0.51 g, 3.3 mmol, 1.1 equiv) and *N*,*N*-diisopropylethylamine (1.6 mL, 9.0 mmol, 3.0 equiv) in dichloromethane (19.0 mL). The crude mixture was purified by flash chromatography on silica gel using a gradient from dichloromethane to dichloromethane/methanol 96:4 as eluent to afford *tert*-butyl (S)-2-(((S)-3-hydroxy-1-methoxy-1-oxopropan-2-yl)carbamoyl)pyrrolidine-1-carboxylate (0.48 g, 1.5 mmol, 51%) as a yellowish sticky oil. **Rf** (dichloromethane/methanol 96:4): 0.4. **^{1}H NMR** (400 MHz, MeOD-d_4, 298 K, mixture of rotamers) δ 4.52 (t, *J* = 4.4 Hz, 1H), 4.27 (app. br s, 1H), 4.02 – 3.88 (m, 1H), 3.88 – 3.75 (m, 1H), 3.74 (s, 3H, OCH_3), 3.59 – 3.46 (m, 1H, NC*H*HCH$_2$CH$_2$CHC(O)), 3.47 – 3.35 (m, 1H, NCH*H*CH$_2$CH$_2$CHC(O)), 2.38 – 2.11 (m, 1H, C$H_{2\text{Pro}}$), 2.10 – 1.74 (m, 3H, C$H_{2\text{Pro}}$), 1.47 (s, 3H, C$H_{3\text{Boc}}$), 1.43 (s, 5H, C$H_{3\text{Boc}}$). The NMR spectrum is in agreement with the reported data.[16]

tert-butyl (*S*)-2-(((S)-3-hydroxy-1-methoxy-1-oxopropan-2-yl)carbamoyl)pyrrolidine-1-carboxylate (0.48 g, 1.5 mmol, 1.0 equiv) was then dissolved in 1.8 mL of anhydrous dichloromethane. The reaction mixture was cooled down to 0 °C and *tert*-butyldimethylsilyl chloride (0.26 g, 1.7 mmol, 1.1 equiv), 4-(dimethylamino)pyridine (16 mg, 0.13 mmol, 0.088 equiv) and triethylamine (0.25 mL, 1.8 mmol, 1.2 equiv) were added. The cooling bath was removed and the reaction was stirred at room temperature 19 hours. The reaction mixture was then quenched with saturated sodium hydrogen carbonate and extracted twice with dichloromethane. The combined organic layers were washed with ammonium chloride and brine, dried over anhydrous magnesium sulfate, filtered and concentrated under reduced pressure. The crude product was purified by flash chromatography on silica gel using a gradient from pentane to pentane/ethyl acetate 8:2 to afford *tert*-butyl (*S*)-2-(((*S*)-3-((tert-butyldimethylsilyl)oxy)-1-methoxy-1-oxopropan-2-yl)carbamoyl)pyrrolidine-1-carboxylate **52** as a clear sticky oil (0.57 g, 1.3 mmol, 87%). **Rf** (pentane/ethyl acetate 8:2): = 0.19.

^{1}H NMR (400 MHz, $CDCl_3$, 298 K, mixture of two rotamers) δ 7.29 (br s, 0.45H, N*H*), 6.81 (br s, 0.55H, N*H*), 4.62 (app. br s, 1H, C*H*CH$_2$OTBS), 4.47 – 4.17 (m, 1H, NCH$_2$CH$_2$CH$_2$C*H*C(O)), 4.10 – 3.92 (m, 1H, CHC*H*HOTBS), 3.82 – 3.74 (m, 1H, CHCH*H*OTBS), 3.71 (s, 3H, OCH_3), 3.57 – 3.25 (m, 2H, NCH_2CH$_2$CH$_2$CHC(O)), 2.36 – 2.06 (m, 2H, NCH$_2$CH$_2$CH_2CHC(O)), 1.98 – 1.70 (m, 2H, NCH$_2$CH_2CH$_2$CHC(O)), 1.45 (s, 9H, C$H_{3\text{Boc}}$), 0.83 (s, 9H, $t\text{Bu}_{\text{TBS}}$), -0.00 (s, 4H, C$H_{3\text{TBS}}$), -0.01 (s, 2H, C$H_{3\text{TBS}}$). **^{13}C NMR** (101 MHz, $CDCl_3$, 298 K, mixture of two rotamers, signals not fully resolved) δ 172.6 ($Cq_{rotamermaj}$), 172.2 ($Cq_{rotamermin}$), 170.7 ($Cq_{rotamermin}$), 170.5 ($Cq_{rotamermaj}$), 155.5 ($Cq_{rotamermin}$), 154.7 ($Cq_{rotamermaj}$), 80.7 ($Cq_{rotamermaj}$), 80.4 ($Cq_{rotamermin}$), 63.7 (CH_2), 61.2 ($CH_{rotamermaj}$), 60.3 ($CH_{rotamermin}$), 54.5 ($CH_{rotamermin}$), 54.2 ($CH_{rotamermaj}$), 52.4 (CH_3), 47.1 (CH_2), 31.2 ($CH_{2rotamermaj}$), 28.9 ($CH_{2rotamermin}$), 28.4 (CH_3), 25.8 (CH_3), 24.5 ($CH_{2rotamermin}$), 23.7 ($CH_{2rotamermaj}$), 18.2 (Cq), -5.5 ($CH_{3rotamermaj}$), -5.6 ($CH_{3rotamermin}$).

NMR spectra are in agreement with the reported data.[16]

[16] Z. Chen, T. Ye, *New J. Chem.* **2006**, *30*, 518–520.

di-*tert*-Butyl (*tert*-butoxycarbonyl)-*L*-prolyl-*L*-glutamate (53)

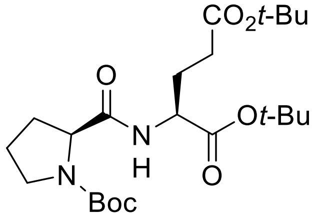

Prepared according to the general procedure **D** from *tert*-butoxycarbonyl)-*L*-proline (0.65 g, 3.0 mmol, 1.0 equiv), di-*tert*-butyl L-glutamate hydrochloride (0.93 g, 3.0 mmol, 1.0 equiv), 1-(3-dimethylaminopropyl)-3-ethylcarbodiimide hydrochloride (0.58 g, 3.0 mmol, 1.0 equiv), 1-hydroxybenzotriazole hydrate (0.51 g, 3.3 mmol, 1.1 equiv) and *N*,*N*-diisopropylethylamine (1.6 mL, 9.0 mmol, 3.0 equiv) in dichloromethane (19.0 mL). The crude mixture was purified by flash chromatography on silica gel using a gradient from pentane to pentane/ethyl acetate 7:3 as eluent to afford di-*tert*-butyl (*tert*-butoxycarbonyl)-*L*-prolyl-*L*-glutamate **53** (1.2 g, 2.5 mmol, 84%) as a white solid.

Rf (pentane/ethyl acetate 8:2): 0.49. **Mp**: 88.0–92.3 °C. **^{1}H NMR** (400 MHz, MeOD-d_4, 298 K, mixture of two rotamers) δ 4.29 (dd, *J* = 9.2, 5.1 Hz, 1H, NHCH_{Glu}C(O)), 4.22 (dd, *J* = 8.6, 3.7 Hz, 1H, $NCH_2CH_2CH_2C\mathit{H}C(O)$), 3.60 – 3.45 (m, 1H, NCH_{Pro}HCH_2CH_2CHC(O)), 3.46 – 3.35 (m, 1H, NCH$H_{Pro}$$CH_2CH_2$CHC(O)), 2.43 – 2.30 (m, 2H, NCH_2CH_2CHH$_{Pro}$CHC(O) + NHCHCH_2CHH$_{Glu}$C(O)O*t*-Bu), 2.30 – 2.04 (m, 2H, NHCHCHH$_{Glu}$$CH_2$C(O)O*t*-Bu + NHCHC$H_2CHH_{Glu}$C(O)O*t*-Bu), 2.04 – 1.81 (m, 4H, NCH_2C$H_{2Pro}$$CH_2$CHC(O) + NHCHCH$H_{Glu}$$CH_2$C(O)O*t*-Bu + NCH_2CH_2CHH_{Pro}CHC(O)), 1.51 – 1.38 (m, 27H, C$H_{3Ot\text{-}Bu}$). **^{13}C NMR** (101 MHz, MeOD-d_4, 298 K, mixture of two rotamers, signals not fully resolved) δ 175.7 (Cq), 175.3 (Cq), 173.9 (Cq), 173.5 (Cq), 172.3 (Cq), 172.2 (Cq), 156.3 (Cq), 156.0 (Cq), 82.9 (Cq), 81.9 (Cq), 81.7 (Cq), 81.5 (Cq), 81.2 (Cq), 61.4 (CH), 61.1 (CH), 53.7 (CH), 48.2 (CH_r), 47.9 (CH_2), 32.6 (CH_2), 32.5 (CH_2), 31.3 (CH_2), 28.8 (CH_3), 28.4 (CH_3), 28.3 (CH_3), 27.8 (CH_2), 25.4 (CH_2), 24.5 (CH_2). **IR** (ν_{max}, cm^{-1}) 3329 (w), 2978 (w), 2935 (w), 1732 (m), 1666 (m), 1419 (m), 1149 (s). **HRMS** (ESI/QTOF) m/z: [M + Na]$^+$ Calcd for $C_{23}H_{40}N_2NaO_7^+$ 479.2728; Found 479.2732.

tert-Butyl (*S*)-2-(((*S*)-6-(((benzyloxy)carbonyl)amino)-1-methoxy-1-oxohexan-2-yl)carbamoyl)pyrrolidine-1-carboxylate (54)

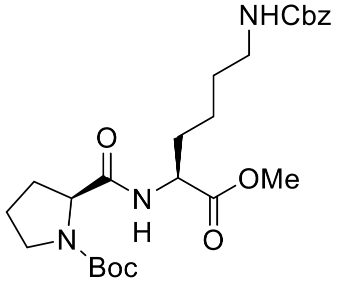

Prepared according to the general procedure **D** from *tert*-butoxycarbonyl)-*L*-proline (0.65 g, 3.0 mmol, 1.0 equiv), methyl *N*-((benzyloxy)carbonyl)-*L*-lysinate hydrochloride (0.99 g, 3.0

mmol, 1.0 equiv), 1-(3-dimethylaminopropyl)-3-ethylcarbodiimide hydrochloride (0.58 g, 3.0 mmol, 1.0 equiv), 1-hydroxybenzotriazole hydrate (0.51 g, 3.3 mmol, 1.1 equiv) and *N*,*N*-diisopropylethylamine (1.6 mL, 9.0 mmol, 3.0 equiv) in dichloromethane (19.0 mL). The crude mixture was purified by flash chromatography on silica gel using a gradient from dichloromethane to dichloromethane/methanol 96:4 as eluent to afford *tert*-butyl (*S*)-2-(((*S*)-6-(((benzyloxy)carbonyl)amino)-1-methoxy-1-oxohexan-2-yl)carbamoyl)pyrrolidine-1-carboxylate **54** (1.5 g, 3.0 mmol, 99%) as a yellow sticky oil.

Rf (dichloromethane/methanol 96:4): 0.53. **^{1}H NMR** (400 MHz, MeOD-d_4, 298 K, mixture of two rotamers) δ 7.41 – 7.23 (m, 5H, Ar*H*), 5.17 – 4.99 (m, 2H, OCH_2Ph), 4.45 – 4.32 (m, 1H, NHCH_{Lys}C(O)), 4.28 – 4.17 (m, 1H, $NCH_2CH_2CH_2C$*H*C(O)), 3.70 (s, 3H, OCH_3), 3.56 – 3.45 (m, 1H, NC*H*HCH_2CH_2CHC(O)), 3.44 – 3.33 (m, 1H, NCH*H*CH_2CH_2CHC(O)), 3.11 (t, *J* = 6.7 Hz, 2H, $CH_2CH_2CH_2CH_{2Lys}$NHCBz), 2.28 – 2.12 (m, 1H, NCH_2CH_2C*H*HCHC(O)), 2.05 – 1.78 (m, 4H, NCH_2CH_2CH*H*CHC(O) + $NCH_2CH_2CH_2$CHC(O) + C*H*$H_{Lys}CH_2CH_2CH_2$NHCBz), 1.77 – 1.68 (m, 1H, CH$H_{Lys}CH_2CH_2CH_2$NHCBz), 1.54 – 1.41 (m, 13H, $CH_2CH_2CH_{2Lys}CH_2$NHCBz + OCH_{3Boc}). **^{13}C NMR** (101 MHz, MeOD-d_4, 298 K, mixture of two rotamers, signals not fully resolved) δ 175.9 ($Cq_{rotamermaj}$), 175.4 ($Cq_{rotamermin}$), 174.1 ($Cq_{rotamermin}$), 173.9 ($Cq_{rotamermaj}$), 158.9 (Cq), 156.3 ($Cq_{rotamermin}$), 156.0 ($Cq_{rotamermaj}$), 138.4 (Cq), 129.5 (CH), 129.0 (CH), 128.8 (CH), 81.4 ($Cq_{rotamermaj}$), 81.2 ($Cq_{rotamermin}$), 67.7 ($CH_{2rotamermin}$), 67.3 ($CH_{2rotamermaj}$), 61.4 ($CH_{rotamermaj}$), 61.0 ($CH_{rotamermin}$), 53.7 ($CH_{rotamermaj}$), 53.6 ($CH_{rotamermin}$), 52.6 (CH_3), 48.3 ($CH_{2rotamermin}$), 47.9 ($CH_{2rotamermaj}$), 42.1 ($CH_{2rotamermin}$), 41.4 ($CH_{2rotamermaj}$), 32.4 (CH_2), 32.0 (CH_2), 31.3 (CH_2), 30.4 ($CH_{2rotamermaj}$), 30.2 ($CH_{2rotamermin}$), 28.7 ($CH_{3rotamermin}$), 28.7 ($CH_{3rotamermaj}$), 25.3 ($CH_{2rotamermin}$), 24.5 ($CH_{2rotamermaj}$), 24.2 ($CH_{2rotamermaj}$), 23.9 ($CH_{2rotamermin}$). **IR** (ν_{max}, cm^{-1}) 3316 (w), 2951 (m), 2888 (m), 2480 (m), 1742 (s), 1680 (s), 1428 (s), 1402 (s), 1165 (s), 735 (m), 698 (m). **HRMS** (ESI/QTOF) m/z: $[M + Na]^+$ Calcd for $C_{25}H_{37}N_3NaO_7^+$ 514.2524; Found 514.2526.

Methyl (*tert*-butoxycarbonyl)glycyl-*L*-valinate (55)

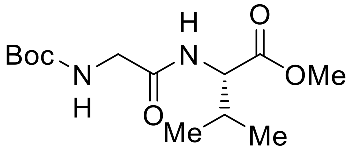

Prepared according to the general procedure **D** from (*tert*-butoxycarbonyl)glycine (0.53 g, 3.0 mmol, 1.0 equiv), methyl *L*-valinate hydrochloride (0.50 g, 3.0 mmol, 1.0 equiv), 1-(3-dimethylaminopropyl)-3-ethylcarbodiimide hydrochloride (0.58 g, 3.0 mmol, 1.0 equiv), 1-hydroxybenzotriazole hydrate (0.51 g, 3.3 mmol, 1.1 equiv) and *N*,*N*-diisopropylethylamine (1.6 mL, 9.0 mmol, 3.0 equiv) in dichloromethane (19.0 mL). The crude mixture was purified by flash chromatography on silica gel using a gradient from pentane to pentane/ethyl acetate 1:1 as eluent to afford methyl (*tert*-butoxycarbonyl)glycyl-*L*-valinate **55** (0.74 g, 2.5 mmol, 85%) as a yellowish sticky oil.

Rf (pentane/ethyl acetate 1:1): 0.66. **^{1}H NMR** (400 MHz, $CDCl_3$, 298 K) δ 6.65 (br s, 1H, N*H*Boc), 5.21 (br s, 1H, N*H*CH_{Val}C(O)), 4.54 (dd, *J* = 8.9, 4.9 Hz, 1H, NHC*H*$_{Val}$C(O)), 3.82 (qd, *J* = 16.0, 15.2, 4.1 Hz, 2H, BocNHCH_{2Gly}), 3.73 (s, 3H, OCH_3), 2.17 (m, 1H, NHCHC*H*$(CH_3)_2$), 1.45 (s, 9H, CH_{3Boc}), 0.93 (d, *J* = 6.8 Hz, 3H, CH_{3Val}), 0.89 (d, *J* = 6.9 Hz, 3H, CH_{3Val}). **^{13}C NMR** (101 MHz, $CDCl_3$, 298 K) δ 172.4 (Cq), 169.6 (Cq), 156.2 (Cq), 80.5 (Cq), 57.1 (CH), 52.3 (CH_3), 44.6 (CH_2), 31.4 (CH), 28.4 (CH_3), 19.1 (CH_3), 17.8 (CH_3).

NMR spectra are in agreement with the reported data.[17]

***tert*-Butyl (*tert*-butoxycarbonyl)glycyl-*L*-leucinate (56)**

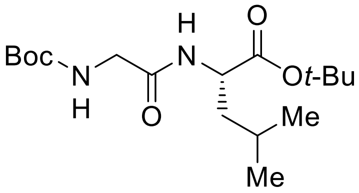

Prepared according to the general procedure **D** from (*tert*-butoxycarbonyl)glycine (0.53 g, 3.0 mmol, 1.0 equiv), *tert*-butyl *L*-leucinate hydrochloride (0.67 g, 3.0 mmol, 1.0 equiv), 1-(3-dimethylaminopropyl)-3-ethylcarbodiimide hydrochloride (0.58 g, 3.0 mmol, 1.0 equiv), 1-hydroxybenzotriazole hydrate (0.51 g, 3.3 mmol, 1.1 equiv) and *N*,*N*-diisopropylethylamine (1.6 mL, 9.0 mmol, 3.0 equiv) in dichloromethane (19.0 mL). The crude mixture was purified by flash chromatography on silica gel using a gradient from pentane to pentane/ethyl acetate 8:2 as eluent to afford *tert*-butyl (*tert*-butoxycarbonyl)glycyl-*L*-leucinate **56** (0.83 g, 2.4 mmol, 81%) as a yellowish sticky oil.

Rf (pentane/ethyl acetate 8:2): 0.21. **^{1}H NMR** (400 MHz, MeOD-*d*$_4$, 298 K) δ 4.34 (br t, *J* = 7.5 Hz, 1H, NHC*H*$_{Leu}$ $CH_2CH(CH_3)_2$), 3.79 – 3.63 (m, 2H, NHCH_{2Gly}C(O)), 1.69 (m, 1H, NHCHCH_2C*H*$_{Leu}$$(CH_3)_2$), 1.62 – 1.54 (m, 2H, NHCHCH_{2Leu}CH $(CH_3)_2$), 1.46 (s, 9H, CH_{3Boc} or C$H_{3t\text{-}Bu}$), 1.45 (s, 9H, CH_{3Boc} or C$H_{3t\text{-}Bu}$), 0.96 (d, *J* = 6.6 Hz, 3H, NHCHCH_2CH(CH_{3Leu})$_2$), 0.92 (d, *J* = 6.5 Hz, 3H, NHCHCH_2CH(CH_{3Leu})$_2$). **^{13}C NMR** (101 MHz, MeOD-*d*$_4$, 298 K) δ 173.4 (Cq), 172.4 (Cq), 158.3 (Cq), 82.8 (Cq), 80.7 (Cq), 52.9 (CH), 44.4 (CH_2), 41.7 (CH_2), 28.7 (CH_3), 28.2 (CH_3), 26.0 (CH), 23.3 (CH_3), 22.0 (CH_3). **IR** (ν_{max}, cm^{-1}) 3329 (w), 2966 (w), 1720 (m), 1674 (m), 1516 (m), 1369 (m), 1257 (m), 1149 (s). **HRMS** (ESI/QTOF) m/z: $[M + Na]^+$ Calcd for $C_{17}H_{32}N_2NaO_5^+$ 367.2203; Found 367.2208.

[17] M. Sayes, A. B. Charette, *Green Chem.* **2017**, *9*, 5060 – 5064.

tert-Butyl (_S_)-2-((2-(((_S_)-1-((2-methoxy-2-oxoethyl)amino)-4-methyl-1-oxopentan-2-yl)amino)-2-oxoethyl)carbamoyl)pyrrolidine-1-carboxylate (57)

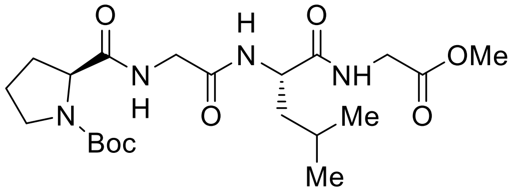

Prepared according to the general procedure **D** from (*tert*-butoxycarbonyl)-*L*-prolylglycine (1.0 g, 3.7 mmol, 1.0 equiv), methyl *L*-leucylglycinate hydrochloride (0.88 g, 3.7 mmol, 1.0 equiv), 1-(3-dimethylaminopropyl)-3-ethylcarbodiimide hydrochloride (0.70 g, 3.7 mmol, 1.0 equiv), 1-hydroxybenzotriazole hydrate (0.63 g, 4.0 mmol, 1.1 equiv) and *N*,*N*-diisopropylethylamine (1.9 mL, 11 mmol, 3.0 equiv) in dichloromethane (23.0 mL). The crude mixture was purified by flash chromatography on silica gel using a gradient from dichloromethane to dichloromethane/methanol 96:4 as eluent to afford *tert*-butyl (*S*)-2-((2-(((*S*)-1-((2-methoxy-2-oxoethyl)amino)-4-methyl-1-oxopentan-2-yl)amino)-2-oxoethyl) carbamoyl)pyrrolidine-1-carboxylate **57** (1.1 g, 2.5 mmol, 67%) as a white solid.

Rf (dichloromethane/methanol 96:4): 0.24. **Mp:** 55.7–72.6 °C. **^{1}H NMR** (400 MHz, MeOD-*d$_4$*, 298 K, complex mixture of rotamers) δ 4.53 – 4.38 (m, 1H, NHCH_{Leu}C(O)), 4.19 (dd, *J* = 8.5, 4.2 Hz, 1H, NCH$_2$CH$_2$CH$_2$C*H*C(O)), 4.02 – 3.78 (m, 4H, CH_{2Gly}), 3.71 (s, 3H, OCH_3), 3.62 – 3.36 (m, 2H, NCH_2CH$_2$CH$_2$CHC(O)), 2.27 – 2.20 (m, 1H, NCH$_2$CH$_2$C*H*HCHC(O)), 2.09 – 1.81 (m, 3H, NCH$_2$CH$_2$CH*H*CHC(O) + NCH$_2$CH_2CH$_2$CHC(O))), 1.78 – 1.56 (m, 3H, NHCHCH_2CH(CH$_3$)$_{2Leu}$ + NHCHCH$_2$C*H*(CH$_3$)$_{2Leu}$), 1.48 (s, 5H, CH_{3Boc}), 1.42 (s, 4H, CH_{3Boc}), 0.97 – 0.61 (m, 6H, CH_{3Leu}). **^{13}C NMR** (101 MHz, MeOD-*d$_4$*, 298 K, complex mixture of rotamers, signals not fully resolved) δ 176.4 (Cq$_{rotamermin}$), 176.3 (Cq$_{rotamermaj}$), 175.3 (Cq$_{rotamermin}$), 175.2 (Cq$_{rotamermaj}$), 171.6 (Cq), 171.5 (Cq), 171.5 (Cq), 171.1 (Cq), 156.7 (Cq$_{rotamermaj}$), 156.0 (Cq$_{rotamermin}$), 81.5 (Cq), 61.9 (CH), 53.1 (CH$_{rotamermaj}$), 52.9 (CH$_{rotamermin}$), 52.6 (CH$_{3rotamermin}$), 52.6 (CH$_{3rotamermaj}$), 47.9 (CH$_2$), 43.8 (CH$_{2rotamermaj}$), 43.4 (CH$_{2rotamermin}$), 42.0 (CH$_2$), 41.8 (CH$_2$), 41.7 (CH$_2$), 32.4 (CH$_{2rotamermin}$), 31.4 (CH$_{2rotamermaj}$), 28.8 (CH$_{3rotamermaj}$), 28.6 (CH$_{3rotamermin}$), 25.8 (CH), 25.5 (CH$_{2rotamermaj}$), 24.6 (CH$_{2rotamermin}$), 23.5 (CH$_3$), 23.4 (CH$_3$), 21.9 (CH$_{3rotamermaj}$). **IR** (ν_{max}, cm^{-1}) 3294 (w), 3057 (w), 2955 (w), 1754 (w), 1650 (s), 1525 (m), 1393 (m), 1162 (m). **HRMS** (ESI/QTOF) m/z: [M + Na]$^+$ Calcd for $C_{21}H_{36}N_4NaO_7^+$ 479.2476; Found 479.2481.

***tert*-Butyl (*S*)-2-(((*S*)-1-(((S)-1-(((*S*)-1-methoxy-4-methyl-1-oxopentan-2-yl)amino)-4-methyl-1-oxopentan-2-yl)amino)-3-methyl-1-oxobutan-2-yl)carbamoyl)pyrrolidine-1-carboxylate (58)**

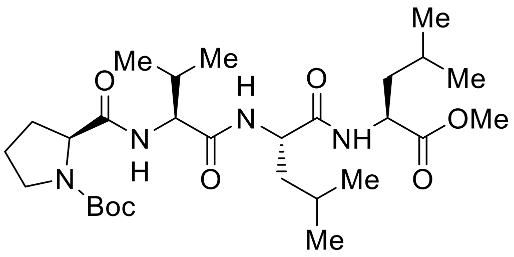

Prepared according to the general procedure **D** from (*tert*-butoxycarbonyl)-*L*-prolyl-*L*-valine (0.53 g, 1.7 mmol, 1.0 equiv), methyl *L*-leucyl-*L*-leucinate hydrochloride (0.50 g, 1.7 mmol, 1.0 equiv), 1-(3-dimethylaminopropyl)-3-ethylcarbodiimide hydrochloride (0.33 g, 1.7 mmol, 1.0 equiv), 1-hydroxybenzotriazole hydrate (0.29 g, 1.9 mmol, 1.1 equiv) and *N*,*N*-diisopropylethylamine (0.90 mL, 5.1 mmol, 3.0 equiv) in dichloromethane (11 mL). The crude mixture was purified by flash chromatography on silica gel using a gradient from dichloromethane to dichloromethane/methanol 96:4 as eluent to afford *tert*-butyl (*S*)-2-(((*S*)-1-(((S)-1-(((*S*)-1-methoxy-4-methyl-1-oxopentan-2-yl)amino)-4-methyl-1-oxopentan-2-yl)amino)-3-methyl-1-oxobutan-2-yl)carbamoyl) pyrrolidine-1-carboxylate **58** (0.82 g, 1.5 mmol, 88%) as a yellowish solid.

Rf (dichloromethane/methanol 96:4): 0.35. **Mp:** 115.5–128.6 °C. **^{1}H NMR** (400 MHz, MeOD-*d*$_4$, 298 K, mixture of two rotamers) δ 4.50 – 4.38 (m, 2H, NHCH_{Leu}C(O) x 2), 4.29 – 4.21 (m, 1H, NCH$_2$CH$_2$CH$_2$C*H*C(O)), 4.17 (d, *J* = 7.3 Hz, 1H, NHCH_{Val}C(O)), 3.70 (s, 3H, OC*H$_3$*), 3.61 – 3.46 (m, 1H, NC*H*HCH$_2$CH$_2$CHC(O)), 3.46 – 3.36 (m, 1H, NCH*H*CH$_2$CH$_2$CHC(O)), 2.31 – 1.81 (m, 5H, NCH$_2$CH$_2$C*H$_2$*CHC(O) + NCH$_2$CH$_2$C*H$_2$*CHC(O) + CH_{Val}(CH$_3$)$_2$), 1.77 – 1.52 (m, 6H, C*H$_2$*C*H*(CH$_3$)$_{2Leu}$ x 2), 1.47 (s, 4H, C$H_{3Bocrotamermin}$), 1.42 (s, 5H, C$H_{3Bocrotamermaj}$), 1.02 – 0.86 (m, 18H, C$H_{3Val+Leu}$). **^{13}C NMR** (101 MHz, MeOD-*d*$_4$, 298 K, mixture of two rotamers, signals not fully resolved) δ 175.4 (Cq), 175.2 (Cq), 175.1 (Cq), 174.5 (Cq), 174.4 (Cq), 173.2 (Cq), 156.6 (Cq), 156.0 (Cq), 81.5 (Cq), 81.4 (Cq), 61.5 (CH), 61.4 (CH), 60.2 (CH), 60.0 (CH), 53.0 (CH or CH$_3$), 52.8 (CH or CH$_3$), 52.6 (CH or CH$_3$), 52.1 (CH or CH$_3$), 52.0 (CH or CH$_3$), 47.9 (CH$_2$), 42.0 (CH$_2$), 41.9 (CH$_2$), 41.4 (CH$_2$), 32.6 (CH$_2$), 32.3 (CH), 32.1 (CH), 31.0 (CH$_2$), 28.7 (CH$_3$), 25.9 (CH), 25.8 (CH), 25.8 (CH), 25.7 (CH), 25.4 (CH$_2$), 24.5 (CH$_2$), 23.4 (CH$_3$), 23.3 (CH$_3$), 22.3 (CH$_3$), 22.1 (CH$_3$), 21.8 (CH$_3$), 21.7 (CH$_3$), 19.9 (CH$_3$), 19.1 (CH$_3$), 18.7 (CH$_3$). **IR** (ν_{max}, cm^{-1}) 3278 (m), 3078 (w), 2957 (m), 1748 (m), 1639 (s), 1549 (m), 1391 (m), 1162 (m). **HRMS** (ESI/QTOF) m/z: [M + Na]$^+$ Calcd for $C_{28}H_{50}N_4NaO_7^+$ 577.3572; Found 577.3572.

Boc-Pro-Val-Leu-Phe-Gly-OMe (59)

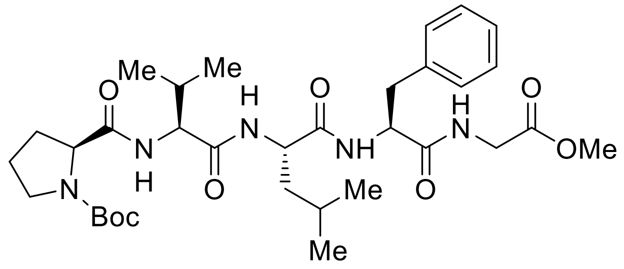

Prepared according to the general procedure **E** using 36 SPPS syringes. After the methylation step, the crude mixture was purified by flash chromatography on a SNAP cartridge KP-SIL 50g column (automatic Biotage system) using a gradient from pentane/ethyl acetate 8:2 to ethyl acetate as eluent to afford Boc-Pro-Val-Leu-Phe-Gly-OMe **59** (1.2 g, 1.9 mmol, 67%) as a white solid.

Rf (pentane/ethyl acetate 2:8): 0.41. **Mp:** 226.1–227.7 °C. **^{1}H NMR** (400 MHz, CD_2Cl_2, 298 K, mixture of two rotamers) δ 7.28 – 7.14 (m, 7H, Ar*H* + NH_{Gly} + NH_{Phe}), 6.49 (d, J = 5.5 Hz, 1H, NH_{Leu}), 4.63 – 4.57 (m, 1H, NHCH_{Phe}C(O)), 4.20 – 4.14 (m, 1H, NHCH_{Leu}C(O)), 4.12 – 4.05 (m, 2.3H, $NCH_2CH_2CH_2CH_{Pro}$C(O) + NHCH_{Val}C(O) + NHC*H*H_{Gly}C(O)), 4.02 (d, J = 6.1 Hz, 0.7H, NHC*H*H_{Gly}C(O)), 3.92 (d, J = 6.1 Hz, 0.7H, NHCHH_{Gly}C(O)), 3.88 (d, J = 6.0 Hz, 0.3H, NHCHH_{Gly}C(O)), 3.72 (s, 3H, OCH_3), 3.56 – 3.49 (m, 2H, N$CH_{2Pro}CH_2CH_2$CHC(O)), 3.41 (dd, J = 14.3, 3.9 Hz, 1H, CHC*H*H_{Phe}Ph), 2.91 (dd, J = 14.3, 11.3 Hz, 1H, C*H*CHH_{Phe}Ph), 2.39 – 2.21 (m, 2H, C$H_{Val}(CH_3)_2$ + NCH_2CH_2C*H*H_{Pro}CHC(O)), 2.10 – 1.98 (m, 1H, NCH_2CH_2CHH_{Pro}CHC(O)), 1.97 – 1.89 (m, 2H, NCH$_2$CH_2CH$_2$CHC(O)), 1.60 (s, 2H, C$H_{3Bocrotamermin}$), 1.52 – 1.58 (m, 1H, CH_2C$H_{Leu}(CH_3)_2$), 1.50 (s, 7H, C$H_{3Bocrotamermaj}$), 1.43 – 1.30 (m, 2H, C$H_{2Leu}CH(CH_3)_2$), 1.00 (d, J = 7.0 Hz, 3H, CH_{3Val}), 0.93 (d, J = 7.0 Hz, 3H, CH_{3Val}), 0.86 (d, J = 6.6 Hz, 3H, CH_{3Leu}), 0.79 (d, J = 6.5 Hz, 3H, CH_{3Leu}). **^{13}C NMR** (101 MHz, CD_2Cl_2, 298 K, mixture of two rotamers, signals not fully resolved) δ 174.5 (Cq), 172.7 (Cq), 172.3 (Cq), 172.1 (Cq), 170.5 (Cq), 156.5 (Cq), 138.8 (Cq), 129.6 (CH), 128.5 (CH), 126.7 (CH), 81.9 (Cq), 62.2 (CH), 60.8 (CH), 54.8 (CH), 52.3 (CH_3), 48.1 (CH_2), 41.6 (CH_2), 40.0 (CH_2), 37.3 (CH_2), 30.5 (CH_2), 29.2 (CH), 28.4 (CH_3), 25.1 (CH_2), 24.9 (CH), 23.2 (CH_3), 20.9 (CH_3), 19.5 (CH_3), 17.7 (CH_3). **IR** (ν_{max}, cm^{-1}) 3665 (w), 2981 (s), 2901 (m), 1631 (w), 1405 (m), 1250 (w), 1228 (w), 1071 (s), 1056 (s), 767 (w), 697 (w). **HRMS** (ESI/QTOF) m/z: $[M + Na]^+$ Calcd for $C_{33}H_{51}N_5NaO_8^+$ 668.3630; Found 668.3634. **LRMS** (ESI) m/z: $[M + H]^+$ Calcd for $C_{33}H_{52}N_5O_8^+$ 646.37; Found 646.7.

Boc-Pro-Val-Pro-Val-Pro-Val-OMe (60)

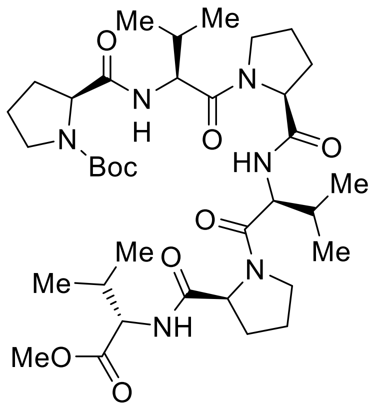

Prepared according to the general procedure **E** using 18 SPPS syringes. After the methylation step, the crude mixture was purified by flash chromatography on a SNAP cartridge KP-SIL 50g column (automatic Biotage system) using a gradient from dichloromethane/methanol 99:01 to dichloromethane/methanol 9:1 as eluent to afford Boc-Pro-Val-Pro-Val-Pro-Val-OMe **60** (0.50 g, 0.70 mmol, 48%) as a white solid.

Rf (dichloromethane/methanol 95:05): 0.43. **Mp:** 109.4–110.1 °C. **^{1}H NMR (400 MHz,** MeOD-*d*$_4$, 298 K, complex mixture of two rotamers) δ 4.55 – 4.39 (m, 4H, $NCH_2CH_2CH_2CH_{Pro}C(O)$ + $NHCH_{Val}C(O)$), 4.30 – 4.25 (m, 2H, $NCH_2CH_2CH_2CH_{Pro}C(O)$ + $NHCH_{Val}C(O)$, 3.89 – 3.84 (m, 2H, $NCH_2CH_2CH_2CHC(O)$), 3.71 (s, 3H, OCH_3), 3.68 – 3.64 (m, 2H, $NCH_2CH_2CH_2CHC(O)$), 3.54 – 3.45 (m, 1H, $NCH_2CH_2CH_2CHC(O)$), 3.44 – 3.35 (m, 1H, $NCH_2CH_2CH_2CHC(O)$), 2.25 – 2.01 (m, 8H, $NCH_2CH_2CH_2CHC(O)$ + $CH_{Val}(CH_3)_2$), 2.01 – 1.82 (m, 7H, $NCH_2CH_2CH_2CHC(O)$ + $CH_{Val}(CH_3)_2$), 1.46 (s, 3H, CH_{3Boc}), 1.42 (s, 6H, CH_{3Boc}), 1.06 – 0.92 (m, 18H, CH_{3Va}). **^{13}C NMR** (101 MHz, MeOD-*d*$_4$, 298 K, complex mixture of two rotamers, signals not fully resolved) δ 175.6 (Cq), 174.5 (Cq), 174.1 (Cq), 173.6 (Cq), 172.5 (Cq), 172.4 (Cq), 156.5 (Cq), 156.0 (Cq), 81.5 (Cq), 81.3 (Cq), 61.5 (CH), 61.3 (CH), 61.2 (CH), 59.3 (CH), 57.9 (CH), 57.9 (CH), 57.7 (CH), 57.5 (CH), 52.4 (CH_3), 47.9 (CH_2), 32.5, 31.9 (CH), 31.0, 30.4 (CH_2), 30.4 (CH_{2j}), 28.7 (CH_3), 26.01 (CH_2), 25.96 (CH_2), 25.4 (CH_2), 24.5 (CH_2), 19.8 (CH_3), 19.5 (CH_3), 19.3 (CH_3), 19.0 (CH_3), 18.7 (CH_3), 18.6 (CH_3); 2 CH_2 are under the MeOD-*d*$_4$ peak (see HSQC). **IR** (ν_{max}, cm^{-1}) 3670 (w), 2983 (s), 2899 (s), 1673 (w), 1624 (w), 1405 (m), 1252 (m), 1228 (m), 1062 (s). **HRMS (ESI/QTOF) m/z**: $[M + Na]^+$ Calcd for $C_{36}H_{60}N_6NaO_9^+$ 743.4314; Found 743.4322. **LRMS** (ESI) m/z: $[M + H]^+$ Calcd for $C_{35}H_{59}N_6O_9^+$ 721.44; Found 721.5.

Boc-Pro-Val-(*t*Bu)Glu-Gly-(*t*Bu)Ser-Phe-OMe (61)

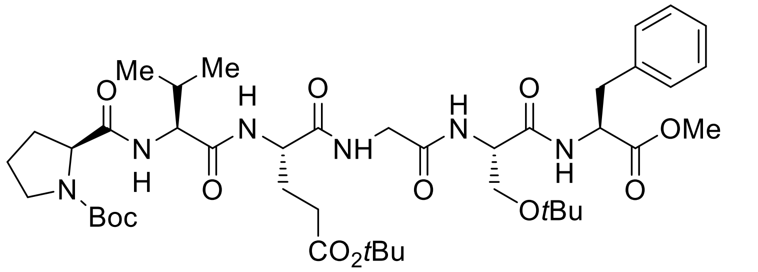

Prepared according to the general procedure **E** using 18 SPPS syringes. After the methylation step, the crude mixture was purified by flash chromatography on a SNAP cartridge KP-SIL 50 g column (automatic Biotage system) using a gradient from pentane/ethyl acetate 75:25 to ethyl acetate as eluent to afford Boc-Pro-Val-Glu-Gly-Ser-Phe-OMe **61** (0.51 g, 0.59 mmol, 41%) as a white solid.

Rf (ethyl acetate): 0.41. **Mp:** 184.5–185.3 °C. **^{1}H NMR** (400 MHz, MeOD-d_4, 298 K, mixture of rotamers) δ 7.31 – 7.24 (m, 2H, Ar*H*), 7.23 – 7.18 (m, 3H, Ar*H*), 4.7 – 4.67 (m, 1H, NHCH_{Phe}C(O)), 4.51 – 4.42 (m, 1H, NHCH_{Ser}C(O)), 4.35 (dd, *J* = 8.7, 5.4 Hz, 1H, NHCH_{Pro}C(O)), 4.31 – 4.25 (m, 1H, NHCH_{Glu}C(O)), 4.15 (app. s, 0.54H, NHCH_{Val}C(O)), 4.15 (app. s, 0.46H, NHCH_{Val}C(O)), 3.96 – 3.81 (m, 2H, NHCH_{2Gly}C(O)), 3.68 (s, 3H, OCH_3), 3.62 – 3.45 (m, 3H, NC*H*HCH_2CH_2CHC(O) + CHCH_2O*t*-Bu), 3.43 – 3.37 (m, 1H, NCH*H*CH_2CH_2CHC(O)), 3.14 (dd, J = 13.8, 6.0 Hz, 1H, CHC*H*H_{Phe}Ph), 3.05 (dd, J = 13.8, 7.5 Hz, 1H, CHCHH_{Phe}Ph), 2.41 – 2.29 (m, 2H, CHCH_2CH_{2Glu}C(O)), 2.26 – 2.03 (m, 2H, C*H*$(CH_3)_{2Val}$ + NCH_2C*H*HCH_2CHC(O)), 2.03 – 1.78 (m, 5H, NCH_2CH_2CH_2CHC(O) + NCH_2C*H*HCH_2CHC(O) + CHCH_{2Glu}CH_2C(O)), 1.47 – 1.42 (m, 18H, CH_{3OtBu}), 1.15 (s, 9H, CH_{3OtBu}), 0.98 (d, J = 6.8 Hz, 6H, $(CH_3)_{2Val}$). **^{13}C NMR** (101 MHz, MeOD-d_4, 298 K, complex mixture of rotamers, signals are not fully resolved) δ 175.7 (Cq), 174.1 (Cq), 173.9 (Cq), 173.8 (Cq), 173.7 (Cq), 173.0 (Cq), 172.0 (Cq), 171.1 (Cq), 156.7 (Cq), 156.0 (Cq), 137.9 (Cq), 130.4 (CH), 129.6 (CH), 127.9 (CH), 81.7 (Cq), 81.4 (Cq), 74.9 (Cq), 62.6 (CH_2), 61.4 (CH), 60.7 (CH), 60.4 (CH), 55.2 (CH), 54.8 (CH), 54.2 (CH), 52.7 (CH_3), 47.9 (CH_2), 43.6 (CH_2), 38.5 (CH_2), 32.6 (CH_2), 32.0 (CH_2), 31.7 (CH), 30.8 (CH_2), 28.8 (CH_3), 28.4 (CH_2), 28.2 (CH_2), 28.0 (CH_3), 27.7 (CH_3), 25.5 (CH_2), 24.6 (CH_2), 19.9 (CH_3), 19.8 (CH_3), 19.2 (CH_3), 18.8 (CH_3). **IR** (ν_{max}, cm^{-1}) IR (cm-1): 3667 (w), 2974 (s), 2905 (s), 1630 (w), 1398 (m), 1249 (m), 1229 (w), 1081 (s), 1063 (s), 747 (w), 696 (w). **HRMS (ESI/QTOF) m/z:** $[M + H]^+$ Calcd for $C_{43}H_{69}N_6O_{12}^+$ 861.4968; Found 861.4977. **LRMS** (ESI) m/z: $[M + H]^+$ Calcd for $C_{43}H_{69}N_6O_{12}^+$ 861.50; Found 861.6.

2.3 Calibration curves for peptides 59, 60 and 61

Starting materials **59**, **60** and **61** were calibrated using RP-HPLC-UV in order to estimate the yields of the subsequent azidation reaction as the azide group is not expected to significantly change the absorbance of the peptides. Samples at different concentrations were prepared and analyzed. Each analysis was performed 3 times for more accuracy.

Calibration of Boc-Pro-Val-Leu-Phe-Gly-OMe **59**

The following linear regression was obtained: y = 1233.3x + 208.33, and R = 0.9929, where axis X is the concentration in mmol/L of **59** and Y the absorbance area of the peak at 210 nm.

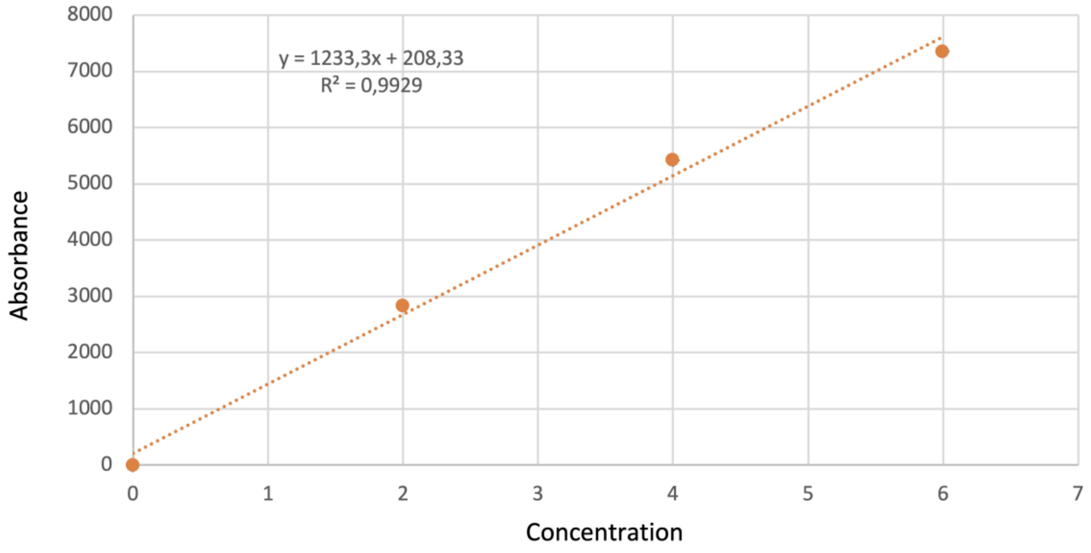

Figure S1. Calibration curve of Boc-Pro-Val-Leu-Phe-Gly-OMe **59**.

Calibration of Boc-Pro-Val-Pro-Val-Pro-Val-OMe **60**

The following linear regression was obtained: y = 1503x + 189.43, and R = 0.9961, where axis X is the concentration mmol/L of **60** and Y the absorbance area of the peak at 210 nm.

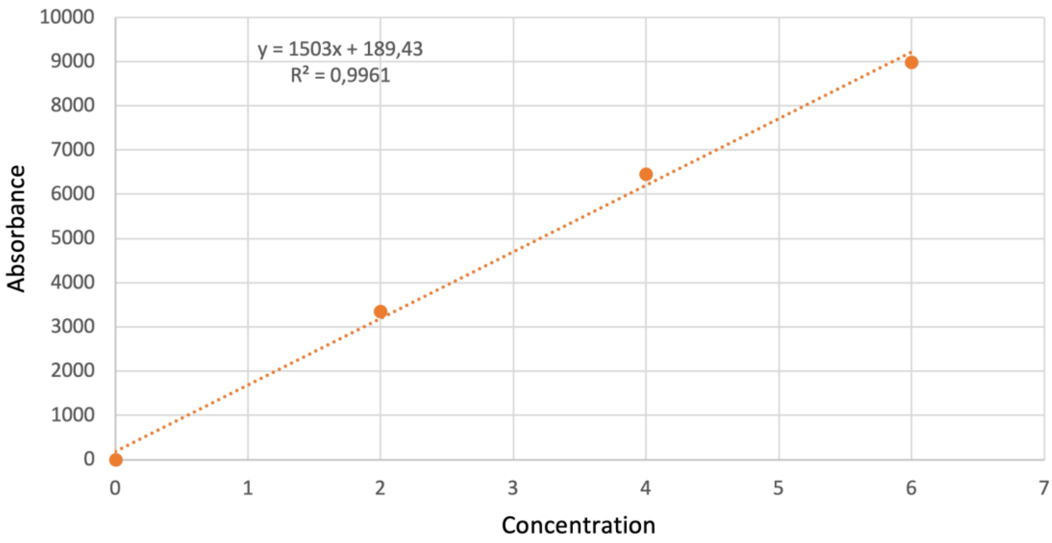

Figure S2. Calibration curve of Boc-Pro-Val-Pro-Val-Pro-Val-OMe **60**.

<u>Calibration of Boc-Pro-Val-Glu-Gly-Ser-Phe-OMe **61**</u>

The following linear regression was obtained: y = 1319.1x + 76.097, and R = 0.9992, where axis X is the concentration in mmol/L of **61** and Y the absorbance area of the peak at 210 nm.

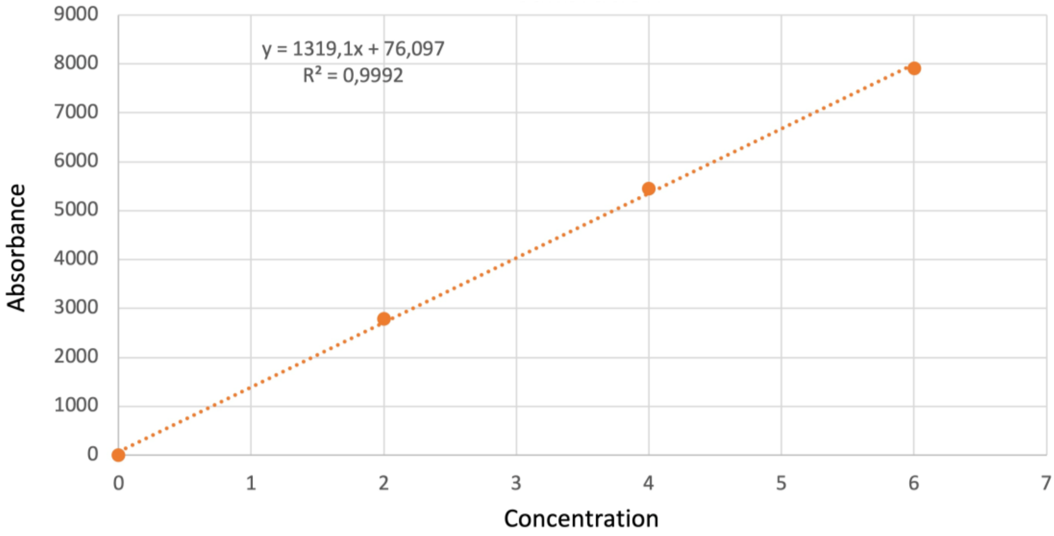

Figure S3. Calibration curve of Boc-Pro-Val-Glu-Gly-Ser-Phe-OMe **61**.

2.3 Procedures for the synthesis of ABX (1) and ABZ (2)

Azidobenziodoxolone (ABX, 1) synthesis

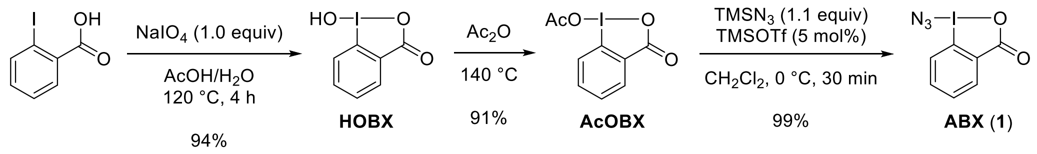

Caution: For safety reasons, the reaction was carried out behind an anti-blast shield!
Following a reported procedure,[18] 2-iodobenzoic acid (10 g, 40 mmol, 1.0 equiv) and sodium periodate (8.6 g, 40 mmol, 1.0 equiv) were suspended in aq. AcOH (30% v/v, 81 mL). The mixture was stirred at reflux (120 °C) for 4 hours. Then, ice-cold water (150 mL) was added under stirring and the mixture was allowed to cool down to room temperature, while protecting it from light with aluminium foil. Finally, the mixture was filtered and the solid was washed twice with ice-cold water (30 mL) and twice with cold acetone (30 mL). Hydroxybenziodoxolone (HOBX) (10 g, 38 mmol, 94%) was obtained as a white solid. **^{1}H NMR** (400 MHz, DMSO-*d$_6$*, 298 K) δ 8.02 (dd, *J* = 7.7, 1.4 Hz, 1 H, Ar*H*), 7.97 (m, 1 H, Ar*H*), 7.85 (dd, *J* = 8.2, 0.7 Hz, 1 H, Ar*H*), 7.71 (td, *J* = 7.6, 1.2 Hz, 1 H, Ar*H*) ppm. The signals are in accordance with the data reported in the literature.[18]

Caution: For safety reasons, the reaction was carried out behind an anti-blast shield!
Following a reported procedure,[18] hydroxybenziodoxolone (HOBX) (5.0 g, 19 mmol, 1.0 equiv) was suspended in acetic anhydride (17 mL). The suspension was stirred at reflux (140 °C) until its full solubilization. Heating was then stopped and the solution was allowed to cool down to room temperature over a period of 1.5 hours, resulting in the precipitation of a crystalline solid. Crystallization was continued at -20 °C overnight. The solid was collected by filtration and washed with several portions of pentane. Acetatebenziodoxolone (AcOBX) (5.3 g, 17 mmol, 91%) was obtained as a white crystalline solid. **^{1}H NMR** (400 MHz, $CDCl_3$, 298 K) δ = 8.25 (dd, *J* = 7.6, 1.4 Hz, 1 H, Ar*H*), 8.00 (dd, *J* = 8.3, 0.5 Hz, 1 H, Ar*H*), 7.92 (dt, *J* = 7.0, 1.7 Hz, 1 H, Ar*H*), 7.71 (td, *J* = 7.6, 0.9 Hz, 1 H, Ar*H*), 2.25 (s, 3 H, CO*CH$_3$*) ppm. The signals are in accordance with the data reported in the literature.[18]

Caution: For safety reasons, the reaction and workup were carried out behind an anti-blast shield with explosion-proof gloves!
Following a reported procedure,[18] acetatebenziodoxolone (AcOBX) (0.31 g, 1.0 mmol, 1.0 equiv) was dissolved in dry dichloromethane (2.0 mL). To the solution cooled down to 0 °C using an ice bath, azidotrimethylsilane (0.20 mL, 1.5 mmol, 1.5 equiv) was added dropwise followed by one drop of trimethylsilyl trifluoromethanesulfonate (ca. 0.9 µL, 5.0 µmol, 5 mol%) and the resulting mixture was stirred for 30 minutes at 0 °C under a nitrogen atmosphere. Pentane (12 mL) was added and the suspension was vigorously stirred for 10

[18] S. Alazet, J. Preindl, R. Simonet-Davin, S. Nicolai, A. Nanchen, T. Meyer, J. Waser *J. Org. Chem.* **2018**, *83*, 12334–12356.

minutes. The solid was then filtered, washed with pentane and dried 15 minutes on the frit under air. Azidobenziodoxolone (ABX, **1**) (0.30 g, 0.99 mmol, 99%) was obtained as a pale-yellow solid. **^{1}H NMR** (400 MHz, $CDCl_3$, 298 K) 8.19 (dd, *J* = 7.5, 1.4 Hz, 1 H, Ar*H*), 7.95 (dd, *J* = 8.4, 1.3 Hz, 1 H, Ar*H*), 7.91 (ddd, *J* = 8.4, 7.0, 1.4 Hz, 1 H, Ar*H*), 7.70 (ddd, *J* = 7.8, 6.8, 1.2 Hz, 1 H, Ar*H*) ppm. The signals are in accordance with the data reported in the literature.[18]

Azidobenziodazolone-*N*-tosyl (ABZ, 2) synthesis

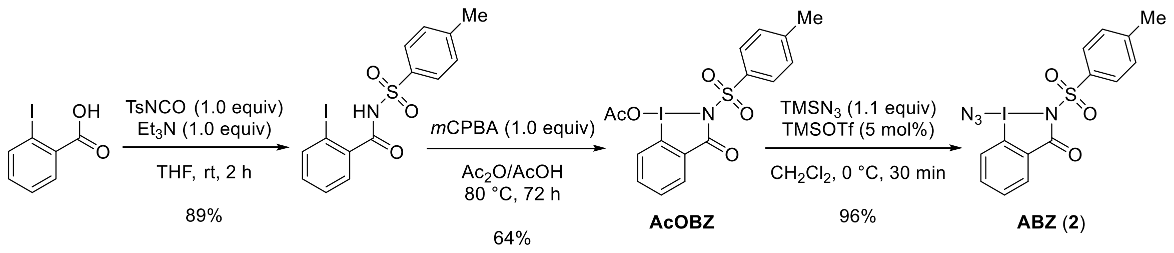

Following a reported procedure,[18] *p*-tosyl-isocyanate (6.58 mL, 40.3 mmol, 1.00 equiv) was added to a solution of 2-iodobenzoic acid (10.0 g, 40.3 mmol, 1.00 equiv) in tetrahydrofuran (115 mL). The resulting colorless mixture was stirred for 10 minutes. Then, triethylamine (5.60 mL, 40.3 mmol, 1.00 equiv) was added dropwise and the stirring was continued for 2 hours. The solution was diluted with EtOAc and washed with 1 M hydrochloride solution and brine, dried over magnesium sulfate and concentrated under reduced pressure. The residue was purified by column chromatography on a SNAP cartridge KP-SIL 120 g column (automatic Biotage system) using dichloromethane as eluent to afford 2-iodo-*N*-tosylbenzamide (14.3 g, 35.7 mmol, 89%) as a colorless oil. **Rf** (dichloromethane): 0.5. **^{1}H NMR** (400 MHz, $CDCl_3$, 298 K) δ 8.78 (s, 1 H, N*H*), 8.01 (d, *J* = 8.4 Hz, 2 H, Ar*H*), 7.80 (dd, *J* = 8.0, 1.0 Hz, 1 H, Ar*H*), 7.43 – 7.31 (m, 4 H, Ar*H*), 7.10 (ddd, *J* = 8.0, 7.2, 2.0 Hz, 1 H, Ar*H*), 2.44 (s, 3 H, C*H₃*) ppm. The signals are in accordance with the data reported in the literature.[18]

Caution: For safety reasons, the reaction was carried out behind an anti-blast shield!
Following a reported procedure,[18] *m*-chloroperoxybenzoic acid (77% purity) (8.00 g, 35.7 mmol, 1.0 equiv) was added to a solution of 2-iodo-*N*-tosylbenzamide (14.3 g, 35.7 mmol, 1.0 equiv) followed by Ac_2O (143 mL) and AcOH (143 mL) and the resulting mixture was heated for 72 hours at 80 °C. The mixture was cooled to room temperature and diethyl ether was added and the reaction flask was cooled down to - 18 °C for overnight crystallization. The precipitate formed was collected by filtration and washed with diethyl ether. Acetatebenziodazolone-*N*-tosyl (AcOBZ) (10.5 g, 22.9 mmol, 64%) was obtained as a white solid. **^{1}H NMR** (400 MHz, DMSO-d_6, 298 K) 8.02 – 7.95 (m, 2 H, Ar*H*), 7.95 – 7.89 (m, 2 H, Ar*H*), 7.86 (dd, *J* = 8.8, 0.9 Hz, 1 H, Ar*H*), 7.80 – 7.71 (m, 1 H, Ar*H*), 7.44 (d, *J* = 8.1 Hz, 2 H, Ar*H*), 2.38 (s, 3 H, CH_3), 2.26 (s, 3 H, CH_{3Ac}) ppm. The signals are in accordance with the data reported in the literature.[18]

Caution: For safety reasons, the reaction and workup were carried out behind an anti-blast shield with explosion-proof gloves!

Following a reported procedure,[18] acetatebenziodazolone-*N*-tosyl (AcOBZ) (3.0 g, 6.5 mmol, 1.0 equiv) was dissolved in dry dichloromethane (13 mL). The reaction mixture was cooled down to 0 °C using an ice bath and azidotrimethylsilane (1.4 mL, 9.8 mmol, 1.5 equiv) was added dropwise followed by trimethylsilyl trifluoromethanesulfonate (5.9 µL, 33 µmol, 5 mol%) and the resulting mixture was stirred for 30 minutes at 0 °C. Pentane was added and the suspension was stirred vigorously for 10 more minutes. The solid was then filtered, washed with pentane and dried 15 minutes on the frit under air. Azidobenziodazolone-*N*-tosyl (ABZ(Ts)) (2.77 g, 6.26 mmol, 96%) was obtained as a pale-yellow solid. **^{1}H NMR** (400 MHz, DMSO-d_6, 298 K) 8.17 (dd, J = 8.3, 0.9 Hz, 1 H, Ar*H*), 8.03 – 7.93 (m, 2 H, Ar*H*), 7.93 – 7.87 (m, 2 H, Ar*H*), 7.75 (td, J = 7.4, 0.9 Hz, 1 H, Ar*H*), 7.46 – 7.37 (m, 2 H, Ar*H*), 2.38 (s, 3 H, CH_3) ppm. The signals are in accordance with the data reported in the literature.[18]

3. Optimization of the C-H azidation reaction

The reaction was optimized using Cbz-Pro-OMe **3** as substrate (expect for the protecting group screening) on a 0.1 mmol scale.

General method for the optimization of the reaction

An oven dried 5 mL microwave vial equipped with a magnetic stirring bar was charged with Cbz-Pro-OMe **3** (26 mg, 0.10 mmol, 1.0 equiv) and the chosen iodoarene or cyclic hypervalent iodine reagent. The flask was flushed with nitrogen during few minutes after which the chosen solvent was added. When applicable, the chosen azide source followed by the chosen oxidant were then added and the flask was sealed and flushed again with nitrogen during few minutes. The reaction mixture was vigorously stirred at the chosen temperature for the chosen time. After this, when applicable, the reaction was cooled down to room temperature and the volatiles were evaporated under reduced pressure. Mesitylene (20 µL, 0.14 mmol, 1.4 equiv) was added and a ^{1}H NMR was taken.

Table S1. Temperatures screening.

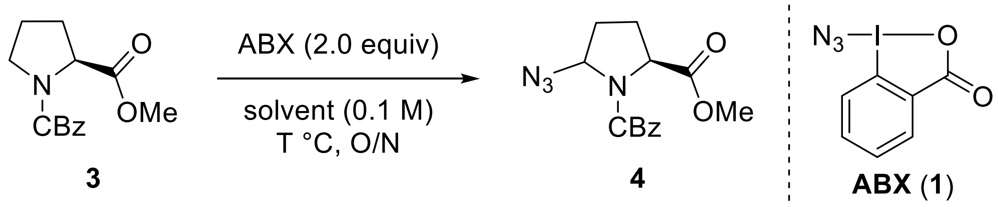

Entry	Solvent (0.1 M)	Temperature (°C)	Yield of **4**[a]	Remaining **3**[a]
1	DCM	rt	<5%	>95%
2[b]	DCM	rt	41%	28%
3	DCM	40 °C	45%	51%
4	DCE	60 °C	50%	50%
5	DCE	80 °C	40%	36%

1:1 mixture of diastereoisomers. O/N: overnight. [a] Determined by ^{1}H NMR using mesitylene as internal standard. [b] The reaction mixture was irradiated using Blue LEDS.

Table S2. Iodine and azide sources screening.

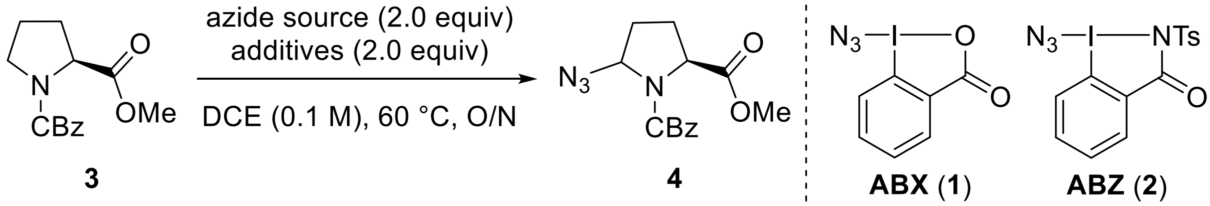

Entry	Azide source	Additives	Yield of **4**[a]	Remaining **3**[a]
1	ABX (**1**)	-	50%	50%
2	ABZ (**2**)	-	<5%	>95%

3[b]	$TMSN_3$	PIDA	32%	68%
4[b]	$TMSN_3$	PIFA	11%	73%
5[b]	$TMSN_3$	Iodobenzene + *m*CPBA	<5%	>95%
6	$TMSN_3$	2-iodobenzoic acid + *m*CPBA	50%	50%
7[c]	$TMSN_3$	2-iodobenzoic acid + *m*CPBA	43%	n.d.
8[d]	$TMSN_3$	2-iodobenzoic acid + *m*CPBA	50%	n.d.
9	NaN_3	2-iodobenzoic acid + *m*CPBA	34%	n.d.
10	$TBAN_3$	2-iodobenzoic acid + *m*CPBA	0%	100%

1:1 mixture of diastereoisomers. O/N: overnight. n.d.: not determined. [a] Determined by ^{1}H NMR using mesitylene as internal standard. [b] Reaction run at room temperature. [c] Using recrystallized mCPBA (93% purity). [d] 2.1 equiv of magnesium sulfate were added to the reaction mixture.

Table S3. Solvents and concentrations screening.

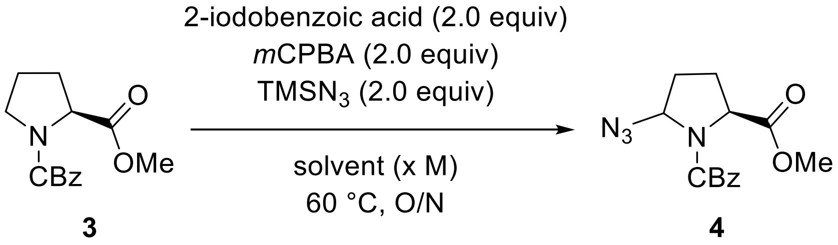

Entry	Solvent	Concentration (xM M)	Yield of **4**[a]	Remaining **3**[a]
1	DCE	0.1 M	50%	50%
2	DCE	0.2 M	50%	n.d.
3	DCE	0.05 M	50%	n.d.
4	DCM	0.1 M	50%	n.d.
5	$CHCl_3$	0.1 M	32%	69%
6	CCl_4	0.1 M	48%	n.d.
7	ACN	0.1 M	<5%	>95%
8	THF	0.1 M	0%	100%
9	DMF	0.1 M	0%	100%

1:1 mixture of diastereoisomers. O/N: overnight. n.d.: not determined. [a] Determined by ^{1}H NMR using mesitylene as internal standard. [b] Reaction run at room temperature instead of 60 °C.

Table S4. Oxidants screening.

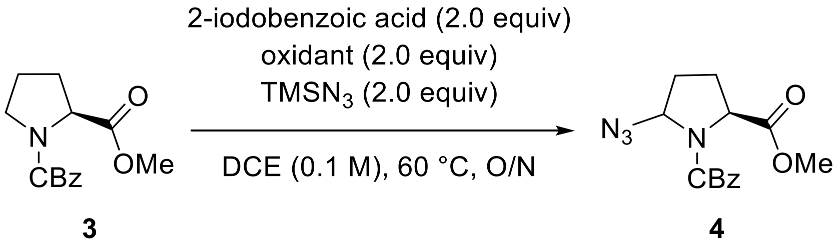

Entry	Oxidant	Yield of **4**[a]	Remaining **3**[a]
1	*m*CPBA	50%	50%
2	aq. H_2O_2	0%	100%

3	H_2O_2·urea	0%	100%
4	oxone	0%	100%
5	aq. *t*BuOOH	0%	100%

1:1 mixture of diastereoisomers. O/N: overnight. n.d.: not determined. [a] Determined by ^{1}H NMR using mesitylene as internal standard.

Table S5. Iodobenzoic acids screening.

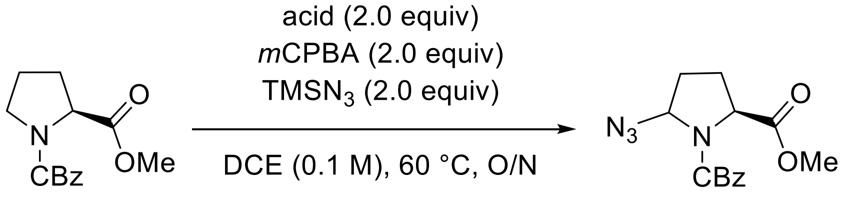

3 **4**

Entry	Acid	Yield of **4**[a]	Remaining **3**[a]
1	2-iodobenzoic acid	50%	50%
2	2-iodo-4,5-dimethoxybenzoic acid	44%	40%
3	5-fluoro-2-iodobenzoic acid	46%	42%
4	5-trifluoro-2-iodobenzoic acid	50%	n.d.

1:1 mixture of diastereoisomers. O/N: overnight. n.d.: not determined. [a] Determined by ^{1}H NMR using mesitylene as internal standard.

Table S6. Reagents ratio study and control reactions.

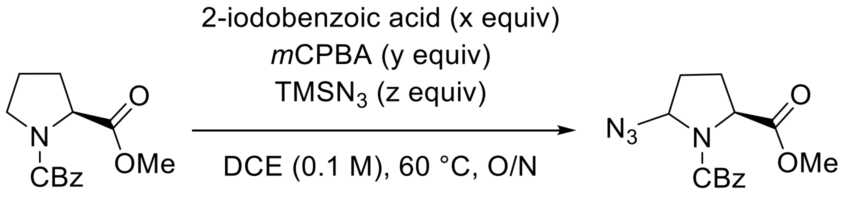

3 **4**

Entry	x equiv	y equiv	z equiv	Yield of **4**[a]	Remaining **3**[a]
1	2	2	2	50%	50%
2	1.1	1.1	1.1	35%	65%
3	4	4	4	22%	<5%
4[b]	4	4	4	32%	n.d.
5	0.5	2	2	33%	n.d.
6	0.1	2	2	16%	84%
7	-	2	2	0%	>95%
8	-	-	2	0%	>95%
9[c]	2	2	2	0%	>95%

1:1 mixture of diastereoisomers. O/N: overnight. n.d.: not determined. [a] Determined by ^{1}H NMR using mesitylene as internal standard. [b] Half of the equivalents were added after a night of reaction. [c] 2-iodobenzoic acid was replaced by benzoic acid.

Table S7. Time range of the reaction.

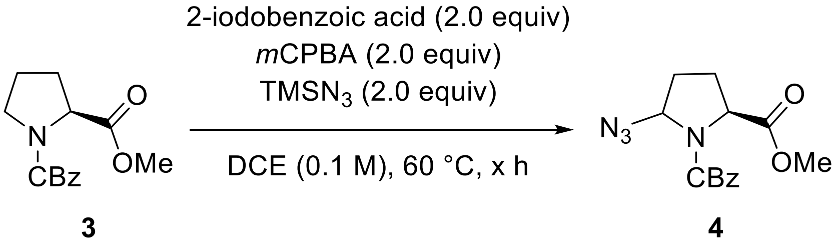

Entry	Time	Yield of **4**[a]	Remaining **3**[a]
1	5 hours	30%	70%
2	14 hours	50%	50%
3[b]	24 hours	50%	n.d.
4	48 hours	50%	n.d.

1:1 mixture of diastereoisomers. O/N: overnight. n.d.: not determined. [a] Determined by ^{1}H NMR using mesitylene as internal standard. [b] The reaction was performed on 4.0 mmol scale.

For the protecting groups screening, the same procedure was used except that Cbz-Pro-OMe **3** was replaced by the chosen proline substrate.

Table S8. Protecting groups screening.

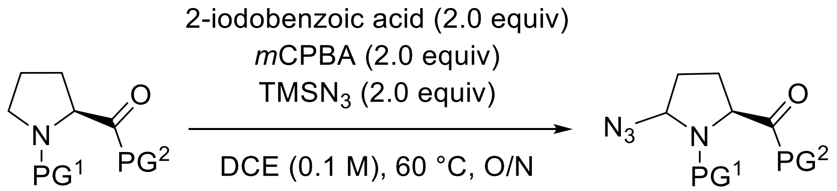

Entry	PG^1	PG^2	Yield of DP[a]	Remaining SM[a]
1[b]	CBz	Me	50% (36%)	50%
2[b]	CBz	Bn	50% (40%)	50%
3[b]	Boc	Bn	70% (68%)	traces
4	COOEt	Me	50%	n.d.
5	COO*n*Bu	Me	50%	n.d.
6	Troc	Me	0%	0%
7	Ac	Me	0%	0%
8	Piv	Me	0%	35%
9	Ts	Me	0%	56%
10	*p*-methoxybenzoyl	Me	0%	57%
11	*N,N*-dimethylcarboxamide	Me	0%	18%
12	*N,N*-diphenylcarboxamide	Bn	messy[c]	traces

1:1 mixture of diastereoisomers. O/N: overnight. n.d.: not determined. Isolated yields are given in parentheses. [a] Determined by ^{1}H NMR using mesitylene as internal standard. [b] Reactions run on a 0.4 mmol scale. [c] Multi-azidated compounds observed. PG = protecting group.

4. Scope of the azidation reaction

4.1 General procedures

General procedure F for the azidation reaction done on 0.4 mmol scale

An oven-dried 20 mL microwave vial equipped with a magnetic stirring bar was charged with the chosen substrate (0.40 mmol, 1.0 equiv) and 2-iodobenzoic acid (0.20 g, 0.80 mmol, 2.0 equiv). The flask was flushed with nitrogen during few minutes after which 1,2-dichloroethane (4.0 mL, 0.1 M) was added followed by trimethylsilyl azide (0. 11 mL, 0.80 mmol, 2.0 equiv). Solid *m*-chloroperoxybenzoic acid (77% purity) (0.18 g, 0.80 mmol, 2.0 equiv) was finally added in one portion and the flask was sealed and flushed again with nitrogen during few minutes. The heterogeneous mixture was vigorously stirred at 60 °C for 24 hours. After this time, the reaction was cooled down to room temperature, triethylamine (0.28 mL, 2.0 mmol, 5.0 equiv) was added and the mixture was stirred a few minutes, filtered over a pad of silica using ethyl acetate to rinse the silica (≈200 mL) and concentrated under reduced pressure. The crude residue was then purified by column chromatography on silica gel.

<u>NB:</u> To ensure no net loss of azidated product on the silica during the triethylamine/filtration work-up, the ^{1}H NMR yields were analyzed before and after the work-up for each compound. All the ^{1}H NMR yields were matching.

General procedure G for the azidation of free acid substrates

An oven-dried 20 mL microwave vial equipped with a magnetic stirring bar was charged with the chosen substrate (0.40 mmol, 1.0 equiv) and 2-iodobenzoic acid (0.20 g, 0.80 mmol, 2.0 equiv). The flask was flushed with nitrogen during few minutes after which 1,2-dichloroethane (4.0 mL, 0.1 M) was added followed by trimethylsilyl azide (0. 11 mL, 0.80 mmol, 2.0 equiv). Solid *m*-chloroperoxybenzoic acid (77% purity) (0.18 g, 0.80 mmol, 2.0 equiv) was finally added in one portion and the flask was sealed and flushed again with nitrogen during few minutes. The heterogeneous mixture was vigorously stirred at 60 °C for 24 hours. After this time, the reaction was cooled down to room temperature and the volatiles were evaporated under reduced pressure. Mesitylene (20 μL, 0.14 mmol, 0.36 equiv) was added and a ^{1}H NMR was taken.

General procedure H for the azidation of pentamers and hexamers done on 0.1 mmol scale

An oven dried 5 mL microwave vial equipped with a magnetic stirring bar was charged with the chosen substrate (0.1 mmol, 1.0 equiv) and 2-iodobenzoic acid (51 mg, 0.20 mmol, 2.0 equiv). The flask was flushed with nitrogen during few minutes after which 1,2-

dichloroethane (1.0 mL, 0.1 M) was added followed by trimethylsilyl azide (28 μL, 0.20 mmol, 2.0 equiv). Solid *m*-chloroperoxybenzoic acid (77% purity) (45 mg, 0.20 mmol, 2.0 equiv) was finally added in one portion, the flask was sealed and flushed again with nitrogen during few minutes. The heterogeneous mixture was vigorously stirred at 60 °C for 24 hours. After this time, the reaction was cooled down to room temperature, triethylamine (70 μL, 0.50 mmol, 5.0 equiv) was added and the mixture was stirred few minutes, filtered over a pad of silica using ethyl acetate to rinse the silica (≈150 mL) and concentrated under reduced pressure. The crude residue was diluted with dichloromethane (10 mL) and 1.0 μL of this solution was injected in an analytical HPLC-MS for analysis.

NB: To ensure no net loss of azidated product on the silica during the triethylamine/filtration work-up, the ^{1}H NMR yields were analyzed before and after the work-up for each compound. All the ^{1}H NMR yields were matching.

4.2 Characterization data

1-Benzyl 2-methyl (2*S*)-5-azidopyrrolidine-1,2-dicarboxylate (4)

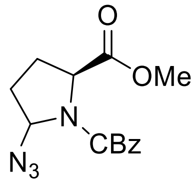

Synthesized from 1-benzyl 2-methyl (*S*)-pyrrolidine-1,2-dicarboxylate **3** (105 mg, 0.400 mmol, 1.00 equiv) following general procedure **F**. 1-benzyl 2-methyl (2*S*)-5-azidopyrrolidine-1,2-dicarboxylate **4** (43.9 mg, 0.144 mmol, 36%) (mixture of diastereoisomers, 1:1 dr determined by integration of the ^{1}H NMR peaks at 5.75 and 5.62 ppm) was obtained as a yellowish oil after purification by column chromatography on silica using a gradient from pentane to pentane/ethyl acetate 8:2 as eluent. [**4** was generated in a 50% ^{1}H NMR yield using mesitylene as internal standard].

Rf (pentane/ethyl acetate 8:2): 0.42. **^{1}H NMR** (400 MHz, $CDCl_3$, 298 K, mixture of two diastereoisomers) δ 7.44 – 7.27 (m, 5H, Ar*H*), 5.75 (d, *J* = 6.1 Hz, 0.5H, C*H*N_3), 5.62 (d, *J* = 5.9 Hz, 0.5H, C*H*N_3), 5.29 – 5.00 (m, 2H, CO_2C*H*$_2$Ph), 4.44 (ddd, *J* = 15.8, 9.3, 1.0 Hz, 1H, NC*H*C(O)), 3.74 (s, 1.5H, OC*H*$_3$), 3.55 (s, 1.5H, OC*H*$_3$), 2.47 – 2.27 (m, 1H, NCHCH_2C*H*HCHC(O)), 2.25 – 2.06 (m, 1H, NCHC*H*HCH_2CHC(O)), 2.02 – 1.96 (m, 1H, NCHCH_2CH*H*CH(O)), 1.91 – 1.79 (m, 1H, NCHCH*H*CH_2CHC(O)). **^{13}C NMR** (101 MHz, $CDCl_3$, 298 K, mixture of diastereoisomers, signals not fully resolved) δ 172.4 (Cq), 172.3 (Cq), 154.7 (Cq), 154.1 (Cq), 136.0 (Cq), 135.7 (Cq), 128.69 (CH), 128.67 (CH), 128.6 (CH), 128.5 (CH), 128.4 (CH), 128.1 (CH), 75.6 (CH), 74.8 (CH), 68.3 (CH_2), 67.8 (CH_2), 59.3 (CH), 59.1 (CH), 52.6 (CH_3), 52.4 (CH_3), 31.9 (CH_2), 30.8 (CH_2), 28.4 (CH_2), 27.3 (CH_2). **IR** (ν_{max}, cm^{-1}) 2957 (m), 2111 (s), 1746 (s), 1714 (s), 1404 (s), 1352 (s), 1200 (s), 1121 (s), 1067 (m), 1002 (m), 911 (s), 733 (s), 699 (s). **HRMS** (ESI/QTOF) m/z: $[M + Na]^+$ Calcd for $C_{14}H_{16}N_4NaO_4$ 327.1069; Found 327.1075.

(2S)-5-Azido-1-((benzyloxy)carbonyl)pyrrolidine-2-carboxylic acid (6)

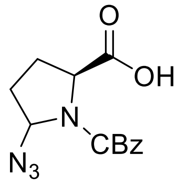

Synthesized from ((benzyloxy)carbonyl)-*L*-proline **5** (0.10 g, 0.40 mmol, 1.0 equiv) following general procedure **G**. (2S)-5-azido-1-((benzyloxy)carbonyl)pyrrolidine-2-carboxylic acid **6** was observed in a 35% ^{1}H NMR yield determined using the peaks corresponding to the C*H*N_3. [26% of remaining ((benzyloxy)carbonyl)-*L*-proline **5** were observed at the end of the reaction]. **^{1}H NMR** (400 MHz, $CDCl_3$, 298 K, complex mixture of diastereomers and rotamers) δ 5.77 (dd, *J* = 8.2, 6.3 Hz, 0.52H, C*H*N_3), 5.63 (app. t, *J* = 5.4 Hz, 0.48H, C*H*N_3). Only characteristic peaks are listed as the crude ^{1}H NMR was too complex to give the complete ^{1}H NMR listing.

Dibenzyl (2*S*)-5-azidopyrrolidine-1,2-dicarboxylate (7)

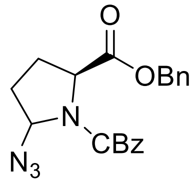

Synthesized from dibenzyl (*S*)-pyrrolidine-1,2-dicarboxylate **40** (138 mg, 0.400 mmol, 1.00 equiv) following general procedure **F**. Dibenzyl (2*S*)-5-azidopyrrolidine-1,2-dicarboxylate **7** (61.0 mg, 0.160 mmol, 40%) (mixture of diastereoisomers, 1:1 dr determined by integration of the ^{1}H NMR peaks at 5.75 and 5.62 ppm) was obtained as a yellow oil after purification by column chromatography on silica using a gradient from pentane to pentane/ethyl acetate 9:1 as eluent. [**7** was generated in a 50% ^{1}H NMR yield using mesitylene as internal standard].

Rf (pentane/ethyl acetate 9:1): 0.32. **^{1}H NMR** (400 MHz, $CDCl_3$, 298 K, mixture of two diastereoisomers) δ 7.44 – 7.17 (m, 10H, Ar*H*), 5.75 (d, *J* = 6.1 Hz, 0.5H, C$H_{diamaj}N_3$), 5.62 (d, *J* = 5.9 Hz, 0.5H, C$H_{diamin}N_3$), 5.29 – 4.92 (m, 4H, NCO_2CH_2Ph + CO_2CH_2Ph), 4.52 (d, *J* = 8.3 Hz, 0.5H, NCH_{diamin}C(O)), 4.46 (dd, *J* = 8.3 Hz, 0.5H, NCH_{diamaj}C(O)), 2.47 – 2.27 (m, 1H, $NCHCH_2C$*H*HCHC(O)), 2.22 – 2.05 (m, 1H, NCHC*H*HCH_2CHC(O)), 2.04 – 1.93 (m, 1H, $NCHCH_2$CH*H*CH(O)), 1.89 – 1.81 (m, 1H, NCHCH*H*CH_2CHC(O)). **^{13}C NMR** (101 MHz, $CDCl_3$, 298 K, mixture of diastereoisomers, signals not fully resolved) δ 171.8 (Cq_{diamaj}), 171.6 (Cq_{diamin}), 154.7 (Cq_{diamaj}), 154.2 (Cq_{diamin}), 135.9 (Cq), 135.7 (Cq), 135.5 (Cq), 135.3 (Cq), 128.8 (CH), 128.7 (CH), 128.6 (CH), 128.5 (CH), 128.4 (CH), 128.3 (CH), 128.1 (CH), 75.6 (CH_{diamaj}), 74.8 (CH_{diamin}), 68.3 (CH_2), 67.8 (CH_2), 67.3 (CH_2), 67.2 (CH_2), 59.5 (CH_{diamin}), 59.3 (CH_{diamaj}), 31.9 ($CH_{2diamin}$), 30.8 ($CH_{2diamaj}$), 28.4 ($CH_{2diamaj}$), 27.3 ($CH_{2diamin}$). **IR** (ν_{max}, cm^{-1}) 3065 (w), 3034 (w), 2959 (m), 2110 (s), 1745 (s), 1714 (s), 1405 (s), 1352 (s), 1190 (s), 1174 (s), 914 (m), 735 (s), 698 (s). **HRMS** (ESI/QTOF) m/z: $[M + Na]^+$ Calcd for $C_{20}H_{20}N_4NaO_4^+$ 403.1377; Found 403.1384.

1-(*tert*-Butyl) 2-methyl (2*S*)-5-azidopyrrolidine-1,2-dicarboxylate (8)

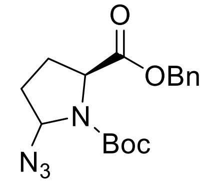

Synthesized from 2-benzyl 1-(*tert*-butyl) (*S*)-pyrrolidine-1,2-dicarboxylate **41** (122 mg, 0.400 mmol, 1.00 equiv) following general procedure **F**. 1-(*tert*-butyl) 2-methyl (2*S*)-5-azidopyrrolidine-1,2-dicarboxylate **8** (54.5 mg (rotamer maj **8a**) + 40.1 mg (rotamer min **8b**) = 94.6 mg, 0.273 mmol, 68%) (mixtures of diastereoisomers, 1:1 dr, determined by integration of the ^{1}H NMR peaks at 5.69 and 5.58 ppm (rotamer maj) and 5.70 and 5.60 ppm (rotamer min)) was obtained as yellow oils after purification by column chromatography on silica using a gradient from pentane to pentane/ethyl acetate 9:1 as eluent.

<u>**8a**: rotamer maj:</u> **Rf** (pentane/ethyl acetate 9:1)$_{rotamermaj}$: 0.64.

^{1}H NMR (400 MHz, $CDCl_3$, 298 K, mixture of two diastereoisomers) δ 7.40 – 7.29 (m, 5H, Ar*H*), 5.69 (d, *J* = 6.0 Hz, 0.5H, C$H_{diamin}N_3$), 5.58 (d, *J* = 5.9 Hz, 0.5H, C$H_{diamaj}N_3$), 5.34 – 5.02 (m, 2H, CO_2CH_2Ph), 4.45 (dd, *J* = 9.3, 0.9 Hz, 0.50H, NCH_{diamaj}C(O)), 4.36 (dd, *J* = 9.2, 0.9 Hz, 0.43H, NCH_{diamin}C(O)), 2.44 – 2.24 (m, 1H, $NCHCH_2CHHCHC(O)$), 2.18 – 2.04 (m, 1H, $NCHCHHCH_2CHC(O)$), 2.01 – 1.89 (m, 1H, $NCHCH_2CHHCH(O)$), 1.86 – 1.77 (m, 1H, $NCHCHHCH_2CHC(O)$), 1.51 (s, 5H, $CH_{3Bocdiamaj}$), 1.35 (s, 4H, $CH_{3Bocdiamin}$). **^{13}C NMR** (101 MHz, $CDCl_3$, 298 K, mixture of two diastereoisomers, signals not full resolved) δ 172.2 (Cq_{diamin}), 171.9 (Cq_{diamaj}), 153.8 (Cq_{diamin}), 153.4 (Cq_{diamaj}), 135.7 (Cq_{diamaj}), 135.5 (Cq_{diamin}), 128.8 (CH), 128.7 (CH), 128.6 (CH), 128.5 (CH), 128.3 (CH), 82.2 (Cq_{diamaj}), 81.6 (Cq_{diamin}), 75.2 (CH_{diamaj}), 75.1 (CH_{diamin}), 67.1 (CH_2), 59.5 (CH_{diamin}), 59.2 (CH_{diamaj}), 31.9 (CH_2), 30.9 (CH_2), 28.3 ($CH_{3diamaj}$), 28.1 ($CH_{3diamin}$), 27.3 (CH_2). **IR** (ν_{max}, cm^{-1}) 2979 (w), 2110 (s), 1747 (s), 1708 (s), 1379 (s), 1368 (s), 1255 (m), 1182 (s), 1156 (s). **HRMS** (nanochip-ESI/LTQ-Orbitrap) m/z: $[M + Na]^+$ Calcd for $C_{17}H_{22}N_4NaO_4^+$ 369.1533; Found 369.1541.

<u>**8b**: rotamer min:</u> **Rf** (pentane/ethyl acetate 9:1)$_{rotamermin}$: 0.37.

^{1}H NMR (400 MHz, $CDCl_3$, 298 K, mixture of two diastereoisomers) δ 7.43 – 7.28 (m, 5H, Ar*H*), 5.70 (d, *J* = 5.5 Hz, 0.5H, C$H_{diamin}N_3$), 5.60 (d, *J* = 5.6 Hz, 0.5H, C$H_{diamaj}N_3$), 5.34 – 5.10 (m, 2H, CO_2CH_2Ph), 4.37 (app t, *J* = 8.5 Hz, 0.5H, NCH_{diamaj}C(O)), 4.27 (dd, *J* = 9.4, 7.7 Hz, 0.5H, NCH_{diamaj}C(O)), 2.37 – 2.28 (m, 1H, $NCHCH_2CHHCHC(O)$), 2.20 – 2.01 (m, 1H, $NCHCHHCH_2CHC(O)$, 2.00 – 1.81 (m, 2H, NCHCH*H*CH*H*CHC(O)), 1.49 (s, 5H, $CH_{3Bocdiamaj}$), 1.34 (s, 4H, $CH_{3Bocdiamaj}$). **^{13}C NMR** (101 MHz, $CDCl_3$, 298 K, mixture of two diastereoisomers, signals not fully resolved) δ 172.1 (Cq_{diamaj}), 171.8 (Cq_{diamin}), 153.8 (Cq_{diamin}), 153.2 (Cq_{diamaj}), 135.8 (Cq_{diamaj}), 135.6 (Cq_{diamin}), 128.8 (CH), 128.7 (CH), 128.6 (CH), 128.3 (CH), 128.3 (CH), 82.2 (Cq_{diamaj}), 81.7 (Cq_{diamin}), 74.5 (CH_{diamin}), 74.3 (CH_{diamaj}), 67.1 (CH_2), 59.9 (CH_{diamin}), 59.6 (CH_{diamaj}), 32.6 ($CH_{2diamaj}$), 31.9 ($CH_{2diamin}$), 28.6 (CH_2), 28.3 ($CH_{3diamaj}$), 28.2 (CH_2), 28.1

($CH_{3diamin}$), 27.5 (CH_2). **IR** (ν_{max}, cm^{-1}) 2987 (s), 2109 (s), 1753 (s), 1705 (s), 1382 (s), 1155 (s). **HRMS** (ESI/QTOF) m/z: $[M + Na]^+$ Calcd for $C_{17}H_{22}N_4NaO_4^+$ 369.1533; Found 369.1534.

1-(*tert*-Butyl) 2-methyl (2*S*)-5-azido-2-methylpyrrolidine-1,2-dicarboxylate (9)

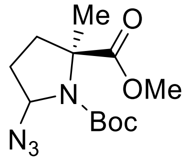

Synthesized from 1-(*tert*-butyl) 2-methyl (*S*)-2-methylpyrrolidine-1,2-dicarboxylate **42** (97.3 mg, 0.400 mmol, 1.00 equiv) following general procedure **F**. 1-(*tert*-butyl) 2-methyl (2*S*)-5-azido-2-methylpyrrolidine-1,2-dicarboxylate **9** (82.2 mg, 0.289 mmol, 72%) (complex mixture of diastereoisomers and rotamers, n.d. dr) was obtained as a yellowish oil after purification by column chromatography on silica using a gradient from pentane to pentane/ethyl acetate 9:1 as eluent.

Rf (pentane/ethyl acetate 9:1): 0.37. **^{1}H NMR** (400 MHz, $CDCl_3$, 298 K, complex mixture of diastereoisomers and rotamers) δ 5.77 (d, *J* = 5.5 Hz, 0.33H, C*H*N_3), 5.66 (d, *J* = 3.7 Hz, 0.54H, C*H*N_3), 5.53 (d, *J* = 5.4 Hz, 0.13H, C*H*N_3), 3.78 (s, 0.5H, OC*H*$_3$), 3.76 (s, 0.5H, OC*H*$_3$), 3.70 (s, 2H, OC*H*$_3$), 2.46 – 2.29 (m, 0.27H, NCHCH$_2$C*H*$_2$CC(O)), 2.21 – 1.95 (m, 2.3H, NCHC*H*$_2$CH$_2$CC(O) + NCHCH$_2$C*H*$_2$CC(O)), 1.95 – 1.79 (m, 0.65H, NCHCH$_2$C*H*$_2$CC(O)), 1.79 – 1.67 (m, 1.56H, NCHCH$_2$C*H*$_2$CC(O) + NCC*H*$_3$), 1.65 (s, 1H, NCC*H*$_3$), 1.54 (s, 0.38H, NCC*H*$_3$), 1.50 (s, 4.8H, NCC*H*$_3$ + CH_{3Boc}), 1.43 (s, 4.7H, CH_{3Boc}). **^{13}C NMR** (101 MHz, $CDCl_3$, 298 K, complex mixture of diastereoisomers and rotamers, signals not fully resolved) δ 174.5 (Cq), 174.3 (Cq), 174.1 (Cq), 153.8 (Cq), 153.4 (Cq), 153.0 (Cq), 152.4 (Cq), 82.0 (Cq), 81.9 (Cq), 81.7 (Cq), 81.6 (Cq), 76.8 (CH), 76.4 (CH), 75.0 (CH), 74.7 (CH), 66.3 (Cq), 66.2 (Cq), 65.9 (Cq), 65.8 (Cq), 52.7 (CH_3), 52.6 (CH_3), 52.5 (CH_3), 38.1 (CH_2), 37.1 (CH_2), 37.0 (CH_2), 35.9 (CH_2), 31.5 (CH_2), 30.7 (CH_2), 30.5 (CH_2), 30.2 (CH_2), 28.3 (CH_3), 28.3 (CH_3), 28.2 (CH_3), 24.9 (CH_3), 23.9 (CH_3), 22.1 (CH_3), 21.1 (CH_3). **IR** (ν_{max}, cm^{-1}) 2978 (m), 2939 (m), 2110 (s), 1743 (s), 1705 (s), 1458 (w), 1369 (s), 1250 (m), 1207 (m), 1161 (s), 1065 (m). **HRMS** (ESI/QTOF) m/z: $[M + Na]^+$ Calcd for $C_{12}H_{20}N_4NaO_4^+$ 307.1377; Found 307.1382.

(2S)-5-Azido-1-(tert-butoxycarbonyl)pyrrolidine-2-carboxylic acid (10)

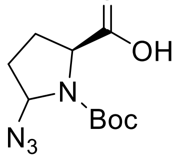

Synthesized from (tert-butoxycarbonyl)-*L*-proline (88 mg, 0.40 mmol, 1.0 equiv) following general procedure **G**. (2*S*)-5-azido-1-(tert-butoxycarbonyl)pyrrolidine-2-carboxylic acid **5** was observed in a 50% ^{1}H NMR yield determined using the peaks corresponding to the C*H*N_3. **^{1}H**

NMR (400 MHz, $CDCl_3$, 298 K, complex mixture of diastereomers and rotamers) δ 5.76 – 5.70 (m, 0.45H, C*H*N_3), 5.63 – 5.55 (m, 0.55H, C*H*N_3). Only characteristic peaks are listed as the crude ^{1}H NMR was too complex to give the complete ^{1}H NMR listing.

tert-Butyl (5*S*)-2-azido-5-((2-methoxy-2-oxoethyl)carbamoyl)pyrrolidine-1-carboxylate (12)

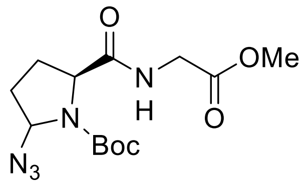

Synthesized from *tert*-butyl (*S*)-2-((2-methoxy-2-oxoethyl)carbamoyl)pyrrolidine-1-carboxylate **11** (115 mg, 0.400 mmol, 1.00 equiv) following general procedure **F**. *tert*-butyl (5*S*)-2-azido-5-((2-methoxy-2-oxoethyl)carbamoyl)pyrrolidine-1-carboxylate **12** (74.1 mg, 0.226 mmol, 57%) (mixture of diastereoisomers, n.d. dr) was obtained as yellow oil after purification by column chromatography on silica using a gradient from pentane to pentane/ethyl acetate 6:4 as eluent.

Rf (pentane/ethyl acetate 6:4): 0.33. **^{1}H NMR** (400 MHz, MeOD-*d*$_4$, 278.2 K, complex mixture of diastereoisomers and rotamers) δ 5.68 – 5.62 (m, 1H, C*H*N_3), 4.31 (dd, *J* = 8.8, 4.0 Hz, 0.45H, $NCHCH_2CH_2$C*H*C(O)), 4.23 (q, *J* = 8.6 Hz, 0.55H, $NCHCH_2CH_2$C*H*C(O)), 3.75 – 3.69 (m, 2H, $NHCH_{2Gly}$C(O)), 3.75 – 3.68 (app. m, 3H, OCH_3), 2.43 – 1.93 (m, 3H, CH_{2Pro}), 1.87 – 1.78 (m, 1H, CH_{2Pro}), 1.55 – 1.40 (m, 9H, CH_{3Boc}). **^{13}C NMR** (101 MHz, MeOD-*d*$_4$, 278.2 K, complex mixture of diastereoisomers and rotamers, signals not fully resolved) δ 175.43 (Cq), 175.35 (Cq), 175.2 (Cq), 175.0 (Cq), 174.8 (Cq), 174.7 (Cq), 171.5 (Cq), 171.5 (Cq), 171.4 (Cq), 155.7 (Cq), 155.5 (Cq), 155.1 (Cq), 155.0 (Cq), 83.2 (Cq), 83.0 (Cq), 82.7 (Cq), 82.6 (Cq), 77.0 (CH), 76.9 (CH), 76.69 (CH), 76.65 (CH), 62.4 (CH), 62.1 (CH), 61.5 (CH), 61.1 (CH), 52.61 (CH_3), 52.59 (CH_3), 52.57 (CH_3), 41.9 (CH_2), 41.8 (CH_2), 41.7 (CH_2), 41.6 (CH_2), 33.2 (CH_2), 32.8 (CH_2), 32.6 (CH_2), 31.7 (CH_2), 30.1 (CH_2), 29.8 (CH_2), 29.1 (CH_2), 28.8 (CH_2), 28.5 (CH_3), 28.3 (CH_3). **IR** (ν_{max}, cm^{-1}) 3339 (m), 2985 (m), 2947 (m), 2111 (s), 1756 (s), 1708 (s), 1676 (s), 1381 (s), 1209 (s), 1161 (s). **HRMS** (ESI/QTOF) m/z: $[M + Na]^+$ Calcd for $C_{13}H_{21}N_5NaO_5^+$ 350.1435; Found 350.1445.

tert-Butyl (5*S*)-2-azido-5-((*S*)-2-((benzyloxy)carbonyl)pyrrolidine-1-carbonyl)pyrrolidine-1-carboxylate (14)

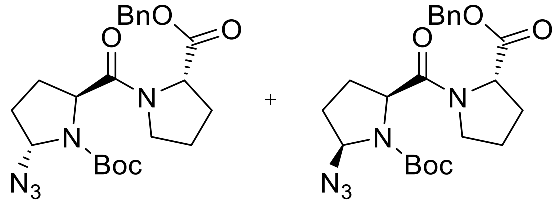

14a (dia min) **14b** (dia maj)

Synthesized from *tert*-butyl (*S*)-2-((*S*)-2-((benzyloxy)carbonyl)pyrrolidine-1-carbonyl)pyrrolidine-1-carboxylate **13** (163 mg, 0.400 mmol, 1.00 equiv) following general procedure **F**. *tert*-butyl (5*S*)-2-azido-5-((*S*)-2-((benzyloxy)carbonyl)pyrrolidine-1-carbonyl)pyrrolidine-1-carboxylate **14** (97.1 mg, 0.219 mmol, 55%) (mixture of diastereoisomers, 3.3:1 dr determined by integration of the ^{1}H NMR peaks at 5.70 and 5.59 ppm) was obtained as yellow sticky oil after purification by column chromatography on silica using a gradient from pentane to pentane/ethyl acetate 7:3 as eluent.

Rf (pentane/ethyl acetate 7:3): 0.26. **^{1}H NMR** (400 MHz, $CDCl_3$, 298 K, mixture of two diastereoisomers) δ 7.40 – 7.26 (m, 5H, Ar*H*), 5.70 (d, *J* = 5.7 Hz, 0.23H, $CH_{diamin}N_3$), 5.59 (d, *J* = 5.3 Hz, 0.77H, $CH_{diamaj}N_3$), 5.29 – 5.11 (m, 1H, OC*H*HPh), 5.11 – 4.98 (m, 1H, OCH*H*Ph), 4.76 – 4.58 (m, 1H, NC*H*C(O)$_{C\text{-terminalPro}}$), 4.55 – 4.46 (m, 1H, NC*H*C(O)$_{N\text{-terminalPro}}$), 3.85 – 3.51 (m, 2H, N$CH_{2C\text{-terminalPro}}$$CH_2CH_2$CHC(O)), 2.32 – 1.82 (m, 7.3H, NCHCH_2CH_2CHC(O)$_{N\text{-terminalPro}}$ + N$CH_2CH_2CH_2$CHC(O)$_{C\text{-terminalPro}}$), 1.76 – 1.66 (m, 0.7H, NCHCH*H*CH_2CHC(O)$_{N\text{-terminalPro}}$), 1.50 (s, 6.9H, $CH_{3Bocdiamaj}$), 1.39 (s, 2.1H, $CH_{3Bocdiamin}$). **^{13}C NMR** (101 MHz, $CDCl_3$, 298 K, mixture of two diastereoisomers, signals not fully resolved) δ 172.2 (Cq_{diamaj}), 171.9 (Cq_{diamin}), 170.4 (Cq_{diamin}), 169.8 (Cq_{diamaj}), 153.8 (Cq_{diamin}), 153.6 (Cq_{diamaj}), 135.7 (Cq_{diamaj}), 135.6 (Cq_{diamin}), 128.7 (CH), 128.7 (CH), 128.5 (CH), 128.5 (CH), 128.4 (CH), 128.3 (CH), 81.9 (Cq_{diamaj}), 81.0 (Cq_{diamin}), 75.7 (CH_{diamin}), 75.5 (CH_{diamaj}), 67.2 ($CH_{2diamin}$), 67. ($CH_{2diamaj}$), 59.03 (CH_{diamaj}), 58.98 (CH_{diamin}), 58.6 (CH_{diamin}), 58.3 (CH_{diamaj}), 46.6 ($CH_{2diamin}$), 46.6 ($CH_{2diamaj}$), 31.7 ($CH_{2diamaj}$), 30.7 ($CH_{2diamin}$), 29.0 ($CH_{2diamin}$), 28.8 ($CH_{2diamaj}$), 28.4 ($CH_{3diamaj}$), 28.2 ($CH_{3diamin}$), 27.5 ($CH_{2diamin}$), 26.6 ($CH_{2diamaj}$), 25.1 ($CH_{2diamin}$), 25.0 ($CH_{2diamaj}$). **IR** (ν_{max}, cm^{-1}) 2978 (m), 2885 (w), 2110 (s), 1743 (m), 1705 (s), 1662 (s), 1385 (s), 1165 (s). **HRMS** (ESI/QTOF) m/z: $[M + Na]^+$ Calcd for $C_{22}H_{29}N_5NaO_5^+$ 466.2061; Found 466.2061.

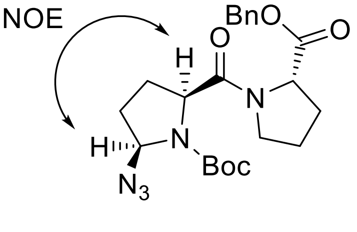

14b (dia maj)

Methyl 2-azido-2-((*tert*-butoxycarbonyl)amino)acetate (15)

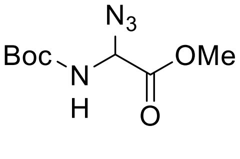

Synthesized from methyl (*tert*-butoxycarbonyl)glycinate **43** (75.7 mg, 0.400 mmol, 1.00 equiv) following general procedure **F**. Methyl 2-azido-2-((*tert*-butoxycarbonyl)amino)acetate **15** (31.5 mg, 0.137 mmol, 34%) was obtained as a yellow oil after purification by column chromatography on silica using a gradient from pentane to pentane/ethyl acetate 92:8 as eluent.

Rf (pentane/ethyl acetate 92:8): 0.24. **^{1}H NMR** (400 MHz, $CDCl_3$, 298 K) δ 5.73 (br s, 1H, N*H*), 5.57 (d, *J* = 7.9 Hz, 1H, C*H*N_3), 3.86 (s, 3H, CO_2CH_3), 1.48 (s, 9H, $CO_2C(CH_3)_3$). **^{13}C NMR** (101 MHz, $CDCl_3$) δ 167.2 (Cq), 154.4 (Cq), 81.7 (Cq), 66.7 (CH), 53.6 (CH_3), 28.3 (CH_3). **IR** ($ν_{max}$, cm^{-1}) 3348 (m), 2981 (m), 2110 (s), 1755 (s), 1713 (s), 1504 (s), 1346 (s), 1235 (s), 1211 (s), 1151 (s), 1059 (s), 1000 (s). **HRMS** (nanochip-ESI/LTQ-Orbitrap) m/z: $[M + Na]^+$ Calcd for $C_8H_{14}N_4NaO_4^+$ 253.0907; Found 253.0907.

Benzyl 2-azido-2-((*tert*-butoxycarbonyl)amino)acetate (16)

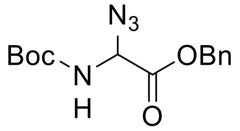

Synthesized from benzyl (*tert*-butoxycarbonyl)glycinate **F** (106 mg, 0.400 mmol, 1.00 equiv) following general procedure **44**. Benzyl 2-azido-2-((*tert*-butoxycarbonyl)amino)acetate **16** (39.8 mg, 0.130 mmol, 32%) was obtained as a yellowish solid after purification by column chromatography on silica using a gradient from pentane to pentane/ethyl acetate 94:6 as eluent.

Rf (pentane/ethyl acetate 94:6): 0.26. **Mp:** 59.0–68.0 °C. **^{1}H NMR** (400 MHz, $CDCl_3$, 298 K) δ 7.42 – 7.34 (m, 5H, Ar*H*), 5.72 (br s, 1H, N*H*), 5.59 (d, *J* = 8.0 Hz, 1H, C*H*N_3), 5.27 (dd, *J* = 17.4, 12.1 Hz, 2H, CO_2CH_2Ph), 1.47 (s, 9H, $CO_2C(CH_3)_3$). **^{13}C NMR** (101 MHz, $CDCl_3$) δ 166.6 (Cq), 154.4 (Cq), 134.5 (Cq), 129.0 (CH), 128.9 (CH), 128.7 (CH), 81.7 (Cq), 68.6 (CH_2), 66.8 (CH), 28.3 (CH_3). **IR** ($ν_{max}$, cm^{-1}) 3352 (w), 2981 (w), 2114 (s), 1720 (s), 1504 (m), 1331 (s), 1238 (s), 1161 (s), 1057 (m), 987 (m), 741 (m). **HRMS** (ESI/QTOF) m/z: $[M + Na]^+$ Calcd for $C_{14}H_{18}N_4NaO_4^+$ 329.1226; Found 329.1229.

Methyl 2-azido-2-(3,3-diphenylureido)acetate (17)

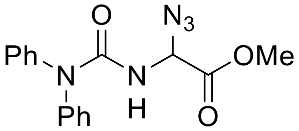

Synthesized from methyl (diphenylcarbamoyl)glycinate **45** (114 mg, 0.400 mmol, 1.00 equiv) following general procedure **F**. Methyl 2-azido-2-(3,3-diphenylureido)acetate **17** (50.1 mg, 0.154 mmol, 39%) was obtained as an orange oil after purification by column chromatography on silica using a gradient from dichloromethane to dichloromethane/ethyl acetate 9:1 as eluent.

Rf (dichloromethane/ethyl acetate 9:1): 0.71. **^{1}H NMR** (400 MHz, MeOD-*d4*, 298 K) δ 7.44 – 7.38 (m, 4H, Ar*H*), 7.31 – 7.27 (m, 6H, Ar*H*), 5.50 (s, 1H, C*H*N_3), 3.79 (s, 3H, CO_2CH_3). **^{13}C NMR** (101 MHz, MeOD-*d4*, 298 K) δ 169.0 (Cq), 157.6 (Cq), 143.5 (Cq), 130.6 (CH), 128.7 (CH), 128.1 (CH), 68.2 (CH), 53.7 (CH_3). **IR** ($ν_{max}$, cm^{-1}) 3417 (w), 3064 (w), 3040 (w), 2956 (w), 2111 (s),

1751 (m), 1676 (s), 1490 (s), 1351 (m), 1210 (s), 759 (m), 701 (s). **HRMS** (ESI/QTOF) m/z: [M + Na]$^+$ Calcd for $C_{16}H_{15}N_5NaO_3^+$ 348.1067; Found 348.1063.

Methyl (2-azido-2-((*tert*-butoxycarbonyl)amino)acetyl)-*L*-prolinate (18)

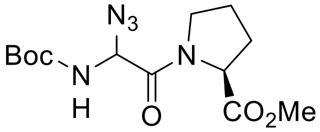

Synthesized from methyl (*tert*-butoxycarbonyl)glycyl-*L*-prolinate **46** (115 mg, 0.400 mmol, 1.00 equiv) following general procedure **F**. Methyl (2-azido-2-((*tert*-butoxycarbonyl)amino)acetyl)-*L*-prolinate **18** (41.3 mg, 0.126 mmol, 32%) (mixture of diastereoisomers, n.d. dr) was obtained as a yellow oil after purification by column chromatography on silica using a gradient from pentane to pentane/ethyl acetate 7:3 as eluent.

Rf (pentane/ethyl acetate 7:3): 0.25. **^{1}H NMR** (400 MHz, $CDCl_3$, 278.2 K, complex mixture of diastereoisomers and rotamers) δ 6.29 – 6.26 (m, 0.9H, N*H*), 6.07 (br s, 0.1H, N*H*), 5.57 (d, *J* = 7.8 Hz, 0.4H, C*H*N_3), 5.44 (d, *J* = 8.0 Hz, 0.4H, C*H*N_3), 5.31 – 5.28 (m, 0.2H, C*H*N_3), 4.70 (dd, *J* = 7.3, 3.2 Hz, 0.08H, NCH_{Pro}C(O)), 4.60 (dd, *J* = 9.4, 3.6 Hz, 0.04H, NCH_{Pro}C(O)), 4.51 (ddd, *J* = 10.7, 6.7, 4.0 Hz, 0.87H, , NCH_{Pro}C(O)), 3.93 – 3.86 (m, 0.43H, NCH_2CH_2CH_2CH_{Pro}), 3.77 – 3.64 (m, 4H, NCH_2CH_2CH_2CH_{Pro} + OCH_3), 3.61 – 3.52 (m, 0.56, NCH_2CH_2CH_2CH_{Pro}), 2.31 – 2.00 (m, 4H, NCH_2CH_2CH_2CH_{Pro}), 1.46 (s, 9H, CH_{3Boc}). **^{13}C NMR** (101 MHz, $CDCl_3$, 278.2 K, complex mixture of diastereoisomers and rotamers, signals not fully resolved) δ 172.1 (Cq), 171.8 (Cq), 171.7 (Cq), 164.2 (Cq), 163.8 (Cq), 163.8 (Cq), 154.7 (Cq), 154.6 (Cq), 154.4 (Cq), 81.3 (Cq), 81.3 (Cq), 81.3 (Cq), 65.4 (CH), 65.2 (CH), 65.1 (CH), 64.9 (CH), 59.3 (CH), 59.2 (CH), 58.9 (CH), 53.2 (CH_3), 52.8 (CH_3), 52.7 (CH_3), 47.0 (CH_2), 47.0 (CH_2), 46.9 (CH_2), 31.1 (CH_2), 29.2 (CH_2), 29.1 (CH_2), 28.4 (CH_3), 28.3 (CH_3), 28.2 (CH_3), 24.8 (CH_2), 24.7 (CH_2), 22.25 (CH_2), 21.9 (CH_2). **IR** (ν_{max}, cm^{-1}) 2971 (m), 2108 (s), 1746 (s), 1718 (s), 1660 (s), 1496 (m), 1436 (s), 1367 (m), 1237 (m), 1155 (s), 1061 (m). **HRMS** (nanochip-ESI/LTQ-Orbitrap) m/z: [M + Na]$^+$ Calcd for $C_{13}H_{21}N_5NaO_5^+$ 350.1435; Found 350.1429.

***tert*-Butyl (5*S*)-2-azido-5-(((*S*)-1-methoxy-1-oxopropan-2-yl)carbamoyl)pyrrolidine-1-carboxylate (20)**

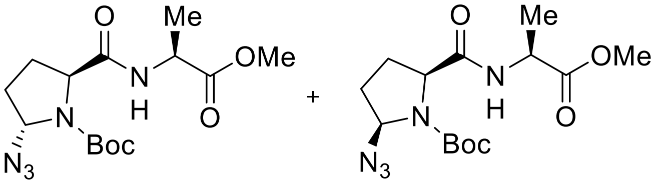

20a (dia min) **20b** (dia maj)

Synthesized from *tert*-butyl (*S*)-2-(((*S*)-1-methoxy-1-oxopropan-2-yl)carbamoyl)pyrrolidine-1-carboxylate **48** (120 mg, 0.400 mmol, 1.00 equiv) following general procedure **F**. *Tert*-butyl (5*S*)-2-azido-5-(((*S*)-1-methoxy-1-oxopropan-2-yl)carbamoyl)pyrrolidine-1-carboxylate **20** (43.8 mg (diastereoisomer min **20a**) + 48.8 mg (diastereoisomer maj **20b**) = 92.6 mg, 0.271 mmol, 68%) (1.1:1 dr) was obtained as yellow oils after purification by column chromatography on silica using a gradient from pentane to pentane/ethyl acetate 7:3 as eluent.

20a: diastereoisomer min, **Rf** (pentane/ethyl acetate 9:1)$_{diamin}$: 0.18.

^{1}**H NMR** (400 MHz, MeOD-*d*$_4$, 278.2 K, complex mixture of two rotamers) δ 5.64 (dd, *J* = 8.9, 5.8 Hz, 1H, C*H*N$_3$), 4.44 – 4.32 (m, 1H, NHC*H*$_{Ala}$C(O)), 4.31 (dd, *J* = 8.2, 6.7 Hz, 1H, NCHCH$_2$CH$_2$C*H*C(O)), 3.70 (app. d, *J* = 4.4 Hz, 3H, OC*H$_3$*), 2.42 – 2.22 (m, 1H, NCHCH$_2$C*H*HCHC(O)), 2.20 – 2.06 (m, 1H, NCHC*H*HCH$_2$CHC(O)), 2.05 – 1.97 (m, 1H, NCHCH$_2$CH*H*CHC(O)), 1.83 – 1.73 (m, 1H, NCHCH*H*CH$_2$CHC(O)), 1.51 + 1.43 (2 s, 9H, C*H$_{3Boc}$*), 1.40 (d, *J* = 7.4 Hz, 3H, C*H$_{3Ala}$*). ^{13}**C NMR** (101 MHz, MeOD-*d*$_4$, 278.2 K, complex mixture of two rotamers, signals not fully resolved) δ 174.6 (Cq), 174.5 (Cq), 174.4 (Cq), 174.2 (Cq), 155.6 (Cq), 155.0 (Cq), 83.0 (Cq), 82.5 (Cq), 76.9 (CH), 61.2 (CH), 60.8 (CH), 52.8 (CH$_3$), 52.7 (CH$_3$), 32.7 (CH$_2$), 31.8 (CH$_2$), 29.6 (CH$_2$), 28.7 (CH$_2$), 28.5 (CH$_3$), 28.4 (CH$_3$), 17.3 (CH$_3$), 17.1 (CH$_3$); one CH is under the MeOD-*d*$_4$ peak (see HSQC). **IR** (ν_{max}, cm^{-1}) 3330 (m), 2982 (m), 2111 (s), 1749 (s), 1708 (s), 1545 (m), 1383 (s), 1208 (s), 1160 (s). **HRMS** (ESI/QTOF) m/z: [M + Na]$^+$ Calcd for $C_{14}H_{23}N_5NaO_5^+$ 364.1591; Found 364.1594.

20b: diastereoisomer maj: **Rf** (pentane/ethyl acetate 7:3)$_{diamaj}$: 0.13.

^{1}**H NMR** (400 MHz, MeOD-*d*$_4$, 278.2 K, complex mixture of two rotamers) δ 5.65 (dd, *J* = 11.1, 4.9 Hz, 0.94H, C*H*N$_3$), 5.23 (d, *J* = 4.6 Hz, 0.06H, C*H*N$_3$), 4.42 (app. qd, *J* = 7.4, 2.6 Hz, 1H, NHC*H*$_{Ala}$C(O)), 4.21 (q, *J* = 8.8 Hz, 1H, NCHCH$_2$CH$_2$C*H*C(O)), 3.72 (app. d, *J* = 2.0 Hz, 3H, OC*H$_3$*), 2.35 – 2.21 (m, 1H, NCHCH$_2$C*H*HCHC(O)), 2.09 – 1.91 (m, 2H, NCHCH$_2$CH*H*CHC(O) + NCHC*H*HCH$_2$CHC(O)), 1.87 – 1.74 (m, 1H, NCHCH*H*CH$_2$CHC(O)), 1.50 (s, 4.5H, C*H$_{3Boc}$*), 1.43 (s, 4.5H, C*H$_{3Boc}$*), 1.41 (d, *J* = 7.3 Hz, 3H, C*H$_{3Ala}$*). ^{13}**C NMR** (101 MHz, MeOD-*d*$_4$, 278.2 K, complex mixture of two rotamers, signals not fully resolved) δ 174.6 (Cq), 174.4 (Cq), 174.2 (Cq), 173.9 (Cq), 155.6 (Cq), 154.9 (Cq), 83.0 (Cq), 82.9 (Cq), 82.5 (Cq), 82.5 (Cq), 76.9 (CH), 76.3 (CH), 76.3 (CH), 62.0 (CH), 61.7 (CH), 61.2 (CH), 60.8 (CH), 52.8 (CH$_3$), 52.8 (CH$_3$), 55.7 (CH$_3$), 33.3 (CH$_2$), 32.7 (CH$_2$), 32.6 (CH$_2$), 31.8 (CH$_2$), 30.8 (CH$_2$), 29.9 (CH$_2$), 29.6 (CH$_2$), 29.0 (CH$_2$), 28.7 (CH$_2$), 28.4 (CH$_3$), 28.3 (CH$_3$), 17.7 (CH$_3$), 17.43 (CH$_3$), 17.35 (CH$_3$), 17.1 (CH$_3$); one CH is under the

MeOD-d_4 peak (see HSQC). **IR** (ν_{max}, cm^{-1}) 3334 (m), 2981 (m), 2111 (s), 1746 (s), 1705 (s), 1539 (m), 1379 (s), 1208 (m), 1160 (s). **HRMS** (ESI/QTOF) m/z: [M + Na]$^+$ Calcd for $C_{14}H_{23}N_5NaO_5^+$ 364.1591; Found 364.1590.

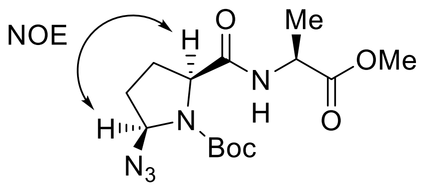

20b (dia maj)

tert-Butyl (5_S_)-2-azido-5-(((_S_)-1-methoxy-3-methyl-1-oxobutan-2-yl)carbamoyl)pyrrolidine-1-carboxylate (21)

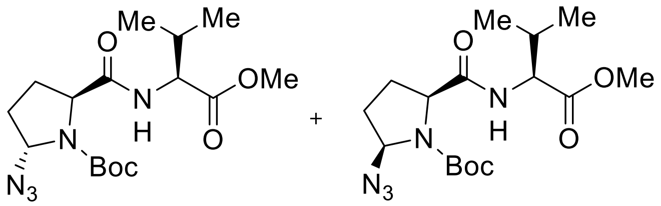

21a (dia min) **21b** (dia maj)

Synthesized from *tert*-butyl (*S*)-2-(((*S*)-1-methoxy-3-methyl-1-oxobutan-2-yl)carbamoyl)pyrrolidine-1-carboxylate **49** (131 mg, 0.400 mmol, 1.00 equiv) following general procedure **F**. *tert*-butyl (5*S*)-2-azido-5-(((*S*)-1-methoxy-3-methyl-1-oxobutan-2-yl)carbamoyl)pyrrolidine-1-carboxylate **21** (39.4 mg (diastereoisomer min **21a**) + 70.4 mg (diastereoisomer maj **21b**) = 110 mg, 0.297 mmol, 74%) (1.8:1 dr) was obtained as yellow oils after purification by column chromatography on silica using a gradient from pentane to pentane/ethyl acetate 7:3 as eluent.

<u>**21a**: diastereoisomer min: </u>**Rf** (pentane/ethyl acetate 7:3)$_{diamin}$: 0.47.

^{1}H NMR (400 MHz, MeOD-d_4, 298 K, mixture of two rotamers) δ 5.61 (dd, *J* = 8.6, 5.8 Hz, 1H, C*H*N$_3$), 4.47 – 4.38 (m, 1H, NCHCH$_2$CH$_2$C*H*C(O)), 4.30 (d, *J* = 5.8 Hz, 0.5H, NHC*H*$_{Val}$C(O)), 4.25 (d, *J* = 5.9 Hz, 0.5H, NHC*H*$_{Val}$C(O)), 3.73 – 3.69 (m, 3H, OC*H$_3$*), 2.43 – 1.90 (m, 4H, NCHCH$_2$CH*H*CHC(O) + C*H*$_{Val}$(CH$_3$)$_2$), 1.86 – 1.74 (ddd, *J* = 12.4, 6.3, 4.0 Hz, 1H, NCHCH$_2$CH*H*CHC(O)), 1.51 (s, 4H, C*H*$_{3Bocrotamermin}$), 1.43 (s, 5H, C*H*$_{3Bocrotamermaj}$), 1.01 – 0.97 (m, 6H, C*H*$_{3Val}$). **^{13}C NMR** (101 MHz, MeOD-d_4, 278.2 K, complex mixture of two rotamers, signals not fully resolved) δ 175.1 (Cq), 175.0 (Cq), 174.7 (Cq), 174.6 (Cq), 173.52 (Cq), 173.50 (Cq), 173.48 (Cq), 173.47 (Cq), 155.6 (Cq), 154.9 (Cq), 82.9 (Cq), 82.4 (Cq), 76.9 (CH), 76.9 (CH), 61.12 (CH), 61.08 (CH), 60.79 (CH), 60.75 (CH), 59.7 (CH), 59.6 (CH), 59.4 (CH), 59.3 (CH), 52.49 (CH$_3$), 52.47 (CH$_3$), 32.8 (CH$_2$), 31.80 (CH), 31.77 (CH), 31.7 (CH$_2$), 31.43 (CH), 31.40 (CH), 29.9 (CH$_2$), 28.7 (CH$_2$), 28.5 (CH$_3$), 28.4 (CH$_3$), 19.8 (CH$_3$), 19.5 (CH$_3$), 18.6 (CH$_3$), 18.4 (CH$_3$). **IR** (ν_{max}, cm^{-1}) 3348 (m), 2980 (m), 2909 (w), 2109 (s), 1743 (s), 1708 (s), 1522 (m), 1381 (s), 1157 (s). **HRMS** (ESI/QTOF) m/z: [M + Na]$^+$ Calcd for $C_{16}H_{27}N_5NaO_5^+$ 392.1915; Found 392.1912.

<u>**21b**: diastereoisomer maj:</u> **Rf** (pentane/ethyl acetate 7:3)$_{diamaj}$: 0.28.

^{1}H NMR (400 MHz, MeOD-*d*$_4$, 278.2 K, complex mixture of rotamers) δ 5.71 – 5.61 (m, 0.7H, C*H*N$_3$), 5.28 (app. br s, 0.3H, C*H*N$_3$), 4.48 – 4.21 (m, 2H, CHCH$_2$CH$_2$C*H*$_{Pro}$C(O) + NHC*H*C(O)), 3.74 – 3.71 (m, 3H, OCH$_3$), 2.28 (app. br s, 1H, CHCH$_2$C*H*H$_{Pro}$CHC(O)), 2.23 – 2.12 (m, 1H, C*H*$_{Val}$(CH$_3$)$_2$), 2.12 – 1.94 (m, 2H, CHC*H*H$_{Pro}$CH$_2$CHC(O) + CHCH$_2$CH*H*$_{Pro}$CHC(O)), 1.89 – 1.77 (m, 1H, CHCH*H*$_{Pro}$CH$_2$CHC(O)), 1.61 – 1.37 (app. m, 9H, C*H*$_{3Boc}$), 1.02 – 0.90 (m, 6H, C*H*$_{3Val}$). **^{13}C NMR** (101 MHz, MeOD-*d*$_4$, 278.2 K, complex mixture of rotamers, signals not fully resolved) δ 176.0 (Cq), 174.9 (Cq), 174.3 (Cq), 173.5 (Cq), 173.4 (Cq), 173.3 (Cq), 173.2 (Cq), 156.0 (Cq), 155.6 (Cq), 154.9 (Cq), 91.0 (CH), 83.0 (Cq), 82.6 (Cq), 82.4 (Cq), 81.4 (Cq), 81.2 (Cq), 76.8 (CH), 76.6 (CH), 62.9 (CH), 62.1 (CH), 61.9 (CH), 61.1 (CH), 60.8 (CH), 59.5 (CH), 59.3 (CH), 59.2 (CH), 59.0 (CH), 56.4 (CH), 52.6 (CH$_3$), 52.6 (CH$_3$), 52.5 (CH$_3$), 47.9 (CH$_3$), 33.3 (CH$_2$), 32.5 (CH$_2$), 32.4 (CH$_2$), 32.20 (CH), 31.8 (CH), 31.6 (CH), 30.1 (CH$_2$), 28.8 (CH$_2$), 28.6 (CH$_3$), 28.44 (CH$_3$), 28.39 (CH$_3$), 19.7 (CH$_3$), 19.6 (CH$_3$), 19.5 (CH$_3$), 18.7 (CH$_3$), 18.5 (CH$_3$), 18.2 (CH$_3$). **IR** (ν_{max}, cm^{-1}) 3327 (m), 2976 (m), 2936 (m), 2111 (s), 1742 (s), 1712 (s), 1525 (m), 1367 (s), 1208 (s), 1157 (s). **HRMS** (nanochip-ESI/LTQ-Orbitrap) m/z: [M + Na]$^+$ Calcd for $C_{16}H_{27}N_5NaO_5^+$ 392.1904; Found 392.1892.

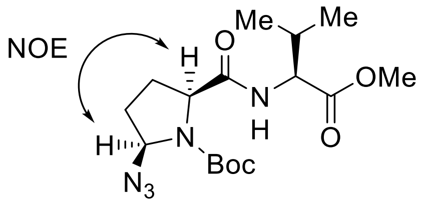

21b (dia maj)

Procedure for the 1.0 mmol scale reaction

An oven dried 20 mL microwave vial equipped with a magnetic stir bar was charged with *tert*-butyl (*S*)-2-(((*S*)-1-methoxy-3-methyl-1-oxobutan-2-yl)carbamoyl)pyrrolidine-1-carboxylate **49** (0.33 g, 1.0 mmol, 1.0 equiv) and 2-iodobenzoic acid (0.50 g, 2.0 mmol, 2.0 equiv). The flask was flushed with nitrogen during few minutes after which 1,2-dichloroethane (10 mL, 0.1 M) was added followed by trimethylsilyl azide (0.28 mL, 2.0 mmol, 2.0 equiv). Solid *m*-chloroperoxybenzoic acid (0.45 g, 2.0 mmol, 2.0 equiv) was finally added in one portion and the vial was sealed and flushed again with nitrogen during few minutes. The heterogeneous mixture was vigorously stirred at 60 °C for 24 hours. After this time, the reaction was cooled down to room temperature, triethylamine (0.70 mL, 5.0 mmol, 5.0 equiv) was added and the mixture was stirred few minutes, filtered over a pad of silica using ethyl acetate to rinse the silica (≈500 mL) and concentrated under reduced pressure. *tert*-butyl (5*S*)-2-azido-5-(((*S*)-1-methoxy-3-methyl-1-oxobutan-2-yl)carbamoyl)pyrrolidine-1-carboxylate **21** (0.11 g (diastereoisomer min **21a**) + 0.18 g (diastereoisomer maj **21b**) = 0.29 g, 0.79 mmol, 79%) (1.8:1 dr) was obtained as yellow oils after purification by column chromatography on silica using a gradient from pentane to pentane/ethyl acetate 7:3 as eluent.

Rf (pentane/ethyl acetate 7:3)$_{diamin}$: 0.47. **Rf** (pentane/ethyl acetate 7:3)$_{diamaj}$: 0.28.

tert-Butyl (5S)-2-azido-5-(((S)-1-(tert-butoxy)-4-methyl-1-oxopentan-2-yl)carbamoyl) pyrrolidine-1-carboxylate (22)

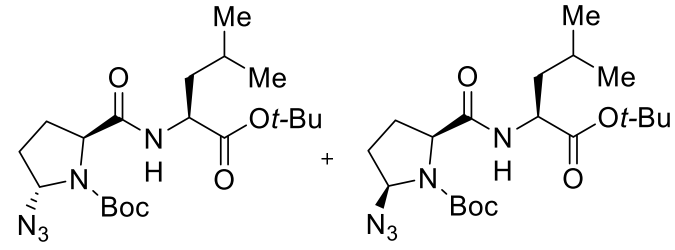

22a **22b**

Synthesized from *tert*-butyl (*S*)-2-(((S)-1-(*tert*-butoxy)-4-methyl-1-oxopentan-2-yl)carbamoyl)pyrrolidine-1-carboxylate **50** (154 mg, 0.400 mmol, 1.00 equiv) following general procedure **F**. *tert*-butyl (5S)-2-azido-5-(((*S*)-1-(tert-butoxy)-4-methyl-1-oxopentan-2-yl)carbamoyl)pyrrolidine-1-carboxylate **22** (56.3 mg (diastereoisomer 1 **22a**) + 54.7 mg (diastereoisomer 2 **22b**) = 111 mg, 0.261 mmol, 65%) (1:1 dr) was obtained as yellow sticky oils after purification by column chromatography on silica using a gradient from pentane to pentane/ethyl acetate 8:2 as eluent.

<u>**22a**: diastereoisomer 1: </u>**Rf** (pentane/ethyl acetate 8:2)$_{dia1}$: 0.58.

^{1}H NMR (400 MHz, MeOD-*d$_4$*, 298 K, complex mixture of rotamers) δ 5.61 (dd, *J* = 9.4, 5.7 Hz, 1H, C*H*N$_3$), 4.37 – 4.31 (m, 1H, NCHCH$_2$CH$_2$C*H*C(O)), 4.31 – 4.22 (m, 1H, NHC*H*$_{Leu}$C(O)), 2.44 – 1.93 (m, 3H, NCHCH$_2$C*H$_2$*CHC(O) + NCHC*H*HC*H$_2$*CHC(O)), 1.87 – 1.69 (m, 2H, NCHCH*H*C*H$_2$*CHC(O)+ CH$_2$C*H*(CH$_3$)$_2$), 1.63 – 1.55 (m, 2H, C*H$_2$*CH(CH$_3$)$_2$), 1.51 (s, 3H, C*H$_{3Ot\text{-}Bu}$*), 1.44 (m, 15H, C*H$_{3Ot\text{-}Bu}$*), 1.02 – 0.87 (m, 6H, C*H$_{3Leu}$*). **^{13}C NMR** (101 MHz, MeOD-*d$_4$*, 298 K, complex mixture of rotamers, signals not fully resolved) δ 174.6 (Cq), 174.2 (Cq), 173.4 (Cq), 173.2 (Cq), 155.7 (Cq), 154.9 (Cq), 91.0 (CH), 83.0 (Cq), 82.8 (Cq), 82.63 (Cq), 82.55 (Cq), 82.4 (Cq), 77.0 (CH), 76.9 (CH), 61.3 (CH), 61.2 (CH), 60.9 (CH), 53.1 (CH), 53.0 (CH), 42.4 (CH$_2$), 41.5 (CH$_2$), 41.4 (CH$_2$), 32.8 (CH$_2$), 31.7 (CH$_2$), 30.5 (CH$_2$), 29.8 (CH$_2$), 29.5 (CH$_2$), 28.6 (CH$_2$), 28.5 (CH$_3$), 28.4 (CH$_3$), 28.2 (CH$_3$), 26.0 (CH), 25.9 (CH), 23.33 (CH$_3$), 23.27 (CH$_3$), 21.9 (CH$_3$), 21.8 (CH$_3$). **IR** (ν_{max}, cm^{-1}) 3342 (m), 2979 (m), 2938 (m), 2113 (s), 1732 (s), 1712 (s), 1382 (s), 1368 (s), 1258 (m), 1158 (s). **HRMS** (ESI/QTOF) m/z: [M + Na]$^+$ Calcd for $C_{20}H_{35}N_5NaO_5^+$ 448.2530; Found 448.2527.

<u>**22b**: diastereoisomers 2: </u>**Rf** (pentane/ethyl acetate 7:3)$_{dia2}$: 0.23.

^{1}H NMR (400 MHz, MeOD-*d$_4$*, 298 K, complex micture of rotamers) δ 5.67 – 5.60 (m, 0.77H, C*H*N$_3$), 5.26 – 5.23 (m, 0.23H, C*H*N$_3$), 4.40 – 4.29 (m, 1H, NHC*H*$_{Leu}$C(O)), 4.28 – 4.18 (m, 1H, NCHCH$_2$CH$_2$C*H*C(O)), 2.45 – 2.14 (m, 1H, NCHCH$_2$C*H*HCHC(O)), 2.12 – 1.91 (m, 2H, NCHCH$_2$CH*H*CHC(O) + NCHC*H*HCH$_2$CHC(O)), 1.91 – 1.68 (m, 2H, C*H*(CH$_3$)$_2$ + NCHCH*H*CH$_2$CHC(O)), 1.65 – 1.56 (m, 2H, CHC*H$_{2Leu}$*CH(CH$_3$)$_2$), 1.55 – 1.38 (app. m, 18H, C*H$_{3Ot\text{-}Bu}$*), 1.01 – 0.88 (m, 6H, C*H$_{3Leu}$*). **^{13}C NMR** (101 MHz, MeOD-*d$_4$*, 298 K, complex micture of rotamers, signals not fully resolved) δ 175.9 (Cq), 175.4 (Cq), 174.6 (Cq), 174.0 (Cq), 173.4

(Cq), 173.2 (Cq), 156.0 (Cq), 155.9 (Cq), 155.7 (Cq), 154.9 (Cq), 91.0 (CH), 90.7 (CH), 82.9 (Cq), 82.8 (Cq), 82.7 (Cq), 82.6 (Cq), 82.5 (Cq), 76.6 (CH), 76.5 (CH), 62.7 (CH), 62.0 (CH), 61.8 (CH), 61.2 (CH), 53.1 (CH), 53.04 (CH), 52.96 (CH), 52.6 (CH), 42.2 (CH_2), 41.8 (CH_2), 41.7 (CH_2), 41.4 (CH_2), 41.3 (CH_2), 33.3 (CH_2), 32.5 (CH_2), 30.8 (CH_2), 30.5 (CH_2), 30.2 (CH_2), 30.0 (CH_2), 28.9 (CH_2), 28.6 (CH_3), 28.5 (CH_3), 28.4 (CH_3), 28.2 (CH_3), 26.0 (CH), 25.8 (CH), 23.4 (CH_3), 23.3 (CH_3), 22.0 (CH_3). **IR** (ν_{max}, cm^{-1}) 3335 (w), 2983 (m), 2930 (m), 2109 (m), 1735 (s), 1709 (s), 1522 (m), 1388 (s), 1368 (s), 1257 (m), 1157 (s). **HRMS** (ESI/QTOF) m/z: $[M + Na]^+$ Calcd for $C_{20}H_{35}N_5NaO_5^+$ 448.2530; Found 448.2532.

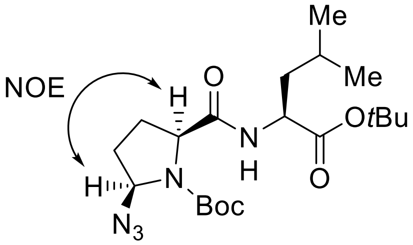

22b

tert-Butyl (5_S_)-2-azido-5-(((_S_)-1-methoxy-1-oxo-3-phenylpropan-2-yl)carbamoyl) pyrrolidine-1-carboxylate (23)

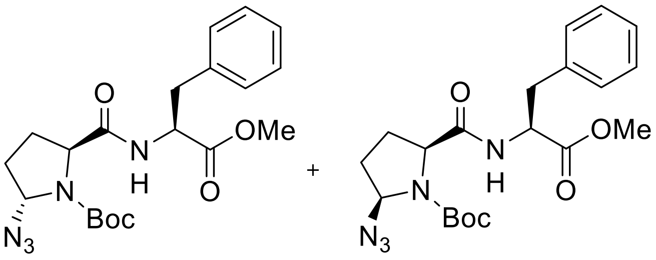

23a (dia min) **23b** (dia maj)

Synthesized from *tert*-butyl (*S*)-2-(((*S*)-1-methoxy-1-oxo-3-phenylpropan-2-yl)carbamoyl)pyrrolidine-1-carboxylate **51** (151 mg, 0.400 mmol, 1.00 equiv) following general procedure **F**. *tert*-butyl (5*S*)-2-azido-5-(((*S*)-1-methoxy-1-oxo-3-phenylpropan-2-yl)carbamoyl)pyrrolidine-1-carboxylate **23** (47.6 mg (diastereoisomer min **23a**) + 65.9 mg (diastereoisomer maj **23b**) = 114 mg, 0.272 mmol, 68%) (1.4:1 dr) was obtained as yellow sticky oils after purification by column chromatography on silica using a gradient from pentane to pentane/ethyl acetate 8:2 as eluent.

<u>**23a**: diastereoisomer min:</u> **Rf** (pentane/ethyl acetate 8:2)$_{diamin}$: 0.21.

^{1}H NMR (400 MHz, MeOD-d_4, mixture of two rotamers) δ 7.34 – 7.11 (m, 5H, Ar*H*), 5.58 (d, *J* = 6.5 Hz, 1H, C*H*N_3), 4.71 – 4.61 (m, 1H, NHC*H*$_{Phe}$C(O)), 4.30 (d, *J* = 9.2 Hz, 0.44H, $NCHCH_2CH_2CH_{rotamermin}$C(O)), 4.23 (d, *J* = 9.2 Hz, 0.57H, $NCHCH_2CH_2CH_{rotamermaj}$C(O)), 3.70 (s, 2H, $OCH_{3rotamermaj}$), 3.65 (s, 1H, $OCH_{3rotamermin}$), 3.25 – 2.87 (m, 2H, C*H*$_2$Ph), 2.35 – 2.18 (m, 1H, NCHC*H*HCH_2CHC(O)), 2.12 – 1.92 (m, 1H, $NCHCH_2$C*H*HCHC(O)), 1.91 – 1.82 (m, 1H, NCHCH*H*CH_2CHC(O)), 1.79 – 1.70 (m, 1H, $NCHCH_2$CH*H*CHC(O)), 1.51 (s, 4H, $CH_{3Bocrotamermin}$),

1.27 (s, 5H, $CH_{3Bocrotamermaj}$). **^{13}C NMR** (101 MHz, MeOD-d_4, 298 K, mixture of two rotamers, signals not fully resolved) δ 174.9 ($Cq_{rotamermaj}$), 174.2 ($Cq_{rotamermin}$), 173.5 ($Cq_{rotamermaj}$), 173.3 ($Cq_{rotamermin}$), 155.6 ($Cq_{rotamermaj}$), 154.9 ($Cq_{rotamermin}$), 138.3 ($Cq_{rotamermaj}$), 138.0 ($Cq_{rotamermin}$), 130.5 (CH), 130.3 (CH), 130.1 (CH), 129.6 (CH), 129.5 (CH), 128.0 (CH), 127.9 (CH), 83.1 ($Cq_{rotamermin}$), 82.5 ($Cq_{rotamermaj}$), 77.0 ($CH_{rotamermin}$), 76.9 ($CH_{rotamermaj}$), 61.4 ($CH_{rotamermaj}$), 61.0 ($CH_{rotamermin}$), 55.4 ($CH_{rotamermaj}$), 55.3 ($CH_{rotamermin}$), 52.7 ($CH_{3rotamermaj}$), 52.6 ($CH_{3rotamermin}$), 38.3 ($CH_{2rotamermin}$), 38.1 ($CH_{2rotamermaj}$), 32.8 (CH_2), 31.6 (CH_2), 29.6 (CH_2), 28.5 (CH_3), 28.3 (CH_3). **IR** (ν_{max}, cm^{-1}) 3329 (w), 2981 (w), 2110 (s), 1701 (s), 1523 (m), 1373 (s), 1254 (m), 1203 (s), 1161 (s), 741 (m), 702 (m). **HRMS** (ESI/QTOF) m/z: [M + Na]$^+$ Calcd for $C_{20}H_{27}N_5NaO_5^+$ 440.1904; Found 440.1905.

<u>**23b**: diastereoisomer maj: </u>**Rf** (pentane/ethyl acetate 8:2)$_{diamaj}$: 0.09.

^{1}H NMR (400 MHz, MeOD-d_4, 298 K mixture of rotamers) δ 7.44 – 7.02 (m, 5H, Ar*H*), 5.62 (d, *J* = 5.2 Hz, 0.88H, C*H*N$_3$), 5.20 (d, *J* = 3.8 Hz, 0.12H, C*H*N$_3$), 4.77 – 4.70 (m, 1H, NHCH_{Phe}C(O)), 4.18 (app. br s, 1H, NCHCH$_2$CH$_2$C*H*C(O)), 3.81 – 3.60 (app. m, 3H, OCH_3), 3.25 – 2.93 (m, 2H, CH_2Ph), 2.25 – 2.22 (m, 1H, NCHCH$_2$C*H*HCHC(O)), 2.01 (app. br s, 1H, NCHC*H*HCH$_2$CHC(O)), 1.89 (app. br s, 1H, NCHCH$_2$CH*H*CHC(O)), 1.76 (app. br s, 1H, NCHCH$_2$CH*H*CHC(O)), 1.61 – 1.19 (app. m, 9H, CH_{3Boc}). **^{13}C NMR** (101 MHz, MeOD-d_4, 278.2 K, complex mixture of rotamers, signals not fully resolved) δ 174.8 (Cq), 174.7 (Cq), 174.1 (Cq), 174.0 (Cq), 173.2 (Cq), 173.1 (Cq), 156.0 (Cq), 155.6 (Cq), 154.9 (Cq), 138.3 (Cq), 137.9 (Cq), 137.7 (Cq), 130.5 (CH), 130.3 (CH), 130.1 (CH), 129.7 (CH), 129.6 (CH), 129.5 (CH), 128.1 (CH), 128.0 (CH), 83.0 (Cq), 82.6 (Cq), 81.5 (Cq), 76.8 (CH), 76.7 (CH), 62.2 (CH), 62.0 (CH), 61.5 (CH), 55.5 (CH), 55.4 (CH), 55.2 (CH), 55.1 (CH), 52.8 (CH_3), 52.74 (CH_3), 52.67 (CH_3), 38.5 (CH_2), 38.3 (CH_2), 33.2 (CH_2), 32.4 (CH_2), 32.2 (CH_2), 29.9 (CH_2), 28.9 (CH_2), 28.7 (CH_2), 28.5 (CH_3), 28.2 (CH_3). **IR** (ν_{max}, cm^{-1}) 3383 (w), 2981 (w), 2951 (w), 2110 (m), 1747 (m), 1697 (s), 1520 (m), 1377 (s), 1165 (m), 741 (m), 706 (w). **HRMS** (ESI/QTOF) m/z: [M + Na]$^+$ Calcd for $C_{20}H_{27}N_5NaO_5^+$ 440.1904; Found 440.1896.

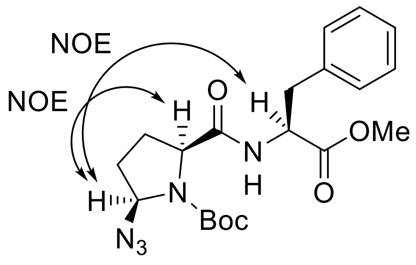

23b (dia maj)

tert-Butyl (5_S_)-2-azido-5-(((_S_)-3-((_tert_-butyldimethylsilyl)oxy)-1-methoxy-1-oxopropan-2-yl)carbamoyl)pyrrolidine-1-carboxylate (24)

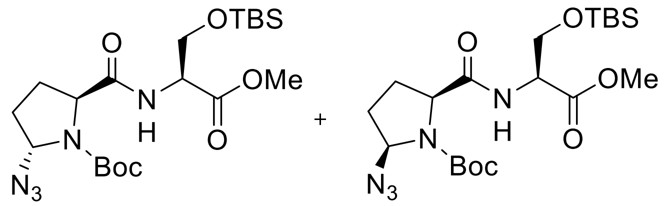

24a (dia min) **24b** (dia maj)

Synthesized from _tert_-butyl (_S_)-2-(((_S_)-3-((_tert_-butyldimethylsilyl)oxy)-1-methoxy-1-oxopropan-2-yl)carbamoyl)pyrrolidine-1-carboxylate **52** (172 mg, 0.400 mmol, 1.00 equiv) following general procedure **F**. _tert_-butyl (5_S_)-2-azido-5-(((_S_)-3-((_tert_-butyldimethylsilyl)oxy)-1-methoxy-1-oxopropan-2-yl)carbamoyl)pyrrolidine-1-carboxylate **24** (52.1 mg (diastereoisomer min **24a**) + 92.7 mg (diastereoisomer maj **24b**) = 145 mg, 0.307 mmol, 77%) (1.8:1 dr) was obtained as clear oils after purification by column chromatography on silica using a gradient from pentane to pentane/ethyl acetate 8:2 as eluent.

24a: diastereoisomer min: **Rf** (pentane/ethyl acetate 8:2)$_{diamin}$: 0.30.

^{1}H NMR (400 MHz, MeOD-d_4, 298 K, mixture of two rotamers) δ 5.62 (dd, _J_ = 11.3, 5.8 Hz, 1H, C_H_N$_3$), 4.60 – 4.50 (m, 1H, NHCH_{Ser}C(O)), 4.43 (d, _J_ = 9.1 Hz, 1H, NCHCH$_2$CH$_2$C_H_C(O)), 4.11 – 4.00 (m, 1H, C_H_HOTBS), 3.94 – 3.81 (m, 1H, CH_H_OTBS), 3.75 – 3.71 (m, 3H, OCH$_3$), 2.45 – 2.23 (m, 1H, NCHCH$_2$C_H_HCHC(O)), 2.23 – 2.08 (m, 1H, NCHC_H_HCH$_2$CHC(O)), 2.08 – 1.95 (m, 1H, NCHCH$_2$CH_H_CHC(O)), 1.88 – 1.74 (m, 1H, NCHCH_H_CH$_2$CHC(O)), 1.51 (s, 4H, C$H_{3Bocrotamermin}$), 1.44 (s, 5H, C$H_{3Bocrotamermaj}$), 0.95 – 0.83 (m, 9H, (CH_3)$_{3TBS}$), 0.12 – 0.01 (m, 6H, CH_{3TBS}). **^{13}C NMR** (101 MHz, MeOD-d_4, 278.2 K, mixture of two rotamers, signals not fully resolved) δ 174.8 (Cq), 174.4 (Cq), 172.1 (Cq), 171.8 (Cq), 155.6 (Cq), 155.0 (Cq), 83.0 (Cq), 82.4 (Cq), 76.9 (CH), 76.9 (CH), 64.5 (CH$_2$), 64.3 (CH$_2$), 61.2 (CH), 60.8 (CH), 56.1 (CH), 56.1 (CH), 52.8 (CH$_3$), 52.7 (CH$_3$), 32.8 (CH$_2$), 31.8 (CH$_2$), 29.8 (CH$_2$), 28.7 (CH$_2$), 28.5 (CH$_3$), 28.3 (CH$_3$), 26.28 (CH$_3$), 26.25 (CH$_3$), 26.2 (CH$_3$), 19.2 (Cq), 19.1 (Cq), -5.4 (CH$_3$), -5.4 (CH$_3$), -5.4 (CH$_3$), -5.5 (CH$_3$), -5.6 (CH$_3$). **IR** (ν_{max}, cm^{-1}) 2954 (m), 2931 (m), 2884 (m), 2859 (m), 2110 (s), 1751 (s), 1712 (s), 1380 (s), 1259 (s), 1162 (s). **HRMS** (nanochip-ESI/LTQ-Orbitrap) m/z: [M + Na]$^+$ Calcd for $C_{20}H_{37}N_5NaO_6Si^+$ 494.2405; Found 494.2402.

24b: diastereoisomer maj: **Rf** (pentane/ethyl acetate 7:3)$_{diamaj}$: 0.15.

^{1}H NMR (400 MHz, MeOD-d_4, 298 K, complex mixture of rotamers) δ 5.68 (dd, _J_ = 5.8, 2.0 Hz, 0.84H, C_H_N$_3$), 5.27 (d, _J_ = 4.6 Hz, 0.16H, C_H_N$_3$), 4.62 – 4.50 (m, 1H, NHCH_{Ser}C(O)), 4.36 – 4.21 (m, 1H, NCHCH$_2$CH$_2$C_H_C(O)), 4.15 – 4.01 (m, 1H, C_H_HOTBS)), 3.90 – 3.82 (m, 1H, CH_H_OTBS), 3.77 – 3.73 (m, 3H, OCH_3), 2.44 – 2.18 (m, 1H, NCHCH$_2$C_H_HCHC(O)), 2.17 – 1.95 (m, 2H, NCHCH$_2$CH_H_CHC(O) + NCHC_H_HCH$_2$CHC(O)), 1.94 – 1.78 (m, 1H, NCHCH_H_CH$_2$CHC(O)), 1.59 – 1.39 (app. m, 9H, CH_{3Boc}), 0.92 – 0.87 (m, 9H, (CH_3)$_{3TBS}$), 0.10 – 0.03 (m, 6H, CH_{3TBS}). **^{13}C NMR** (101 MHz, MeOD-d_4, 298 K, complex mixture of rotamers, signals not fully resolved) δ 175.7 (Cq), 174.7 (Cq), 174.2 (Cq), 172.0 (Cq), 171.8 (Cq), 171.6 (Cq), 156.0 (Cq), 155.5 (Cq), 154.9

(Cq), 83.2 (Cq), 82.8 (Cq), 81.6 (Cq), 81.3 (Cq), 77.1 (CH), 77.0 (CH), 64.4 (CH_2), 64.4 (CH_2), 62.6 (CH), 62.3 (CH), 61.5 (CH), 61.1 (CH), 55.91 (CH), 55.89 (CH), 52.89 (CH_3), 52.87 (CH_3), 52.8 (CH_3), 33.3 (CH_2), 32.43 (CH_2), 32.38 (CH_2), 31.2 (CH_2), 30.1 (CH_2), 29.0 (CH_2), 28.7 (CH_3), 28.6 (CH_3), 28.5 (CH_3), 28.4 (CH_3), 26.2 (CH_3), 26.19 (CH_3), 26.17 (CH_3), 25.3 (CH_2), 24.6 (CH_2), 19.13 (CH), 19.07 (CH), 19.0 (CH), -5.39 (CH_3), -5.44 (CH_3), -5.6 (CH_3), -5.6 (CH_3), -5.6 (CH_3). **IR** (ν_{max}, cm^{-1}) 2955 (s), 2936 (s), 2898 (m), 2859 (m), 2111 (s), 1752 (s), 1705 (s), 1520 (m), 1380 (s), 1255 (s), 1163 (s), 1115 (s). **HRMS** (ESI/QTOF) m/z: $[M + Na]^+$ Calcd for $C_{20}H_{37}N_5NaO_6Si^+$ 494.2405; Found 494.2410.

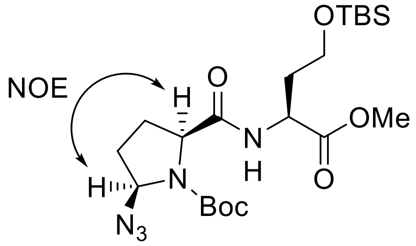

24b (dia maj)

di-*tert*-Butyl ((2*S*)-5-azido-1-(*tert*-butoxycarbonyl)pyrrolidine-2-carbonyl)-*L*-glutamate (25)

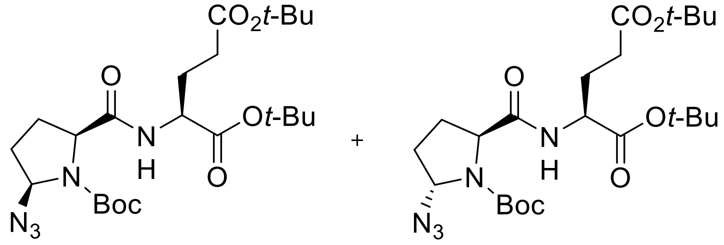

25a (dia maj) **25a** (dia min)

Synthesized from di-*tert*-butyl (*tert*-butoxycarbonyl)-*L*-prolyl-*L*-glutamate **53** (183 mg, 0.400 mmol, 1.00 equiv) following general procedure **F**. Di-*tert*-butyl ((2*S*)-5-azido-1-(*tert*-butoxycarbonyl)pyrrolidine-2-carbonyl)-*L*-glutamate **25** (64.8 mg (diastereoisomer min **25a**) + 76.1 mg (diastereoisomer maj **25b**) = 141 mg, 0.280 mmol, 71%) (1.2:1 dr) was obtained as clear oils after purification by column chromatography on silica using a gradient from pentane to pentane/ethyl acetate 8:2 as eluent.

<u>**25a**: diastereoisomer min: </u>**Rf** (pentane/ethyl acetate 8:2)$_{diamin}$: 0.37.

^{1}H NMR (400 MHz, MeOD-*d$_4$*, 278.2 K, mixture of rotamers) δ 5.64 (t, *J* = 5.1 Hz, 1H, C*H*N_3), 4.32 (dd, *J* = 8.9, 2.6 Hz, 1H, NHC*H*$_{Glu}$C(O)), 4.27 (dd, *J* = 9.6, 4.9 Hz, 1H, NCHCH_2CH_2C*H*$_{Pro}$C(O)), 2.50 – 2.22 (m, 3H, NHCHC*H$_2$*$_{Glu}$$CH_2$C(O)O*t*-Bu + NHCHC*H$_2$*CH*H*$_{Glu}$C(O)O*t*-Bu), 2.22 – 1.96 (m, 3H, NCHC*H$_2$*CH_2C*H*$_{Pro}$C(O) + NHCHC*H$_2$*C*H*H$_{Glu}$C(O)O*t*-Bu), 1.91 – 1.76 (m, 2H, NCHCH_2C*H$_2$*C*H*$_{Pro}$C(O)), 1.57 – 1.41 (m, 27H, $CH_{3Ot\text{-}Bu}$). **^{13}C NMR** (101 MHz, MeOD-*d$_4$*, 278.2 K, complex mixture of three rotamers, signals not fully resolved) δ 174.7 (Cq), 174.4 (Cq), 173.8 (Cq), 173.3 (Cq), 172.3 (Cq), 172.2 (Cq), 155.6 (Cq), 154.9 (Cq), 83.0 (Cq), 82.9 (Cq), 82.8 (Cq), 82.5 (Cq), 81.8 (Cq), 81.6 (Cq), 76.91 (CH), 76.88 (CH), 61.2 (CH), 60.9 (CH), 53.70 (CH), 53.67

(CH), 32.8 (CH_2), 32.5 (CH_2), 32.3 (CH_2), 31.7 (CH_2), 29.7 (CH_2), 28.6 (CH_2), 28.51 (CH_3), 28.46 (CH_3), 28.3 (CH_3), 28.2 (CH_3), 27.8 (CH_2), 27.7 (CH_2). **IR** (ν_{max}, cm^{-1}) 3336 (w), 2979 (m), 2109 (m), 1731 (m), 1709 (s), 1536 (w), 1368 (s), 1257 (m), 1156 (s). **HRMS** (nanochip-ESI/LTQ-Orbitrap) m/z: $[M + Na]^+$ Calcd for $C_{23}H_{39}N_5NaO_7^+$ 520.2742; Found 520.2727.

25b: diastereoisomer maj: **Rf** (pentane/ethyl acetate 7:3)$_{diamaj}$: 0.17.

^{1}H NMR (400 MHz, MeOD-*d*$_4$, 278.2 K, complex mixture of rotamers) δ 5.68 – 5.61 (m, 1H, C*H*N_3), 4.42 – 4.16 (m, 2H, NHCH_{Glu}C(O) + NCHCH$_2$CH$_2$CH_{Pro}C(O)), 2.46 – 2.19 (m, 3H, CH_{2Pro} and/or CH_{2Glu}), 2.16 – 1.77 (m, 5H, CH_{2Pro} and/or CH_{2Glu}), 1.56 – 1.37 (m, 27H, $CH_{3Ot\text{-}Bu}$). **^{13}C NMR** (101 MHz, MeOD-*d*$_4$, 278.2 K, complex mixture of rotamers, signals not fully resolved) δ 174.4 (Cq), 173.2 (Cq), 172.7 (Cq), 172.5 (Cq), 172.0 (Cq), 171.91 (Cq), 170.88 (Cq), 170.80 (Cq), 170.76 (Cq), 154.6 (Cq), 154.2 (Cq), 153.5 (Cq), 81.6 (Cq), 81.54 (Cq), 81.50 (Cq), 81.4 (Cq), 81.2 (Cq), 80.4 (Cq), 80.2 (Cq), 80.1 (Cq), 79.8 (Cq), 75.2 (CH), 74.9 (CH), 60.7 (CH), 60.5 (CH), 59.9 (CH), 52.30 (CH), 52.25 (CH), 52.1 (CH), 32.0 (CH_2), 31.14 (CH_2), 31.07 (CH_2), 31.0 (CH_2), 30.9 (CH_2), 30.8 (CH_2), 30.0 (CH_2), 28.8 (CH_2), 27.5 (CH_2), 27.33 (CH_2), 27.26 (CH_2), 27.2 (CH_3), 27.1 (CH_3), 26.9 (CH_3), 26.81 (CH_3), 26.80 (CH_3), 26.76 (CH_2), 26.6 (CH_2), 26.3 (CH_2), 24.0 (CH_2), 23.2 (CH_2). **IR** (ν_{max}, cm^{-1}) 3323 (w), 2982 (m), 2924 (w), 2111 (m), 1733 (s), 1705 (s), 1392 (m), 1368 (s), 1254 (m), 1158 (s). **HRMS** (ESI/QTOF) m/z: $[M + Na]^+$ Calcd for $C_{23}H_{39}N_5NaO_7^+$ 520.2742; Found 520.2745.

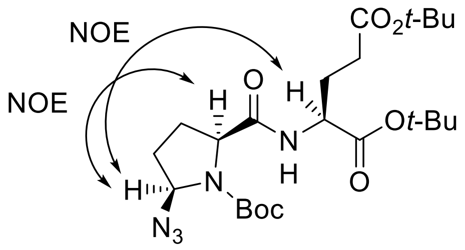

25b (dia maj)

tert-Butyl (5*S*)-2-azido-5-(((*S*)-6-(((benzyloxy)carbonyl)amino)-1-methoxy-1-oxohexan-2-yl)carbamoyl)pyrrolidine-1-carboxylate (26)

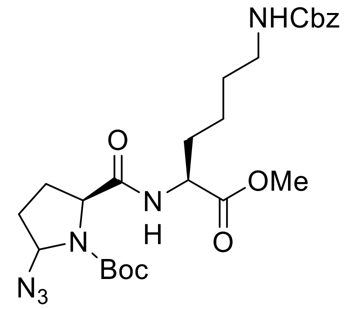

Synthesized from *tert*-butyl (*S*)-2-(((*S*)-6-(((benzyloxy)carbonyl)amino)-1-methoxy-1-oxohexan-2-yl)carbamoyl)pyrrolidine-1-carboxylate **54** (197 mg, 0.400 mmol, 1.00 equiv) following general procedure **F**. *tert*-butyl (5*S*)-2-azido-5-(((*S*)-6-(((benzyloxy)carbonyl)amino)-

1-methoxy-1-oxohexan-2-yl)carbamoyl)pyrrolidine-1-carboxylate **26** (124 mg, 0.130 mmol, 58%) (mixture of diastereoisomers, n.d. dr) was obtained as yellowish solid after purification by column chromatography on silica using a gradient from dichloromethane to dichloromethane/methanol 96:4 as eluent.

Rf (dichloromethane/methanol 96:4): 0.29. **^{1}H NMR** (400 MHz, Methanol-d_4, 298 K, comlex mixture of diastereoisomers and rotamers) δ 7.46 – 7.19 (m, 5H, Ar*H*), 5.67 – 5.57 (m, 1H, C*H*N_3), 5.07 – 5.05 (m, 2H, OC*H$_2$*Ph), 4.51 – 4.18 (m, 2H, NHC*H*$_{Lys}$C(O) + N$CH_2CH_2CH_2$C*H*C(O)), 3.75 – 3.67 (m, 3H, OC*H$_3$*), 3.15 – 3.09 (m, 2H, $CH_2CH_2CH_2$C*H*$_{2Lys}$NHCBz), 2.49 – 1.62 (m, 6H, NCHC*H$_2$*C*H$_2$*CHC(O) + C*H*$_{2Lys}$$CH_2CH_2CH_2$NHCBz), 1.60 – 1.32 (m, 13H, CH_2C*H$_2$*C*H*$_{2Lys}$$CH_2$NHCBz + OC*H*$_{3Boc}$). **^{13}C NMR** (101 MHz, MeOD-d_4, 278.2 K, complex mixture of diastereoisomers and rotamers) δ 174.9 (Cq), 174.7 (Cq), 174.5 (Cq), 174.2 (Cq), 174.0 (Cq), 174.0 (Cq), 173.9 (Cq), 159.8 (Cq) 158.9 (Cq), 158.9 (Cq), 155.6 (Cq), 154.9 (Cq), 138.4 (Cq), 138.4 (Cq), 129.6 (CH), 129.49 (CH), 129.47 (CH), 129.45 (CH), 129.00 (CH), 128.97 (CH), 128.95 (CH), 128.83 (CH), 128.80 (CH), 83.0 (Cq), 82.9 (Cq), 82.5 (Cq), 82.4 (Cq), 76.9 (CH), 76.9 (CH), 76.5 (CH), 76.3 (CH), 67.3 (CH_2), 67.2 (CH_2), 62.0 (CH), 61.8 (CH), 61.2 (CH), 60.8 (CH), 53.9 (CH), 53.8 (CH), 53.5 (CH), 52.8 (CH_3), 52.71 (CH_3), 52.68 (CH_3), 41.5 (CH_2), 41.4 (CH_2), 41.3 (CH_2), 33.3 (CH_2), 32.8 (CH_2), 32.5 (CH_2), 32.4 (CH_2), 32.1 (CH_2), 31.90 (CH_2), 31.85 (CH_2), 31.7 (CH_2), 30.8 (CH_2), 30.4 (CH_2), 30.2 (CH_2), 30.0 (CH_2), 29.7 (CH_2), 28.9 (CH_2), 28.7 (CH_2), 28.5 (CH_3), 28.4 (CH_3), 24.6 (CH_2), 24.3 (CH_2), 24.2 (CH_2), 23.9 (CH_2), 23.8 (CH_2), 19.8 (CH_2). **IR** (ν_{max}, cm^{-1}) 3338 (m), 2954 (m), 2925 (m), 2111 (s), 1733 (s), 1708 (s), 1455 (m), 1433 (m), 1386 (m), 1368 (m), 1261 (m), 1162 (m), 740 (m), 697 (m). **HRMS** (ESI/QTOF) m/z: [M + Na]$^+$ Calcd for $C_{25}H_{36}N_6NaO_7^+$ 555.2538; Found 555.2546.

Methyl (2-azido-2-((*tert*-butoxycarbonyl)amino)acetyl)-*L*-valinate (27)

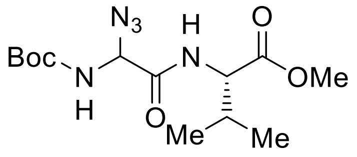

Synthesized from methyl (*tert*-butoxycarbonyl)glycyl-*L*-valinate **55** (115 mg, 0.400 mmol, 1.00 equiv) following general procedure **F**. Methyl (2-azido-2-((*tert*-butoxycarbonyl)amino)acetyl)-*L*-valinate **27** (54.0 mg, 0.164 mmol, 41%) (mixture of diastereoisomers, n.d. dr) was obtained as a yellow oil after purification by column chromatography on silica using a gradient from pentane to pentane/ethyl acetate 8:2 as eluent.

Rf (pentane/ethyl acetate 8:2): 0.43. **^{1}H NMR** (400 MHz, MeOD-d_4, 298 K, complex mixture of diastereoisomers and rotamers) δ 5.52 (s, 0.5H, C*H*N_3), 5.50 (s, 0.5H, C*H*N_3), 4.37 – 4.32 (m, 1H, NHC*H*$_{Val}$C(O)), 3.74 – 3.73 (m, 3H, OC*H$_3$*), 2.28 – 2.13 (m, 1H, NHCHC*H*$_{Val}$(CH_3)$_2$), 1.53 (s, 1H, C*H*$_{3Boc}$), 1.48 (s, 8H, C*H*$_{3Boc}$), 0.97 – 0.95 (m, 6H, C*H*$_{3Val}$). **^{13}C NMR** (101 MHz, $CDCl_3$, 278.2 K, complex mixture of diastereoisomers and rotamers, signals not fully resolved) δ 171.8 (Cq), 170.9 (Cq), 165.8 (Cq), 165.5 (Cq), 158.3 (Cq), 157.5 (Cq), 155.3 (Cq), 155.2 (Cq), 148.6 (Cq), 83.8 (Cq), 81.52 (Cq), 81.51 (Cq), 81.50 (Cq), 67.4 (CH), 67.2 (CH), 58.1 (CH), 57.7 (CH), 57.6

(CH), 52.71 (CH_3), 52.70 (CH_3), 52.65 (CH_3), 31.5 (CH), 31.5 (CH), 31.4 (CH), 28.2 (CH_3), 28.0 (CH_3), 19.11 (CH_3), 19.08 (CH_3), 19.0 (CH_3), 17.8 (CH_3), 17.7 (CH_3), 17.7 (CH_3). **IR** (ν_{max}, cm^{-1}) 3329 (m), 2974 (m), 2110 (s), 1732 (s), 1682 (s), 1489 (s), 1369 (m), 1254 (w), 1215 (m), 1149 (s). **HRMS** (ESI/QTOF) m/z: $[M + Na]^+$ Calcd for $C_{13}H_{23}N_5NaO_5^+$ 352.1591; Found 352.1589.

Methyl (2-azido-2-((*tert*-butoxycarbonyl)amino)acetyl)-*L*-leucinate (28)

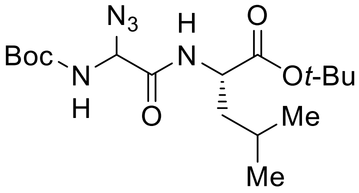

Synthesized from *tert*-butyl (*tert*-butoxycarbonyl)glycyl-*L*-leucinate **56** (138 mg, 0.400 mmol, 1.00 equiv) following general procedure **F**. Methyl (2-azido-2-((*tert*-butoxycarbonyl)amino)acetyl)-*L*-leucinate **28** (52.4 mg, 0.136 mmol, 34%) (mixture of diastereoisomers, n.d. dr) was obtained as a yellow oil after purification by column chromatography on silica using a gradient from pentane to pentane/ethyl acetate 9:1 as eluent.

Rf (pentane/ethyl acetate 9:1): 0.31. **^{1}H NMR** (400 MHz, MeOD-d_4, 298 K, mixture of diastereoisomers and rotamers) δ 5.44 (d, *J* = 14.8 Hz, 1H, C*H*N_3), 4.43 – 4.20 (m, 1H, NHCH_{Leu}C(O)), 1.77 – 1.64 (m, 1H, $CH_2CH(CH_3)_2$), 1.61 (m, 2H, $CH_2CH(CH_3)_2$), 1.50 – 1.45 (m, 18H, CH_{3OtBu}), 0.97 (dd, *J* = 6.5, 2.7 Hz, 3H, $(CH_3)_{2Leu}$), 0.92 (dd, *J* = 6.4, 0.9 Hz, 3H, $(CH_3)_{2Leu}$). **^{13}C NMR** (101 MHz, MeOD-d_4, 298 K, mixture of diastereoisomers and rotamers, signals not fully resolved) δ 173.22 (Cq), 173.15 (Cq), 173.0 (Cq), 172.9 (Cq), 172.4 (Cq), 170.2 (Cq), 167.9 (Cq), 160.4 (Cq), 157.8 (Cq), 151.3 (Cq), 84.2 (Cq), 83.2 (Cq), 83.2 (Cq), 83.02 (Cq), 82.98 (Cq), 82.95 (Cq), 82.4 (CH), 82.3 (CH), 81.7 (CH) 81.1 (CH), 68.5 (CH), 53.4 (CH), 53.3 (CH), 53.2 (CH), 53.0 (CH), 52.9 (CH), 41.6 (CH_2), 41.5 (CH_2), 41.4 (CH_2), 41.3 (CH_2), 28.6 (CH_3), 28.5 (CH_3), 28.2 (CH_3), 28.2 (CH_3), 28.2 (CH_3), 26.1 (CH_3), 26.0 (CH_3), 26.0 (CH_3), 25.9 (CH_3), 23.2 (CH), 23.2 (CH), 22.0 (CH_3), 21.92 (CH_3), 21.85 (CH_3). **IR** (ν_{max}, cm^{-1}) 3312 (m), 2985 (m), 2116 (s), 1736 (s), 1679 (s), 1485 (s), 1370 (s), 1251 (m), 1155 (s). **HRMS** (ESI/QTOF) m/z: $[M + Na]^+$ Calcd for $C_{17}H_{31}N_5NaO_5^+$ 408.2217; Found 408.2215.

***tert*-Butyl (5*S*)-2-azido-5-((2-(((*S*)-1-((2-methoxy-2-oxoethyl)amino)-4-methyl-1-oxopentan-2-yl)amino)-2-oxoethyl)carbamoyl)pyrrolidine-1-carboxylate (29)**

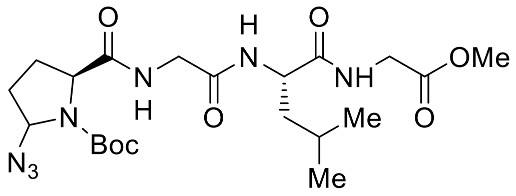

Synthesized from *tert*-butyl (*S*)-2-((2-(((*S*)-1-((2-methoxy-2-oxoethyl)amino)-4-methyl-1-oxopentan-2-yl)amino)-2-oxoethyl)carbamoyl)pyrrolidine-1-carboxylate **57** (183 mg, 0.400

mmol, 1.00 equiv) following general procedure **F**. *tert*-butyl (5*S*)-2-azido-5-((2-(((*S*)-1-((2-methoxy-2-oxoethyl)amino)-4-methyl-1-oxopentan-2-yl)amino)-2-oxoethyl)carbamoyl) pyrrolidine-1-carboxylate **29** (64.9 mg, 0.130 mmol, 33%) (mixture of diastereoisomers, n.d. dr) was obtained as yellowish solid after purification by column chromatography on silica using a gradient from dichloromethane to dichloromethane/methanol 96:4 as eluent.

Rf (dichloromethane/methanol 96:4): 0.24. **^{1}H NMR** (400 MHz, MeOD-*d*$_4$, 278.2 K, complex mixture of diastereoisomers and rotamers) δ 5.74 – 5.55 (m, 1H, C*H*N$_3$), 4.54 – 4.36 (m, 1H, NHC*H*$_{Leu}$C(O)), 4.37 – 4.26 (m, 0.46H, NHCHCH$_2$CH$_2$C*H*$_{Pro}$C(O)), 4.24 – 4.12 (m, 0.53H, NHCHCH$_2$CH$_2$C*H*$_{Pro}$C(O)), 4.07 – 3.75 (m, 4H, C*H*$_{2Gly}$), 3.71 (s, 3H, OC*H*$_3$), 2.43 – 2.12 (m, 2H, NHCHC*H*H$_{Pro}$CH$_2$CHC(O) + NHCHCH$_2$CH*H*$_{Pro}$CHC(O)), 2.10 – 1.92 (m, 2H, NHCHCH*H*$_{Pro}$CH$_2$CHC(O) + NHCHCH$_2$C*H*H$_{Pro}$CHC(O)), 1.92 – 1.57 (m, 3H, NHCHC*H*$_{2Leu}$C*H*(CH$_3$)$_2$ + NHCHCH$_2$C*H*$_{Leu}$(CH$_3$)$_2$), 1.56 – 1.40 (m, 9H, C*H*$_{3Boc}$), 1.02 – 0.81 (m, 6H, C*H*$_{3Leu}$). **^{13}C NMR** (101 MHz, MeOD-*d*$_4$, 278.2 K, complex mixture of diastereoisomers and rotamers, signals not fully resolved) δ 175.1 (Cq), 175.0 (Cq), 174.03(Cq), 173.98 (Cq), 173.9 (Cq), 173.84 (Cq), 173.82 (Cq), 173.75 (Cq), 173.5 (Cq), 170.3 (Cq), 170.11 (Cq), 170.05 (Cq), 170.04 (Cq), 170.01 (Cq), 169.74 (Cq), 169.70 (Cq), 169.65 (Cq), 155.2 (Cq), 154.1 (Cq), 153.9 (Cq), 153.8 (Cq), 82.0 (Cq), 81.9 (Cq), 81.17 (Cq), 80.15 (Cq), 80.1 (Cq), 75.7 (CH), 75.5 (CH), 75.20 (CH), 75.16 (CH), 61.2 (CH), 61.0 (CH), 60.5 (CH), 60.4 (CH), 60.1 (CH), 59.8 (CH), 51.67 (CH), 51.65 (CH), 51.6 (CH), 51.5 (CH), 51.3 (CH), 51.2 (CH), 42.3 (CH$_2$), 42.1 (CH$_2$), 41.8 (CH$_2$), 40.6 (CH$_2$), 40.44 (CH$_2$), 40.39 (CH$_2$), 40.37 (CH$_2$), 40.3 (CH$_2$), 31.9 (CH$_2$), 31.5 (CH$_2$), 31.2 (CH$_2$), 31.0 (CH$_2$), 30.4 (CH$_2$), 30.1 (CH$_2$), 28.6 (CH$_2$), 28.3 (CH$_2$), 27.6 (CH$_3$), 27.4 (CH$_3$), 27.3 (CH$_3$), 27.17 (CH$_2$), 27.15 (CH$_3$), 27.0 (CH$_3$), 24.4 (CH), 24.30 (CH), 24.29 (CH), 24.1 (CH), 23.3 (CH), 22.1 (CH$_3$), 22.1 (CH$_3$), 22.04 (CH$_3$), 21.99 (CH$_3$), 20.7 (CH$_3$), 20.6 (CH$_3$), 20.5 (CH$_3$), 20.4 (CH$_3$). **IR** (ν_{max}, cm^{-1}) 3290 (w), 2954 (w), 2110 (m), 1755 (w), 1651 (s), 1527 (m), 1381 (m). **HRMS** (ESI/QTOF) m/z: [M + Na]$^+$ Calcd for $C_{21}H_{35}N_7NaO_7^+$ 520.2490; Found 520.2508.

tert-Butyl (5*S*)-2-azido-5-(((*S*)-1-(((*S*)-1-(((*S*)-1-methoxy-4-methyl-1-oxopentan-2-yl)amino)-4-methyl-1-oxopentan-2-yl)amino)-3-methyl-1-oxobutan-2-yl)carbamoyl)pyrrolidine-1-carboxylate (30)

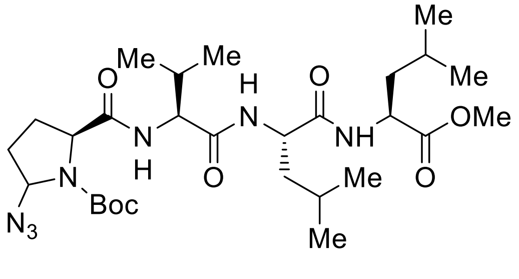

Synthesized from *tert*-butyl (*S*)-2-(((*S*)-1-(((*S*)-1-(((*S*)-1-methoxy-4-methyl-1-oxopentan-2-yl)amino)-4-methyl-1-oxopentan-2-yl)amino)-3-methyl-1-oxobutan-2-yl)carbamoyl) pyrrolidine-1-carboxylate **58** (222 mg, 0.400 mmol, 1.00 equiv) following general procedure **F**. *tert*-butyl (5*S*)-2-azido-5-(((*S*)-1-(((*S*)-1-(((*S*)-1-methoxy-4-methyl-1-oxopentan-2-yl)amino)-4-methyl-1-oxopentan-2-yl)amino)-3-methyl-1-oxobutan-2-yl)carbamoyl)pyrrolidine-1-carboxylate **30** was observed in a 51% ^{1}H NMR yield using mesitylene (40.0 μL, 0.287, 0.719

equiv) as internal standard. The yield was determined using the peaks corresponding to the C*H*N_3. **^{1}H NMR** (400 MHz, MeOD-*d*$_4$, 298 K, complex mixture of diastereomers and rotamers) δ 5.67 (dd, *J* = 5.7, 2.1 Hz, 0.40H, C*H*N_3), 5.62 (dd, *J* = 11.0, 5.6 Hz, 0.60H, C*H*N_3). Only characteristic peaks are listed as the crude ^{1}H NMR was too complex to give the complete ^{1}H NMR listing.

4.3 Yields evalutation and characterization data for compounds 31, 32 and 33

The yields of compounds **31**, **32** and **33** were estimated using the RP-HPLC UV calibration done on starting materials **59**, **60** and **61**, respectively (see section 2.3).

Boc-(N_3)Pro-Val-Leu-Phe-GLy-OMe (31)

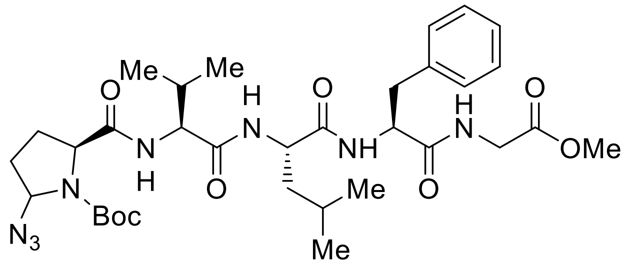

Synthesized from Boc-Pro-Val-Leu-Phe-GLy-OMe **59** (64.6 mg, 0.100 mmol, 1.00 equiv) following general procedure **H**. HPLC was done with Method 1.

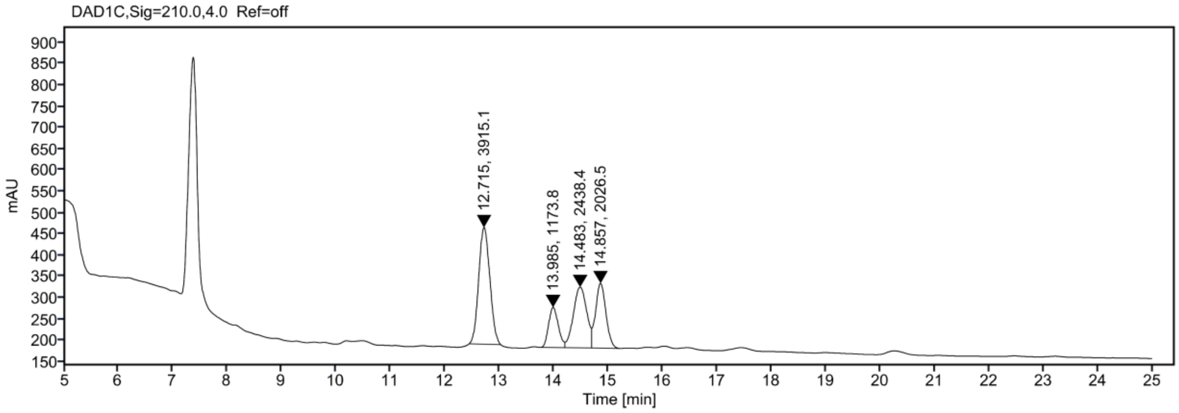

Figure S4. HPLC trace of crude Boc-(N_3)Pro-Val-Leu-Phe-Gly-OMe **31**.

30% remaining starting material (peak at 12.7 min (LRMS: 646.7)). Desired product peaks at 14.5 and 14.7 min: 18% + 15% (2 diastereoisomers) (LRMS: 687.6). A peak corresponding to the product -HN_3 was also observed: LRMS (644.6) at 14.0 min: 8%. As such elimination product has only been observed after HPLC analysis, it seems that the formic acid of the mobile phase triggered such transformation. Thus, to estimate the yield of the azidation reaction, we took into consideration the peaks of the desired product (both dia) and the one of the elimination product.

Overall estimated yield by HPLC-MS: 41%.

This result was confirmed by a ^{1}H NMR estimation of the yield using mesitylene as internal standard: 45%.

HRMS (nanochip-ESI/LTQ-Orbitrap) m/z: [M + H]$^+$ Calcd for $C_{33}H_{51}N_8O_8^+$ 687.3824; Found 687.3796.

MS/MS characterization

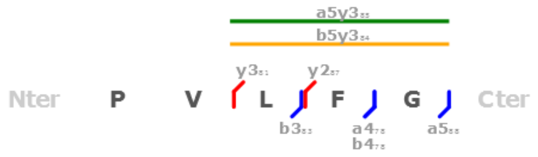

Nter = C5H8O2

Sequence	Type	MF	MF Mass	m/z	ppm	Intensity	Similarity	Qty
**PVL**	b4	C21H35N6O5(+1)	451.2669	451.2663	- 3.68677	16.17835	0.776736	0.025031
LFG	y3	C18H28N3O4(+1)	350.208	350.2074	-3.15722	6.068054	0.806669	0.009066
LF	b5y3	C15H21N2O2(+1)	261.1603	261.1598	-2.5072	0.792967	0.839733	0.001218
LF	a5y3	C14H21N2O(+1)	233.1654	233.1648	-1.90335	0.542679	0.850492	0.000794
FG	y2	C12H17N2O3(+1)	237.1239	237.1234	-2.06492	0.50054	0.865449	0.000765
**PV**	b3	C15H24N5O4(+1)	338.1828	338.1823	-2.99399	0.324931	0.827015	0.000458
PVLFG	y5	C28H44N5O6(+1)	546.3292	546.3286	-3.78924	0.236863	0.866849	0.00059
**PVL**	a4	C20H35N6O4(+1)	423.272	423.2714	-3.28063	0.051661	0.78094	7.63E-05
**PVLF**	a5	C29H44N7O5(+1)	570.3404	570.3398	-4.21743	0.038756	0.876989	8.12E-05

In bold: fragments containing the azide group.

Boc-(N$_3$)Pro-Val-Pro-Val-Pro-Val-OMe (32)

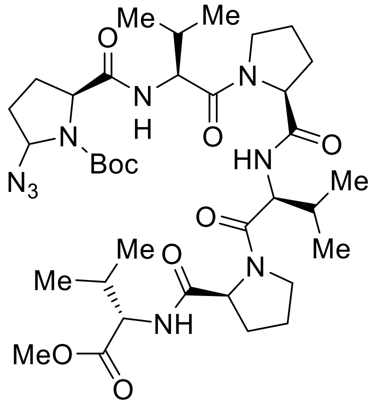

Synthesized from Boc-Pro-Val-Pro-Val-Pro-Val-OMe **60** (72.1 mg, 0.100 mmol, 1.00 equiv) following general procedure **H**. HPLC was done with Method 2.

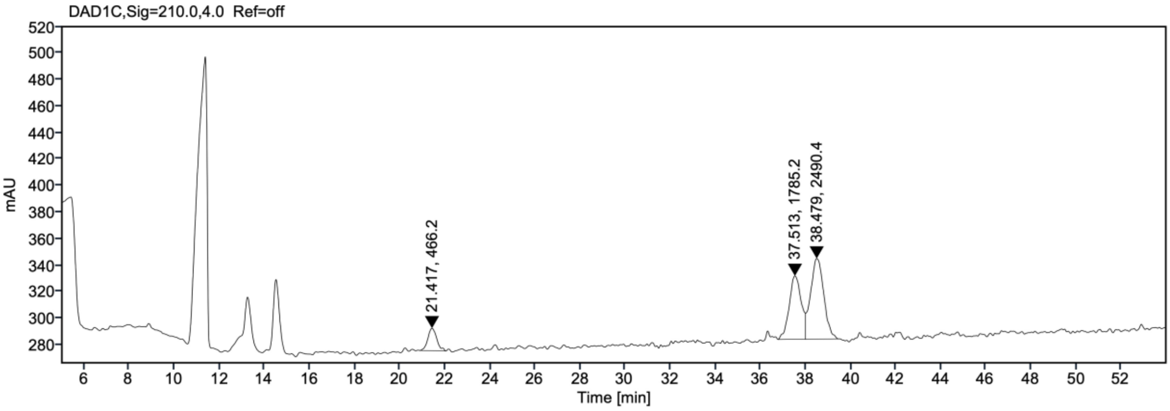

Figure S5. HPLC trace of crude Boc-(N_3)Pro-Val-Pro-Val-Pro-Val-OMe **32**.

2% remaining starting material (peak at 21.4 min (LRMS: 721.5)). Desired product peak at 38.5 min: 15% (LRMS: 762.7). A peak corresponding to the product -HN_3 was also observed: LRMS (719.6) at 37.5 min: 11%. As such elimination product has only been observed after HPLC analysis, it seems that the formic acid of the mobile phase triggered such transformation. Thus, to estimate the yield of the azidation reaction, we took into consideration the peak of the desired product and the one of the elimination product.
Overall estimated yield: 26%.

HRMS (nanochip-ESI/LTQ-Orbitrap) m/z: [M + H]+ Calcd for $C_{36}H_{60}N_9O_9^+$ 762.4509; Found 762.4498.

MS/MS characterization

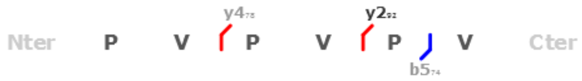

Nter = C5H8O2

Sequence	Type	MF	MF Mass	m/z	ppm	Intensity	Similarity	Qty
PV	y2	C11H21N2O3(+1)	229.1552	229.1547	-1.96693	2.351122	0.918643	0.004675
PVPV	y4	C21H37N4O5(+1)	425.2764	425.2758	-3.91177	0.360397	0.776608	0.000557
**PVPV**	b5	C25H40N7O6(+1)	534.304	534.3035	-4.46695	1.008002	0.735547	0.001548

In bold: fragments containing the azide group.

Boc-(N_3)Pro-Val-(*t*Bu)Glu-Gly-(*t*Bu)Ser-Phe-OMe (33)

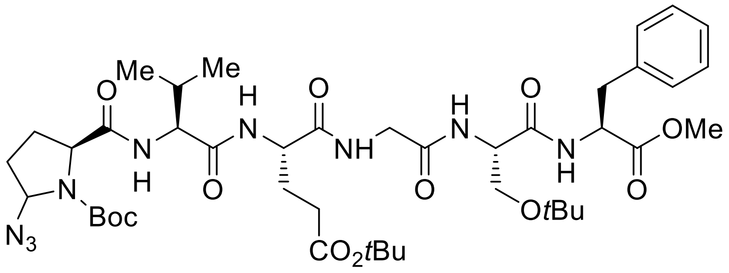

Synthesized from Boc-Pro-Val-(*t*Bu)Glu-Gly-(*t*Bu)Ser-Phe-OMe **61** (86.1 mg, 0.100 mmol, 1.00 equiv) following general procedure **H**. HPLC was done with Method 3 as mobile phase.

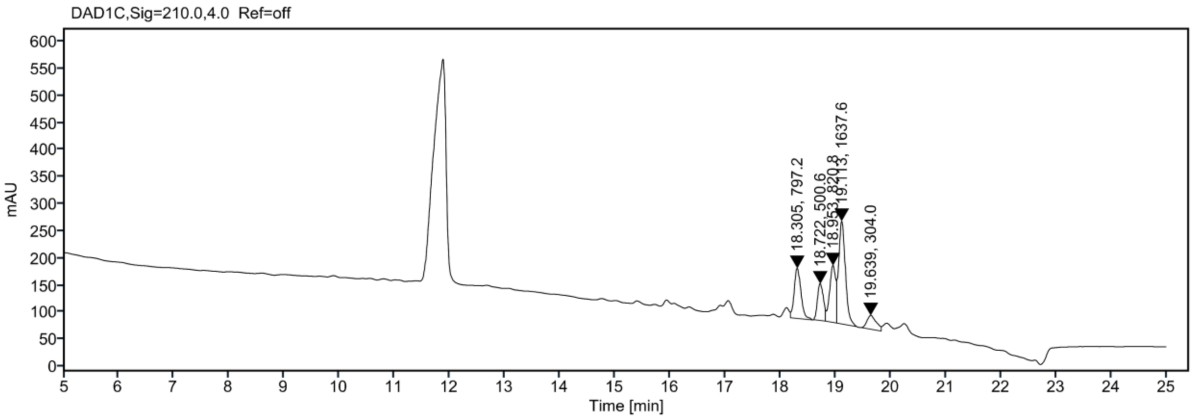

Figure S6. HPLC trace of crude Boc-(N_3)Pro-Val-(*t*Bu)Glu-Gly-(*t*Bu)Ser-Phe-OMe **33**.

5% remaining starting material (peak at 18.3 min (LRMS: 861.5)). Desired product peaks at 19.0, 19.1 and 19.6 min: 6% + 12% + 2% (2 diastereoisomers) (LRMS: 902.7). A peak corresponding to the product – HN_3 was also observed: LRMS (859.6) at 18.7 min: 3%. As such elimination product has only been observed after HPLC analysis, it seems that the formic acid of the mobile phase triggered such transformation. Thus, to estimate the yield of the azidation reaction, we took into consideration the peaks of the desired product (both dia) and the one of the elimination product.
Overall estimated yield: 23%.

HRMS (nanochip-ESI/LTQ-Orbitrap) m/z: $[M + H]^+$ Calcd for $C_{43}H_{68}N_9O_{12}^+$ 902.4982; Found 902.4960.

MS/MS characterization

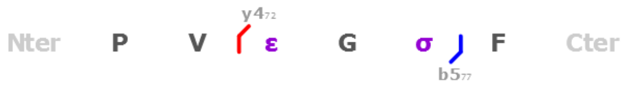

ε = Glu(C4H8)
σ = Ser(C4H8)
Nter = C5H8O2

Sequence	Type	MF	MF Mass	m/z	ppm	Intensity	Similarity	Qty
PVEGSF	y6	C38H61N6O10(+1)	761.4449	761.4444	-2.74301	1.616357	0.773637	0.006577
EGSF	y4	C28H45N4O8(+1)	565.3237	565.3232	-3.45243	0.603292	0.717626	0.000978
**PVEGS**	b5	C26H42N7O8(+1)	580.3095	580.3089	-3.01946	0.248761	0.771826	0.000418

In bold: fragments containing the azide group.

5. Post-functionalizations

5.1 Huisgen [3+2]-cycloadditions

General procedure H

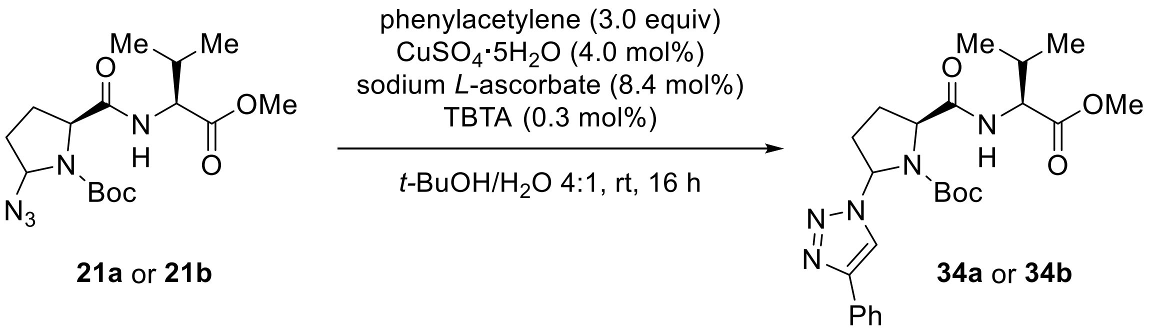

Following a modified literature procedure,[19] in an oven-dried 5 mL glass microwave vial equipped with a magnetic stirring bar was weighed **21** (dia min **21a** or dia maj **21b**) (50.0 mg, 0.135 mmol, 1.00 equiv). The flask was then flushed with nitrogen after which a mixture 4:1 *tert*-butanol/water (1.40 mL) was added followed by phenylacetylene (45.0 μL, 0.410 mmol, 3.00 equiv), copper(II)sulfate pentahydrate (1.40 mg, 0.00500 mmol, 4.00 mol%), sodium ascorbate (2.30 mg, 0.110 mmol, 8.40 mol%) and tris(benzyltriazolylmethyl)amine (0.220 mg, 0.000400 mmol, 0.30 mol%), and the reaction was stirred 16 hours at room temperature under a nitrogen atmosphere. The reaction mixture was then quenched with water and extracted twice with ethyl acetate. The combined organic layers were washed with brine, dried over anhydrous magnesium sulfate, filtered and concentrated under reduced pressure. The crude product was then purified by flash chromatography on silica gel.

***tert*-Butyl (2*S*,5*R*)-2-(((*S*)-1-methoxy-3-methyl-1-oxobutan-2-yl)carbamoyl)-5-(4-phenyl-1*H*-1,2,3-triazol-1-yl)pyrrolidine-1-carboxylate (34a)**

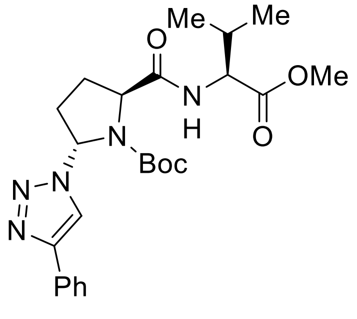

Synthesized from *tert*-butyl (5*S*)-2-azido-5-(((*S*)-1-methoxy-3-methyl-1-oxobutan-2-yl)carbamoyl)pyrrolidine-1-carboxylate dia min **21a** (50.0 mg, 0.135 mmol, 1.0 equiv) following general procedure **H**. *tert*-butyl (2*S*,5*R*)-2-(((*S*)-1-methoxy-3-methyl-1-oxobutan-2-yl)carbamoyl)-5-(4-phenyl-1*H*-1,2,3-triazol-1-yl)pyrrolidine-1-carboxylate **34a** (61.8 mg,

[19] H. Erhardt, F. Mohr, S. F. Kirsch, *Chem. Commun.* **2016**, *52*, 545–548.

0.131 mmol, 97%) was obtained as a yellow solid paste after purification by column chromatography on silica using a gradient from dichloromethane to dichloromethane/methanol 98:2 as eluent.

Rf (dichloromethane/methanol 98:2): 0.23. **Mp**: 54.3–65.3 °C. **^{1}H NMR** (400 MHz, MeOD-d_4, 278.2 K, mixture of rotamers) δ 8.47 (s, 0.44H, C$H_{triazolerotamermin}$), 8.44 (s, 0.58H, C$H_{triazolerotamermaj}$), 7.83 – 7.82 (m, 2H, Ar*H*), 7.51 – 7.40 (m, 2H, Ar*H*), 7.41 – 7.32 (m, 1H, Ar*H*), 6.50 (d, *J* = 7.2 Hz, 0.57H, NC$H_{rotamermaj}$$CH_2CH_2$CHC(O)), 6.45 (d, *J* = 6.9 Hz, 0.43H, NC$H_{rotamermin}$$CH_2CH_2$CHC(O)), 4.76 (d, *J* = 8.9 Hz, 0.43H, NCHCH_2CH_2C$H_{rotamermin}$C(O)), 4.70 (d, *J* = 9.1 Hz, 0.54H, NCHCH_2CH_2C$H_{rotamermaj}$C(O)), 4.37 (d, *J* = 5.8 Hz, 0.42H, NC$H_{Valrotamermin}$C(O)), 4.31 (d, *J* = 5.8 Hz, 0.48H, NC$H_{Valrotamermin}$C(O)), 2.94 – 3.74 (s, 1.4H, $OCH_{3rotamermin}$), 3.74 (s, 1.6H, $OCH_{3rotamermaj}$) 2.92 – 2.74 (m, 1H, NCHCH_2C*H*HCHC(O)), 2.69 – 2.48 (m, 1H, NCHC*H*HCH_2CHC(O)), 2.33 – 2.10 (m, 3H, C$H_{Val}$$(CH_3)_2$ + NCHCH_2CH*H*CHC(O) + NCHCH*H*CH_2CHC(O)), 1.40 (s, 5H, C$H_{3Bocrotamermaj}$), 1.29 (s, 4H, C$H_{3Bocrotamermin}$), 1.07 – 0.97 (m, 6H, CH_{3Val}). **^{13}C NMR** (101 MHz, MeOD-d_4, 278.2 K, mixture of rotamers) δ 175.2 ($Cq_{rotamermaj}$), 174.8 ($Cq_{rotamermin}$), 173.5 ($Cq_{rotamermin}$), 173.5 $Cq_{rotamermaj}$), 155.1 ($Cq_{rotamermaj}$), 154.4 ($Cq_{rotamermin}$), 148.2 ($Cq_{rotamermin}$), 148.1 ($Cq_{rotamermaj}$), 131.6 ($Cq_{rotamermaj}$), 131.6 ($Cq_{rotamermin}$), 130.1 ($CH_{rotamermin}$), 130.0 ($CH_{rotamermaj}$), 129.5 ($CH_{rotamermin}$), 129.4 ($CH_{rotamermaj}$), 126.7 ($CH_{rotamermaj}$), 126.6 ($CH_{rotamermin}$), 122.0 ($CH_{rotamermaj}$), 121.4 ($CH_{rotamermin}$), 82.8 ($Cq_{rotamermaj}$), 82.8 ($Cq_{rotamermin}$), 74.9 (CH), 62.0 ($CH_{rotamermaj}$), 61.7 ($CH_{rotamermin}$), 59.7 ($CH_{rotamermaj}$), 59.4 ($CH_{rotamermin}$), 52.54 ($CH_{3rotamermaj}$), 52.52 ($CH_{3rotamermin}$), 33.7 ($CH_{2rotamermin}$), 32.2 ($CH_{2rotamermaj}$), 31.8 ($CH_{rotamermin}$), 31.4 ($CH_{rotamermaj}$), 30.3 ($CH_{2rotamermaj}$), 28.8 ($CH_{2rotamermin}$), 28.4 ($CH_{3rotamermaj}$), 28.3 ($CH_{3rotamermin}$), 19.8 ($CH_{3rotamermaj}$), 19.5 ($CH_{3rotamermin}$), 18.7 ($CH_{3rotamermaj}$), 18.5 ($CH_{3rotamermin}$). **IR** (ν_{max}, cm^{-1}) 3350 (w), 2973 (m), 2934 (w), 1741 (s), 1705 (s), 1367 (s), 1160 (s), 767 (m), 696 (m). **HRMS** (ESI/QTOF) m/z: $[M + Na]^+$ Calcd for $C_{24}H_{33}N_5NaO_5^+$ 494.2374; Found 494.2376.

tert-Butyl (2*S*,5*S*)-2-(((*S*)-1-methoxy-3-methyl-1-oxobutan-2-yl)carbamoyl)-5-(4-phenyl-1*H*-1,2,3-triazol-1-yl)pyrrolidine-1-carboxylate (34b)

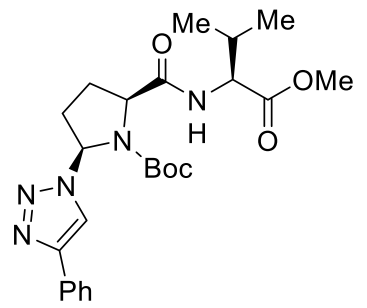

Synthesized from *tert*-butyl (5*S*)-2-azido-5-(((*S*)-1-methoxy-3-methyl-1-oxobutan-2-yl)carbamoyl)pyrrolidine-1-carboxylate dia maj **21b** (50.0 mg, 0.135 mmol, 1.0 equiv) following general procedure **H**. *tert*-butyl (2*S*,5*S*)-2-(((*S*)-1-methoxy-3-methyl-1-oxobutan-2-yl)carbamoyl)-5-(4-phenyl-1*H*-1,2,3-triazol-1-yl)pyrrolidine-1-carboxylate **34b** (61.3 mg, 0.130 mmol, 96%) was obtained as a yellow paste paste after purification by column chromatography on silica using a gradient from dichloromethane to dichloromethane/methanol 96:4 as eluent.

Rf (dichloromethane/methanol 96:4): 0.43. **^{1}H NMR** (400 MHz, MeOD-d_4, 298 K, complex mixture of rotamers) δ 8.93 (s, 0.44H, C$H_{tetrazolerotamermin}$), 8.78 (s, 0.58H, C$H_{tetrazolerotamermaj}$), 7.96 – 7.72 (m, 2H, Ar*H*), 7.44 (t, *J* = 7.6 Hz, 2H, Ar*H*), 7.39 – 7.26 (m, 1H, Ar*H*), 6.48 (app. br s, 1H, NC*H*CH$_2$CH$_2$CHC(O)), 4.56 – 4.20 (m, 2H, NCHCH$_2$CH$_2$C*H*C(O) + NC*H*C(O)), 3.74 – 3.72 (m, 3H, OCH_3), 2.71 – 2.29 (m, 2H, NCHCH_2CH$_2$CHC(O) or/and NCHCH$_2$CH_2CHC(O) and/or CH_{Val}(CH$_3$)$_2$), 2.29 – 2.11 (m, 2H, NCHCH_2CH$_2$CHC(O) or/and NCHCH$_2$CH_2CHC(O) and/or CH_{Val}(CH$_3$)$_2$), 2.09 – 1.83 (m, 1H, NCHCH_2CH$_2$CHC(O) or/and NCHCH$_2$CH_2CHC(O) and/or CH_{Val}(CH$_3$)$_2$), 1.43 (s, 6H, C$H_{3Bocrotamermaj}$), 1.29 (s, 3H, C$H_{3Bocrotamermin}$) 1.05 – 0.92 (m, 6H, CH_{3Val}). **^{13}C NMR** (101 MHz, MeOD-d_4, 278.2 K, mixture of rotamers, signals not fully resolved) δ 175.2 (Cq), 174.8 (Cq), 173.3 (Cq), 173.2 (Cq), 154.9 (Cq), 154.3 (Cq), 148.6 (Cq), 131.69 (Cq$_{rotamermaj}$), 131.67 (Cq$_{rotamermin}$), 130.1 (CH), 130.0 (CH), 129.5 (CH), 129.4 (CH), 126.7 (CH$_{rotamermaj}$), 126.6 (CH$_{rotamermin}$), 121.3 (CH$_{rotamermaj}$), 120.8 (CH$_{rotamermin}$), 83.4 (Cq$_{rotamermaj}$), 82.9 (Cq$_{rotamermin}$), 75.7 (CH$_{rotamermaj}$), 75.6 (CH$_{rotamermin}$), 63.4 (CH), 62.5 (CH), 59.5 (CH$_{rotamermaj}$), 59.3 (CH$_{rotamermin}$), 52.64 (CH$_{3rotamermin}$), 52.56 (CH$_{3rotamermaj}$), 34.6 (CH$_{2rotamermin}$), 33.8 (CH$_{2rotamermin}$), 32.2 (CH$_{rotamermin}$), 32.0 (CH$_{rotamermaj}$), 30.3 (CH$_{2rotamermaj}$), 29.1 (CH$_{2rotamermin}$), 28.3 (CH$_{3rotamermaj}$), 28.3 (CH$_{3rotamermin}$), 19.7 (CH$_{3rotamermaj}$), 19.6 (CH$_{3rotamermin}$), 18.8 (CH$_{3rotamermaj}$), 18.5 (CH$_{3rotamermin}$). **IR** (ν_{max}, cm^{-1}) 2960 (m), 2891 (w), 1742 (s), 1710 (s), 1677 (s), 1366 (s), 1156 (s), 768 (s), 696 (m). **HRMS** (ESI/QTOF) m/z: [M + Na]$^+$ Calcd for $C_{24}H_{33}N_5NaO_5^+$ 494.2374; Found 494.2378.

5.2 Nucleophilic susbtitutions

Methyl ((2*S*)-5-(2-methoxy-2-oxoethyl)pyrrolidine-2-carbonyl)-*L*-valinate (35)

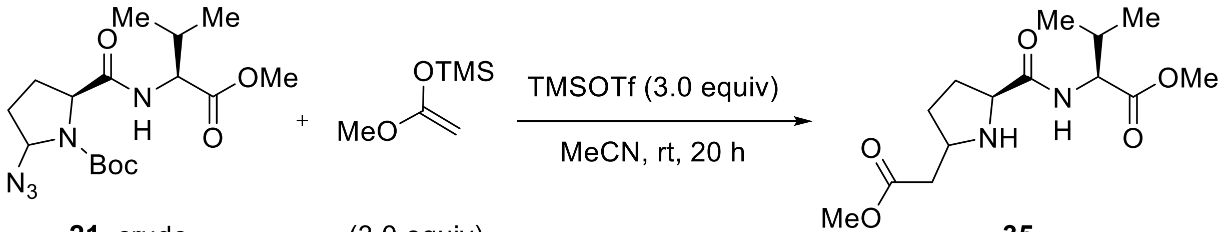

21, crude (3.0 equiv) **35**

In a 25 mL round-bottom flask equipped with a magnetic stirring bar, crude *tert*-butyl (5*S*)-2-azido-5-(((*S*)-1-methoxy-3-methyl-1-oxobutan-2-yl)carbamoyl)pyrrolidine-1-carboxylate **21** (0.40 mmol, 1.0 equiv) was dissolved in 4.0 mL of anhydrous acetonitrile. 1-(*tert*-butyldimethylsilyloxy)-1-methoxyethene (0.26 mL, 1.2 mmol, 3.0 equiv) and trimethylsilyl trifluoromethanesulfonate (0.22 mL, 1.2 mmol, 3.0 equiv) were added and the reaction was stirred at room temperature overnight under a nitrogen atmosphere. The reaction mixture was then quenched with water and extracted twice with ethyl acetate. The combined organic layers were drier over anhydrous magnesium sulfate, filtered and concentrated under reduced pressure. The crude product was purified by flash chromatography on silica gel using a gradient dichloromethane to dichloromethane/methanol 96:4 as eluent to afford methyl ((2*S*)-5-(2-methoxy-2-oxoethyl)pyrrolidine-2-carbonyl)-*L*-valinate **35** as an orange oil (0.71 g, 0.24 mmol, 59%) (mixture of diastereoisomers, n.d. dr).

Rf (dichloromethane/methanol 96:4): 0.30. **^{1}H NMR** (400 MHz, MeOD-*d4*, mixture of two diastereoisomers)) δ 4.35 (m, 1H, NHCH_{Val}C(O)), 3.86 (dd, *J* = 8.6, 6.7 Hz, 0.6H, NCHCH$_2$CH$_2$CH_{Pro}C(O)), 3.78 (dd, *J* = 9.2, 4.5 Hz, 0.4H, N NCHCH$_2$CH$_2$CH_{Pro}C(O)), 3.73 – 3.73 (m, 3H, CO$_2$CH_{3Val}), 3.70 – 3.69 (m, 3H, CH$_2$CO$_2$CH_3), 3.64 (m, 0.4H, NCH_{Pro}CH$_2$CH$_2$CHC(O)), 3.57 (m, 0.6H, NCH_{Pro}CH$_2$CH$_2$CHC(O)), 2.63 – 2.43 (m, 2H, CH_2CO$_2$CH$_3$), 2.31 (dtd, *J* = 12.8, 8.4, 4.4 Hz, 0.6H, NCHCH$_2$CHH_{Pro}CHC(O)), 2.26 – 2.12 (m, 1.4H, CH_{Val}(CH$_3$)$_2$ + NCHCH$_2$CHH_{Pro}CHC(O)), 2.08 – 1.88 (m, 1.4H, NCHCH_{Pro}HCH$_2$CHC(O) + NCHCH$_2$CH_{Pro}HCHC(O)), 1.81 (m, 0.6H, NCHCH$_2$CH_{Pro}HCHC(O)), 1.48 (m, 1H, NCHCHH_{Pro}CH$_2$CHC(O)), 0.95 (dd, *J* = 6.8, 4.6 Hz, 6H, CH_{3Val}). **^{13}C NMR** (101 MHz, MeOD-*d4*, mixture of two diastereoisomers) δ 177.7 (Cq$_{diamin}$), 176.6 (Cq$_{diamaj}$), 174.1 (Cq$_{diamin}$), 173.8 (Cq$_{diamaj}$), 173.39 (Cq$_{diamaj}$), 173.35 (Cq$_{diamin}$), 61.6 (CH$_{diamin}$), 61.1 (CH$_{diamaj}$), 58.8 (CH$_{diamaj}$), 58.5 (CH$_{diamin}$), 57.2 (CH$_{diamaj}$), 57.1 (CH$_{diamin}$), 52.6 (CH$_{3diamin}$), 52.6 (CH$_{3diamaj}$), 52.2 (CH$_{3diamaj}$), 52.1 (CH$_{3diamin}$), 42.0 (CH$_{2diamin}$), 40.0 (CH$_{2diamaj}$), 33.0 (CH$_{2diamaj}$), 32.2 (CH$_{diamin}$), 32.00 (CH$_{2diamin}$), 31.95 (CH$_{diamaj}$), 31.6 (CH$_{2diamin}$), 31.4 (CH$_{2diamaj}$), 19.52 (CH$_{3diamin}$), 19.48 (CH$_{3diamaj}$), 18.2 (CH$_{3diamaj}$), 18.1 (CH$_{3diamin}$). **IR** (ν_{max}, cm^{-1}) 3316 (w), 2961 (m), 1737 (s), 1660 (s), 1514 (m), 1437 (m), 1272 (m), 1205 (m), 1151 (m). **HRMS** (ESI/QTOF) m/z: [M + H]$^+$ Calcd for $C_{14}H_{25}N_2O_5^+$ 301.1758; Found 301.1762.

Methyl ((2*S*)-5-(2-oxo-2-phenylethyl)pyrrolidine-2-carbonyl)-*L*-valinate (36)

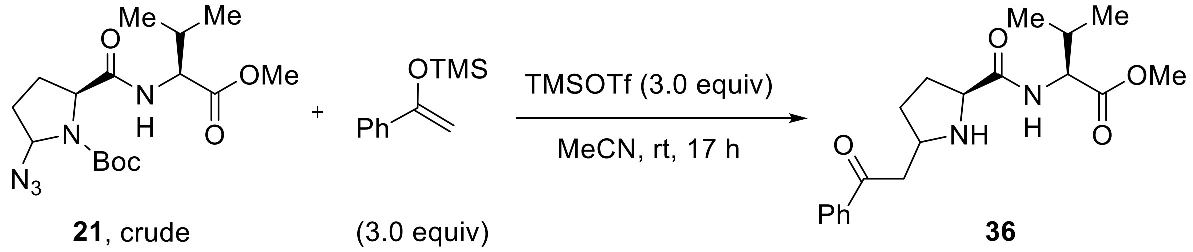

21, crude (3.0 equiv) **36**

In a 25 mL round-bottom flask equipped with a magnetic stir bar, crude *tert*-butyl (5*S*)-2-azido-5-(((*S*)-1-methoxy-3-methyl-1-oxobutan-2-yl)carbamoyl)pyrrolidine-1-carboxylate **21** (0.40 mmol, 1.0 equiv) was dissolved in 4.0 mL of anhydrous acetonitrile. 1-phenyl-1-trimethylsiloxyethylene (0.25 mL, 1.2 mmol, 3.0 equiv) and trimethylsilyl trifluoromethanesulfonate (0.22 mL, 1.2 mmol, 3.0 equiv) were added and the reaction was stirred at room temperature overnight under a nitrogen atmosphere. The reaction mixture was then quenched with water and extracted twice with ethyl acetate. The combined organic layers were drier over anhydrous magnesium sulfate, filtered and concentrated under reduced pressure. The crude product was purified by flash chromatography on silica gel using a gradient dichloromethane to dichloromethane/methanol 96:4 as eluent to afford methyl ((2*S*)-5-(2-oxo-2-phenylethyl)pyrrolidine-2-carbonyl)-*L*-valinate **36** as an orange paste (0.96 g, 0.28 mmol, 69%) (mixture of diastereoisomers, n.d. dr).

Rf (dichloromethane/methanol 96:4): 0.38.**^{1}H NMR** (400 MHz, MeOD-*d4*, complex mixture of diastereoisomers and rotamers) δ 8.04 (d, *J* = 8.5 Hz, 2H, Ar*H*), 7.70 – 7.60 (m, 1H, Ar*H*), 7.53 (t, *J* = 7.6 Hz, 2H, Ar*H*), 4.37 (dd, *J* = 15.2, 5.6 Hz, 1H, NHCH_{Val}C(O)), 4.27 (m, 1H, NCHCH$_2$CH$_2$CH_{Pro}C(O)), 4.06 (m, 1H, NCH_{Pro}CH$_2$CH$_2$CHC(O)), 3.74 – 3.72 (m, 3H, CO$_2$CH_3), 3.65

– 3.39 (m, 2H, CH_2CO$_2$Ph), 2.53 (m, 0.4H, NCHC*H*HCH$_2$CHC(O)), 2.46 – 2.12 (m, 3H, C*H*$_{Val}$(CH$_3$)$_2$ + NCHCH$_2$CH_{2Pro}CHC(O)), 2.03 (m, 0.6H, NCHC*H*HCH$_2$CHC(O)), 1.78 (m, 1H, NCHCH*H*CH$_2$CHC(O)), 0.99 – 0.94 (m, 6H, CH_{3Val}). **^{13}C NMR** (101 MHz, MeOD-*d$_4$*, 298 K, complex mixture of diastereoisomers and rotamers, signals not fully resolved) δ 199.6 (Cq), 199.3 (Cq), 173.2 (Cq), 173.2 (Cq), 172.7 (Cq), 171.8 (Cq), 137.6 (Cq), 137.5 (Cq), 134.9 (CH), 134.8 (CH), 129.89 (CH), 129.85 (CH), 129.3 (CH), 123.4 (CH), 120.2 (CH), 61.4 (CH), 60.9 (CH), 59.5 (CH), 59.4 (CH), 57.9 (CH), 57.8 (CH), 52.6 (CH$_3$), 43.1 (CH$_2$), 42.3 (CH$_2$), 31.7 (CH), 31.6 (CH), 31.0 (CH$_2$), 30.73 (CH$_2$), 30.68 (CH$_2$), 30.5 (CH$_2$), 30.4 (CH$_2$), 19.43 (CH$_3$), 19.42 (CH$_3$), 18.5 (CH$_3$), 18.34 (CH$_3$), 18.28 (CH$_3$). **IR** (ν_{max}, cm^{-1}) 3335 (w), 2962 (w), 1741 (m), 1681 (m), 1276 (m), 1248 (s), 1223 (s), 1156 (m). **HRMS** (ESI/QTOF) m/z: [M + H]$^+$ Calcd for $C_{19}H_{27}N_2O_4^+$ 347.1965; Found 347.1966.

Methyl ((2*S*)-5-allylpyrrolidine-2-carbonyl)-*L*-valinate (37)

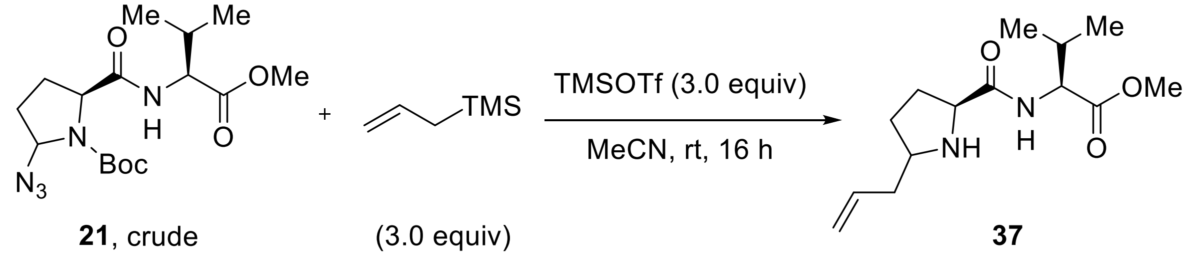

21, crude (3.0 equiv) **37**

In a 25 mL round-bottom flask equipped with a magnetic stir bar, crude *tert*-butyl (5*S*)-2-azido-5-(((*S*)-1-methoxy-3-methyl-1-oxobutan-2-yl)carbamoyl)pyrrolidine-1-carboxylate **21** (0.40 mmol, 1.0 equiv) was dissolved in 4.0 mL of anhydrous acetonitrile. Allyltrimethylsilane (0.20 mL, 1.2 mmol, 3.0 equiv) and trimethylsilyl trifluoromethanesulfonate (0.22 mL, 1.2 mmol, 3.0 equiv) were added and the reaction was stirred at room temperature overnight under a nitrogen atmosphere. The reaction mixture was then quenched with water and extracted twice with ethyl acetate. The combined organic layers were drier over anhydrous magnesium sulfate, filtered and concentrated under reduced pressure. The crude product was purified by flash chromatography on silica gel using a gradient dichloromethane to dichloromethane/methanol 96:4 as eluent to afford methyl ((2*S*)-5-allylpyrrolidine-2-carbonyl)-*L*-valinate **37** as a brown oil (0.71 g, 0.26 mmol, 66%) (mixture of diastereoisomers, 2:1 dr determined by integration of the ^{1}H NMR peaks at 3.95 and 3.81 ppm).

Rf (dichloromethane/methanol 96:4): = 0.40. **^{1}H NMR** (400 MHz, MeOD-*d$_4$*, 298 K, mixture of two diastereoisomers) δ 5.87 (m, 1H, CH$_2$=C*H*$_{allyl}$), 5.29 – 4.96 (m, 2H, CH=CH_{2allyl}), 4.32 (m, 1H, NHC*H*$_{Val}$C(O)), 3.95 (dd, *J* = 8.5, 6.9 Hz, 0.33H, NCHCH$_2$CH$_2$C*H*C(O)), 3.81 (dd, *J* = 9.7, 4.4 Hz, 0.66H, NCHCH$_2$CH$_2$C*H*C(O)), 3.73 (2 x s, 3H, OCH_3), 3.37 (m, 1H, NC*H*CH$_2$CH$_2$CHC(O)), 2.41 – 2.12 (m, 4H, CH$_2$=CHCH_{2allyl}, C*H*$_{Val}$(CH$_3$)$_2$ + NHCHCH$_2$C*H*HCHC(O)), 2.05 – 1.79 (m, 2H, NHCHCH*H*CH$_2$CHC(O) + NHCHCH$_2$C*H*HCHC(O)), 1.54 (m, 0.33H, NHCHCH*H*CH$_2$CHC(O)), 1.40 (m, 0.66H, NHCHCH*H*CH$_2$CHC(O)), 0.96 – 0.93 (m, 6H, CH$_{3Val}$). **^{13}C NMR** (101 MHz, MeOD-*d$_4$*, 298 K, mixture of two diastereoisomers, signals not fully resolved) δ 176.9 (Cq$_{diamaj}$), 175.5 (Cq$_{diamin}$), 173.3 (Cq), 136.6 (CH$_{diamaj}$), 136.01 (CH$_{diarmin}$), 117.9 (CH$_{2diamin}$), 117.5 (CH$_{2diamaj}$), 61.3

(CH$_{diamaj}$), 61.1 (CH$_{diamin}$), 60.8 (CH$_{diamin}$), 60.5 (CH$_{diamaj}$), 58.9 (CH$_{diamin}$), 58.7 (CH$_{diamaj}$), 52.60 (CH$_{3diamaj}$), 52.57 (CH$_{3diamin}$), 41.3 (CH$_{2diamaj}$), 39.9 (CH$_{2diamin}$), 32.4 (CH$_{2diamin}$), 32.1 (CH$_{diamaj}$), 31.9 (CH$_{diamin}$), 31.7 (CH$_{diamaj}$), 31.6 (CH$_{diamaj}$), 31.3 (CH$_{2diamin}$), 19.51 (CH$_{3diamaj}$), 19.47 (CH$_{3diamin}$), 18.2 (CH$_{3diamin}$), 18.1 (CH$_{3diamaj}$). **IR** (ν_{max}, cm^{-1}) 2960 (m), 1740 (s), 1655 (s), 1510 (s), 1209 (m), 1151 (s), 1031 (s), 997 (m), 912 (m). **HRMS** (ESI/QTOF) m/z: [M + H]$^+$ Calcd for $C_{14}H_{25}N_2O_3^+$ 269.1860; Found 269.1859.

Methyl ((2*S*)-5-(2-hydroxynaphthalen-1-yl)pyrrolidine-2-carbonyl)-*L*-valinate (38)

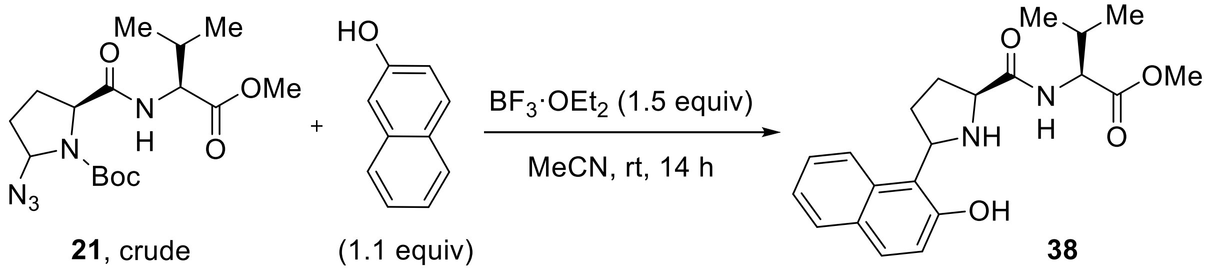

21, crude (1.1 equiv) **38**

In a 25 mL round-bottom flask equipped with a magnetic stirring bar, crude *tert*-butyl (5*S*)-2-azido-5-(((*S*)-1-methoxy-3-methyl-1-oxobutan-2-yl)carbamoyl)pyrrolidine-1-carboxylate **21** (0.40 mmol, 1.0 equiv) was dissolved in 4.0 mL of anhydrous acetonitrile. 2-naphtol (0.87 g, 0.6 mmol, 1.5 equiv) and boron trifluoride diethyl etherate (0.12 mL, 0.44 mmol, 1.1 equiv) were added and the reaction was stirred at room temperature overnight under a nitrogen atmosphere. The reaction mixture was then quenched with water and extracted twice with ethyl acetate. The combined organic layers were drier over anhydrous magnesium sulfate, filtered and concentrated under reduced pressure. The crude product was purified by flash chromatography on deactivated silica gel using a gradient dichloromethane to dichloromethane/methanol 96:4 as eluent to afford methyl ((2*S*)-5-(2-hydroxynaphthalen-1-yl)pyrrolidine-2-carbonyl)-*L*-valinate **38** as a brown solid (0.86 g, 0.23 mmol, 58%) (mixture of diastereoisomers, 1.2:1 dr, determined by integration of the ^{1}H NMR peaks at 5.35 and 5.24 ppm).

Rf (dichloromethane/methanol 96:4): = 0.28, 0.24 (2 dia). **^{1}H NMR** (400 MHz, MeOD-*d$_4$*, 298 K, mixture of two diastereoisomers) δ 7.83 (dd, *J* = 19.8, 8.4 Hz, 1H, Ar*H*), 7.75 – 7.66 (m, 1H, Ar*H*), 7.61 (dd, *J* = 8.7, 2.6 Hz, 1H, Ar*H*), 7.40 (ddt, *J* = 8.5, 6.8, 1.5 Hz, 1H, Ar*H*), 7.24 (ddt, *J* = 7.9, 6.8, 1.0 Hz, 1H, Ar*H*), 6.97 (dd, *J* = 10.9, 8.9 Hz, 1H, Ar*H*), 5.35 (dd, *J* = 9.8, 6.5 Hz, 0.55H, NC*H*CH$_2$CH$_2$CHC(O)), 5.24 (m, 0.45H, NC*H*CH$_2$CH$_2$CHC(O)), 4.40 (dd, *J* = 8.9, 6.0 Hz, 1H, NHC*H*$_{Val}$C(O)), 4.13 (m, 1H, NCHCH$_2$CH$_2$C*H*C(O)), 3.75 (s, 1.5H, OC*H$_3$*), 3.73 (s, 1.5H, OC*H$_3$*), 2.58 – 2.31 (m, 2H, NHCHCH$_2$C*H*HCHC(O) + NHCHCH*H*CH$_2$CHC(O)), 2.24 – 1.97 (m, 2H, C*H*$_{Val}$(CH$_3$)$_2$ + NHCHCH$_2$CH*H*CHC(O)), 1.82 (m, 1H, NHCHC*H*HCH$_2$CHC(O)), 1.01 –0.99 (m, 6H, C*H$_3$*$_{Val}$). **^{13}C NMR** (101 MHz, MeOD-*d$_4$*, 298 K, mixture of two diastereoisomers, signals not fully resolved) δ 176.6 (Cq$_{diamin}$), 176.1 (Cq$_{diamaj}$), 173.7 (Cq$_{diamin}$), 173.5 (Cq$_{diamaj}$), 157.0 (Cq$_{diamaj}$), 156.7 (Cq$_{diamin}$), 133.9 (Cq$_{diamin}$), 133.7 (Cq$_{diamaj}$), 129.9 (Cq), 129.83 (CH$_{diamin}$), 129.80 (Cq$_{diamaj}$), 129.66 (CH$_{diamin}$), 129.65 (CH$_{diamaj}$), 127.37 (CH$_{diamaj}$), 127.35 (CH$_{diamin}$), 123.36 (CH$_{diamaj}$), 123.3 (CH$_{diamin}$), 122.5 (CH$_{diamaj}$), 122.3 (CH$_{diamin}$), 120.8 (CH$_{diamaj}$), 120.7 (CH$_{diamin}$), 116.7 (Cq$_{diamin}$), 116.6 (Cq$_{diamaj}$), 60.7 (CH$_{diamaj}$), 60.5 (CH$_{diamin}$), 60.4 (CH$_{diamaj}$), 60.1 (CH$_{diamin}$), 59.6 (CH$_{diamaj}$)

59.3 (CH_{diamin}), 52.5 (CH_3), 34.6 ($CH_{2diamaj}$), 32.8 ($CH_{2diamin}$), 32.4 (CH_{diamaj}), 31.8 (CH_{diamaj}), 31.6(CH_{diamin}), 31.1 ($CH_{2diamin}$), 19.52 ($CH_{3diamin}$), 19.47 ($CH_{3diamaj}$), 18.6 ($CH_{3diamaj}$), 18.5 ($CH_{3diamin}$). **IR** (ν_{max}, cm^{-1}) 3315 (m), 2970 (s), 1744 (s), 1675 (s), 1661 (s), 1623 (s), 1521 (s), 1471 (s), 1372 (m), 1271 (s), 816 (s). **HRMS** (nanochip-ESI/LTQ-Orbitrap) m/z: $[M + H]^+$ Calcd for $C_{21}H_{27}N_2O_4^+$ 371.1965; Found 371.1962.

Methyl ((2*S*)-5-((E)-styryl)pyrrolidine-2-carbonyl)-*L*-valinate (39)

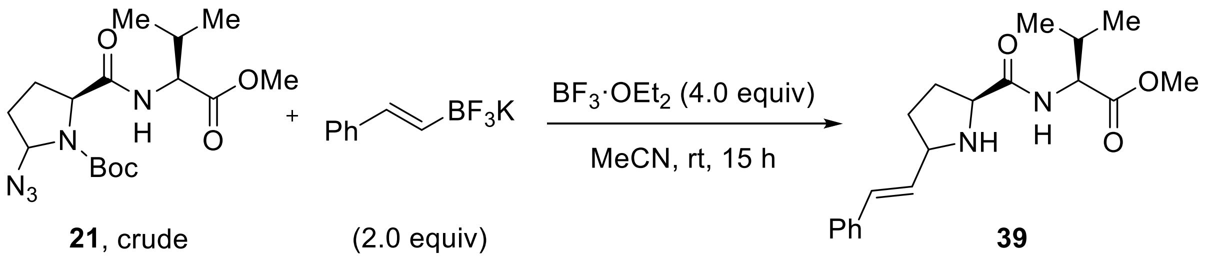

In a 25 mL round-bottom flask equipped with a magnetic stirring bar under a nitrogen atmosphere, crude *tert*-butyl (5*S*)-2-azido-5-(((*S*)-1-methoxy-3-methyl-1-oxobutan-2-yl)carbamoyl)pyrrolidine-1-carboxylate **21** (0.40 mmol, 1.0 equiv) was dissolved in 4.0 mL of anhydrous acetonitrile. Potassium *trans*-styryltrifluoroborate (0.17 g, 0.80 mmol, 2.0 equiv) and boron trifluoride diethyl etherate (0.44 mL, 1.6 mmol, 4.0 equiv) were added and the reaction was stirred at room temperature overnight under a nitrogen atmosphere. The reaction mixture was then quenched with water and extracted twice with ethyl acetate. The combined organic layers were drier over anhydrous magnesium sulfate, filtered and concentrated under reduced pressure. The crude product was purified by flash chromatography on deactivated silica gel using a gradient dichloromethane to dichloromethane/methanol 96:4 as eluent to afford methyl ((2*S*)-5-((E)-styryl)pyrrolidine-2-carbonyl)-*L*-valinate **39** as an orange oil (0.84 g, 0.25 mmol, 63%) (mixture of diastereoisomers, 6.7:1 dr determined by integration of the ^{1}H NMR peaks at 6.66 and 6.55 ppm).

Rf (dichloromethane/methanol 96:4): = 0.31. **^{1}H NMR** (400 MHz, MeOD-*d₄*, 298 K, mixture of two diastereoisomers) δ 7.43 – 7.35 (m, 2H, Ar*H*), 7.34 – 7.25 (m, 2H, Ar*H*), 7.25 – 7.16 (m, 1H, Ar*H*), 6.66 (d, *J* = 15.8 Hz, 0.13H, PhCH_{diamin}=CH), 6.55 (d, *J* = 15.9 Hz, 0.87H, PhCH_{diamaj}=CH), 6.25 + 6.28 (dd, *J* = 15.8, 7.4 Hz, 1H, PhCH=C*H*), 4.39 (d, *J* = 5.1 Hz, 0.14H, NHC$H_{Valdiamin}$C(O)), 4.36 (d, *J* = 5.6 Hz, 0.86H, NHC$H_{Valdiamaj}$C(O)), 3.91 – 3.86 (m, 1.85H, NCHCH_2CH_2C*H*C(O) + NC*H*CH_2CH_2CHC(O)), 3.81 (dd, *J* = 9.5, 3.5 Hz, 0.15H, NCHCH_2CH_2C*H*C(O) and/or NC*H*CH_2CH_2CHC(O)), 3.74 – 3.72 (app. m, 3H, OCH_3), 2.35 (m, 1H, C*H*H_{Pro}), 2.19 (m, 1H, CH_{Val}(CH_3)$_2$), 2.03 (m, 1H, C*H*H_{Pro}), 1.85 (m, 1H, C*H*H_{Pro}), 1.67 (m, 1H, C*H*H_{Pro}), 1.00 – 0.88 (m, 6H, CH_{3Val}). **^{13}C NMR** (101 MHz, MeOD-*d₄*, 298 K, mixture of two diastereoisomers) δ 178.0 (Cq_{diamin}), 177.6 (Cq_{diamaj}), 173.42 (Cq_{diamaj}), 173.4 (Cq_{diamin}), 138.5 (Cq_{diamin}), 138.4 (Cq_{diamaj}), 133.5 (CH_{diamin}), 132.2 (CH_{diamaj}), 131.6 (CH_{diamaj}), 130.9 (CH_{diamin}), 129.6 (CH), 128.5 (CH_{diamaj}), 128.4 (CH_{diamin}), 127.4 (CH_{diamaj}), 127.3 (CH_{diamin}), 62.8 (CH_{diamaj}), 62. (CH_{diamin}), 61.5 (CH_{diamin}), 61.3 (CH_{diamaj}), 58.6 (CH_{diamaj}), 58.5 (CH_{diamin}), 52.7 ($CH_{3diamaj}$), 52.6 ($CH_{3diamin}$), 34.1 ($CH_{2diamaj}$), 33.2 ($CH_{2diamin}$), 32.2 (CH_{diamin}), 32.14 ($CH_{2diamin}$), 32.1 (CH_{diamaj}), 31.7 ($CH_{2diamin}$), 19.5 ($CH_{2diamaj}$),

18.3 ($CH_{2diamaj}$), 18.2 ($CH_{2diamin}$), 18.05 ($CH_{2diamin}$). **IR** (ν_{max}, cm^{-1}) 3332 (m), 2964 (m), 2862 (w), 1739 (s), 1665 (s), 1505 (s), 1435 (m), 1206 (s), 750 (s), 694 (s). **HRMS** (ESI/QTOF) m/z: $[M + H]^+$ Calcd for $C_{19}H_{27}N_2O_3^+$ 331.2016; Found 331.2021.

6. Competitive Huisgen [3+2]-cycloaddition experiment

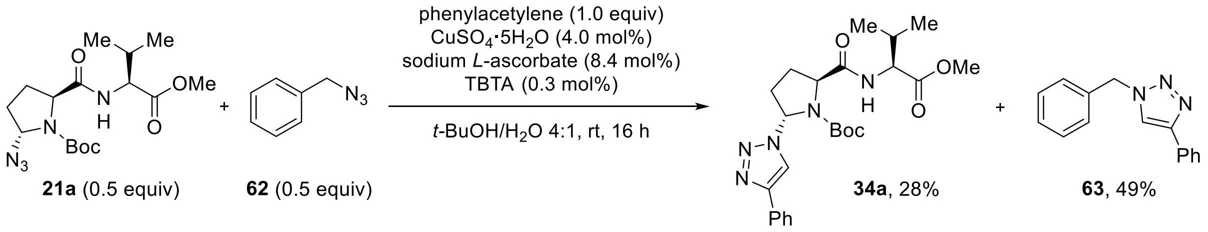

Following a modified literature procedure,[19] in an oven-dried 5 mL glass microwave vial equipped with a magnetic stirring bar was weighed **21a** (21 mg, 0.056 mmol, 0.50 equiv). The flask was flushed with nitrogen after which a mixture 4:1 *tert*-butanol/water (1.3 mL) was added followed by benzyl azide (**62**) (7.5 μL, 0.056 mmol, 0.50 equiv), phenylacetylene (12 μL, 0.11 mmol, 1.0 equiv), copper(II)sulfate pentahydrate (1.1 mg, 0.0045 mmol, 4.0 mol%), sodium ascorbate (1.9 mg, 0.0094 mmol, 8.4 mol%) and tris(benzyltriazolylmethyl)amine (0.18 mg, 0.00034 mmol, 0.30 mol%), and the reaction was stirred 16 hours at room temperature under a nitrogen atmosphere. The reaction mixture was then quenched with water and extracted twice with ethyl acetate. The combined organic layers were washed with brine, dried over anhydrous magnesium sulfate, filtered and concentrated under reduced pressure. Mesitylene (20 μL, 0.14 mmol, 1.3 equiv) was added and a ^1H NMR was taken. *tert*-Butyl (2*S*,5*R*)-2-(((*S*)-1-methoxy-3-methyl-1-oxobutan-2-yl)carbamoyl)-5-(4-phenyl-1*H*-1,2,3-triazol-1-yl)pyrrolidine-1-carboxylate **34a** was observed in a 28% yield determined using the peaks corresponding to the triazole **^{1}H NMR** (400 MHz, MeOD-d_4, 298 K, mixture of rotamers) δ 8.47 (s, 0.12H, $CH_{triazolerotamerminj}$), 8.44 (s, 0.16H, $CH_{triazolerotamermaj}$). 1-Benzyl-4-phenyl-1*H*-1,2,3-triazole **63** was observed in a 49% yield determined using the peaks corresponding to the triazole **^{1}H NMR** (400 MHz, MeOD-d_4, 298 K) 8.35 (s, 0.49H, $CH_{triazole}$).[20] Only characteristic peaks are listed as the crude ^1H NMR was too complex to give the complete ^1H NMR listing.

[20] H.-B. Chen, N. Abeyrathna, Y. Liao, *Tetrahedron Lett.* **2014**, *55*, 6575–6576.

7. NMR spectra

^{1}H NMR (400 MHz, $CDCl_3$, 298 K, mixture of two rotamers) of compound **42**

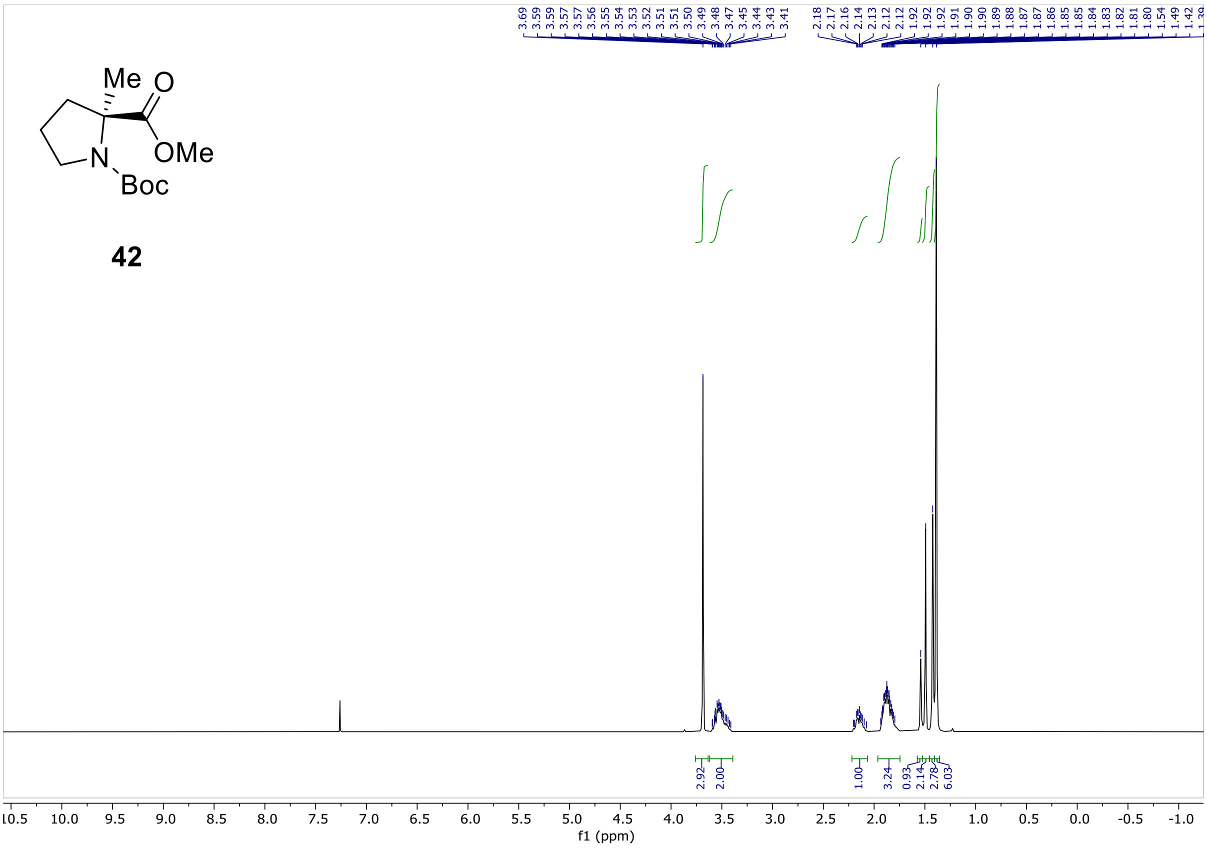

^{13}C NMR (101 MHz, $CDCl_3$, 298 K, mixture of two rotamers) of compound **42**

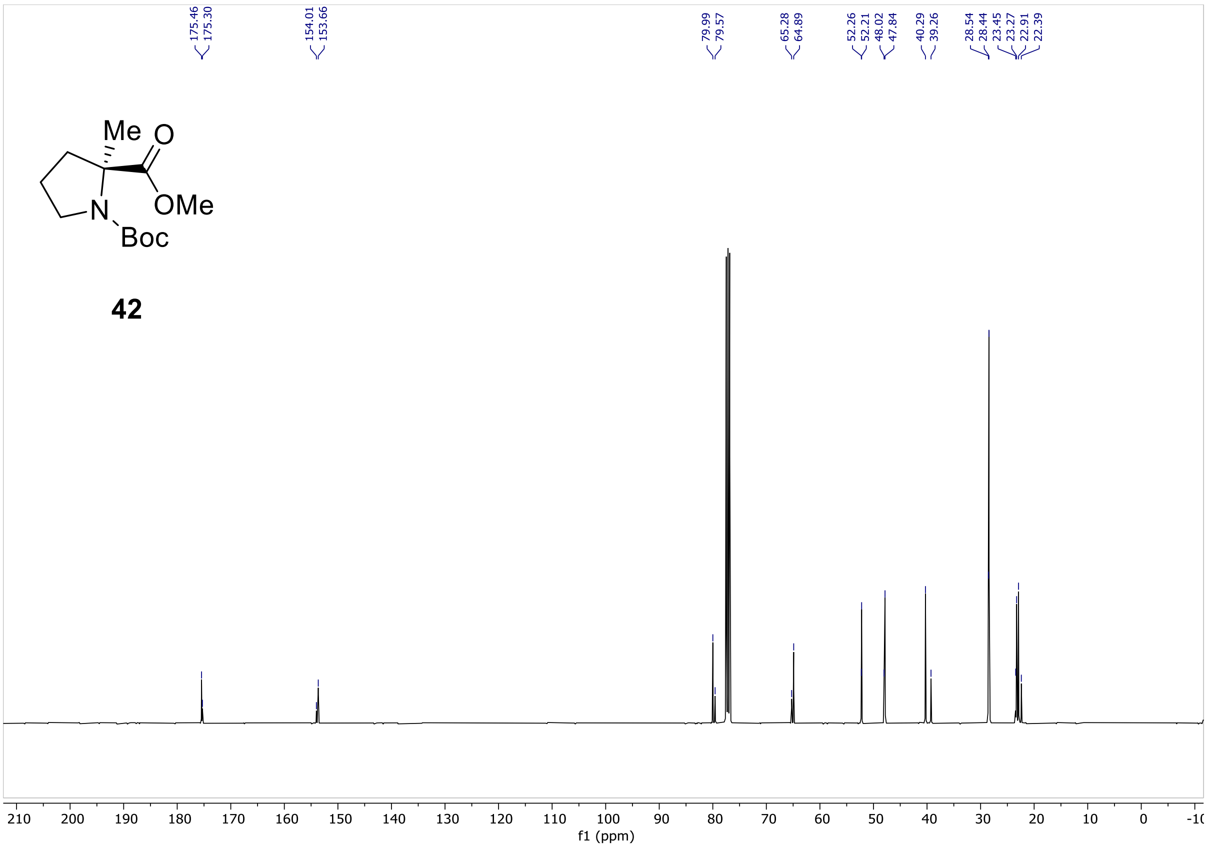

DEPT (101 MHz, $CDCl_3$, 298 K, mixture of two rotamers) of compounds **42**

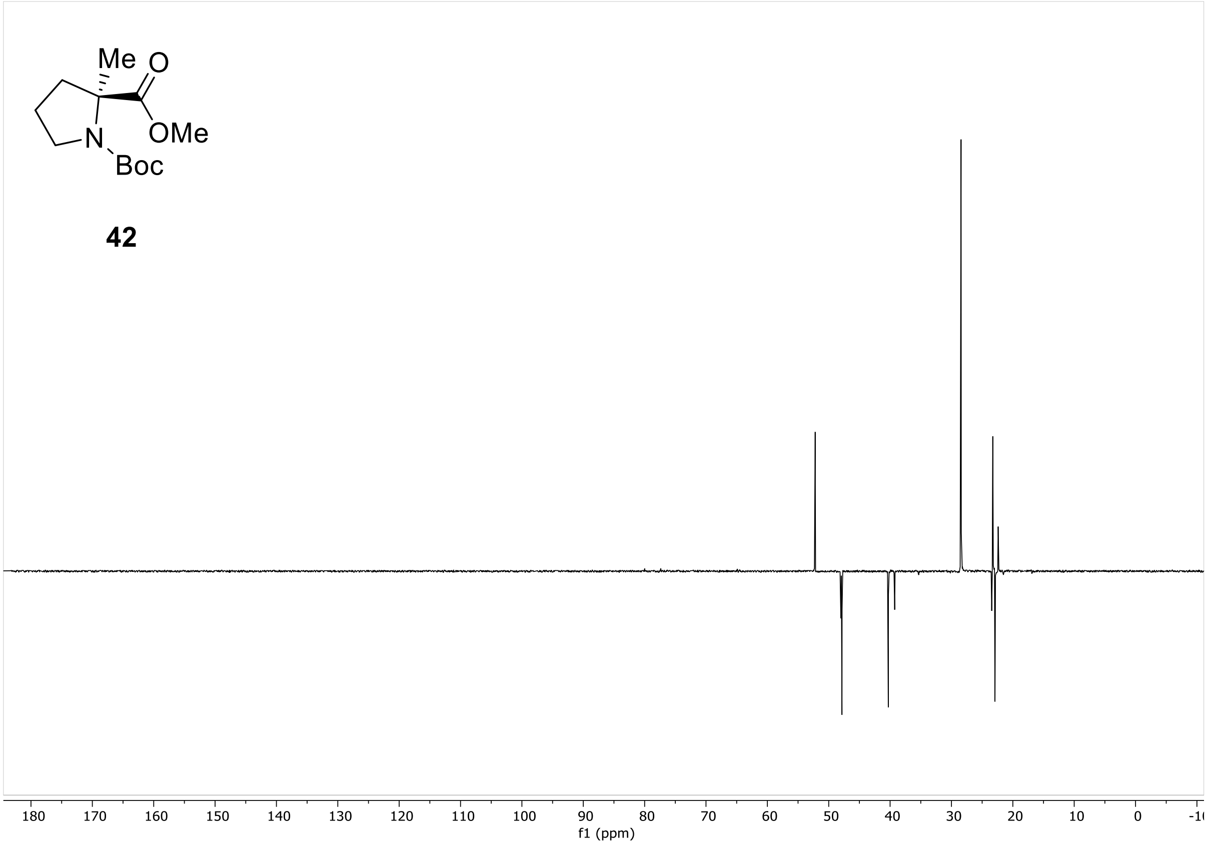

^{1}H NMR (400 MHz, MeOD-d_4, 298 K, mixture of two rotamers) of compound **13**

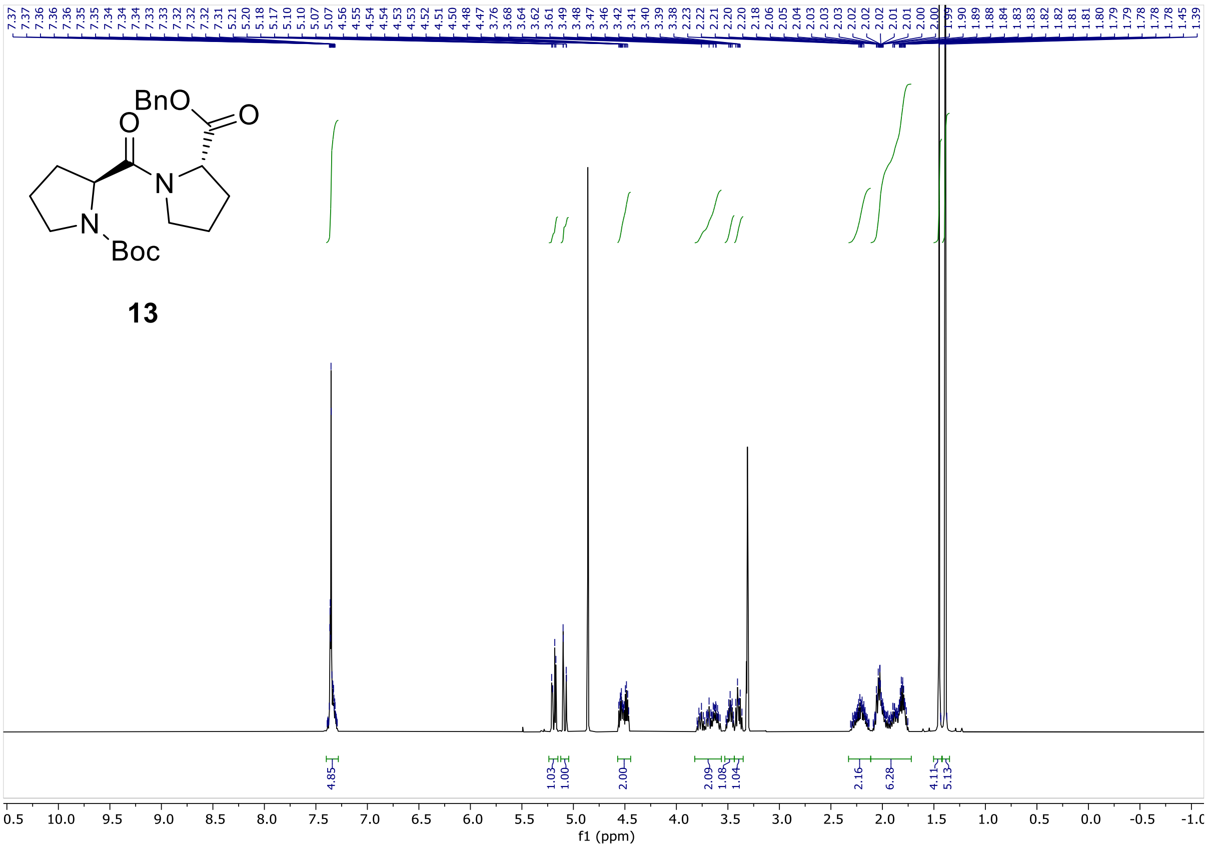

^{13}C NMR (101 MHz, MeOD-d_4, 298 K, mixture of two rotamers) of compound **13**

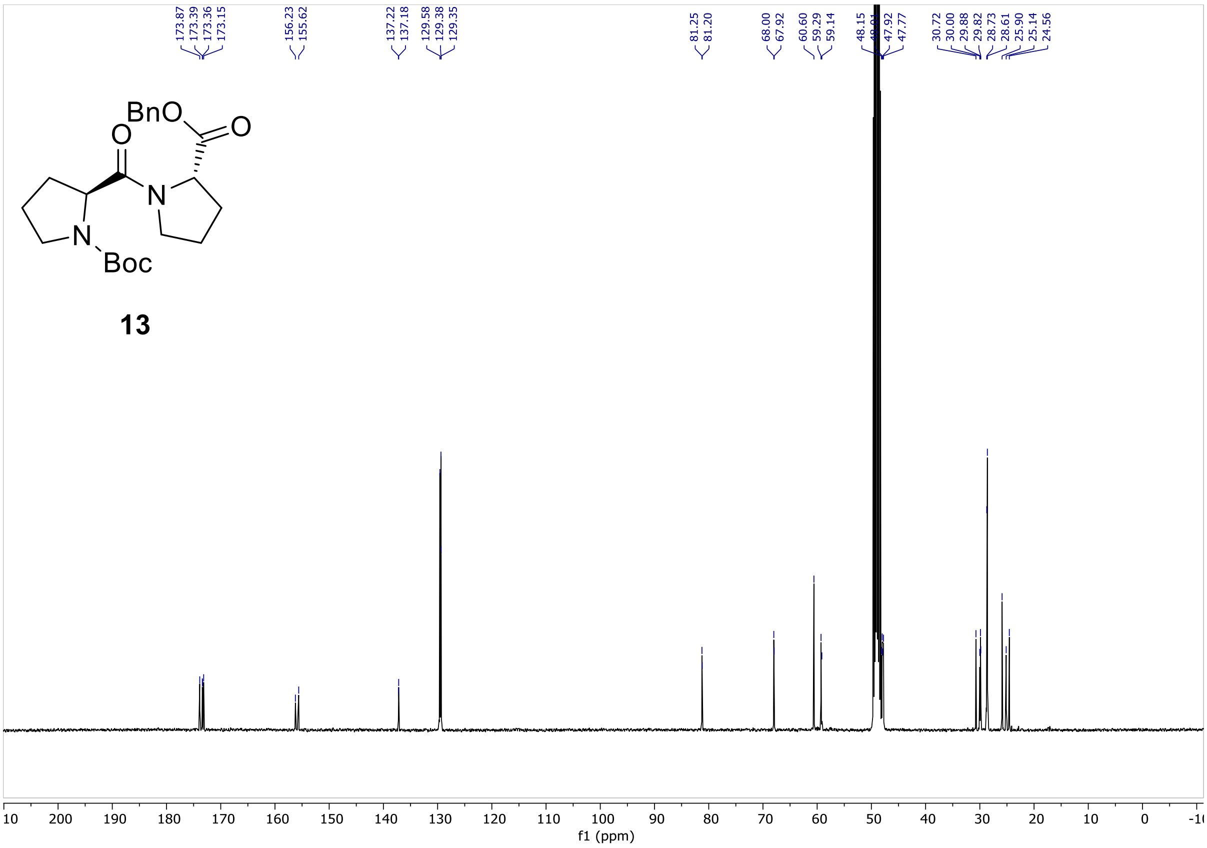

DEPT-135 (101 MHz, MeOD-d_4, 298 K, mixture of tw orotamers) of compound **13**

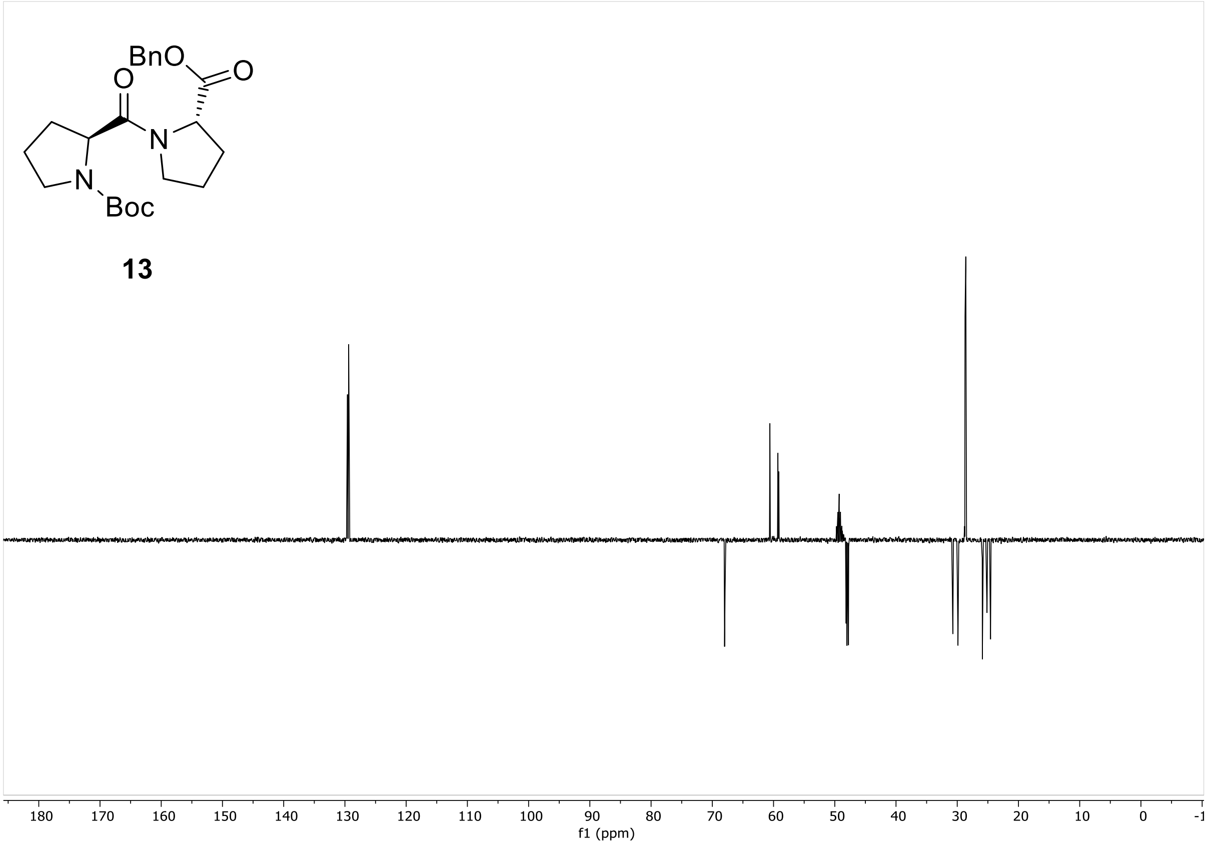

^{1}**H NMR** (400 MHz, $CDCl_3$, 298 K) of compound **45**

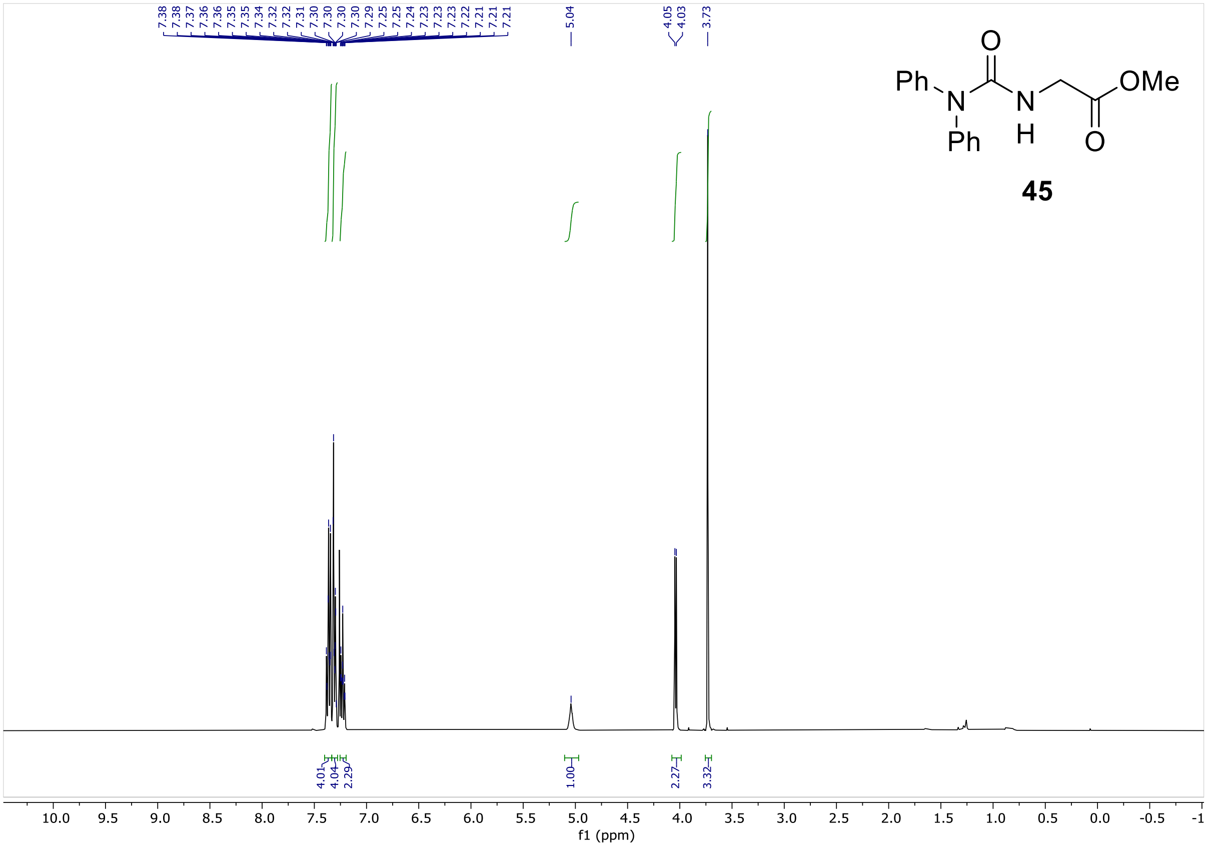

^{13}**C NMR** (101 MHz, $CDCl_3$, 298 K) of compound **45**

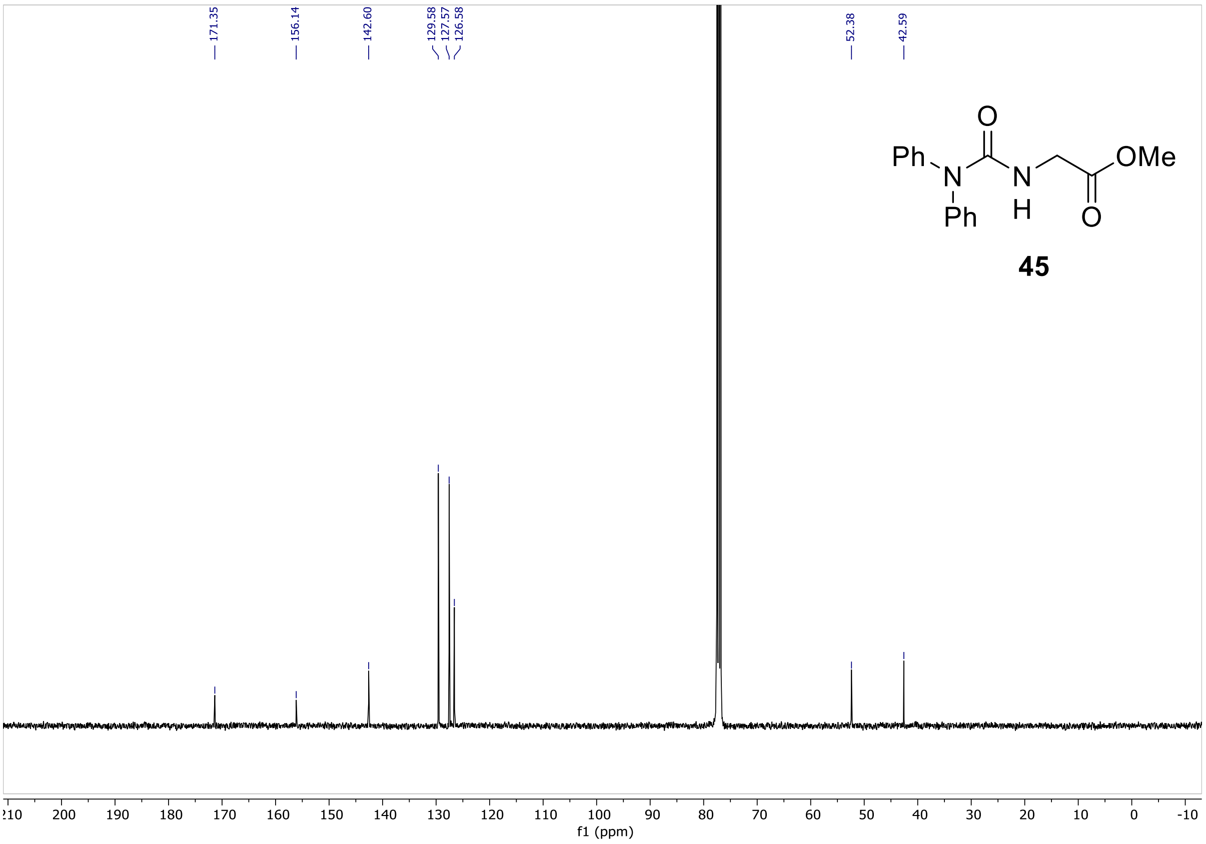

DEPT-135 (101 MHz, $CDCl_3$, 298 K) of compound **45**

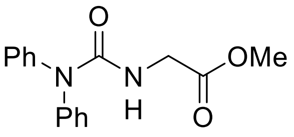

45

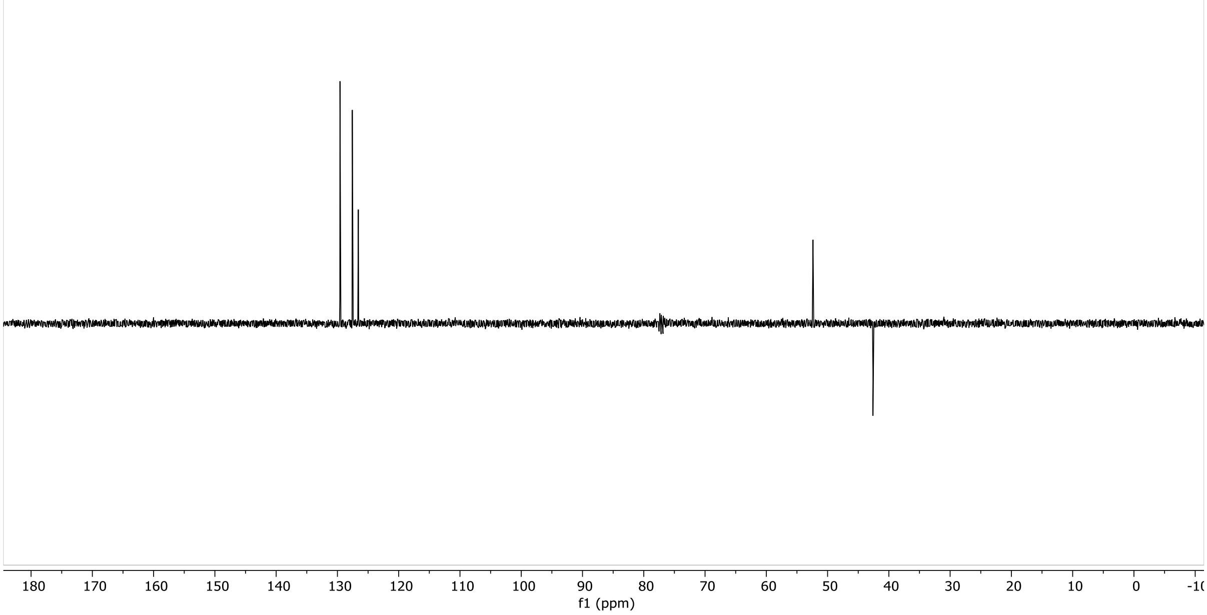

^{1}H NMR (400 MHz, MeOD-d_4, 298 K, mixture of two rotamers) of compound **50**

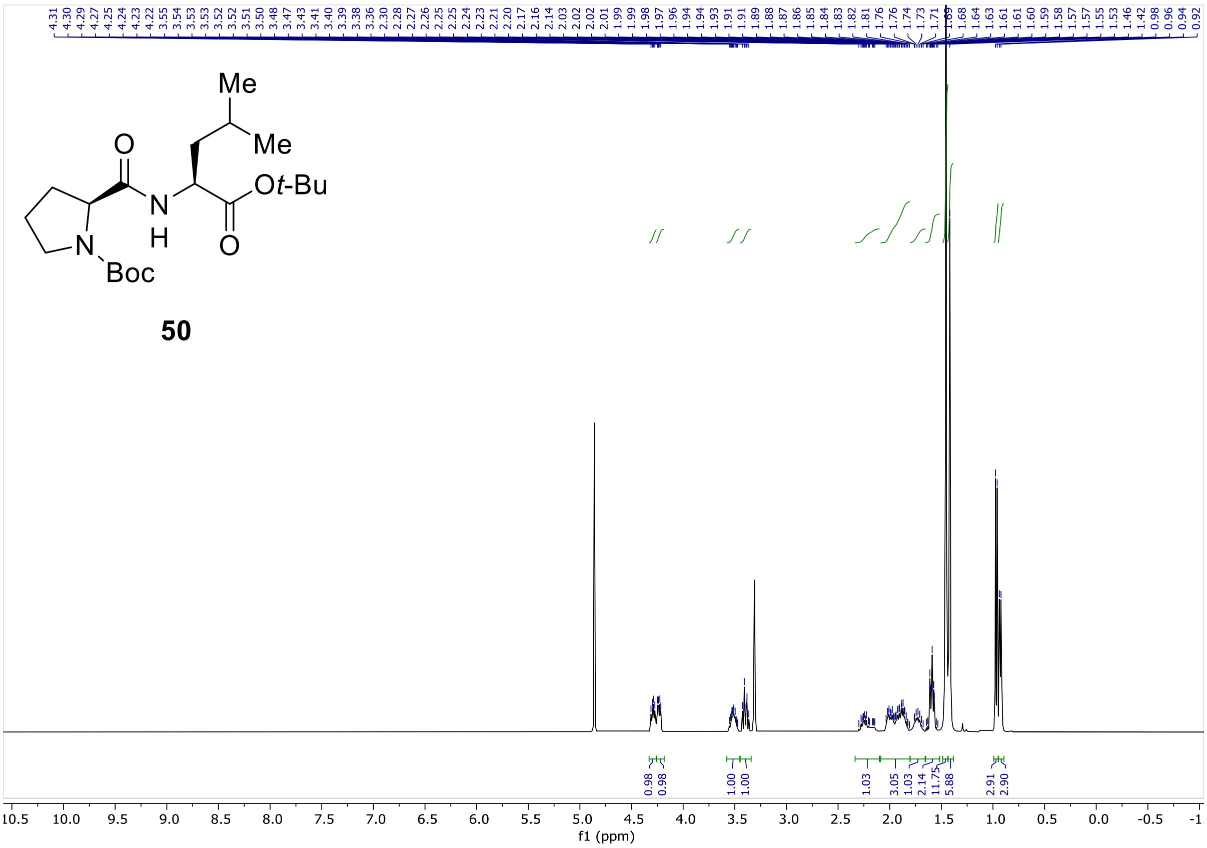

^{13}C NMR (101 MHz, MeOD-d_4, 298 K, mixture of two rotamers) of compound **50**

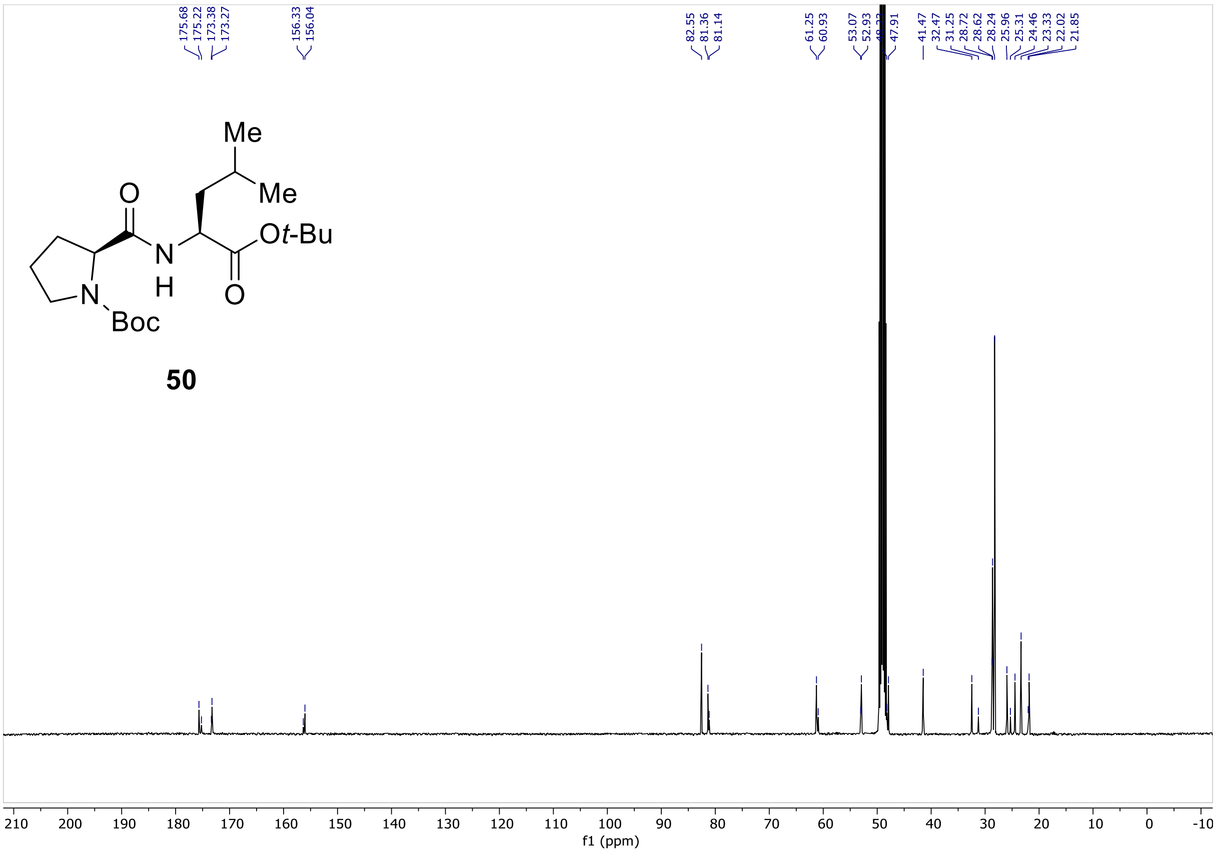

DEPT-135 (101 MHz, MeOD-d_4, 298 K, mixture of two rotamers) of compound **50**

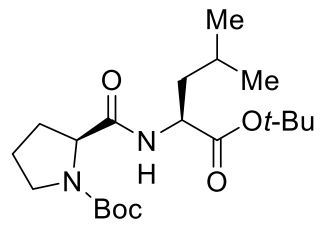

50

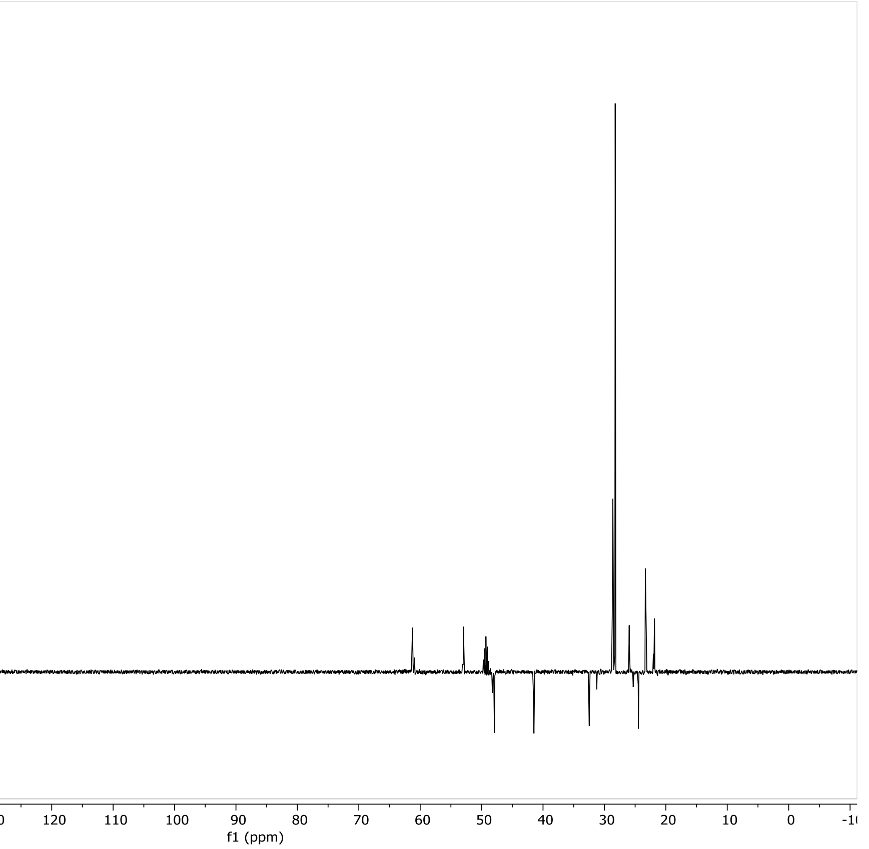

^{1}H NMR (400 MHz, MeOD-d_4, 298 K, mixture of two rotamers) of compound **53**

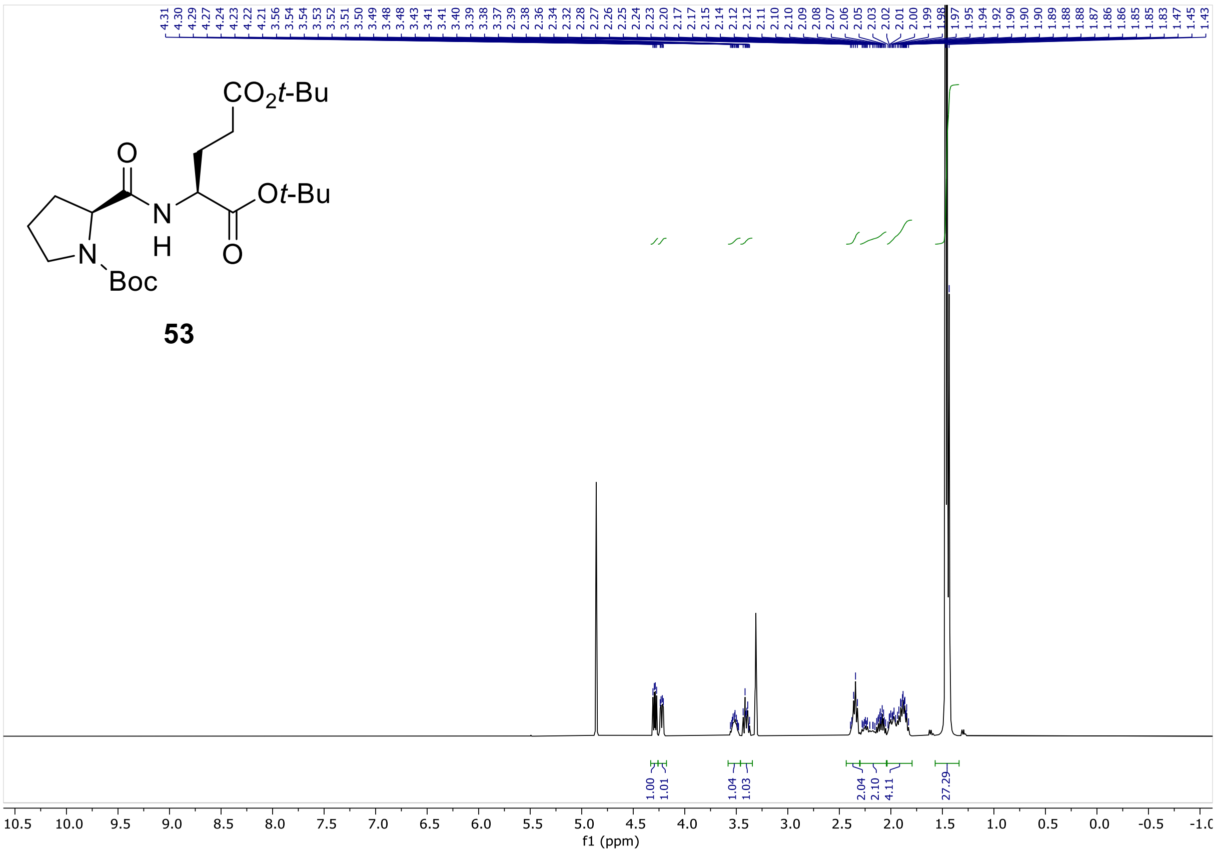

^{13}C NMR (101 MHz, MeOD-d_4, 298 K, mixture of two rotamers) of compound **53**

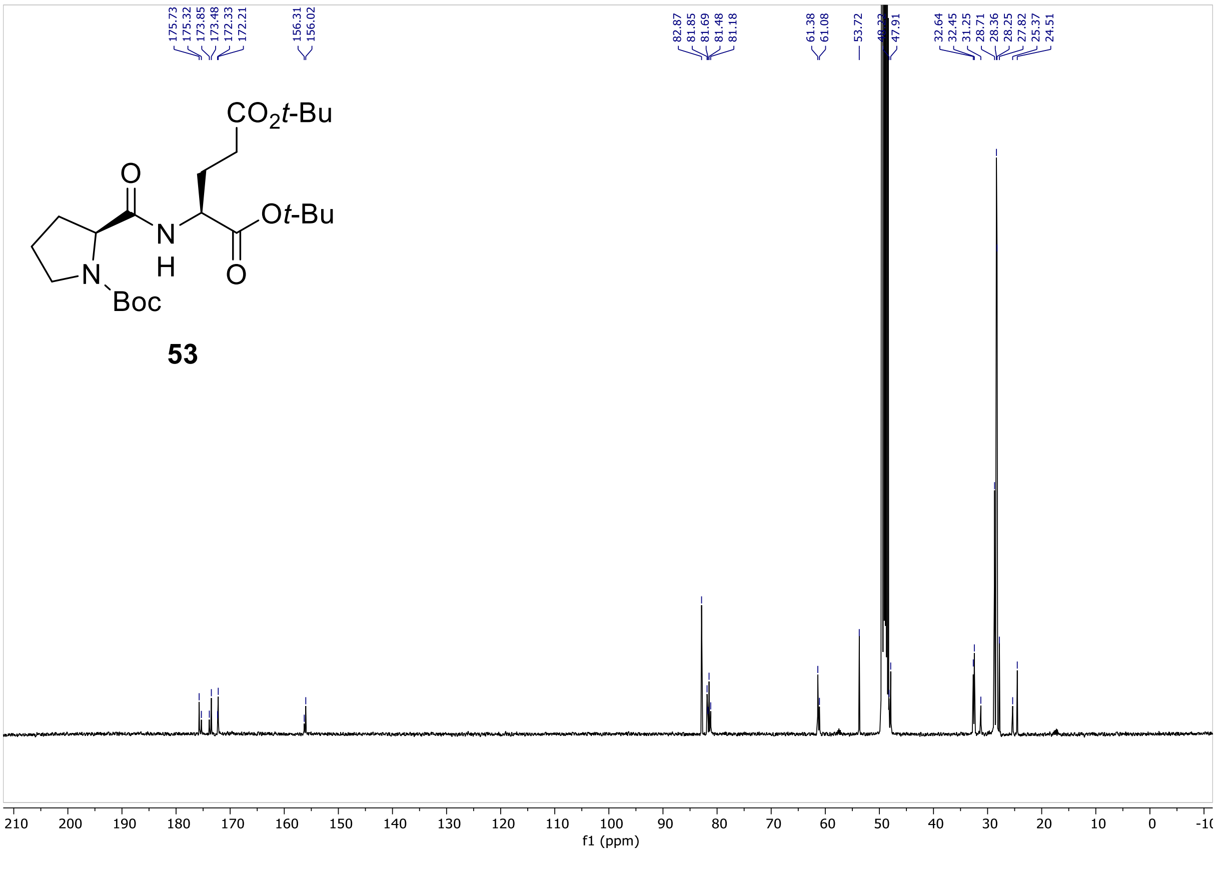

DEPT-135 (101 MHz, MeOD-d_4, 298 K, mixture of two rotamers) of compound **53**

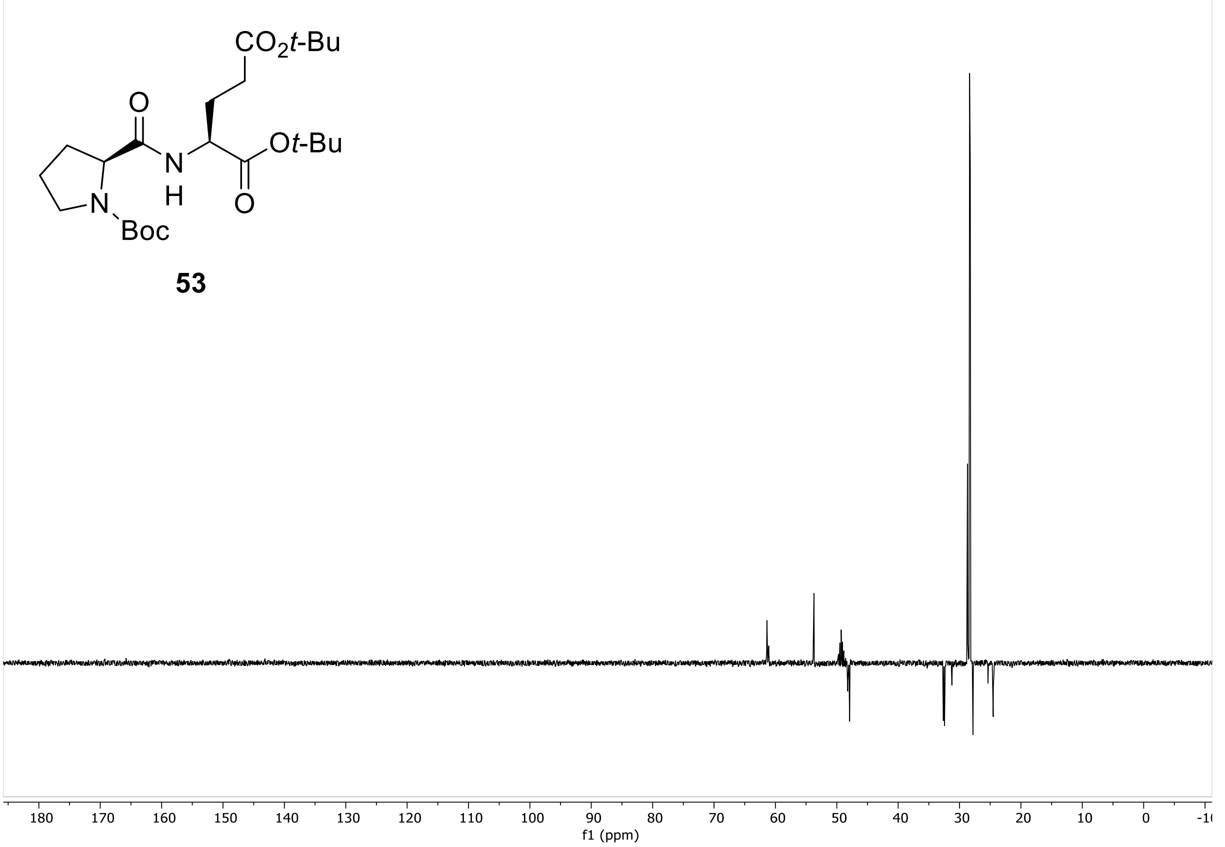

^{1}H NMR (400 MHz, MeOD-d_4, 298 K, mixture of two rotamers) of compound **54**

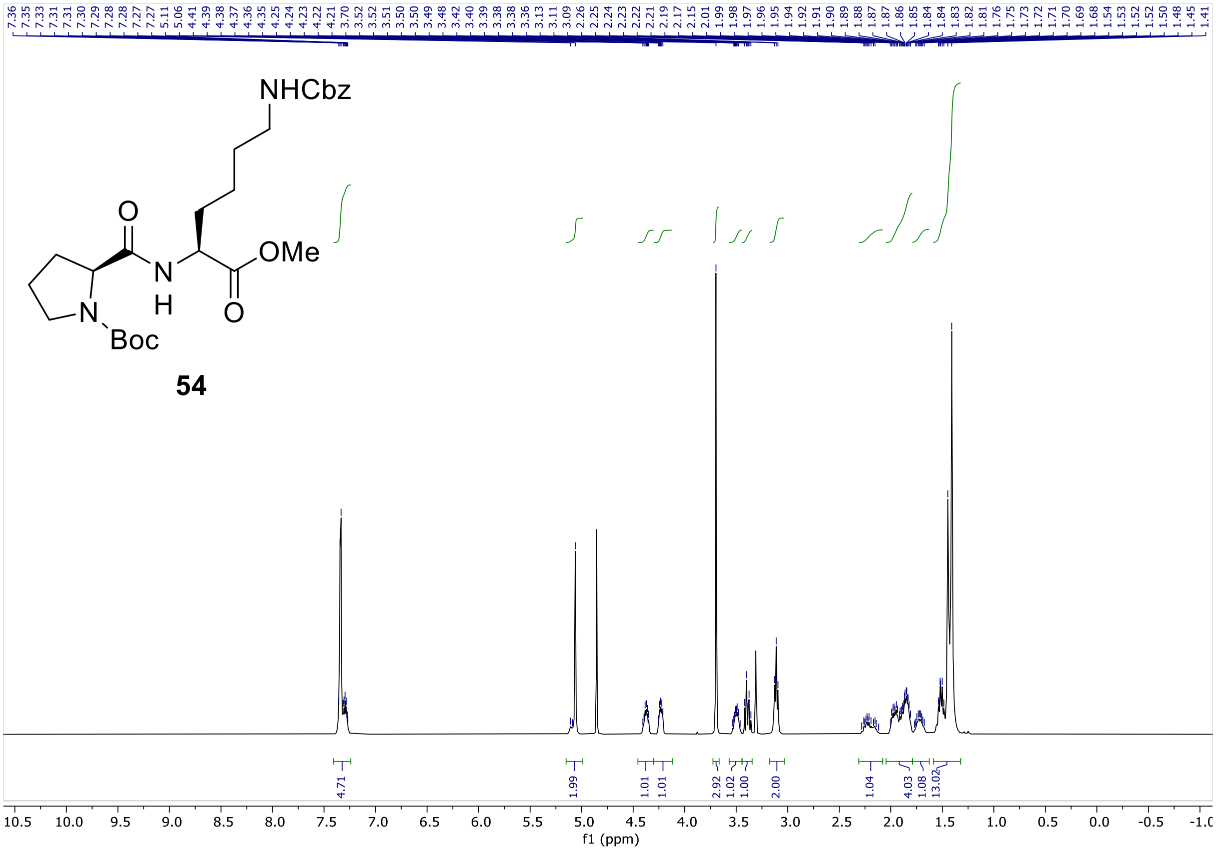

^{13}C NMR (101 MHz, MeOD-d_4, 298 K, mixture of two rotamers) of compound **54**

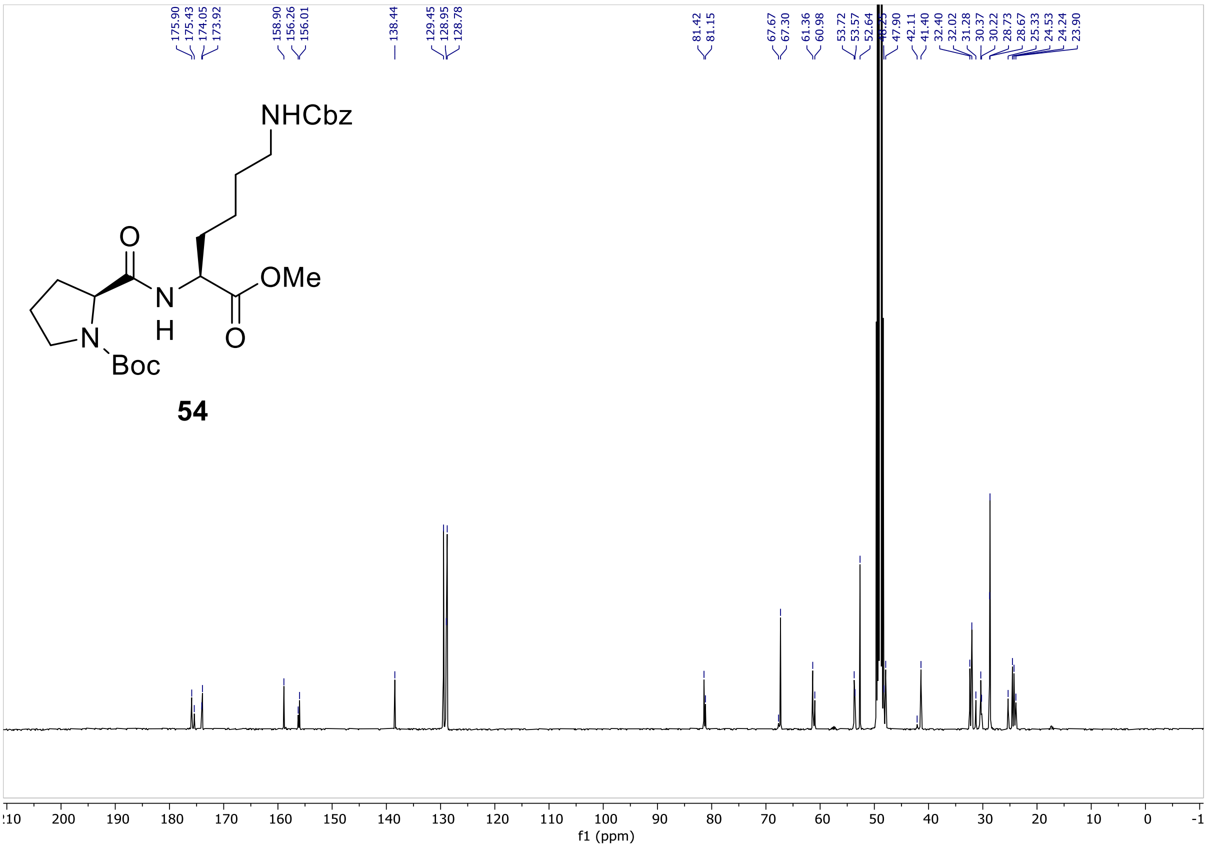

DEPT-135 (101 MHz, MeOD-d_4, 298 K, mixture of two rotamers) of compound **54**

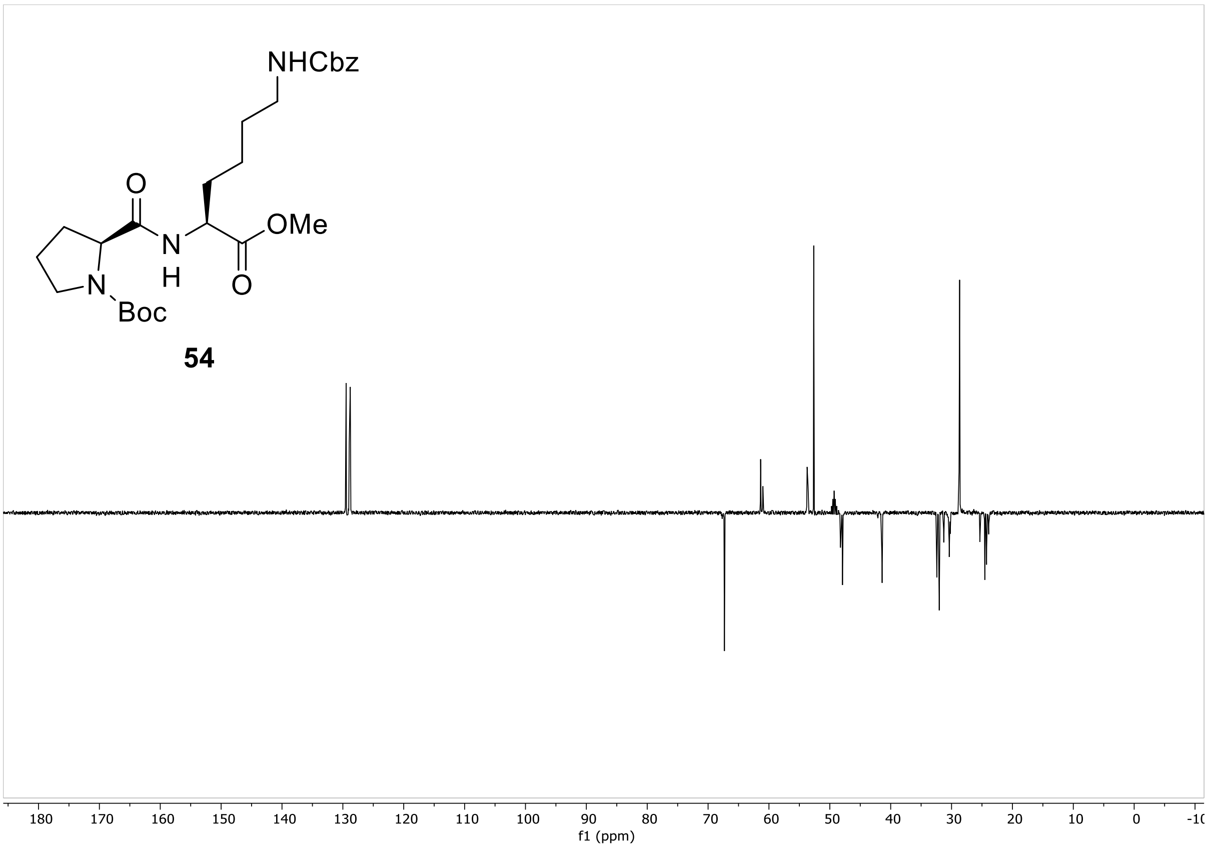

^{1}H NMR (400 MHz, MeOD-d_4, 298 K) of compound **56**

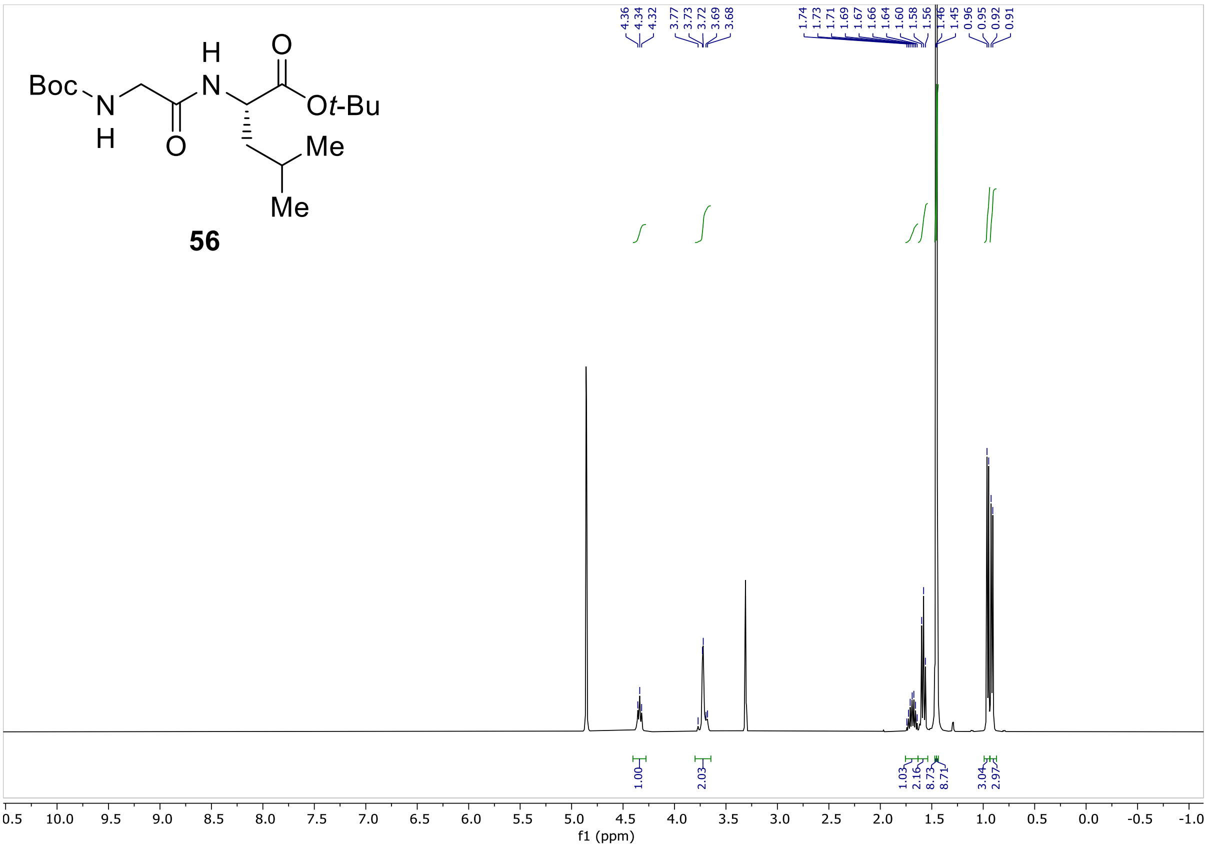

^{13}C NMR (101 MHz, MeOD-d_4, 298 K) of compound **56**

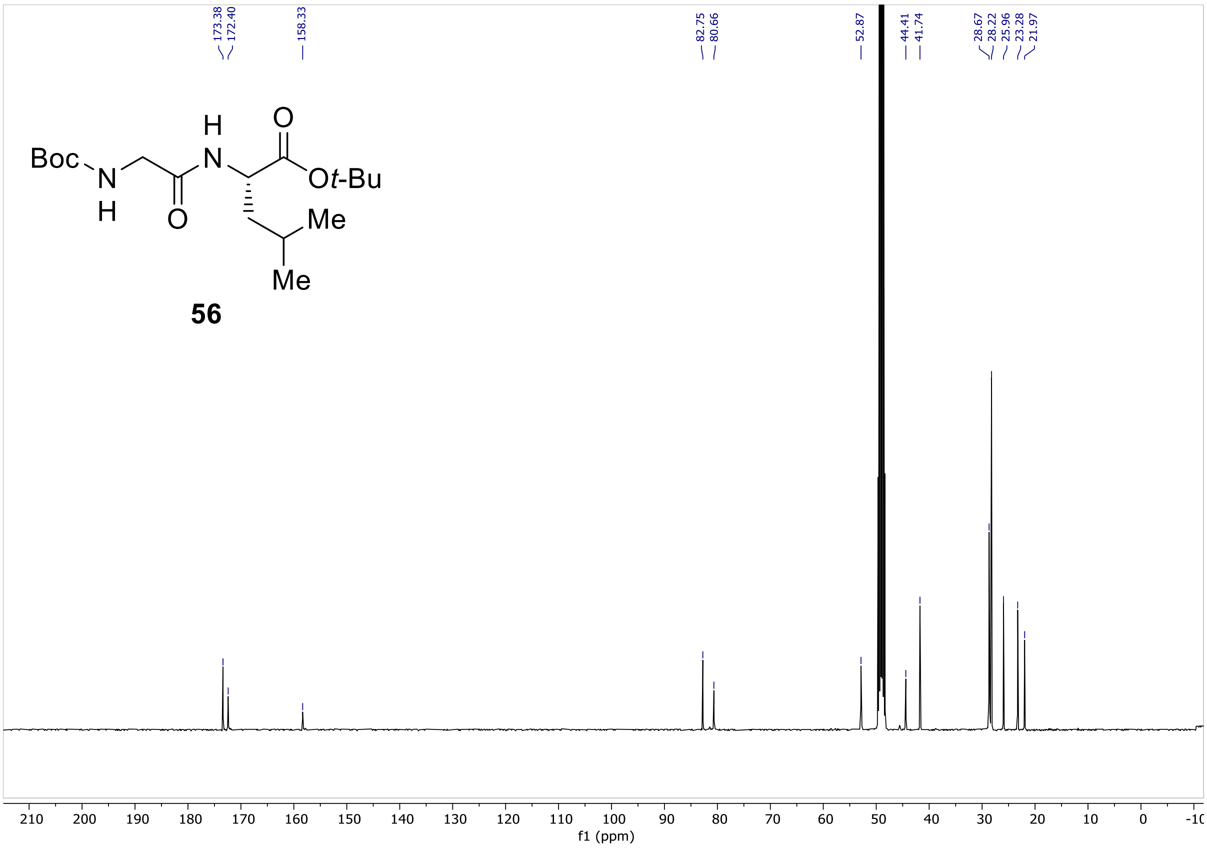

DEPT-135 (101 MHz, MeOD-d_4, 298 K) of compound **56**

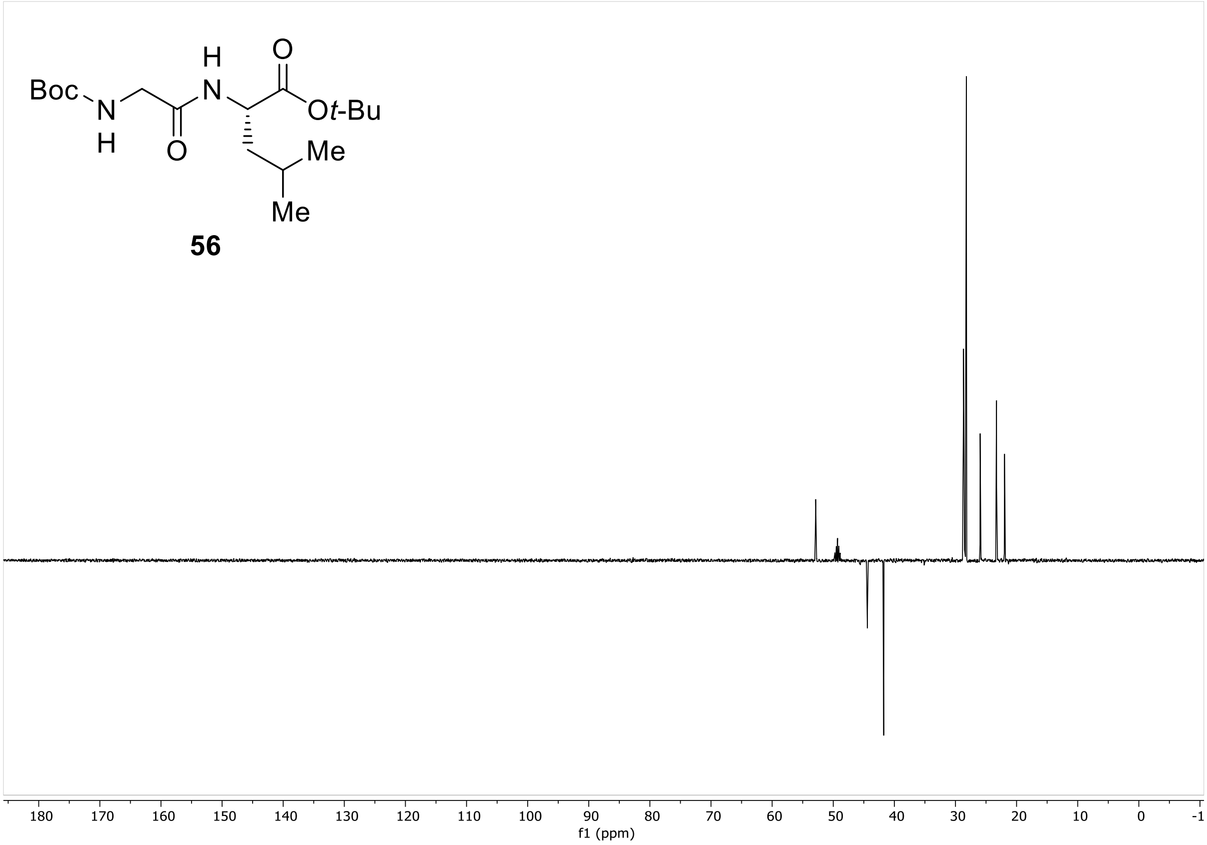

^{1}H NMR (400 MHz, MeOD-d_4, 298 K, complex mixture of rotamers) of compound **57**

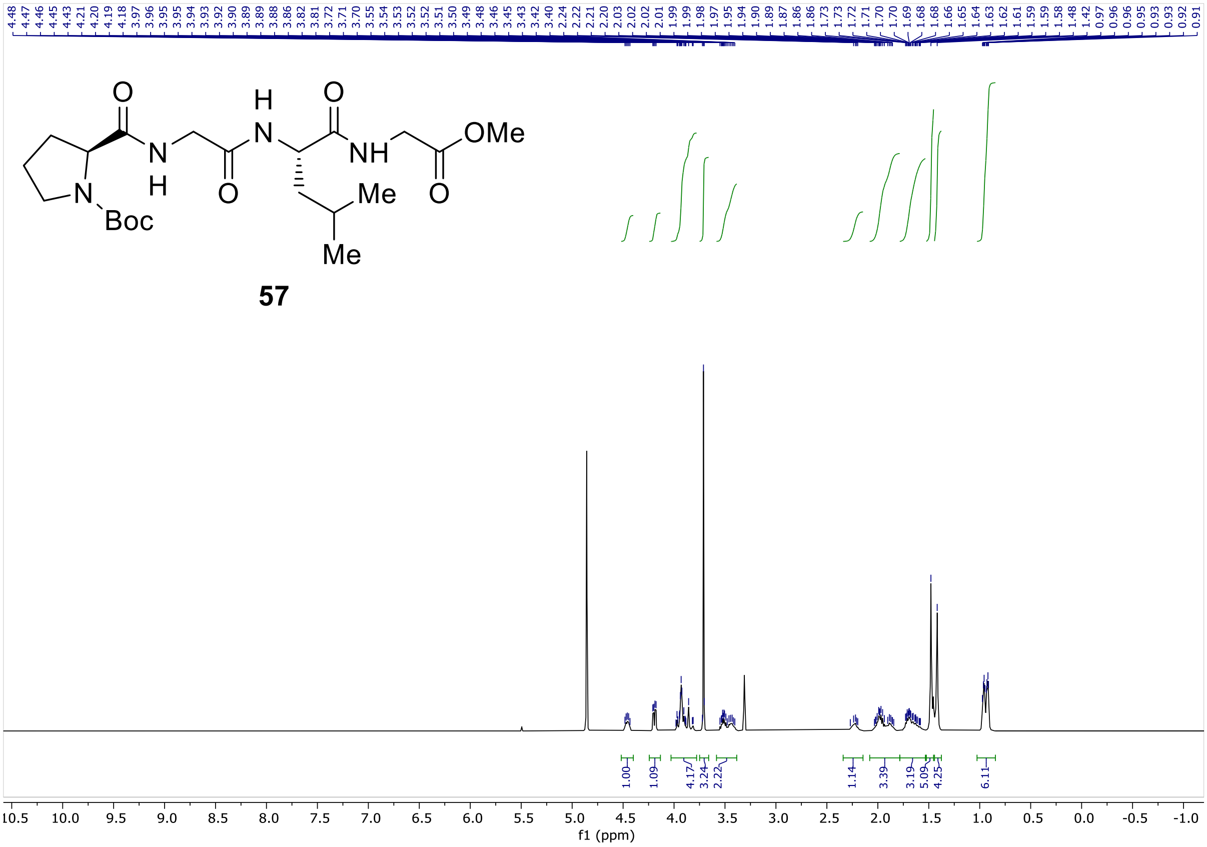

^{13}C NMR (101 MHz, MeOD-d_4, 298 K, complex mixture of rotamers) of compound **57**

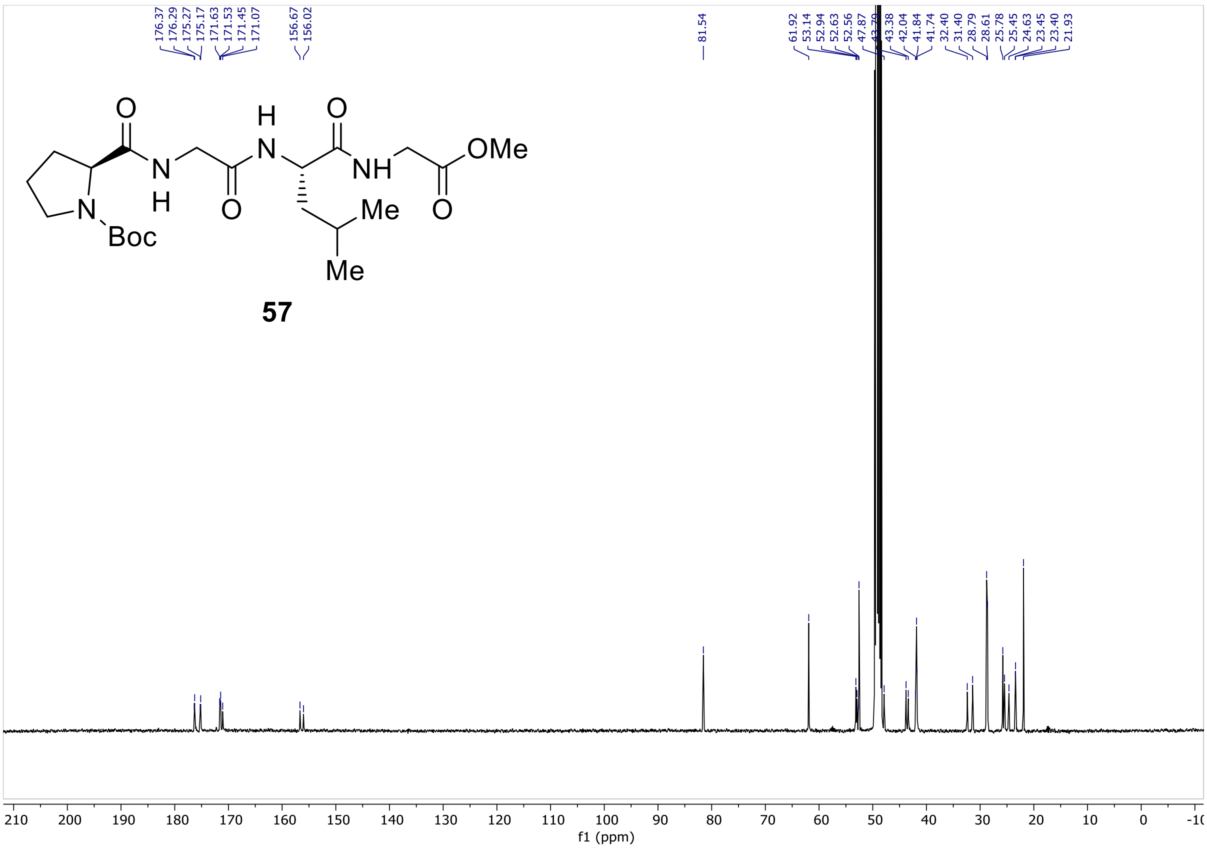

DEPT-135 (101 MHz, MeOD-d_4, 298 K, complex mixture of rotamers) of compound **57**

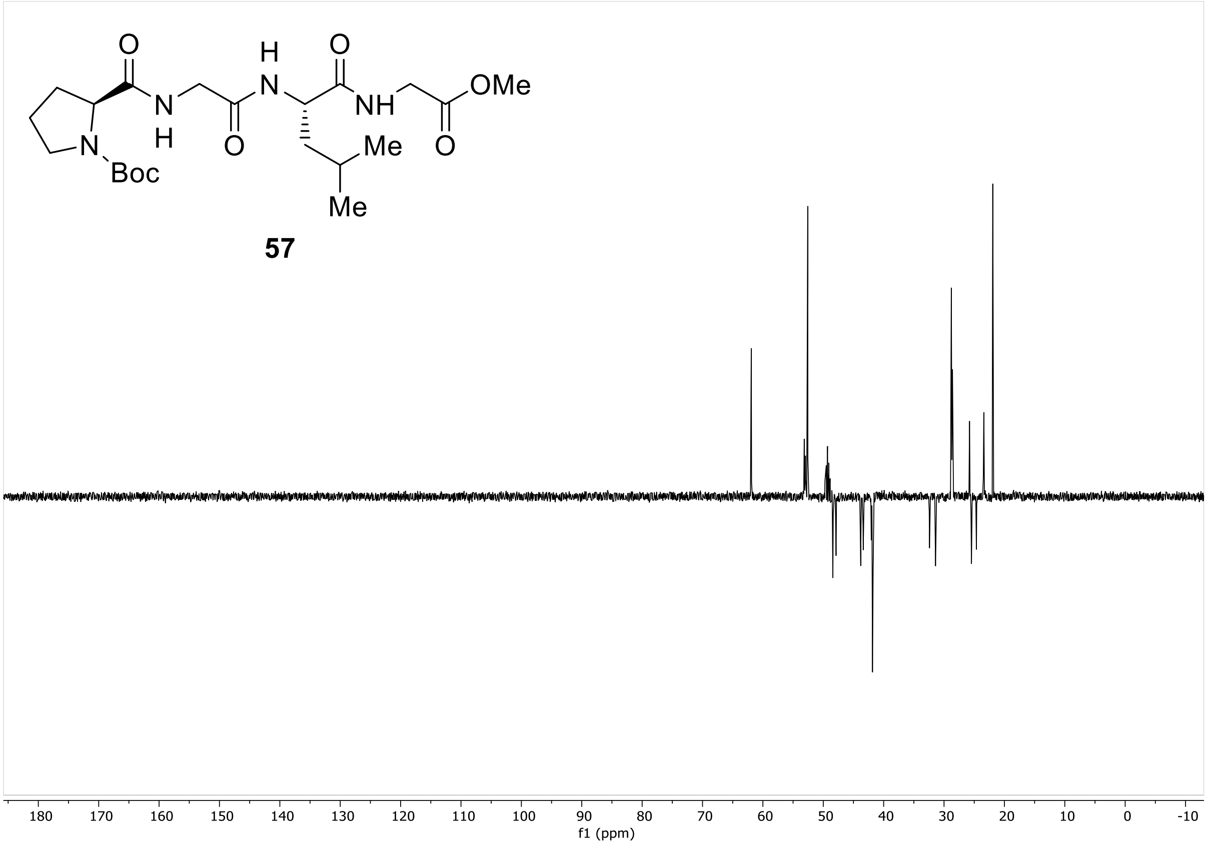

^{1}**H NMR** (400 MHz, MeOD-*d*$_4$, 298 K, mixture of rotamers) of compound **58**

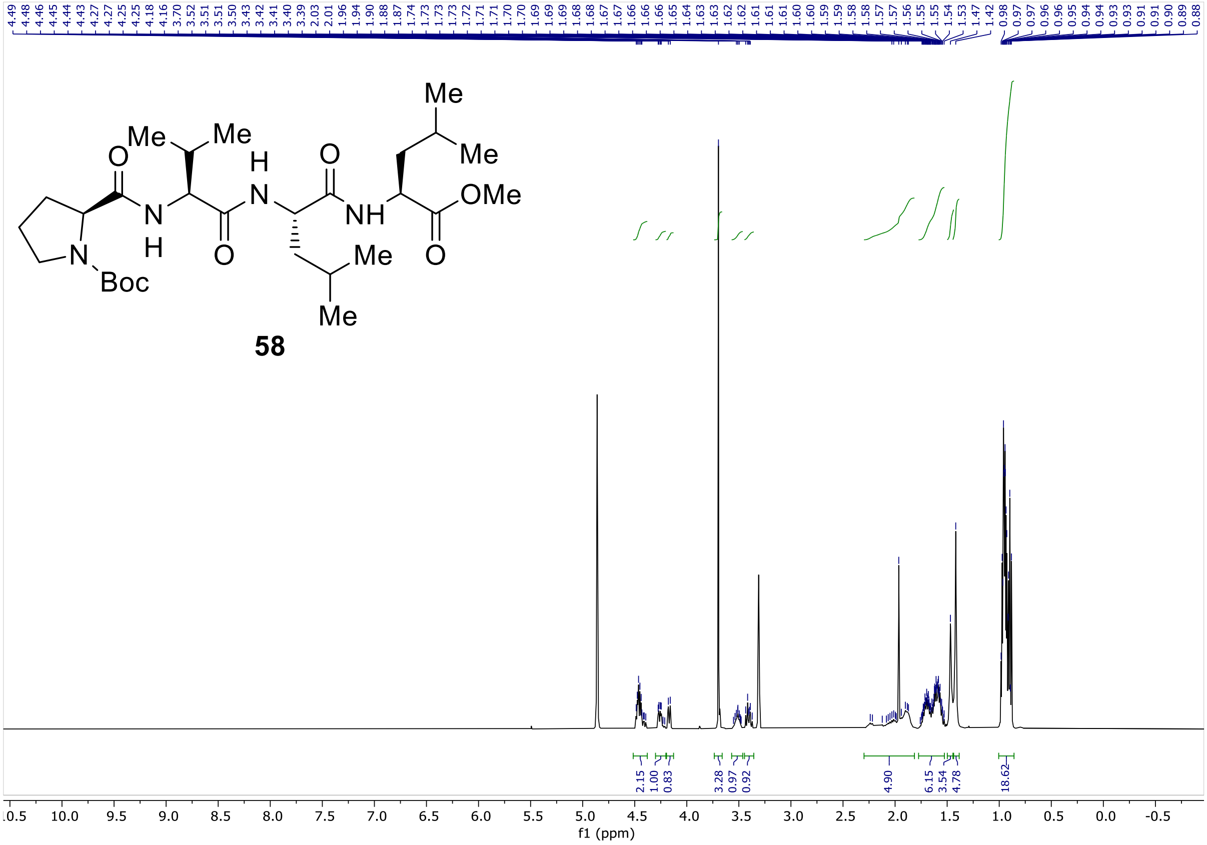

^{13}**C NMR** (101 MHz, MeOD-*d*$_4$, 298 K, mixture of rotamers) of compound **58**

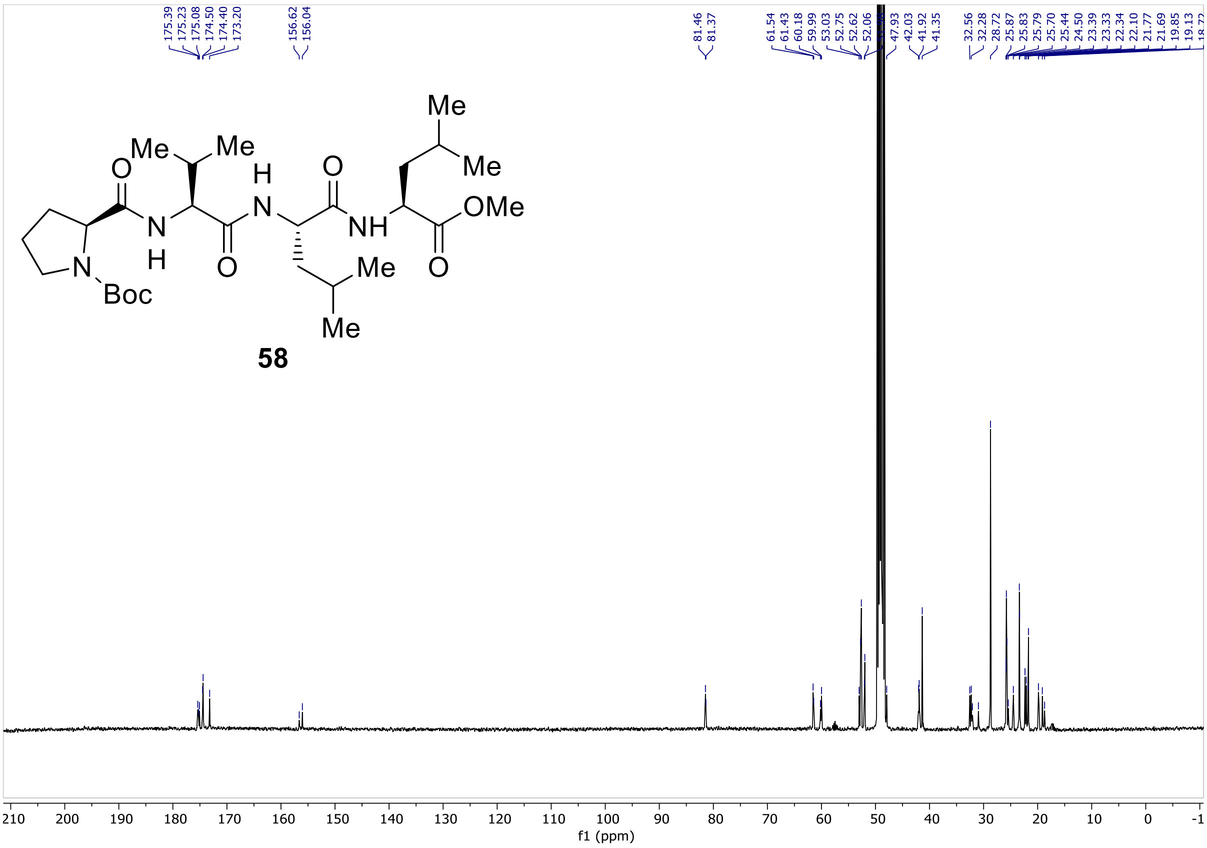

DEPT-135 (101 MHz, MeOD-d_4, 298 K, mixture of rotamers) of compound **58**

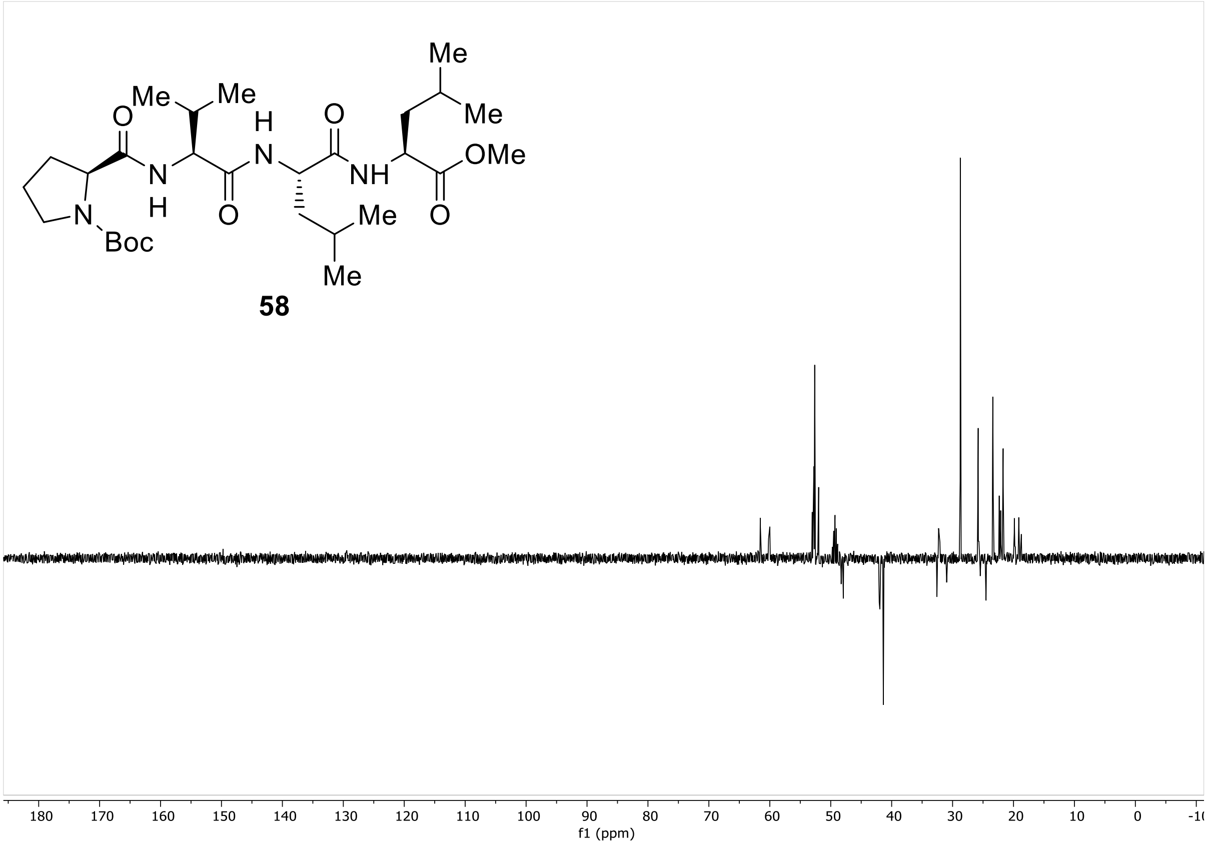

^{1}H NMR (400 MHz, CD_2Cl_2, 298 K, mixture of rotamers) of compound **59**

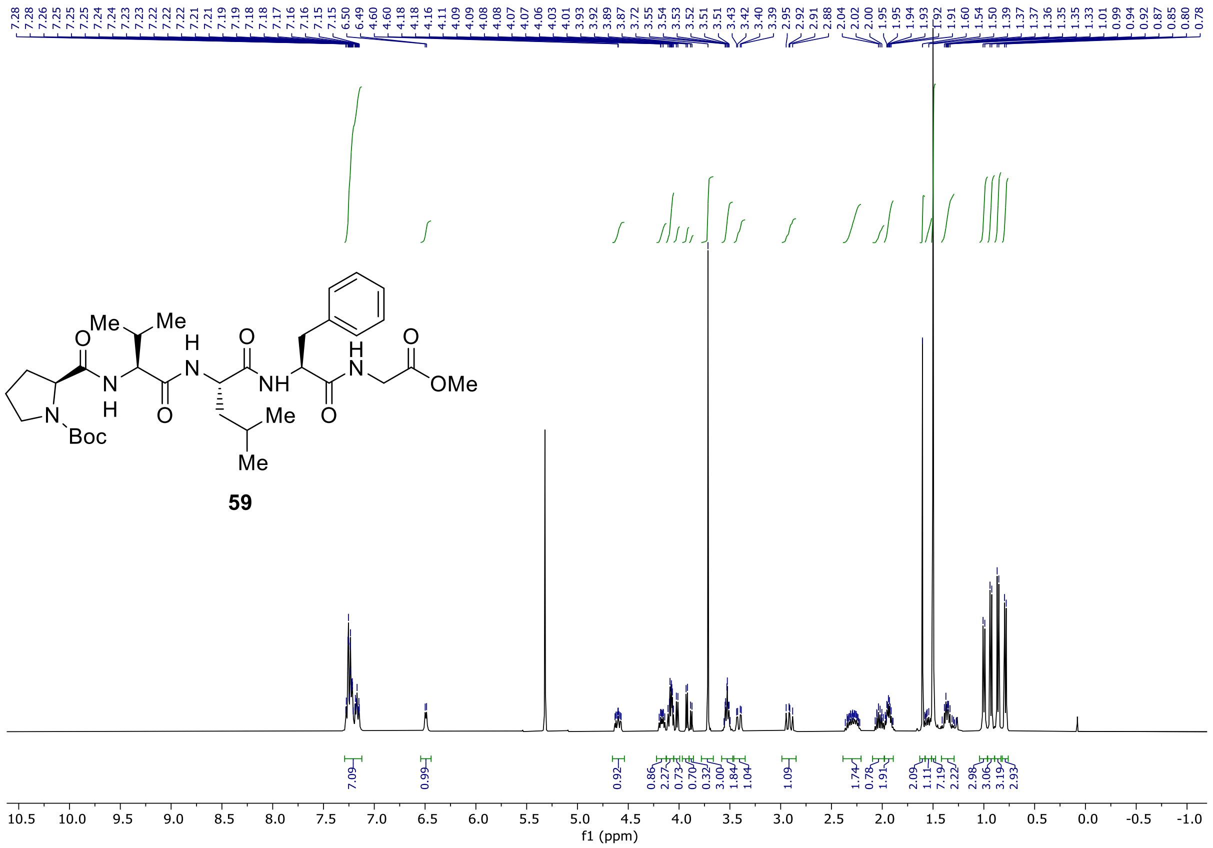

^{13}C NMR (101 MHz, CD_2Cl_2, 298 K, mixture of rotamers) of compound **59**

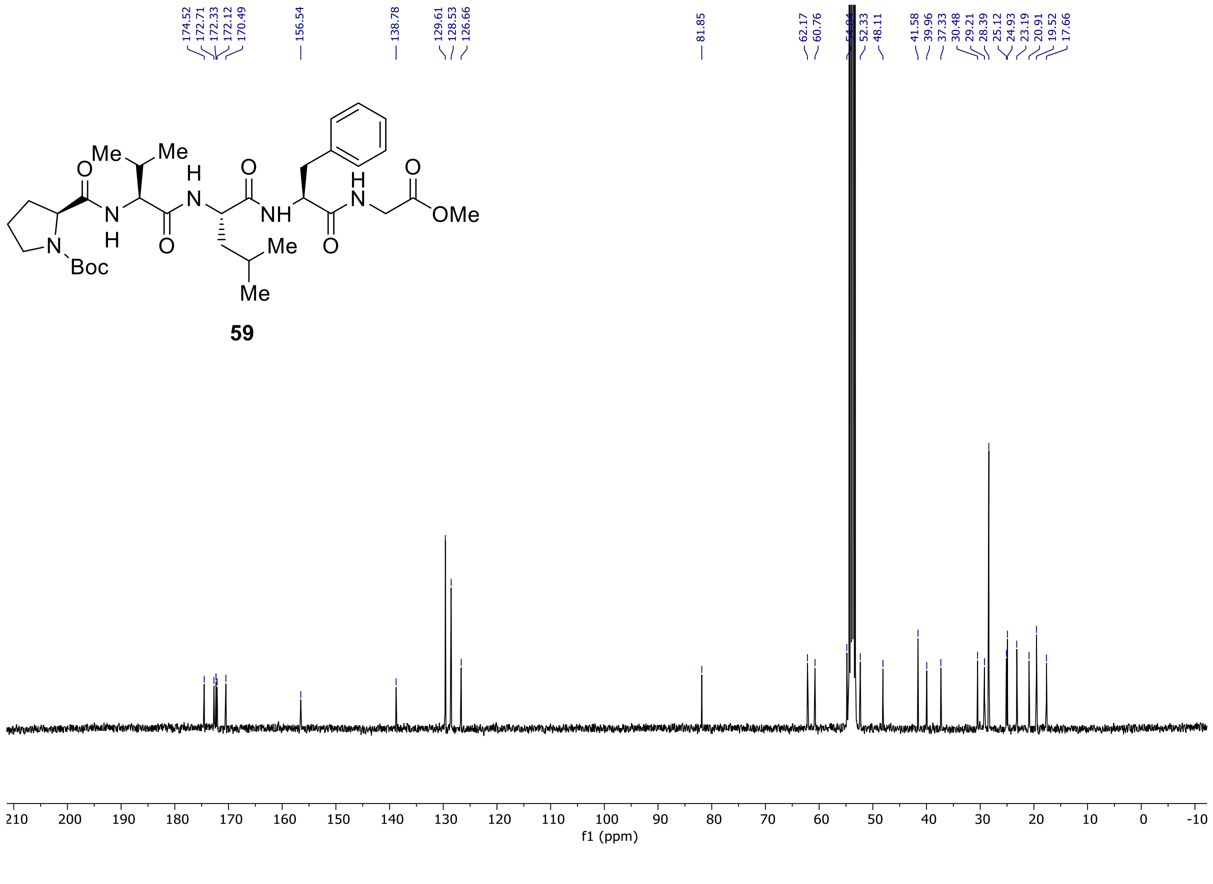

DEPT-135 (101 MHz, CD_2Cl_2, 298 K, mixture of rotamers) of compound **59**

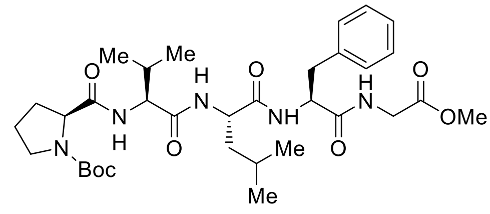

59

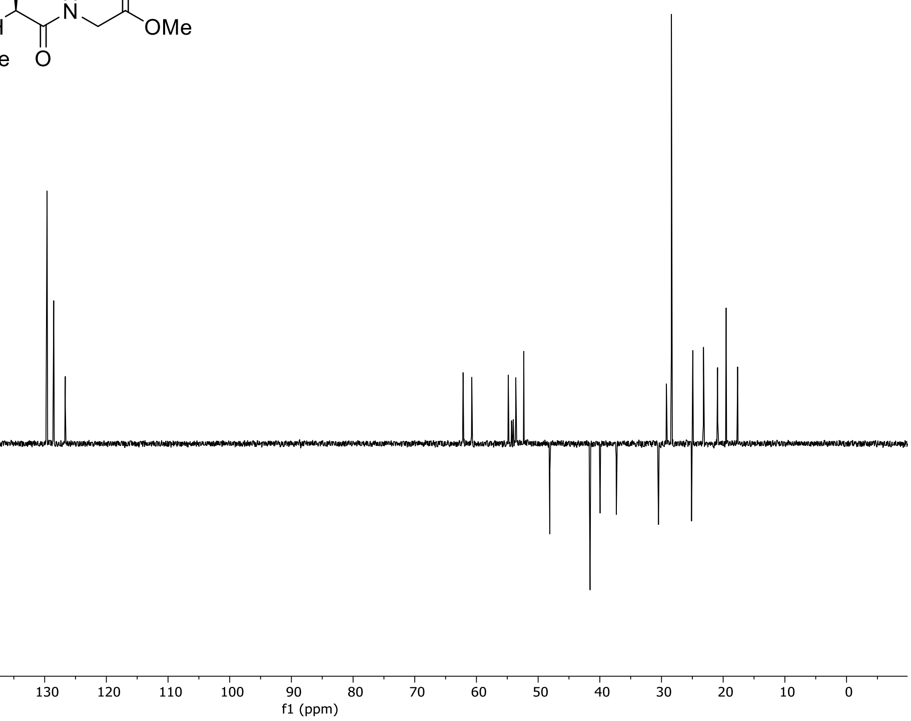

^{1}H NMR (400 MHz, MeOD-d_4, 298 K, mixture of rotamers) of compound **60**

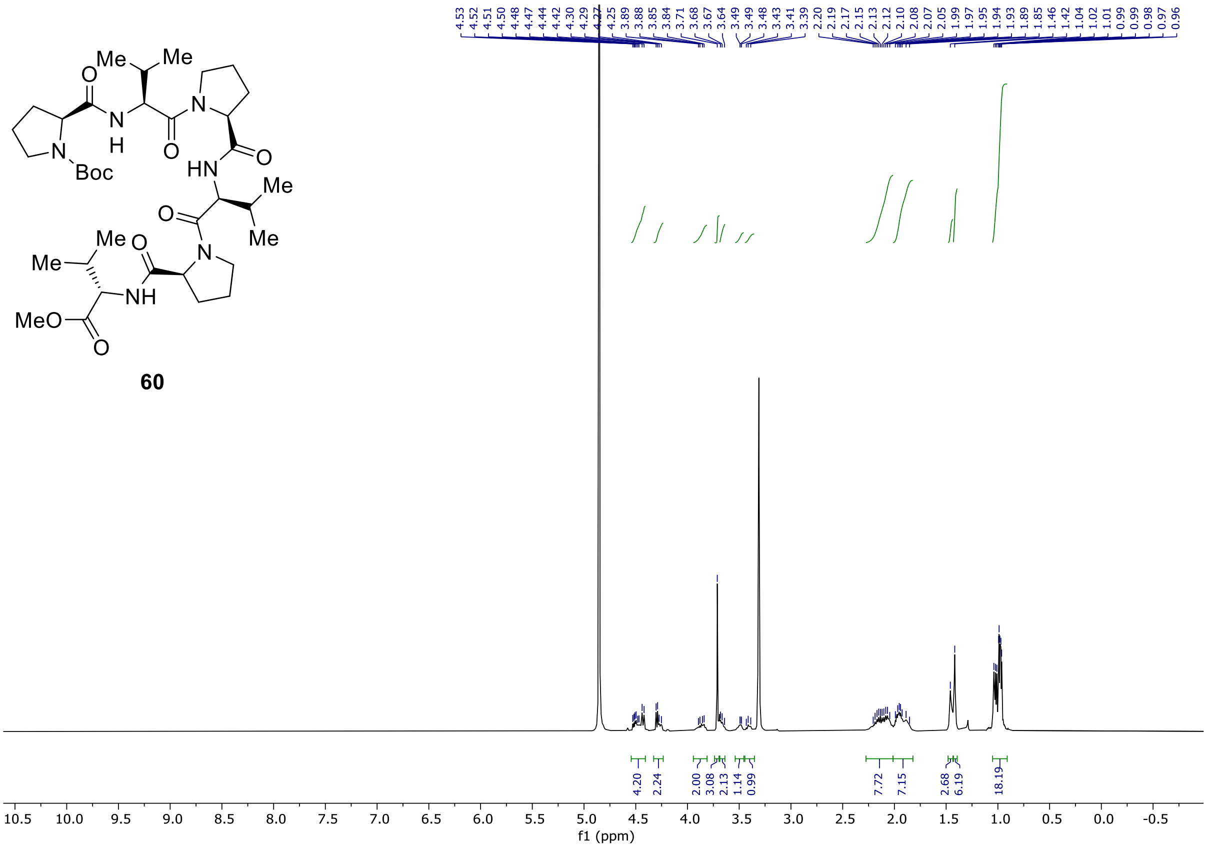

^{13}C NMR (101 MHz, MeOD-d_4, 298 K, complex mixture of rotamers) of compound **60**

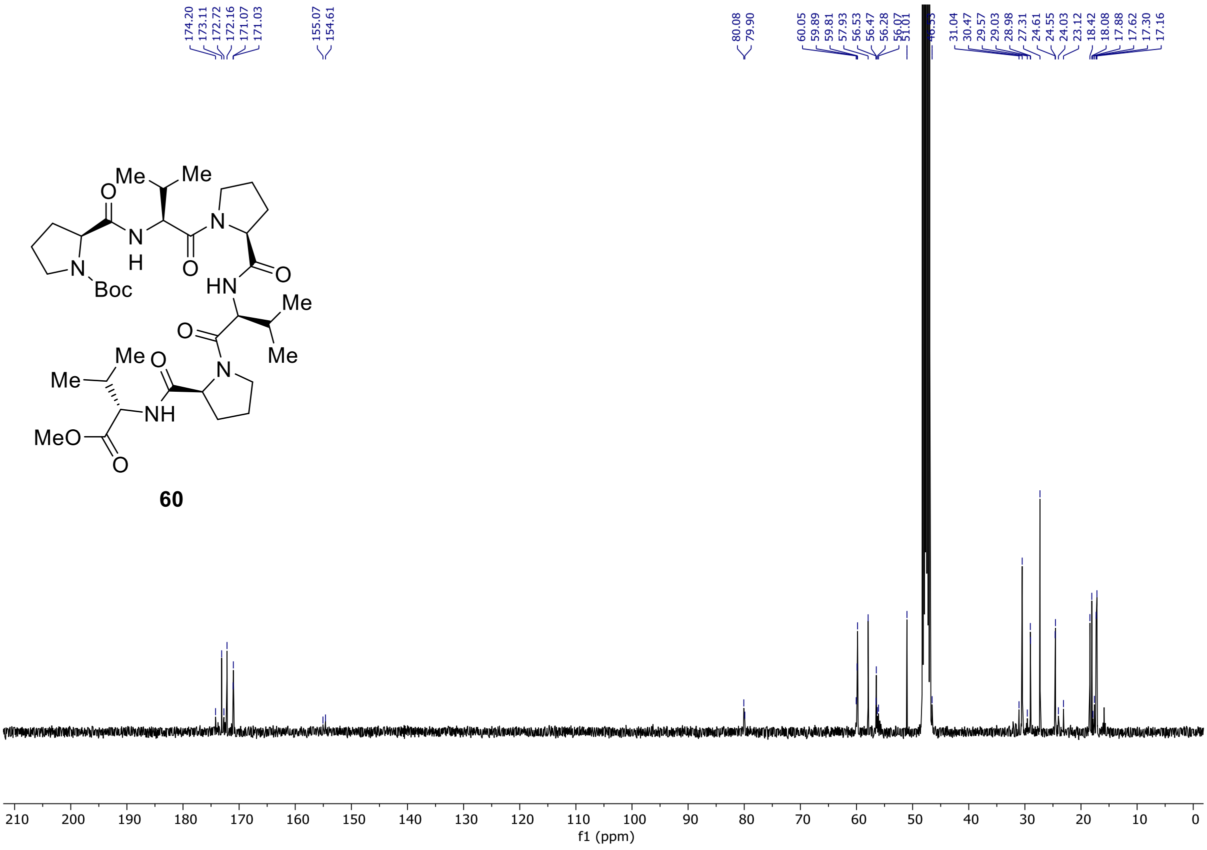

HSQC (MeOD-d_4, 298 K, complex mixture of rotamers) of compound **60**

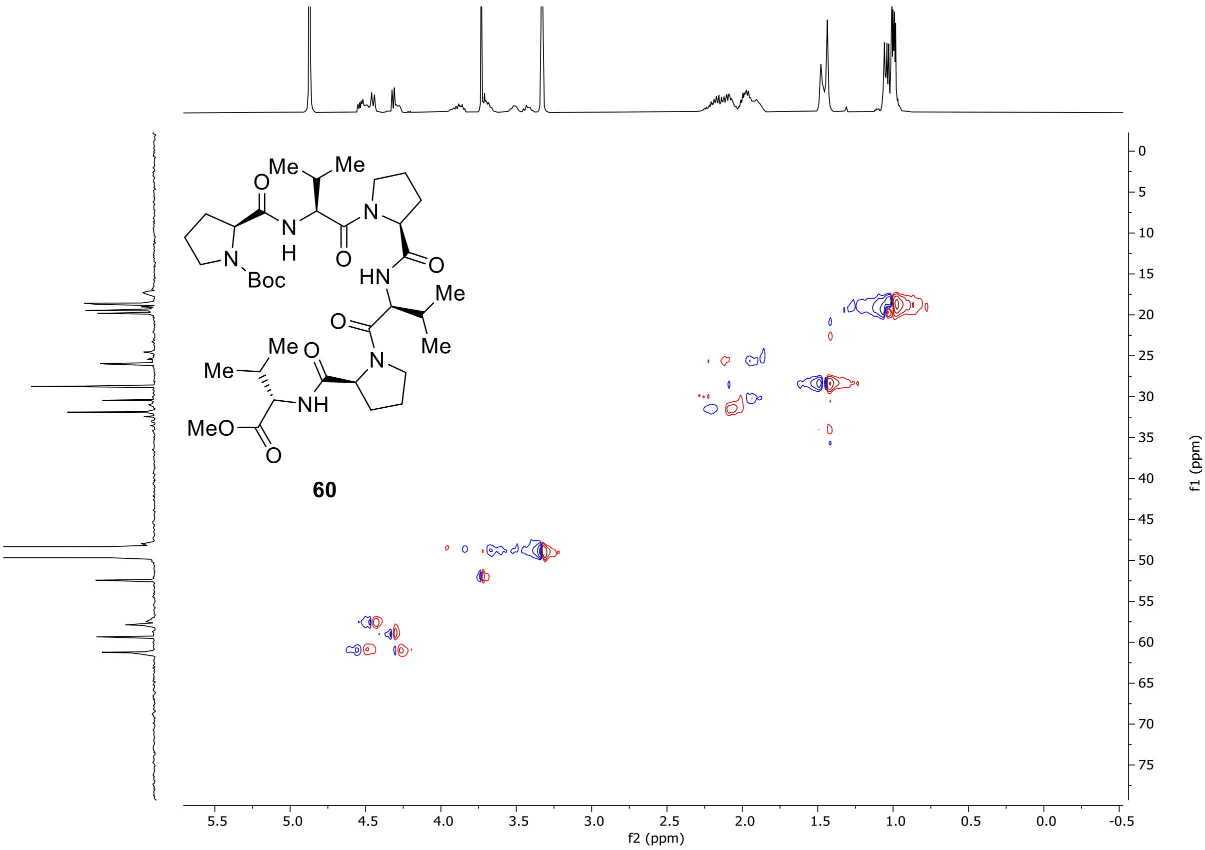

DEPT-135 (101 MHz, MeOD-d_4, 298 K, complex mixture of rotamers) of compound **60**

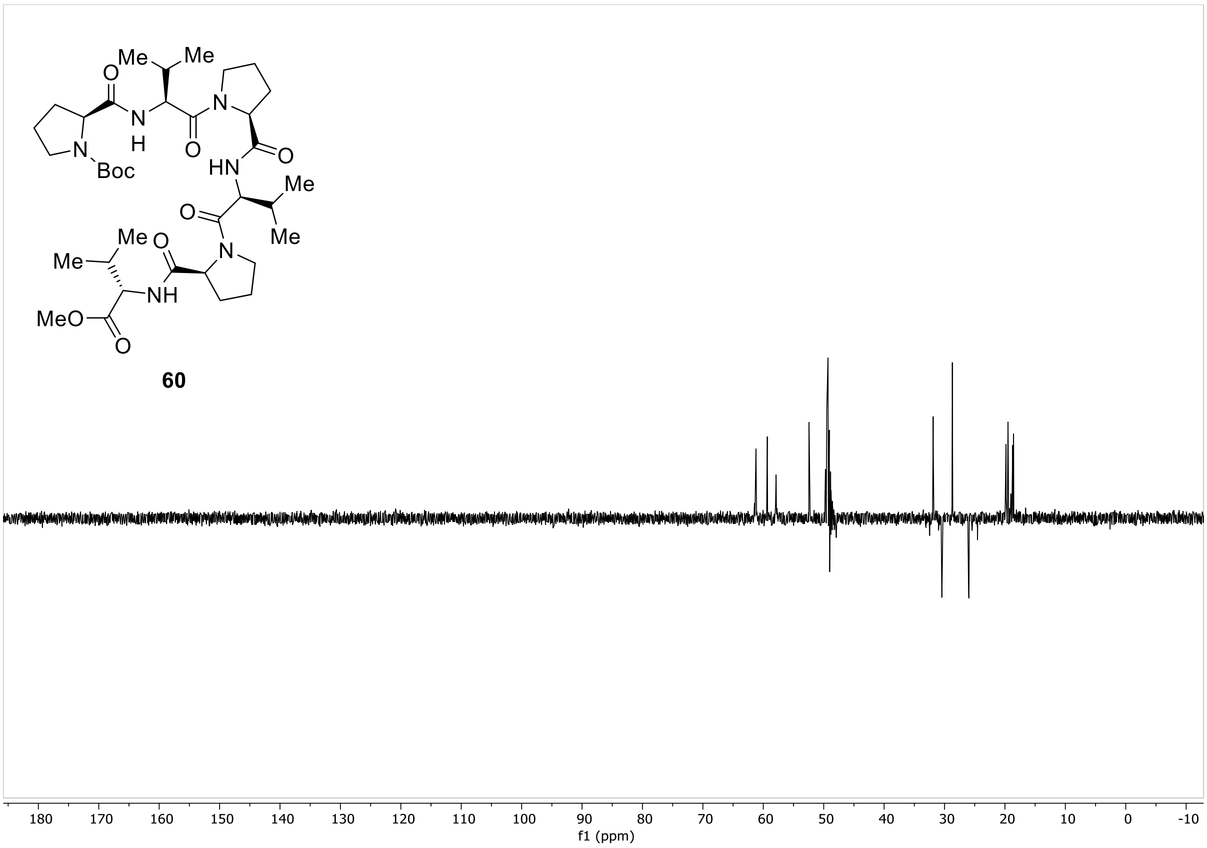

^{1}H NMR (400 MHz, MeOD-*d₄*, 298 K, mixture of rotamers) of compound **61**

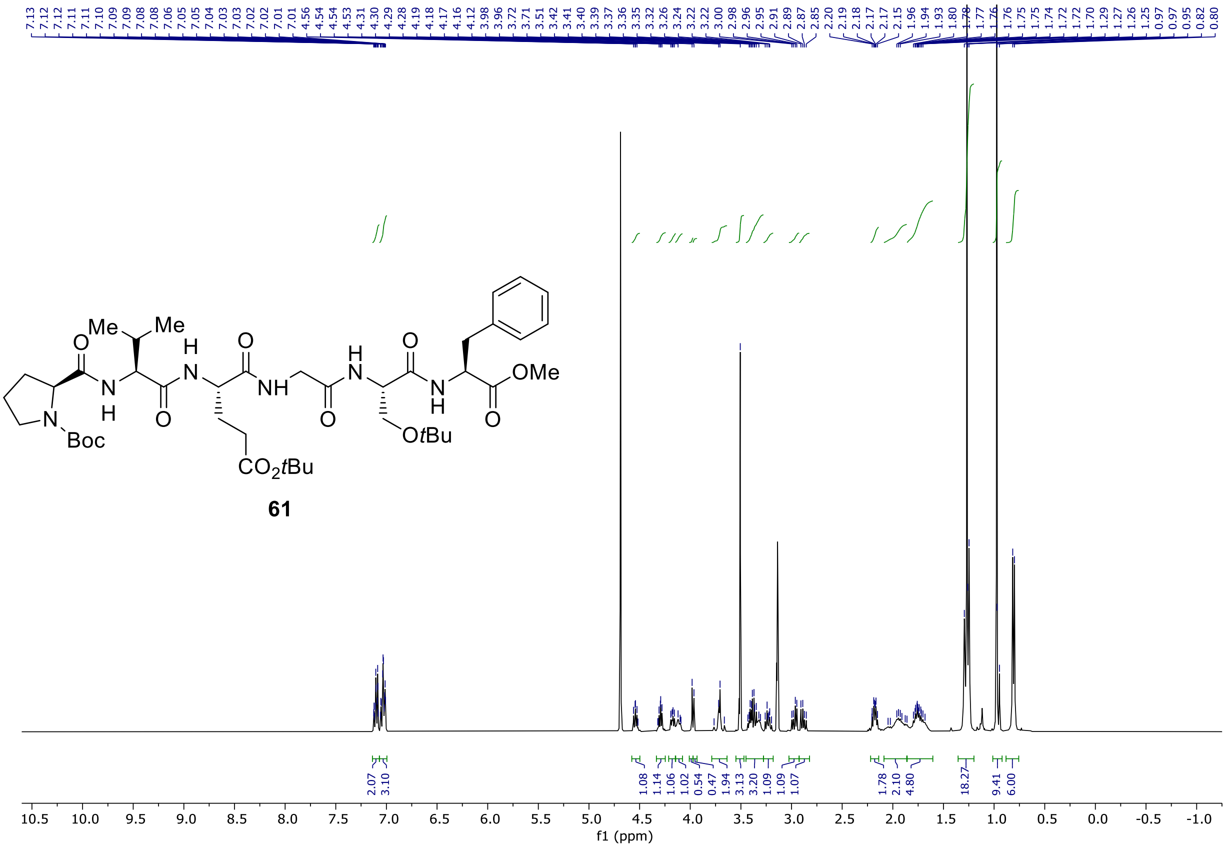

^{13}C NMR (101 MHz, MeOD-*d₄*, 298 K, complex mixture of rotamers) of compound **61**

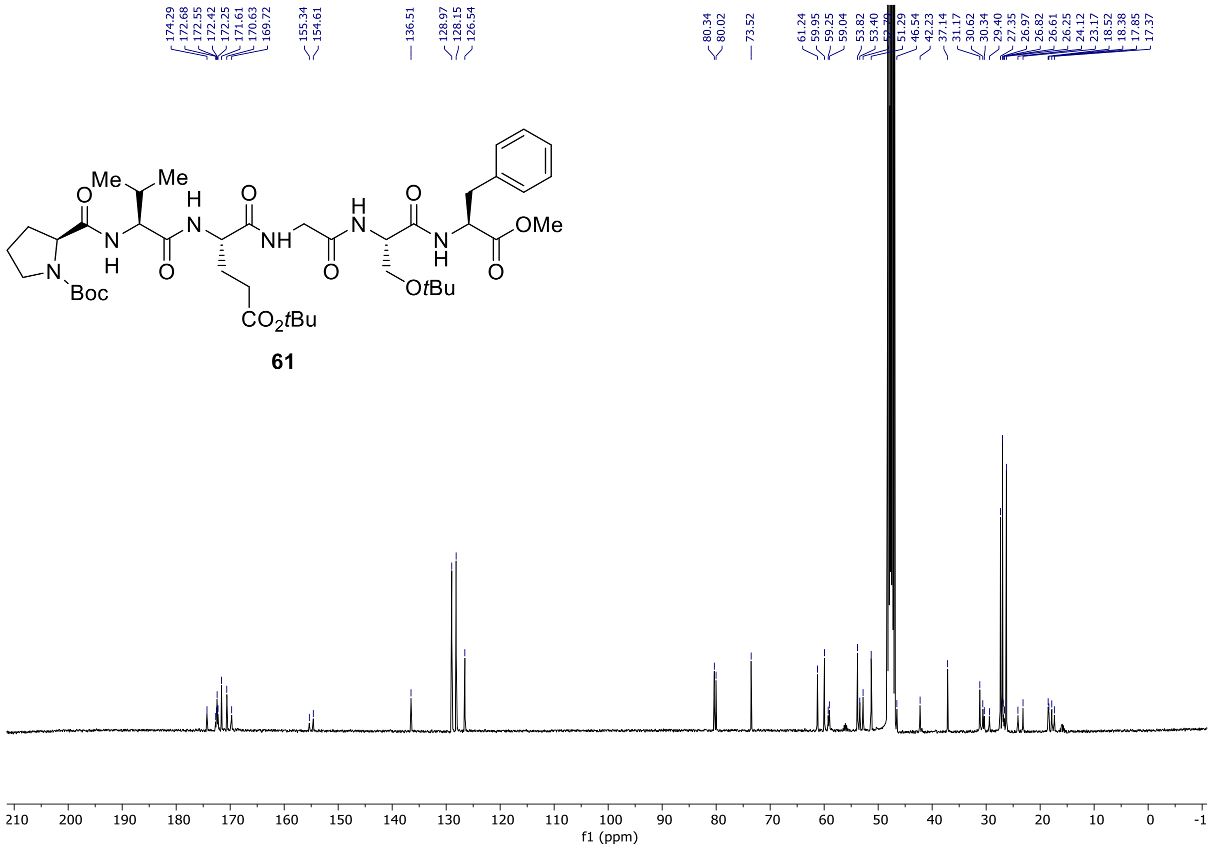

DEPT-135 (101 MHz, MeOD-d_4, 298 K, complex mixture of rotamers) of compound **61**

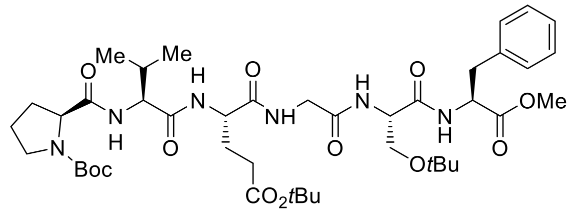

61

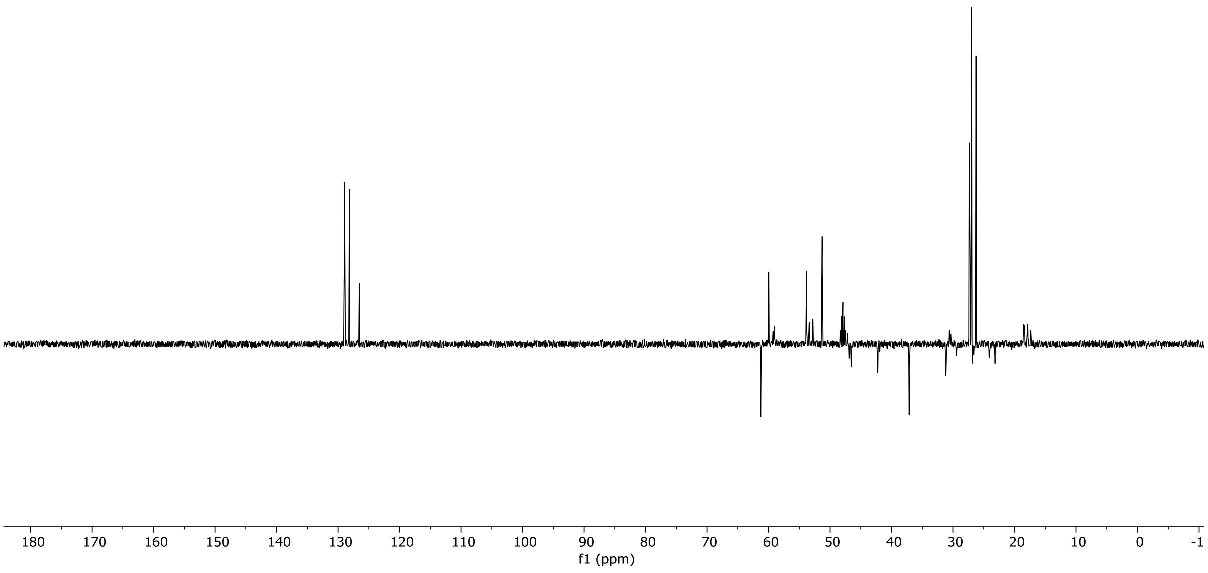

^{1}H NMR (400 MHz, $CDCl_3$, 298 K, mixture of two diastereoisomers) of compound **4**

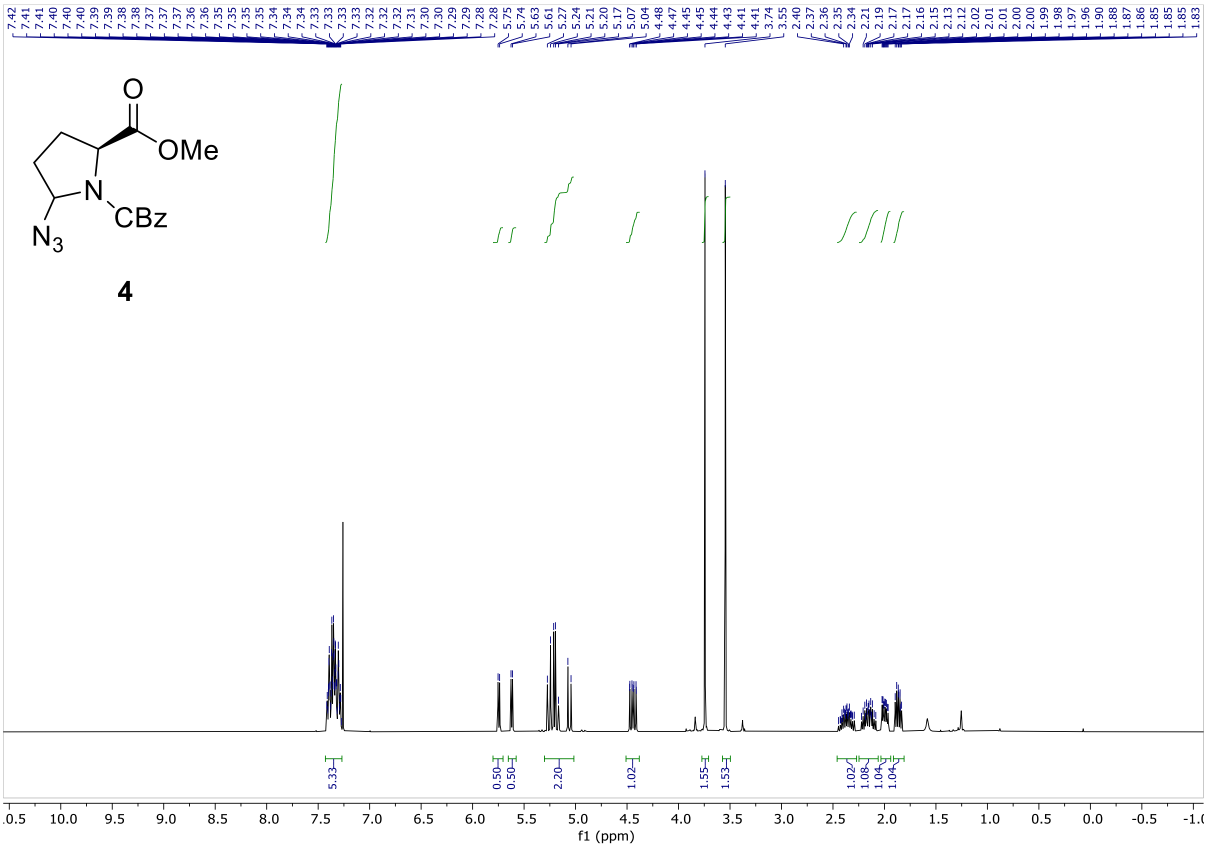

^{13}C NMR (101 MHz, $CDCl_3$, 298 K, mixture of two diastereoisomers) of compound **4**

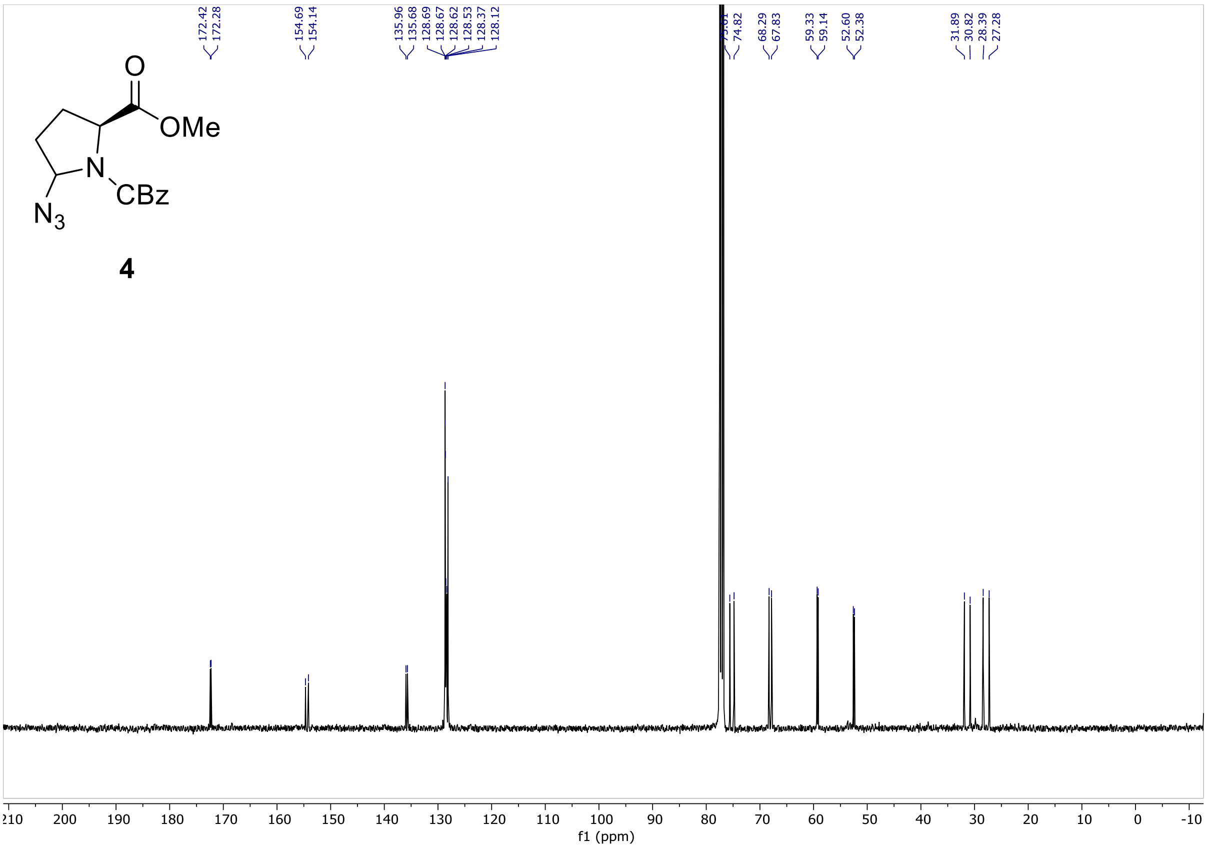

DEPT-135 (101 MHz, $CDCl_3$, 298 K, mixture of two diastereoisomers) of compound **4**

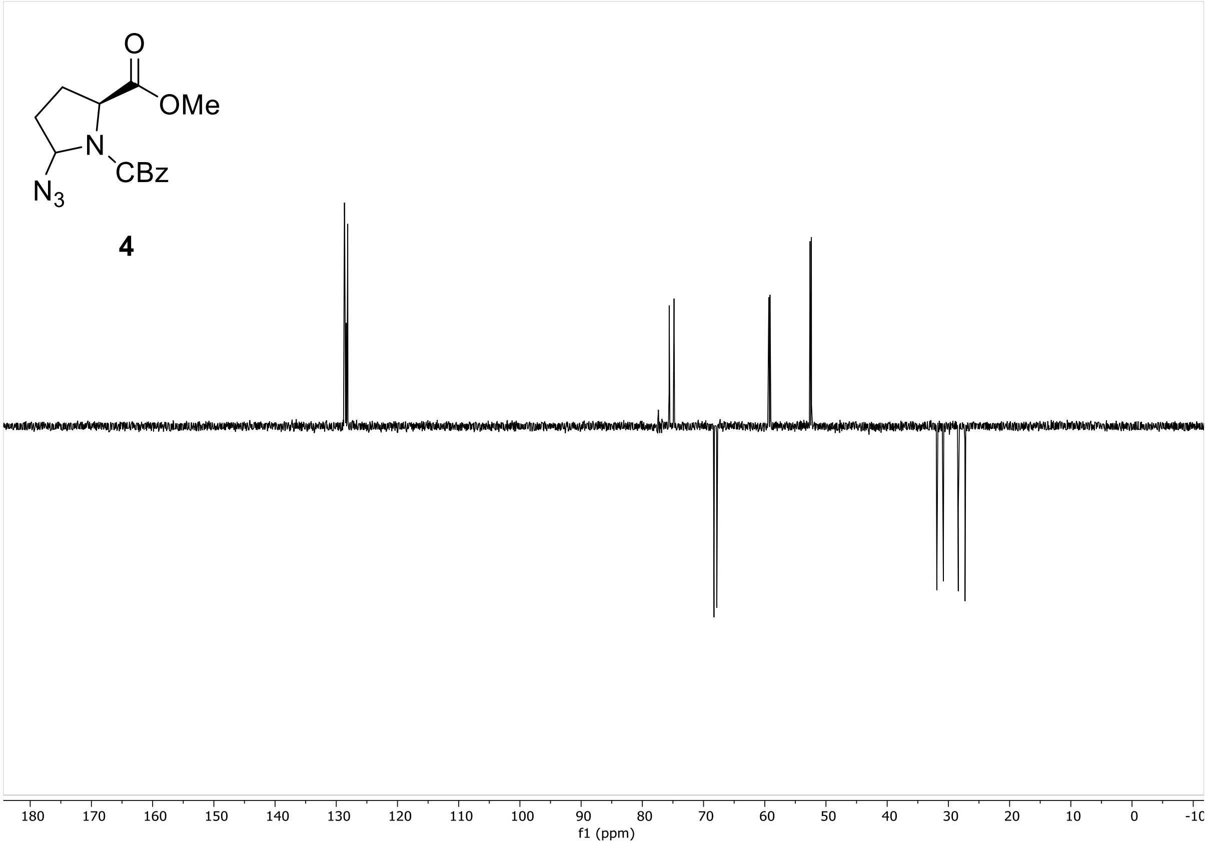

^{1}H NMR (400 MHz, $CDCl_3$, 298 K, mixture of two diastereoisomers) of compound **7**

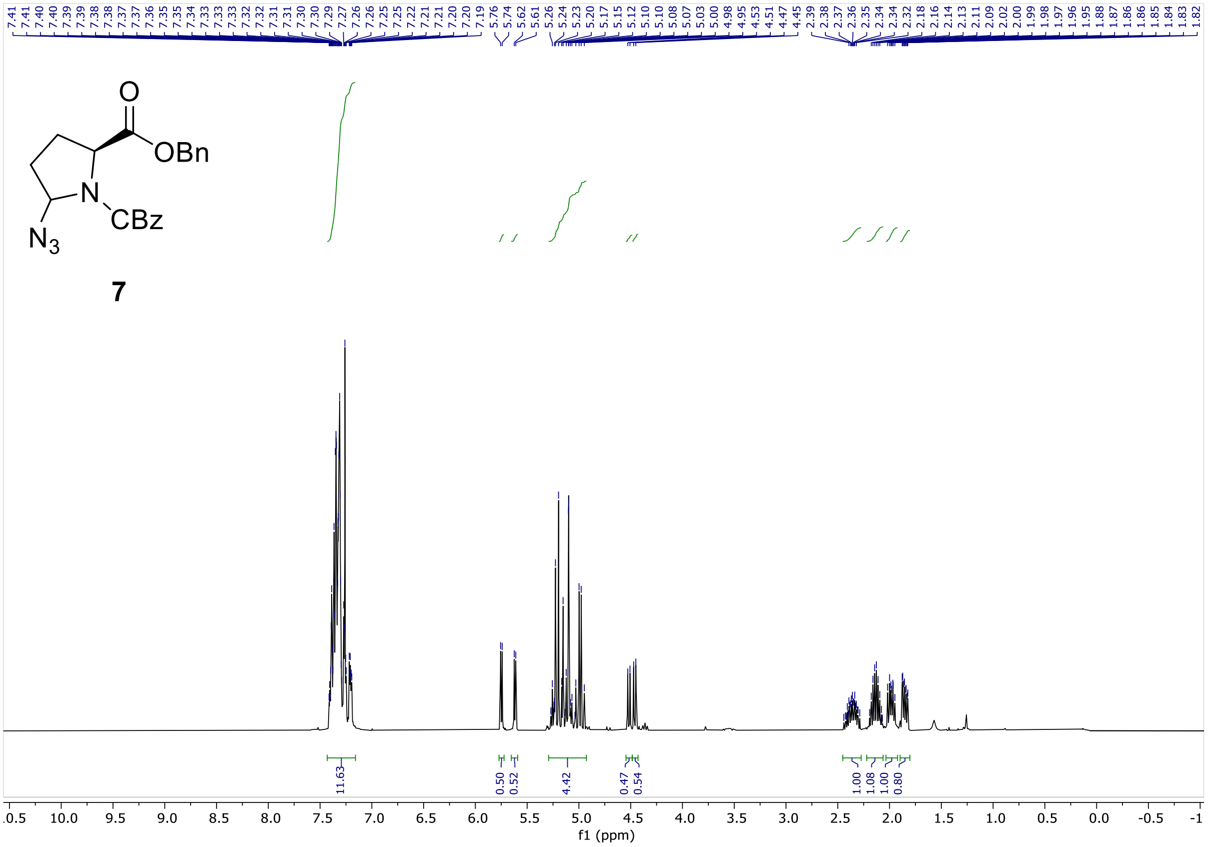

^{13}C NMR (101 MHz, $CDCl_3$, 298 K, mixture of two diastereoisomers) of compound **7**

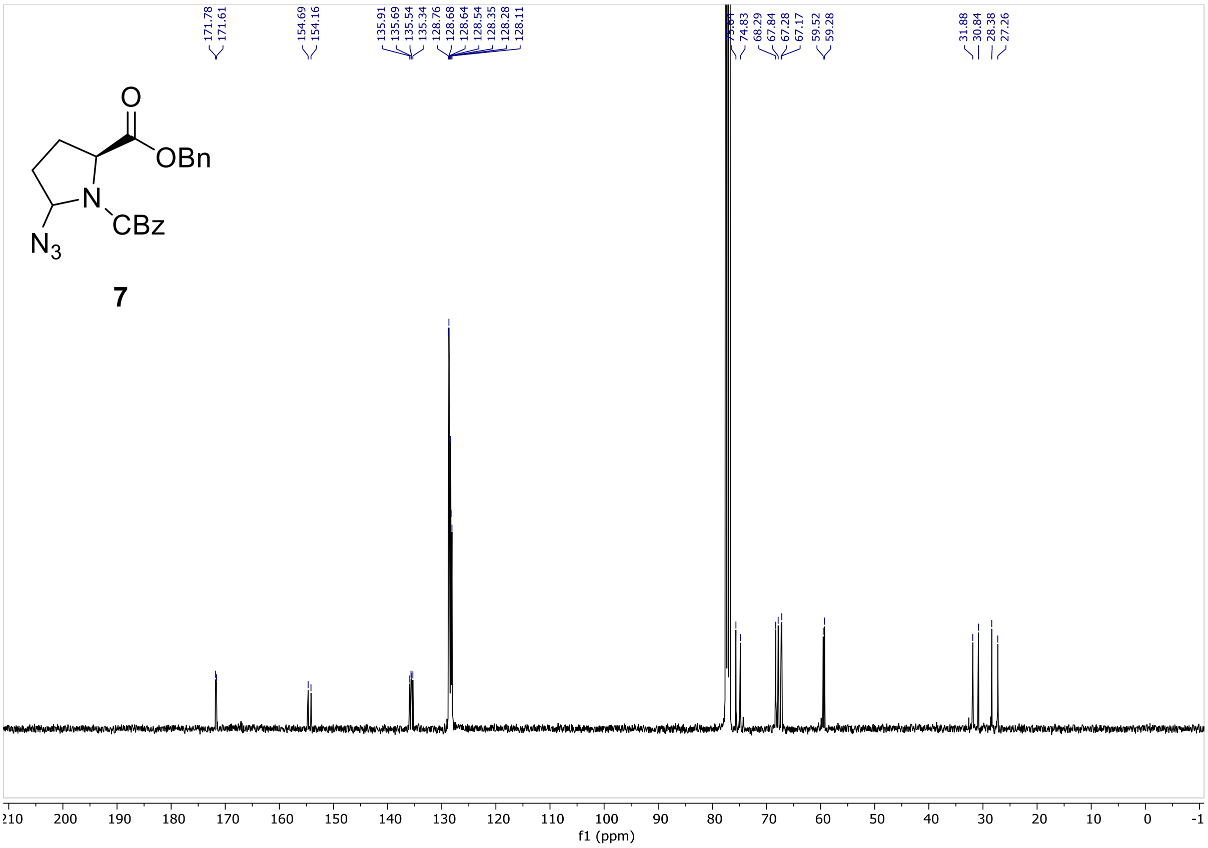

DEPT (101 MHz, $CDCl_3$, 298 K, mixture of two diastereoisomers) of compound **7**

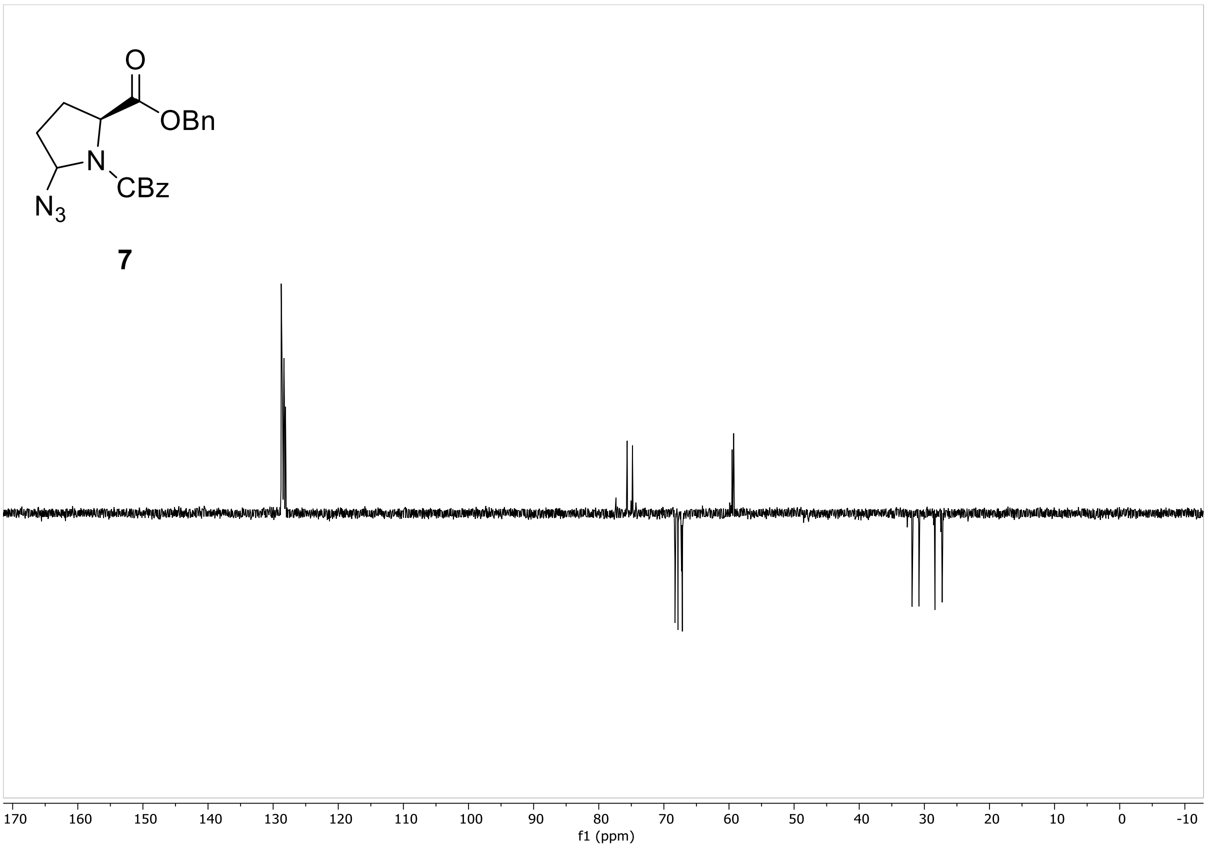

^{1}H NMR (400 MHz, $CDCl_3$, 298 K, mixture of two diastereoisomers) of compound **8a**

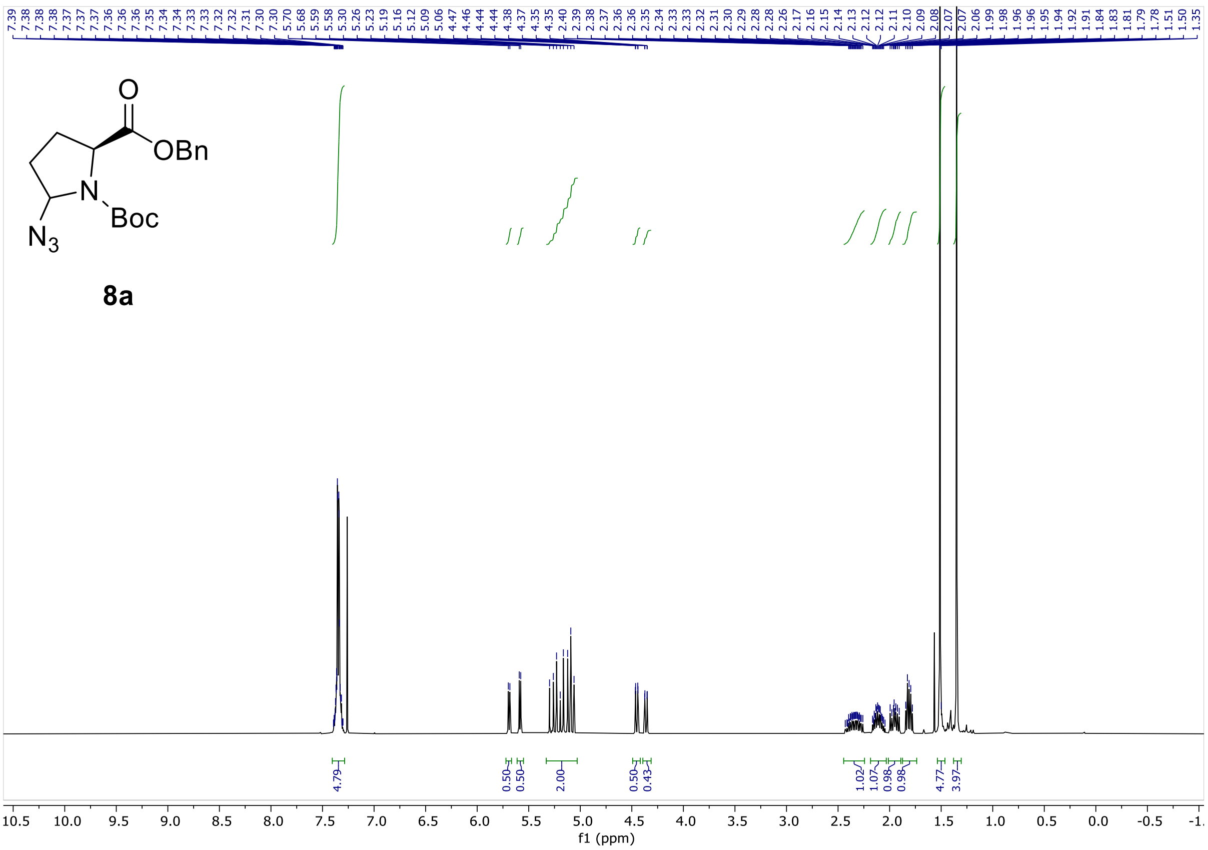

^{13}C NMR (101 MHz, $CDCl_3$, 298 K, mixture of diastereoisomers) of compound **8a**

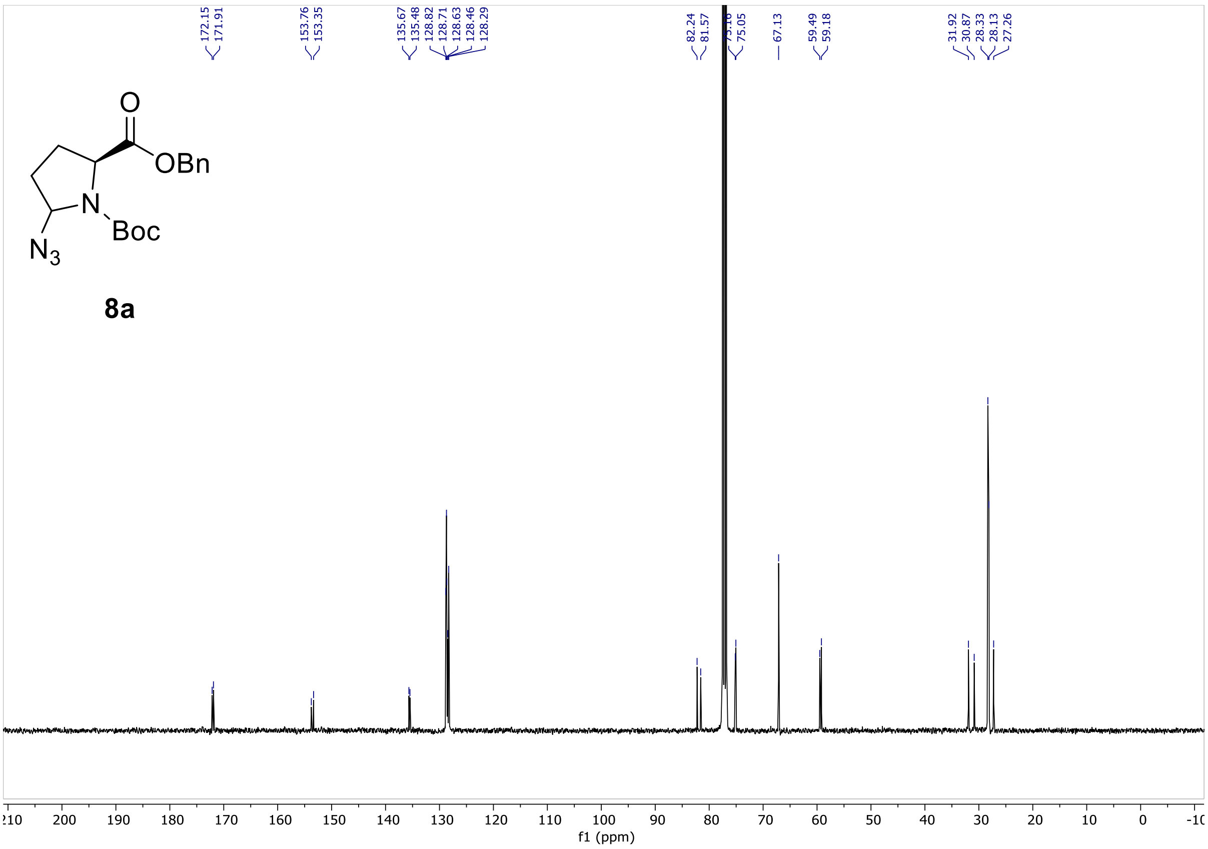

NMR (101 MHz, $CDCl_3$, 298 K, mixture of two diastereoisomers) of compound **8b**

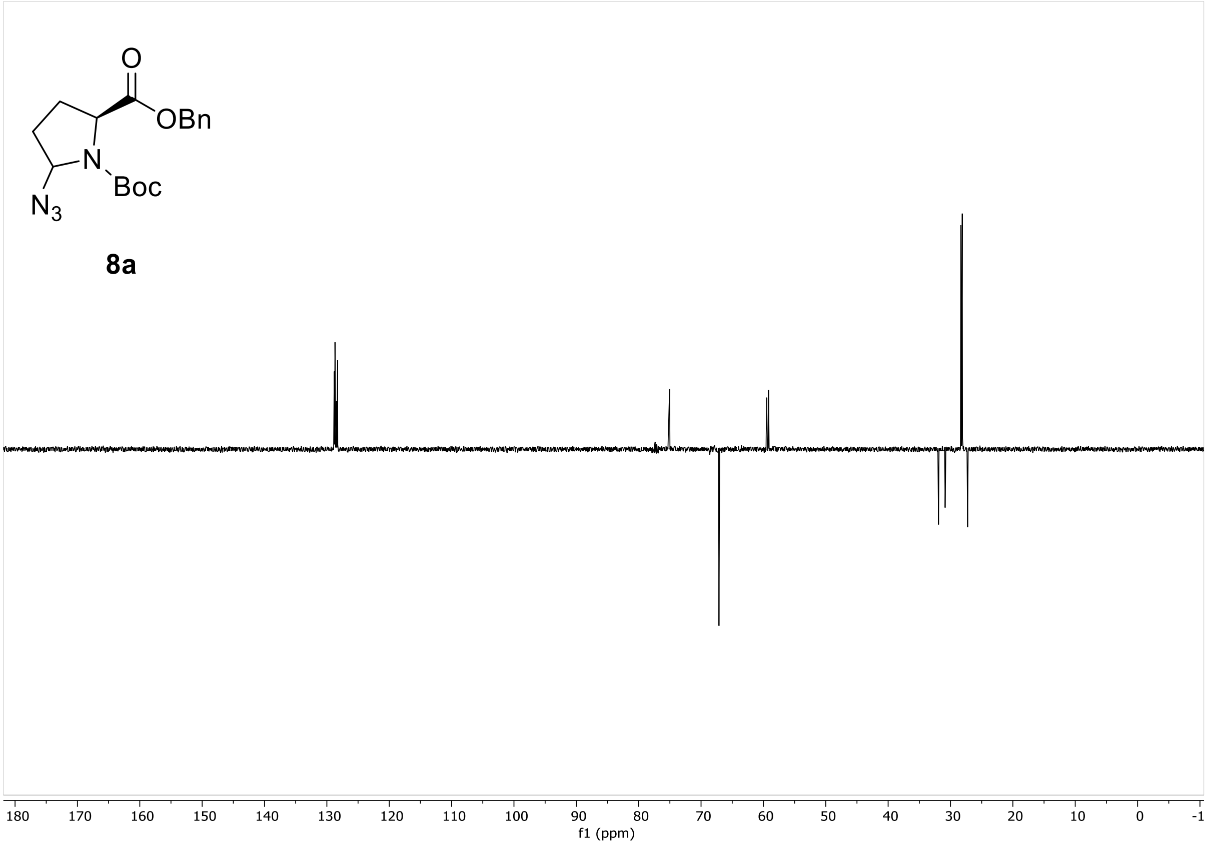

^{1}H **NMR** (400 MHz, $CDCl_3$, 298 K, mixture of two diastereoisomers) of compound **8b**

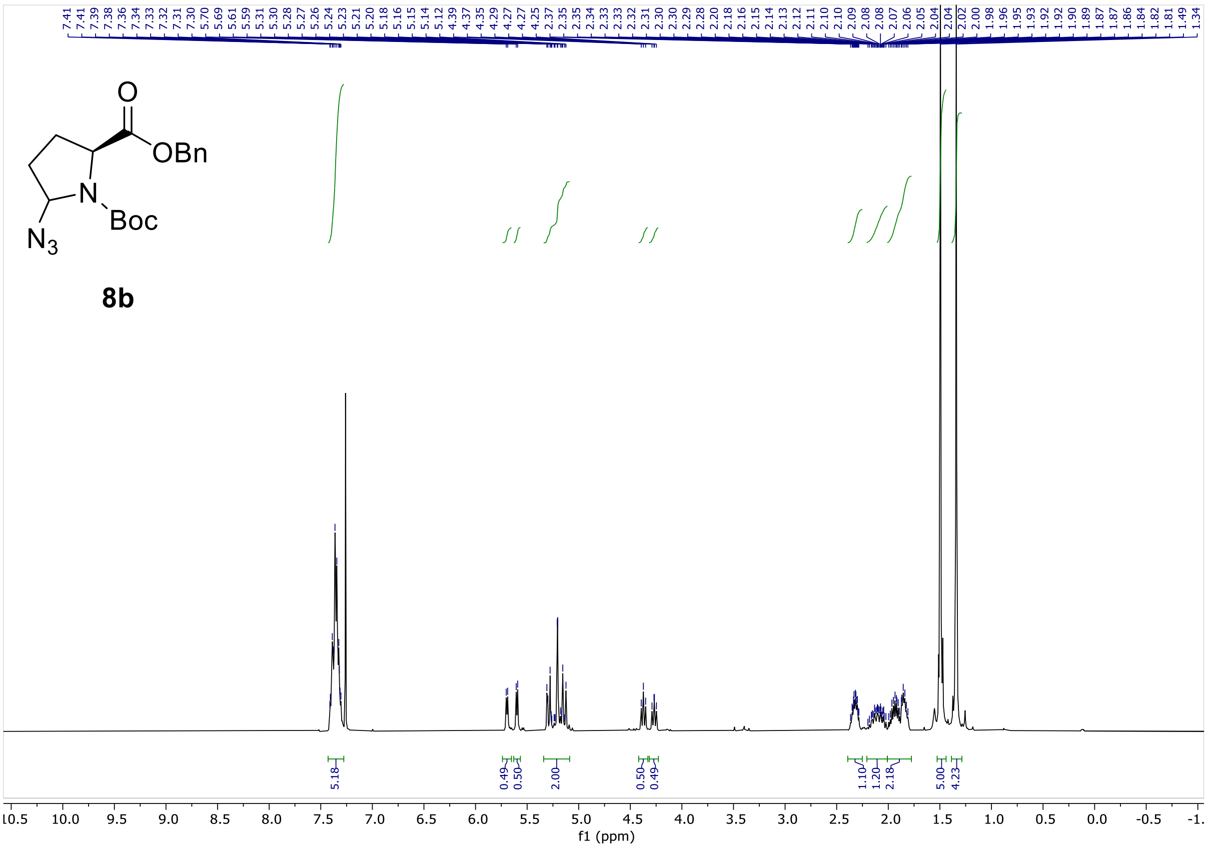

^{13}C **NMR** (101 MHz, $CDCl_3$, 298 K, mixture of two diastereoisomers) of compound **8b**

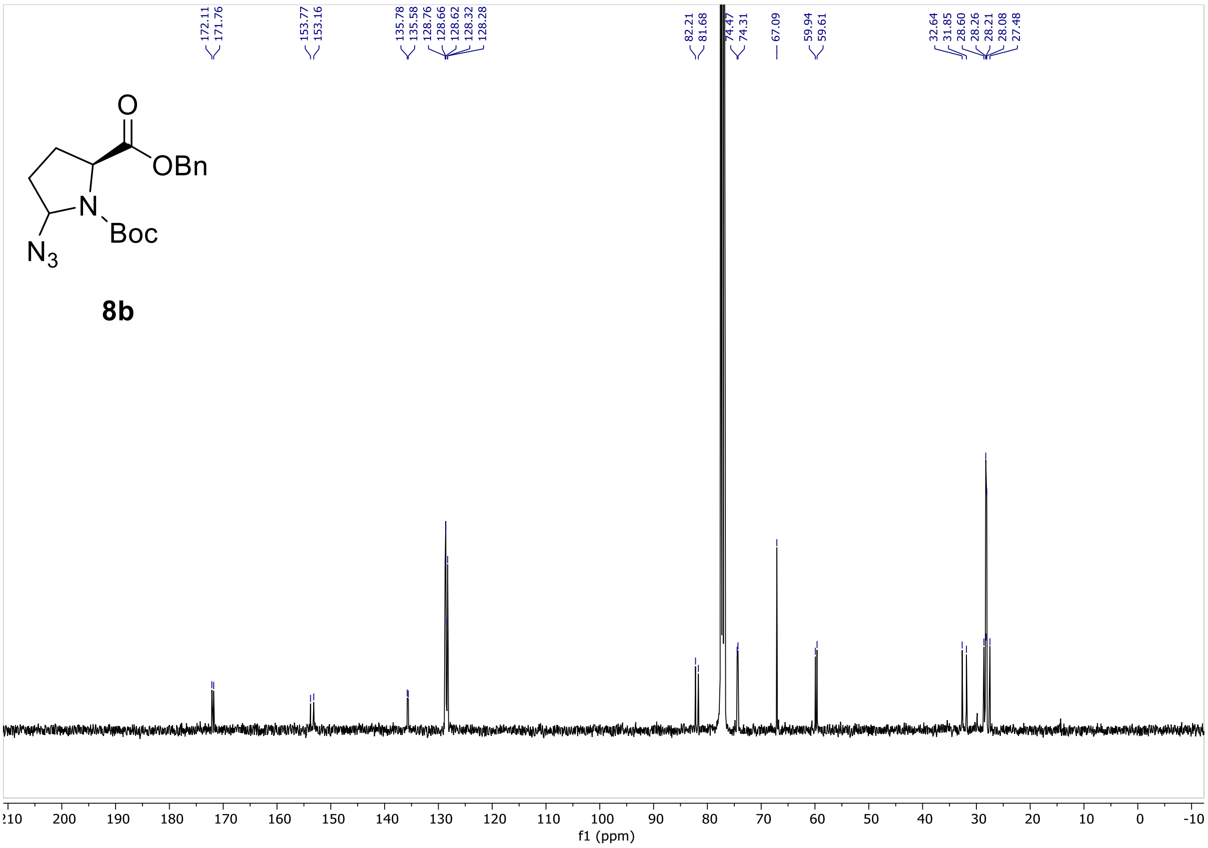

DEPT-135 (101 MHz, $CDCl_3$, 298 K, mixture of two diastereoisomers) of compound **8b**

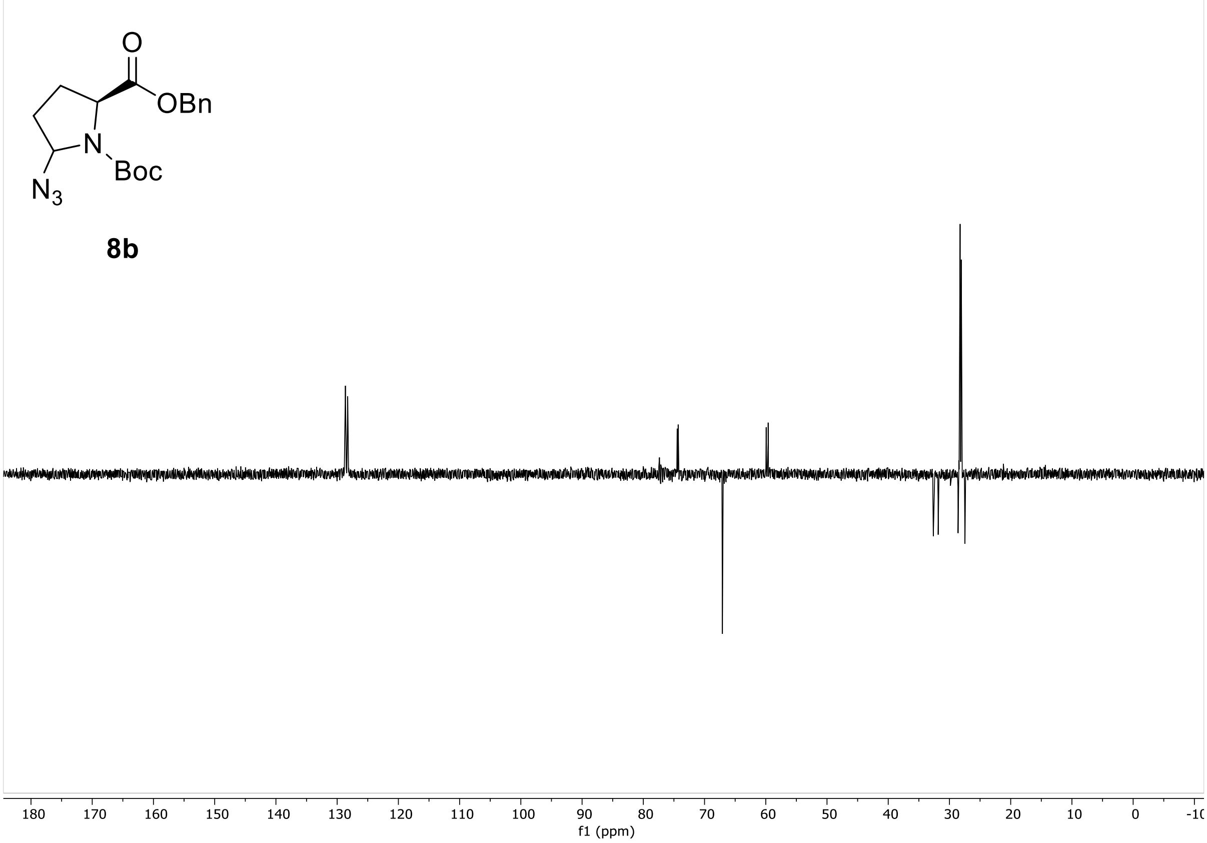

^{1}H NMR (400 MHz, CDCl$_3$, 298 K, complex mixture of diastereoisomers and rotamers) of **9**

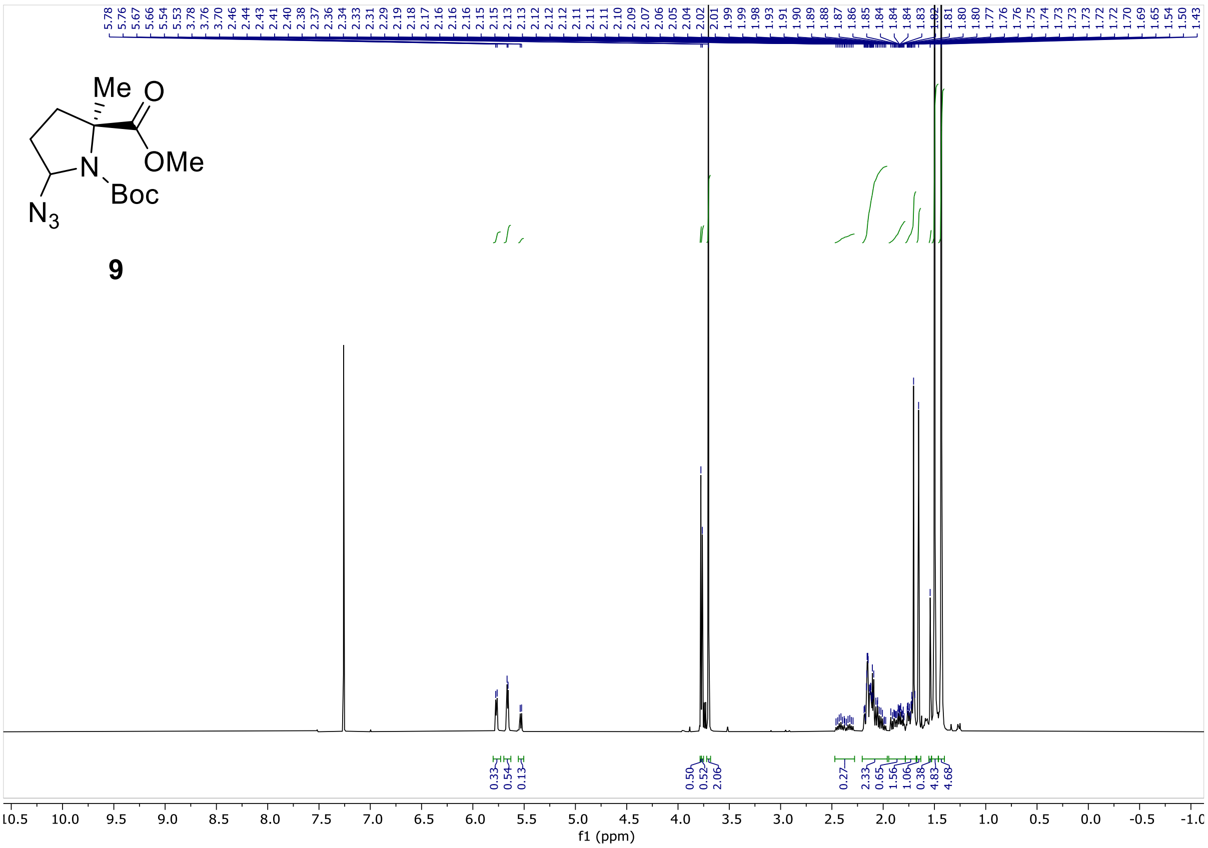

^{13}C NMR (101 MHz, CDCl$_3$, 298 K, complexe mixture of diastereoisomers and rotamers) of **9**

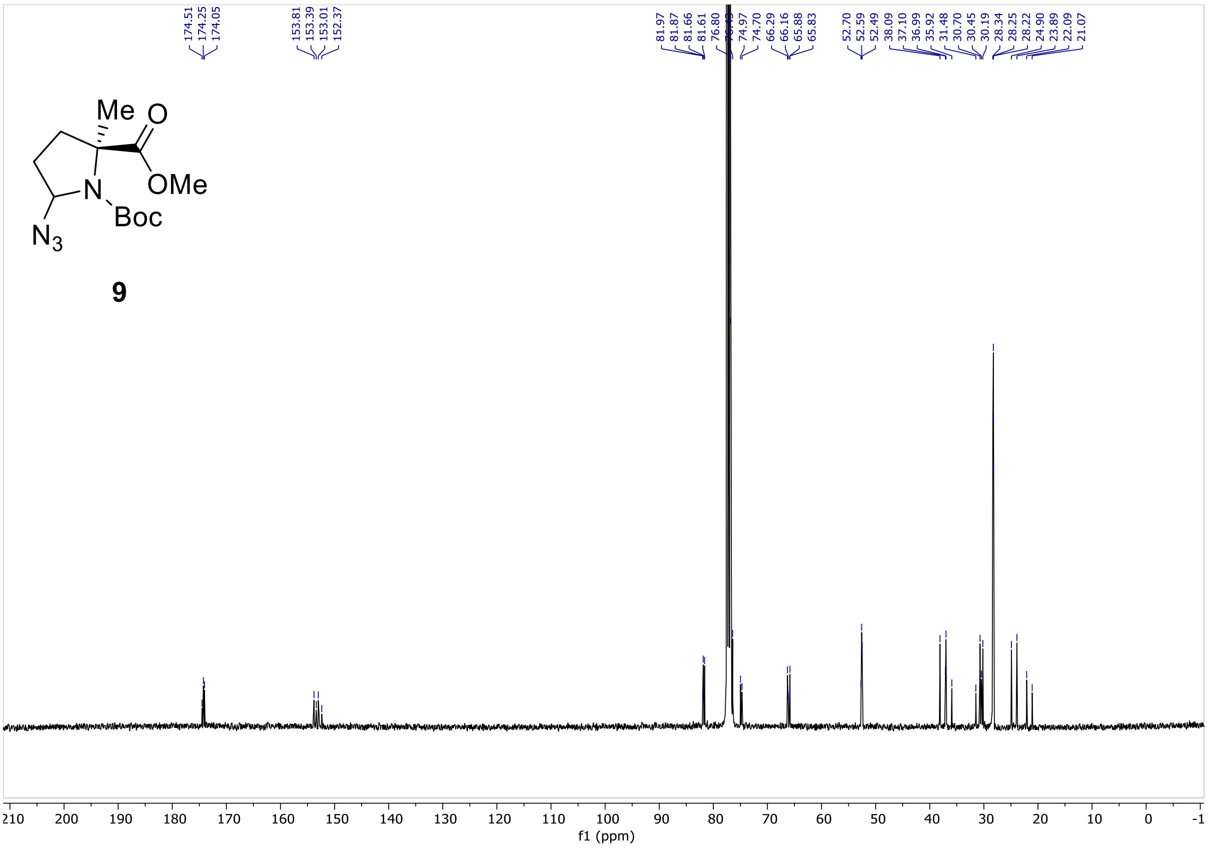

DEPT-135 (101 MHz, CDCl₃, 298 K, complexe mixture of diastereoisomers and rotamers) of **9**

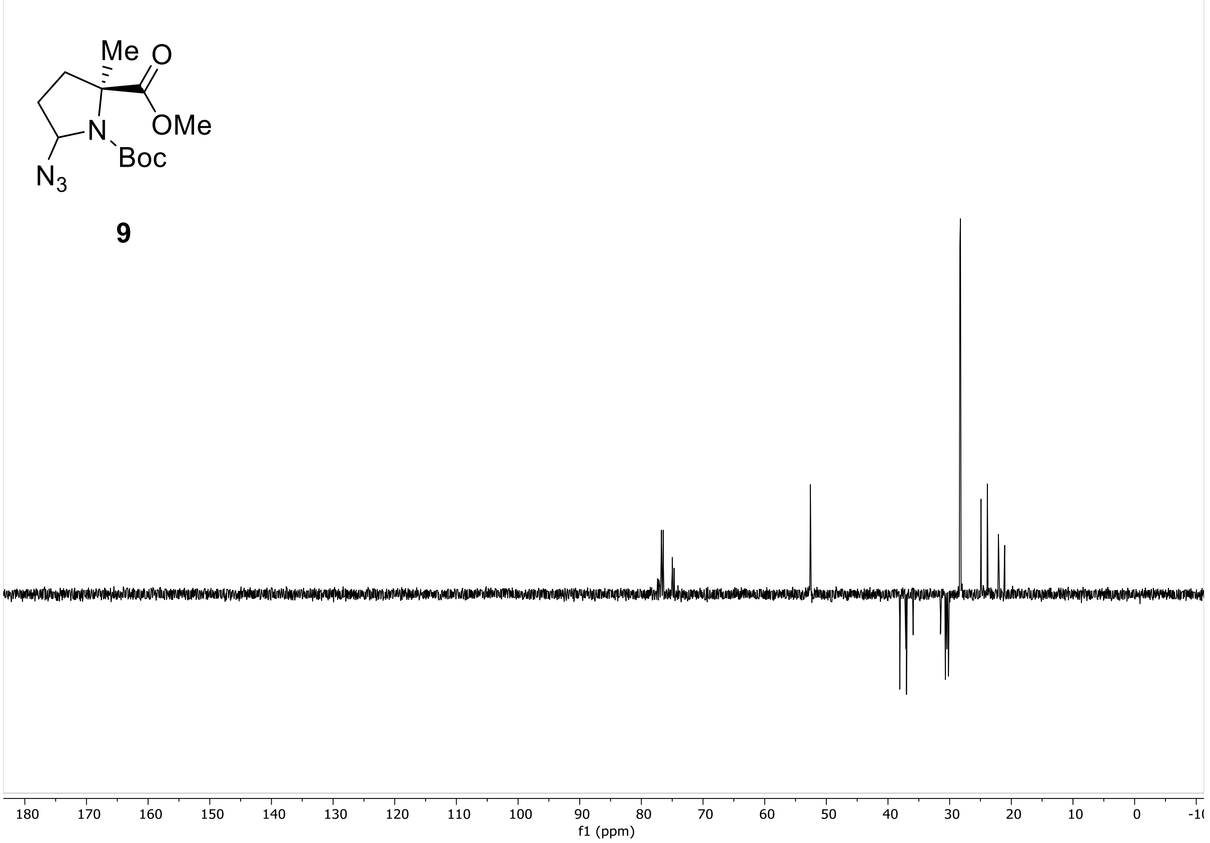

^{1}**H NMR** (400 MHz, MeOD-d_4, 278.2 K, complex mixture of diastereoisomers and rotamers) of **12**

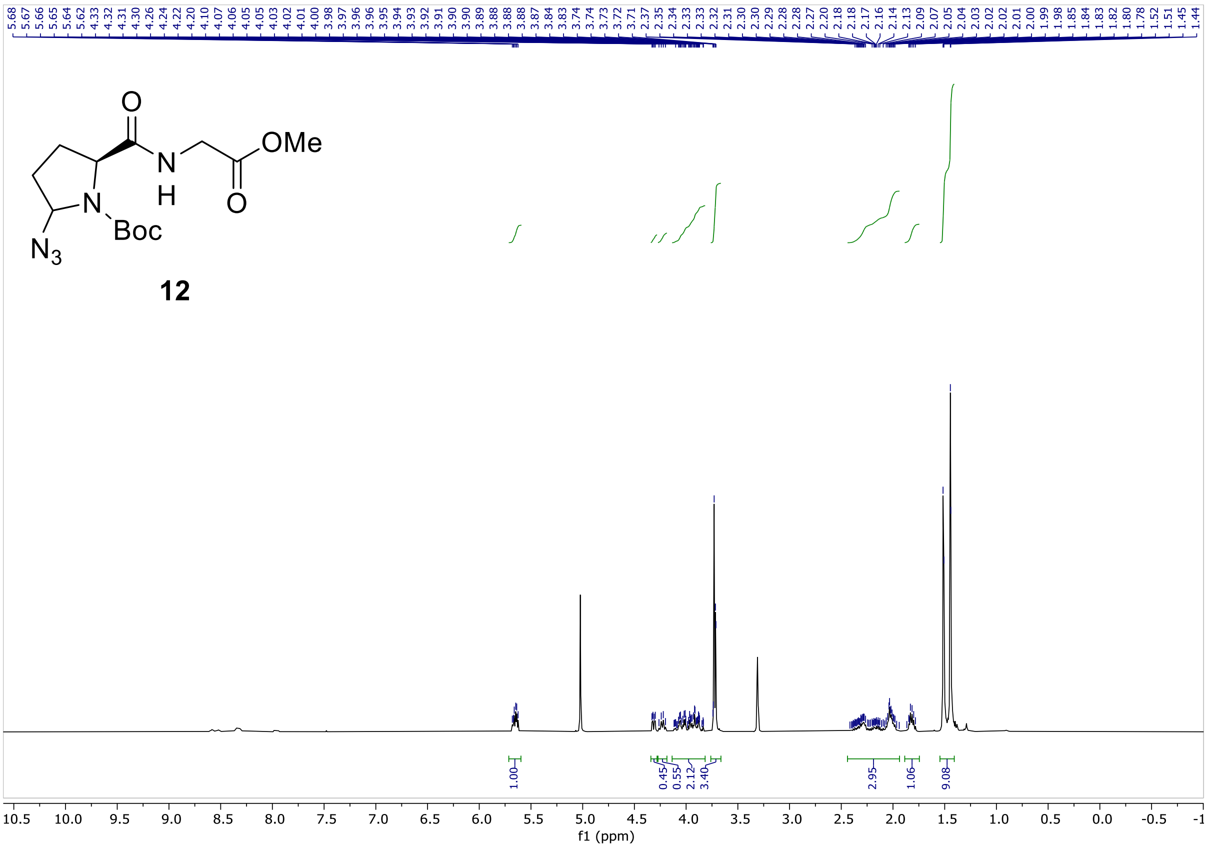

^{13}**C NMR** (101 MHz, MeOD-d_4, 278.2 K, complex mixture of diastereoisomers and rotamers) of **12**

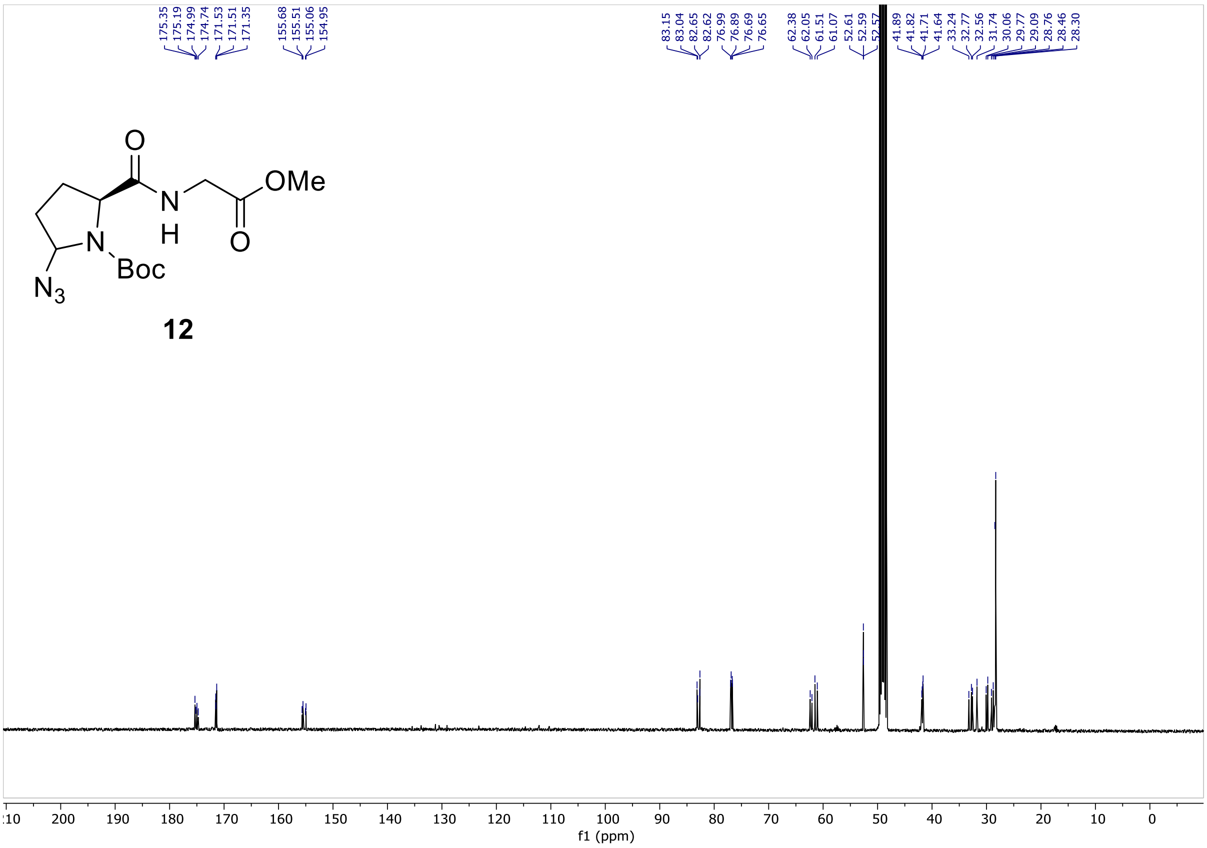

HSQC (MeOD-d_4, 278.2 K, complex mixture of diastereoisomers and rotamers) of **12**

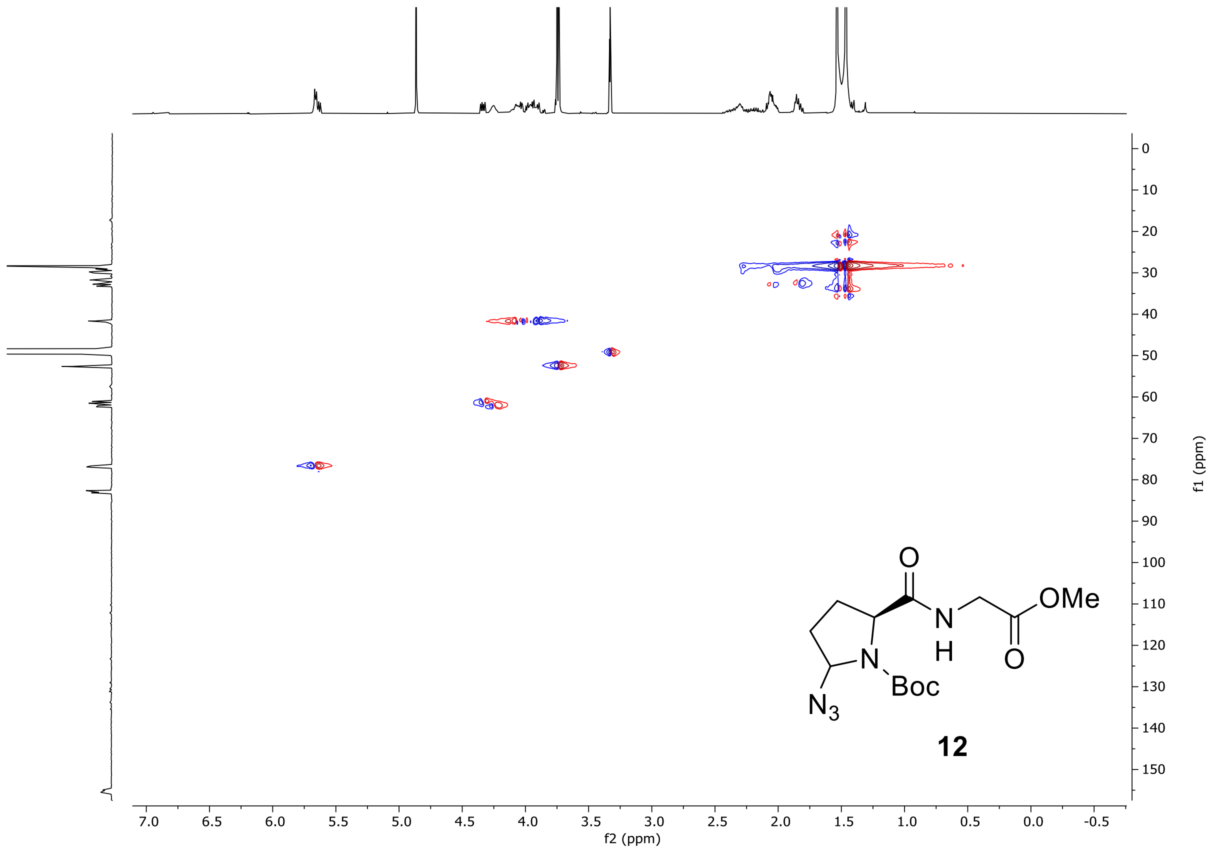

^{1}**H NMR** (400 MHz, $CDCl_3$, 298 K, mixture of two diastereoisomers) of compound **14**

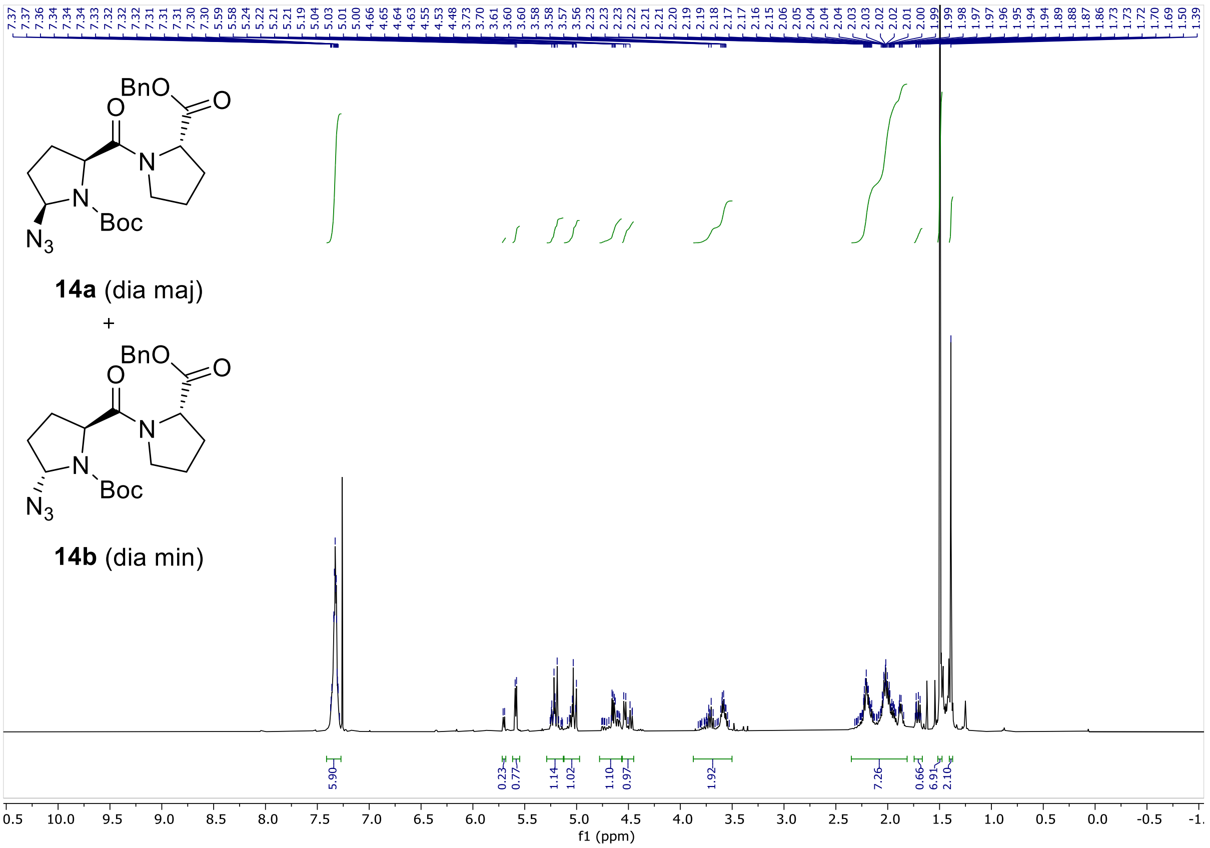

^{13}**C NMR** (101 MHz, $CDCl_3$, 298 K, mixture of two diastereoisomers) of compound **14**

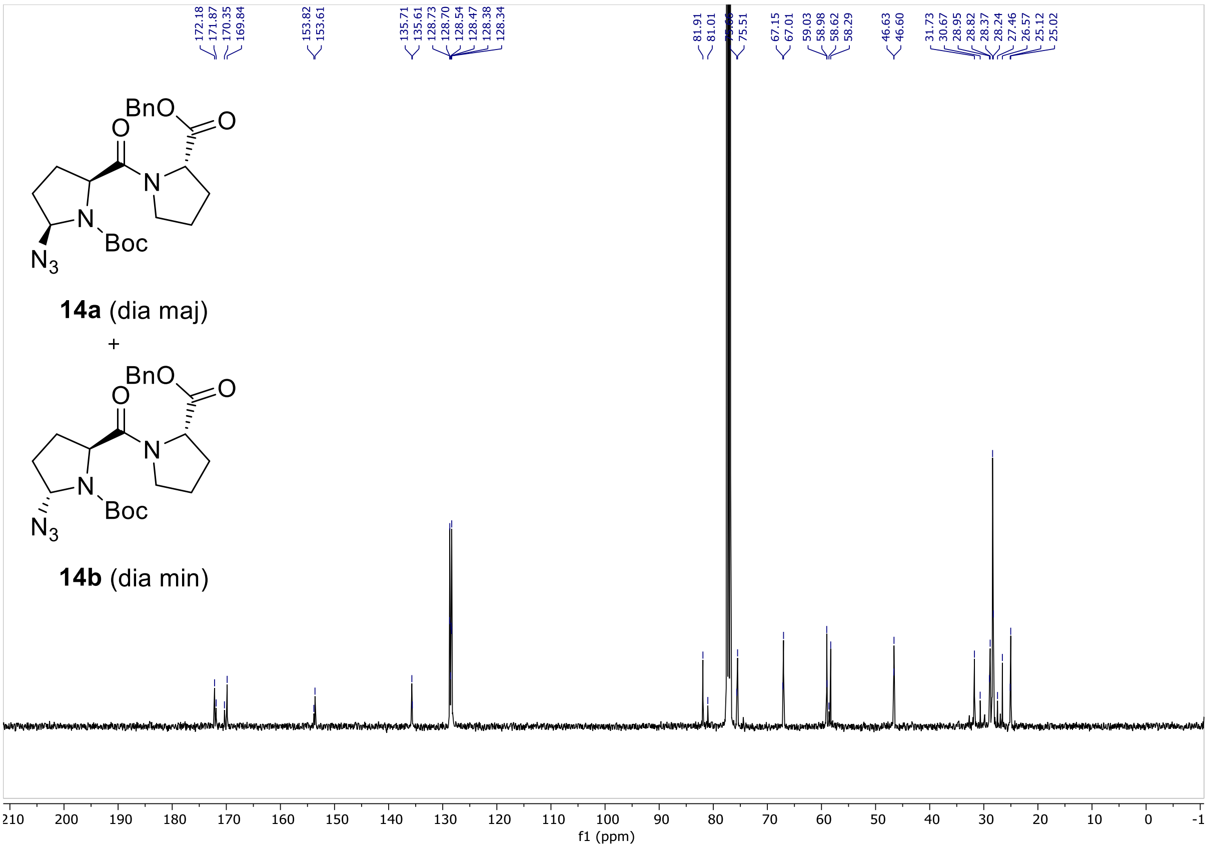

DEPT-135(101 MHz, $CDCl_3$, 298 K, mixture of two diastereoisomers) of compound **14**

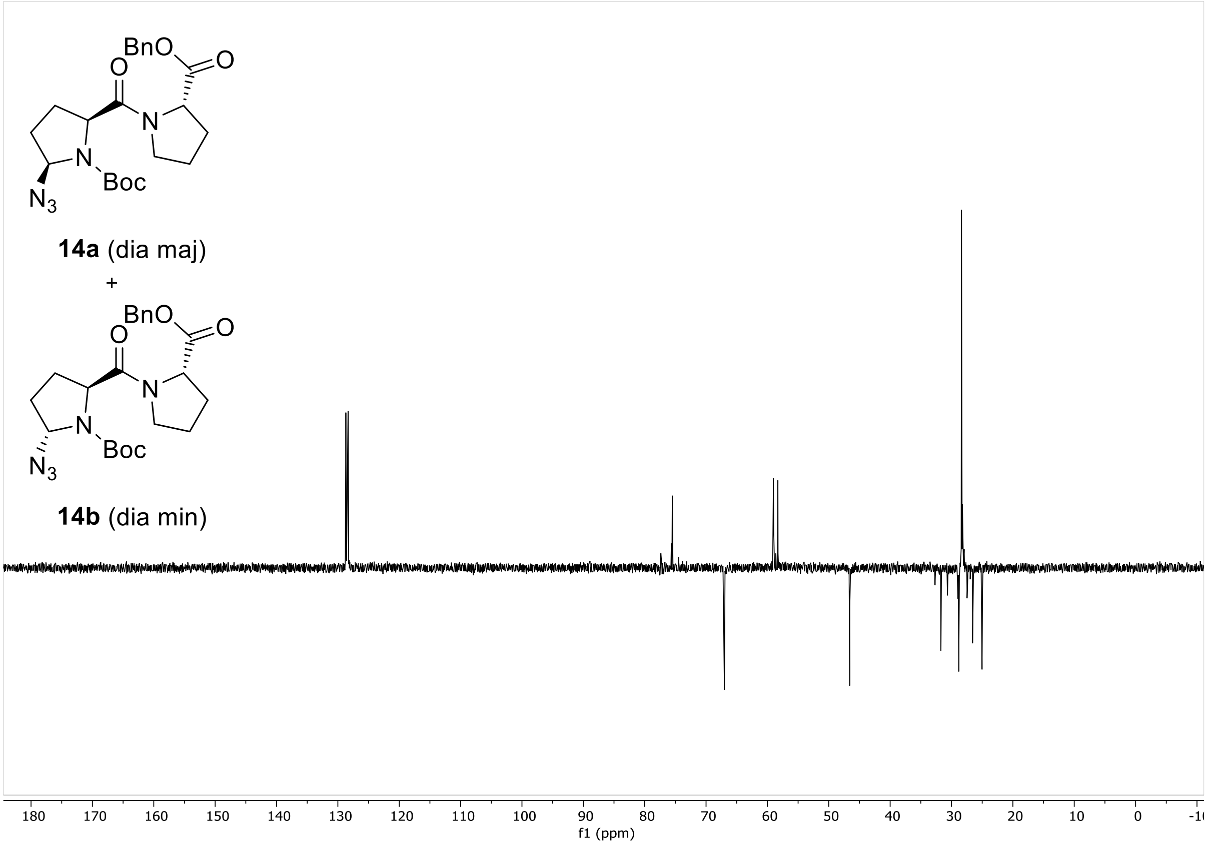

NOESY (400 MHz, $CDCl_3$, 298 K, mixture of two diastereoisomers) of compound **14**

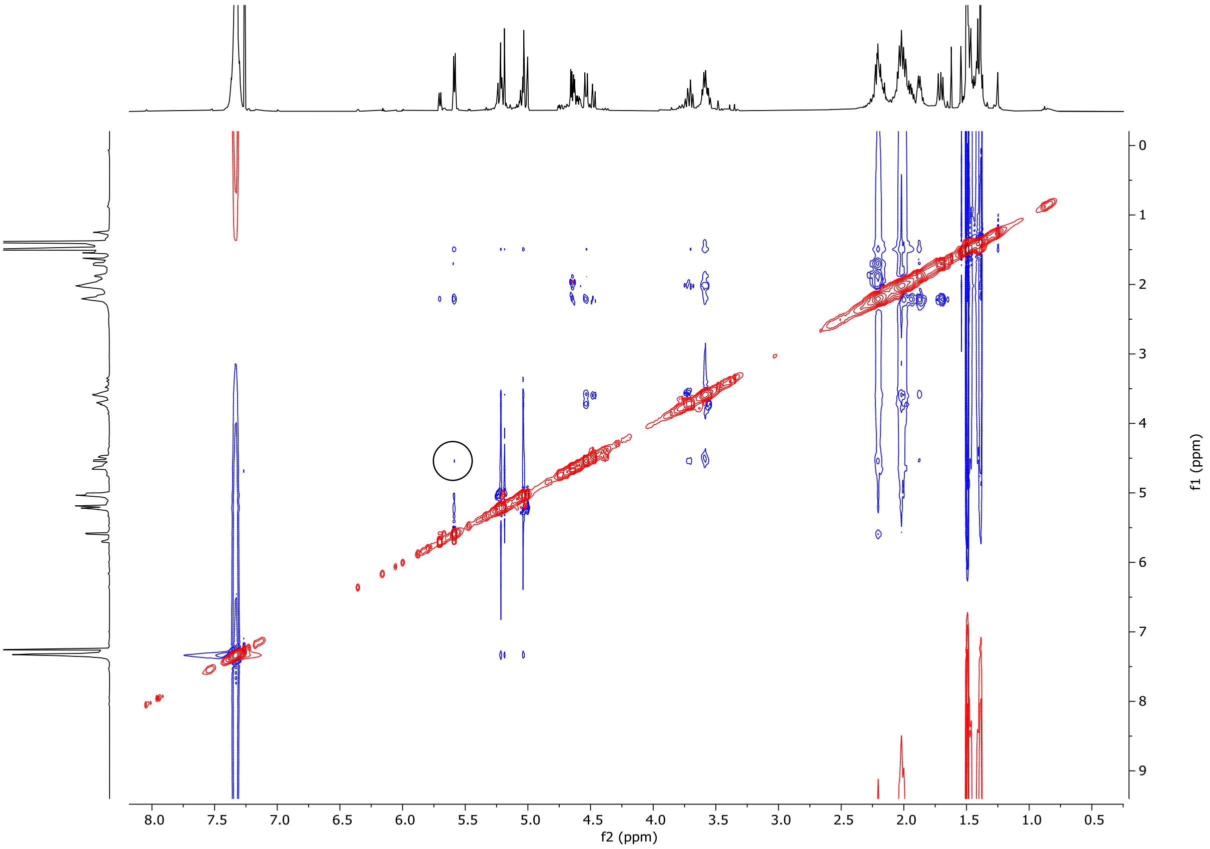

¹H NMR (400 MHz, $CDCl_3$, 298 K) of compound **15**

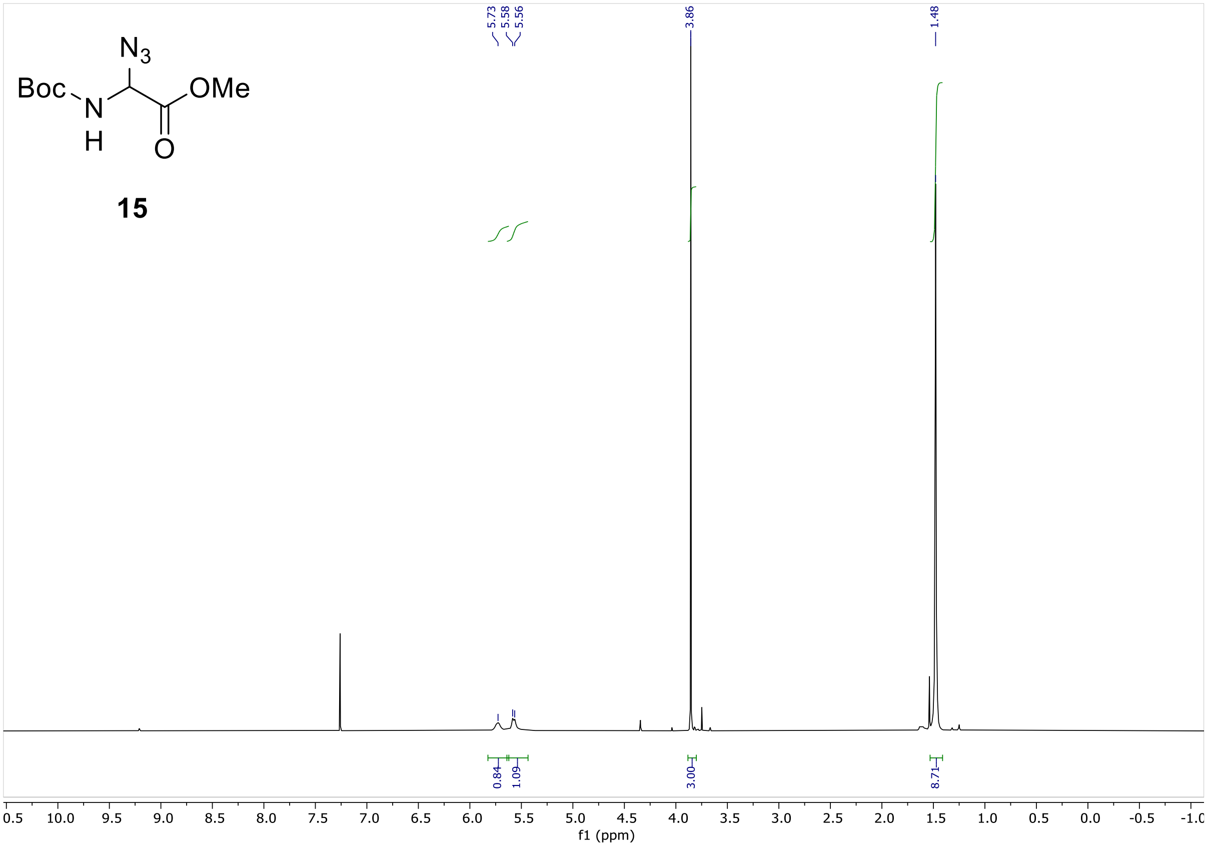

¹³C NMR (101 MHz, $CDCl_3$, 298 K) of compound **15**

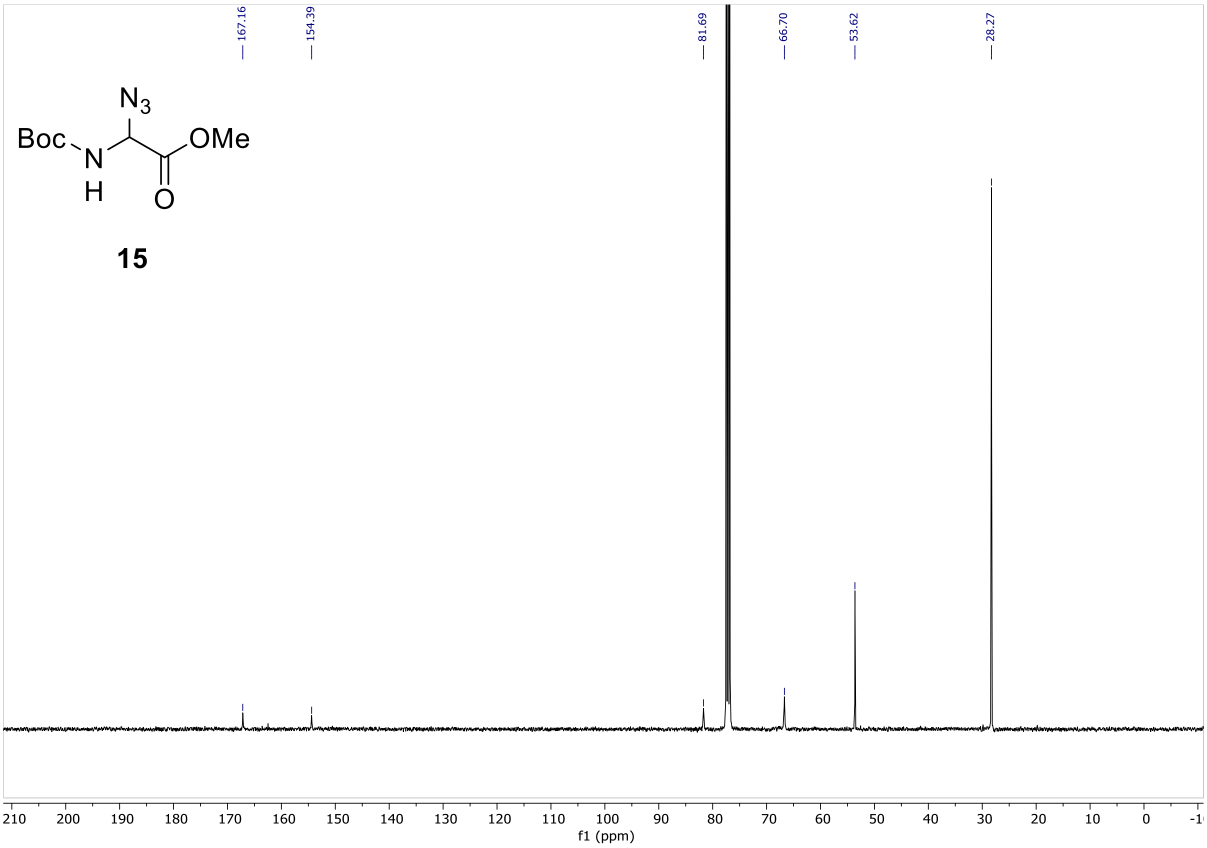

DEPT-135 (101 MHz, $CDCl_3$, 298 K) of compound **15**

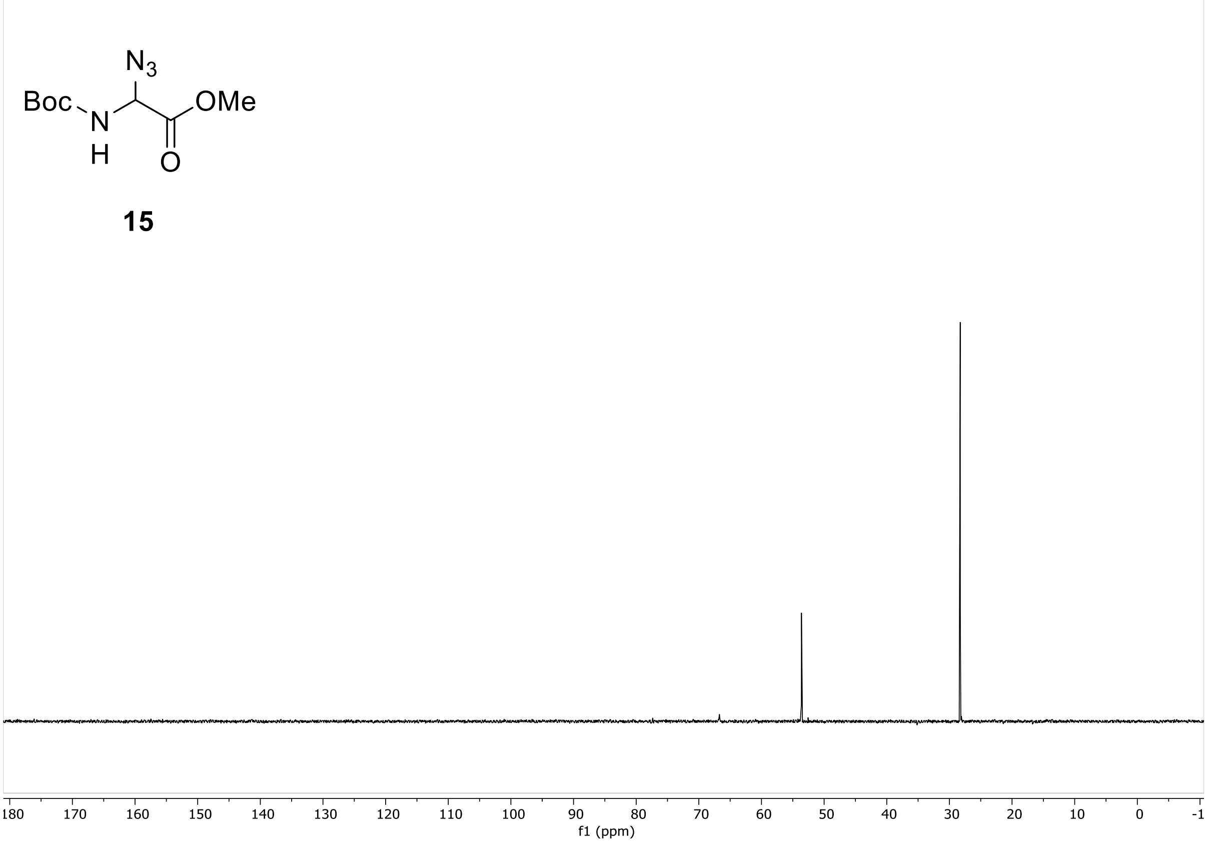

^{1}H NMR (400 MHz, $CDCl_3$, 298 K) of compound **16**

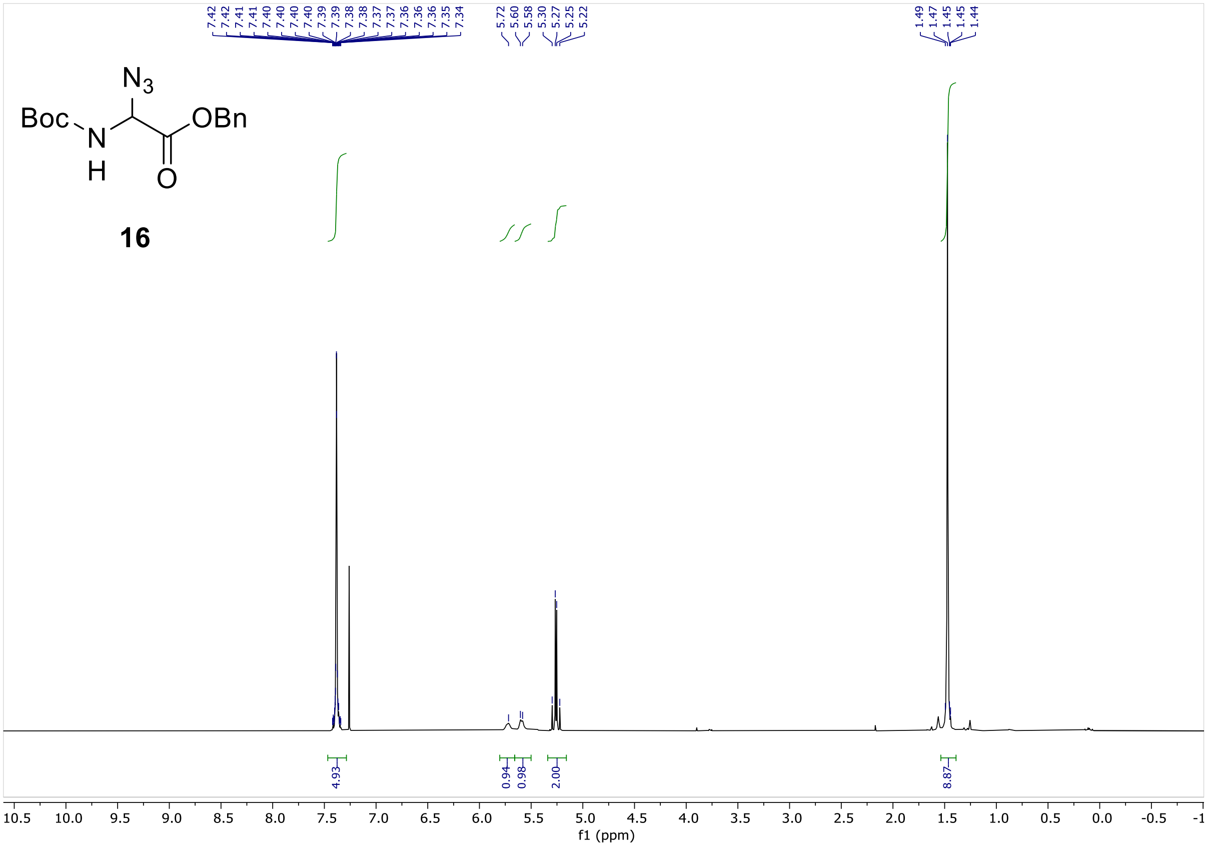

^{13}C NMR (101 MHz, $CDCl_3$, 298 K) of compound **16**

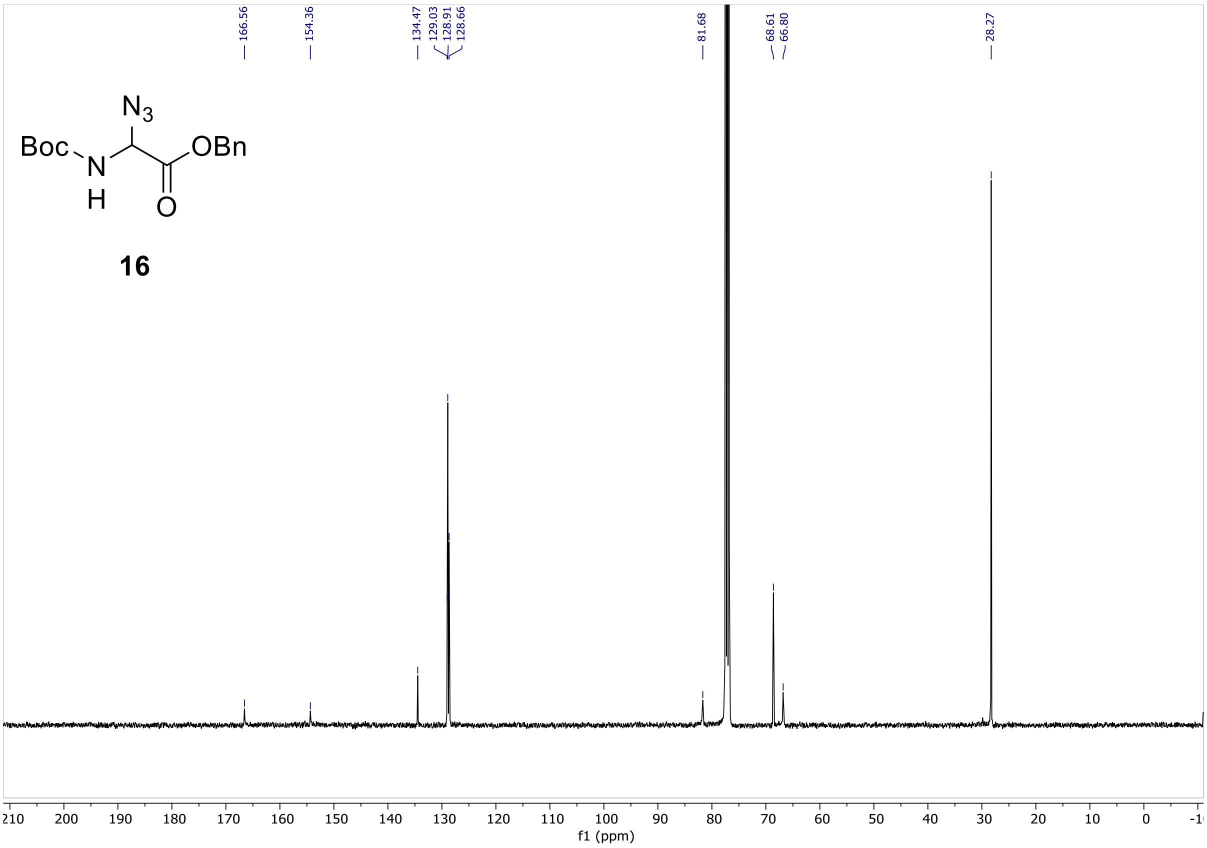

DEPT-135 (101 MHz, $CDCl_3$, 298 K) of compound **16**

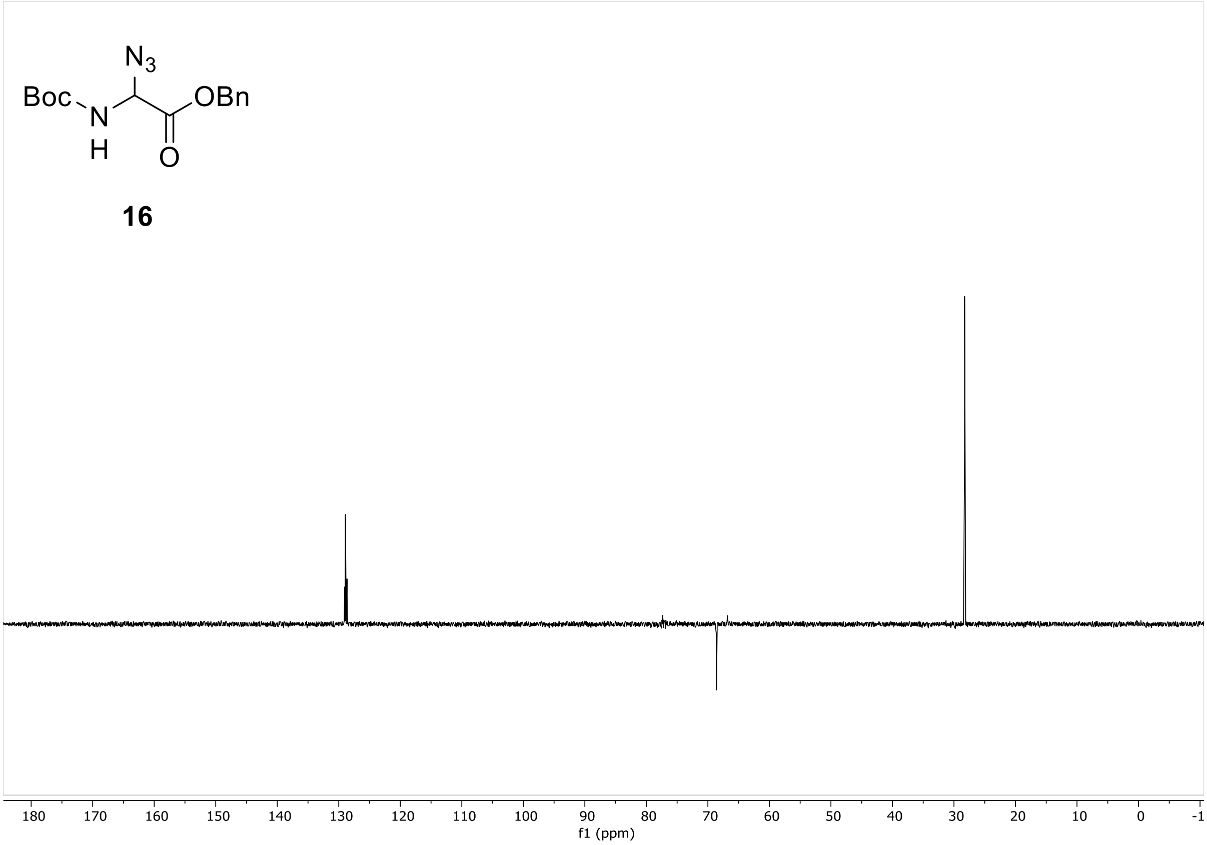

^{1}H NMR (400 MHz, MeOD-d_4, 298 K) of compound **17**

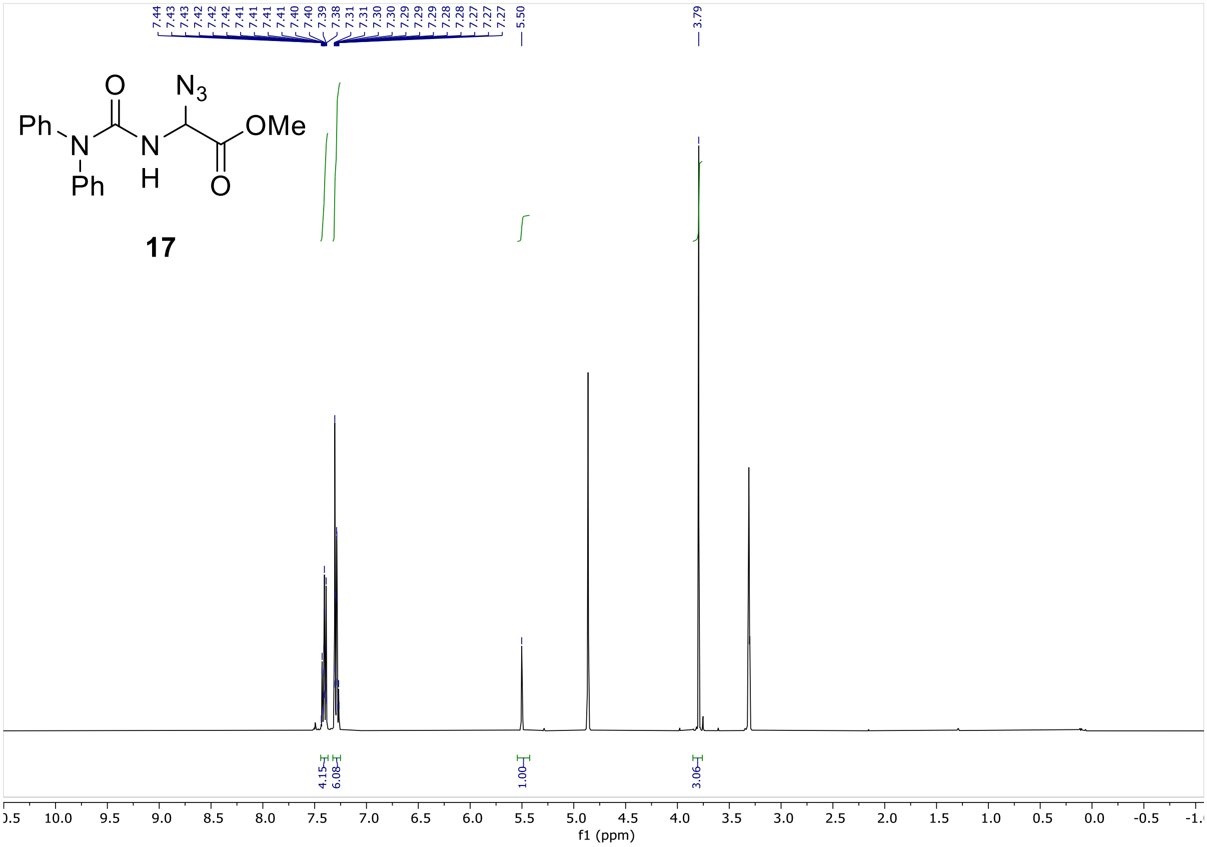

^{13}C NMR (101 MHz, MeOD-d_4, 298 K) of compound **17**

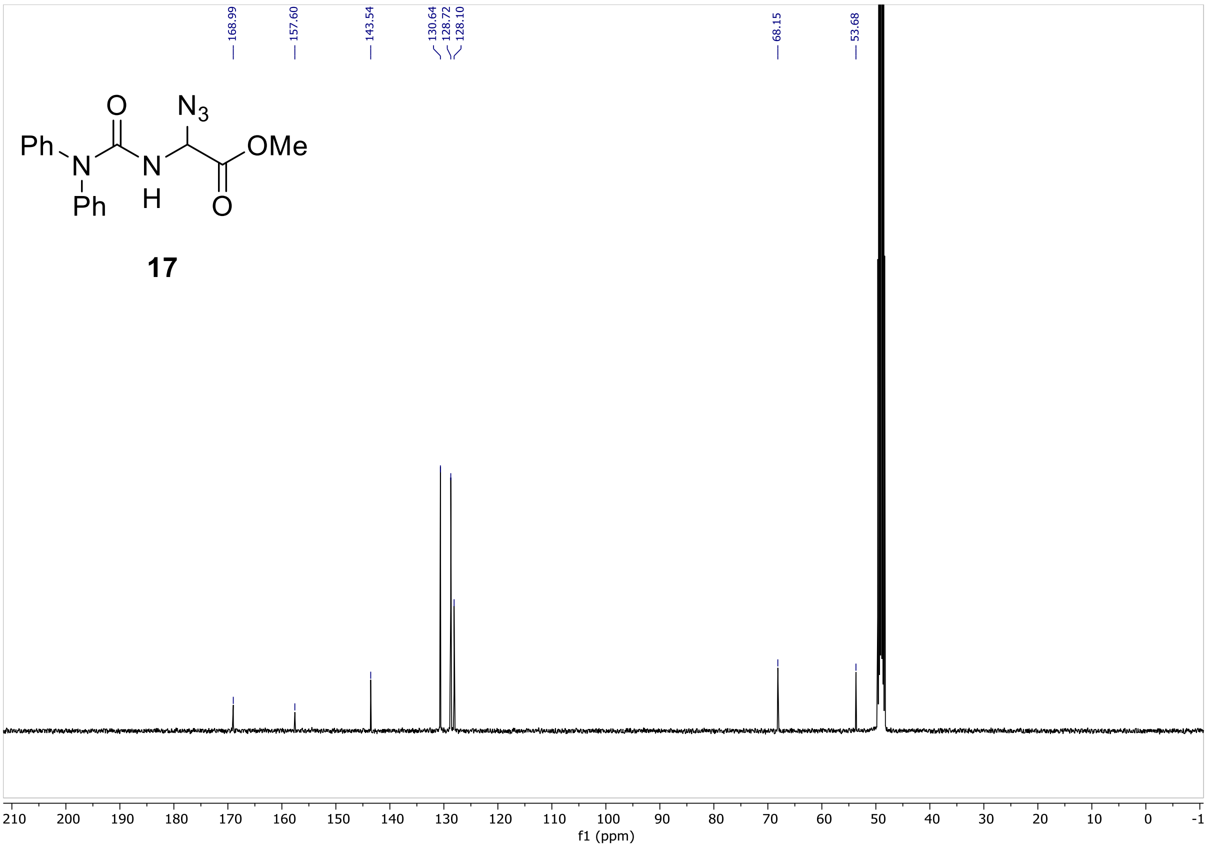

DEPT-135 (101 MHz, MeOD-d_4, 298 K) of compound **17**

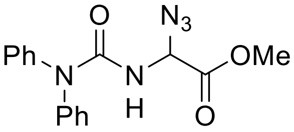

17

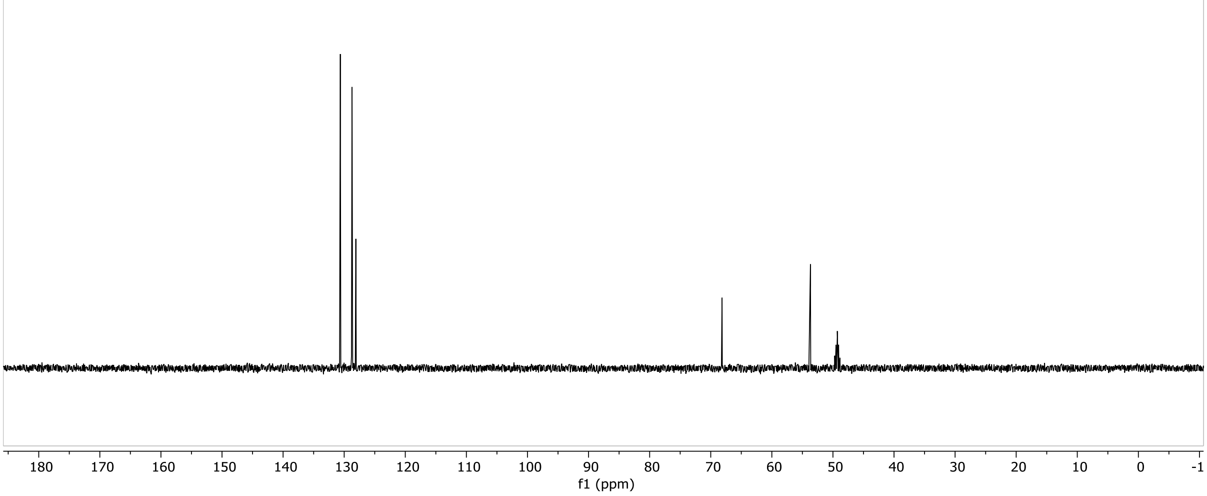

^{1}H NMR (400 MHz, $CDCl_3$, 278.2 K, complex mixture of diastereoisomers and rotamers) of **18**

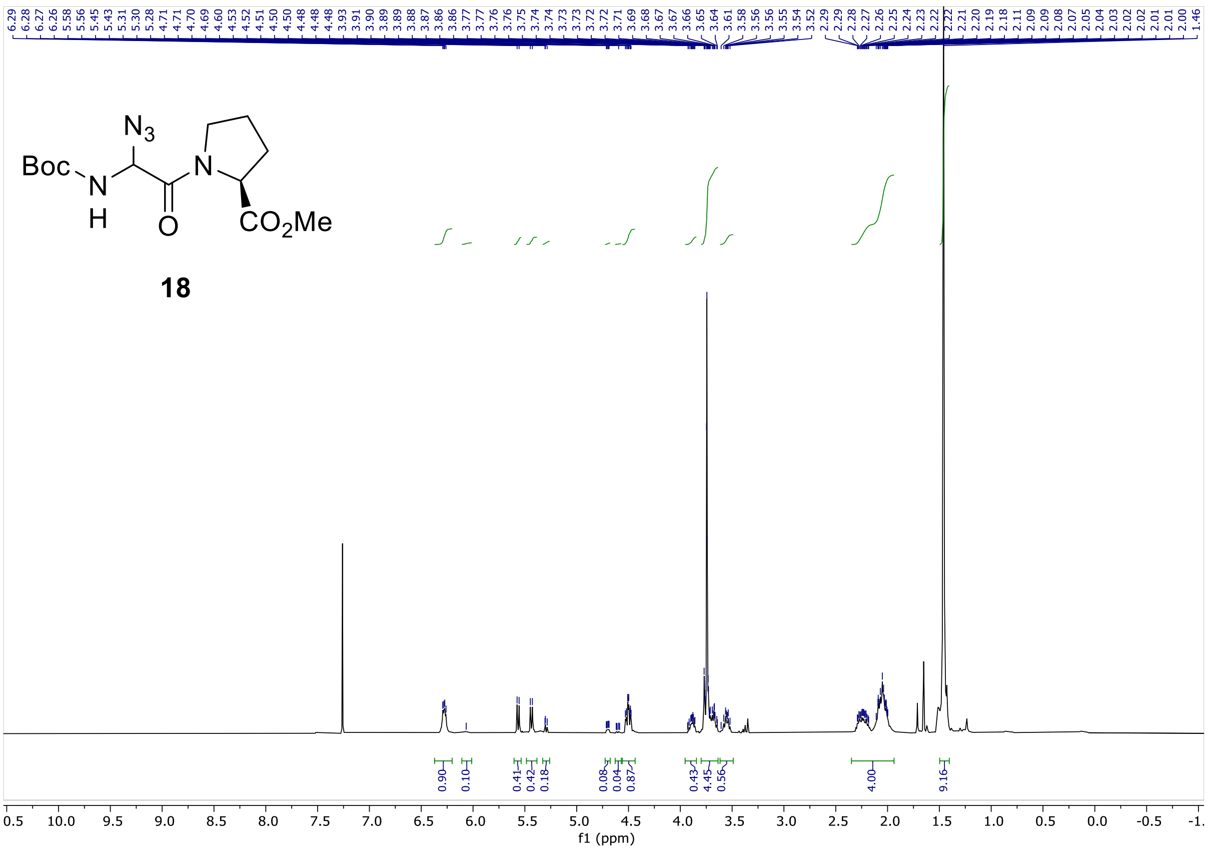

^{13}C NMR (101 MHz, $CDCl_3$, 278.2 K, complex mixture of diastereoisomers and rotamers) of **18**

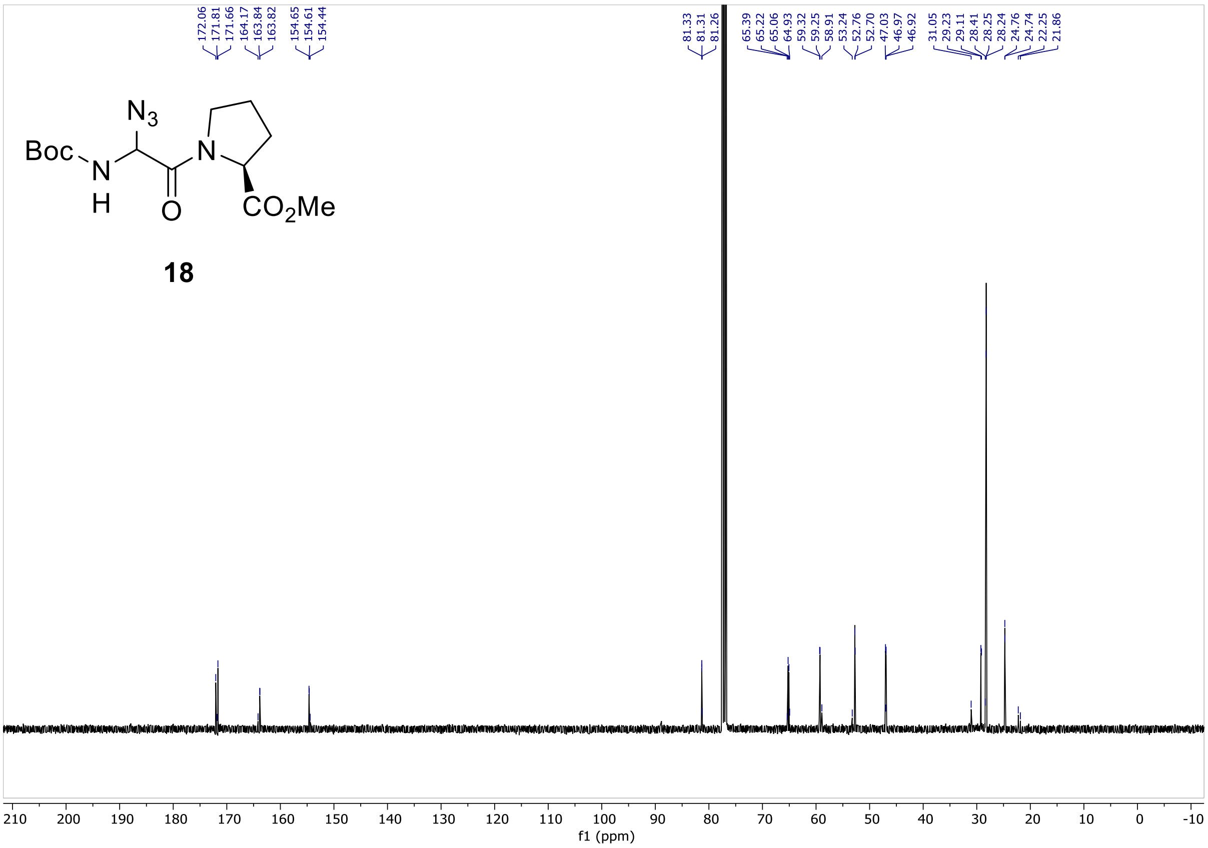

DEPT-135 (101 MHz, $CDCl_3$, 278.2 K, complex mixture of diastereoisomers and rotamers) of **18**

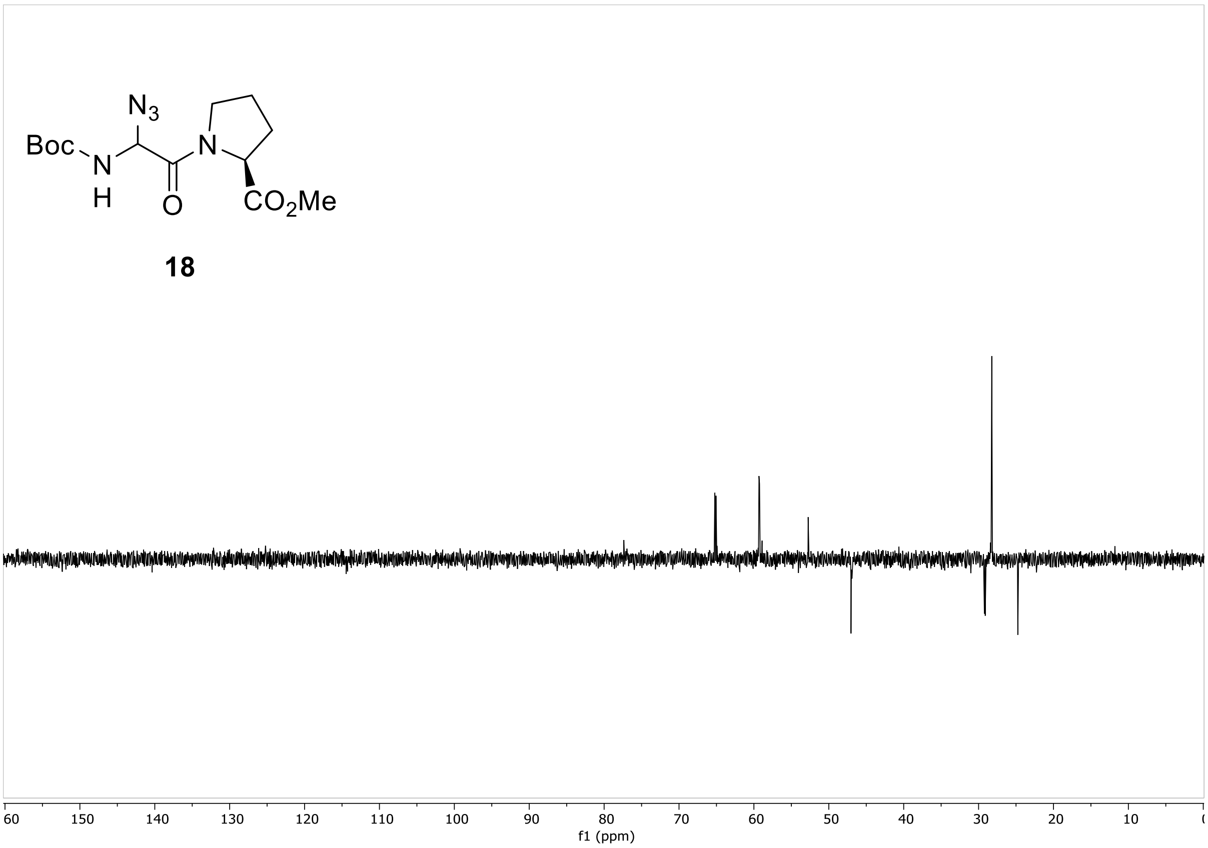

^{1}H NMR (400 MHz, MeOD-d_4, 298 K, complex mixture of rotamers) of compound **20a**

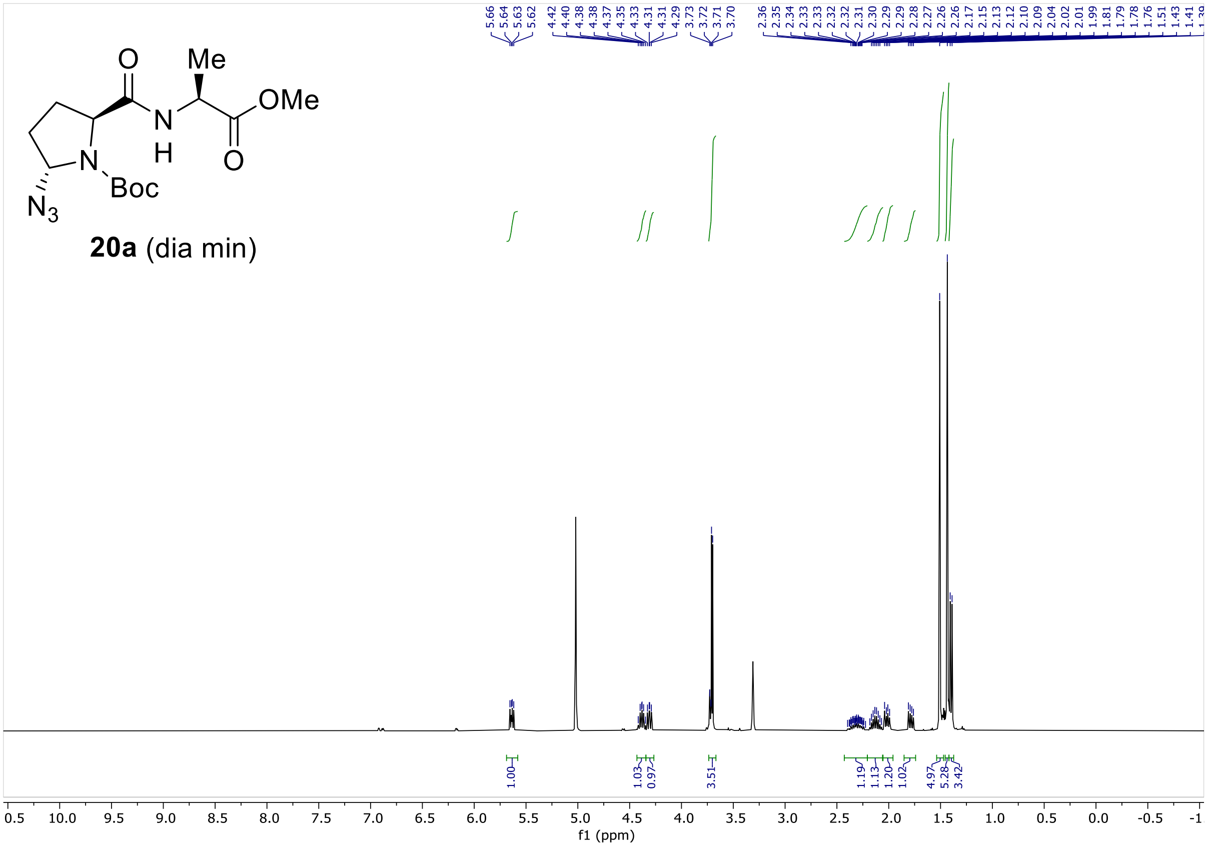

^{13}C NMR (101 MHz, MeOD-d_4, 278.2 K, complex mixture of rotamers) of compound **20a**

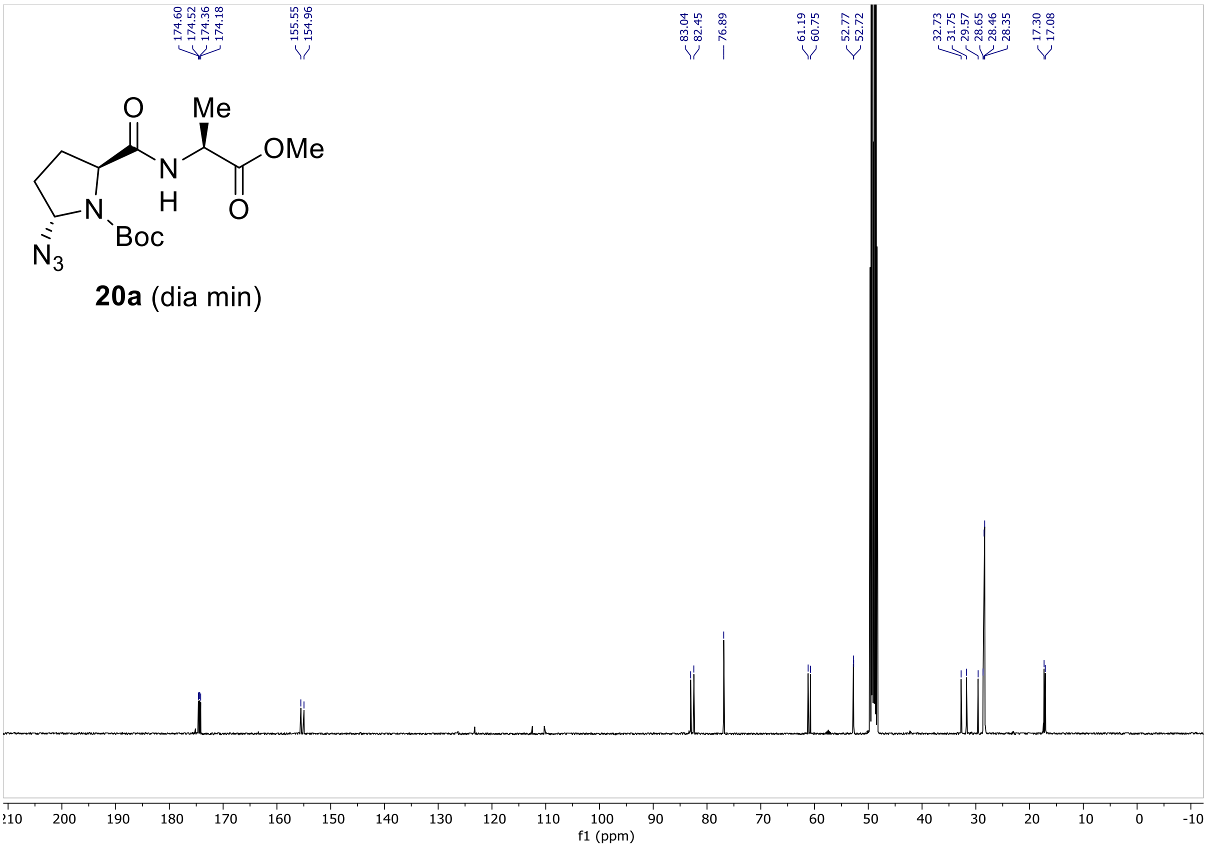

HSQC (MeOD-d_4, 278.2 K, complex mixture of rotamers) of compound **20a**

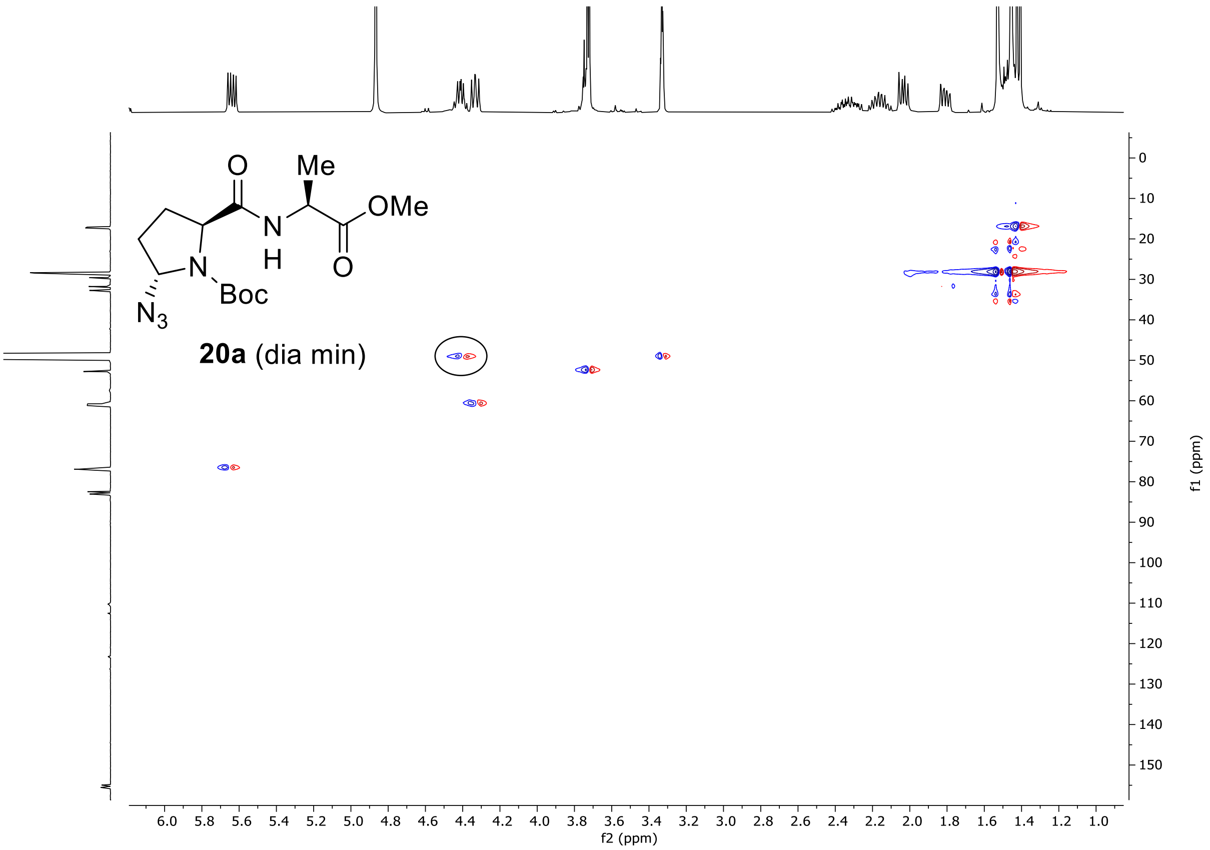

DEPT-135 (101 MHz, MeOD-d_4, 278.2 K, complex mixture of rotamers) of compound **20a**

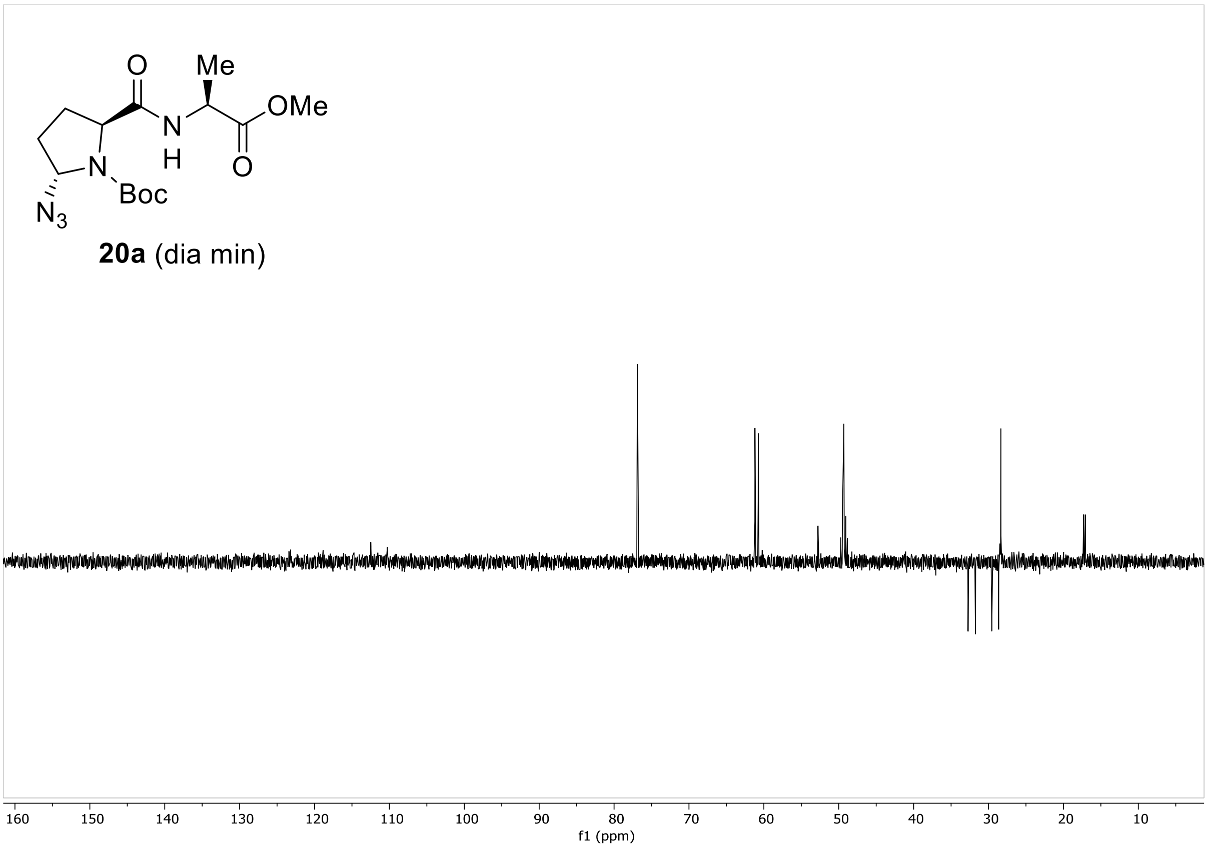

^{1}H NMR (400 MHz, MeOD-d_4, 278.2 K, complex mixture of rotamers) of compound **20b**

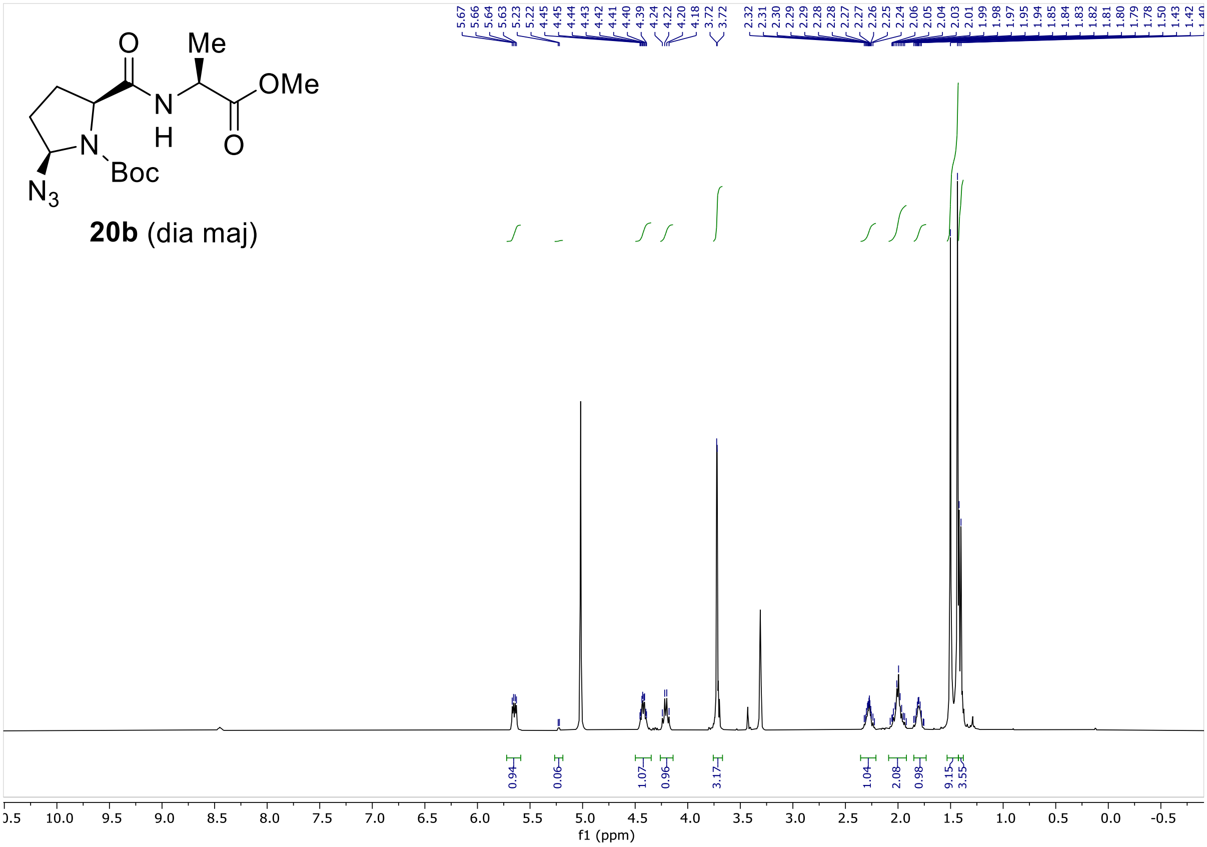

^{13}C NMR (101 MHz, MeOD-d_4, 278.2 K, complex mixture of rotamers) of compound **20b**

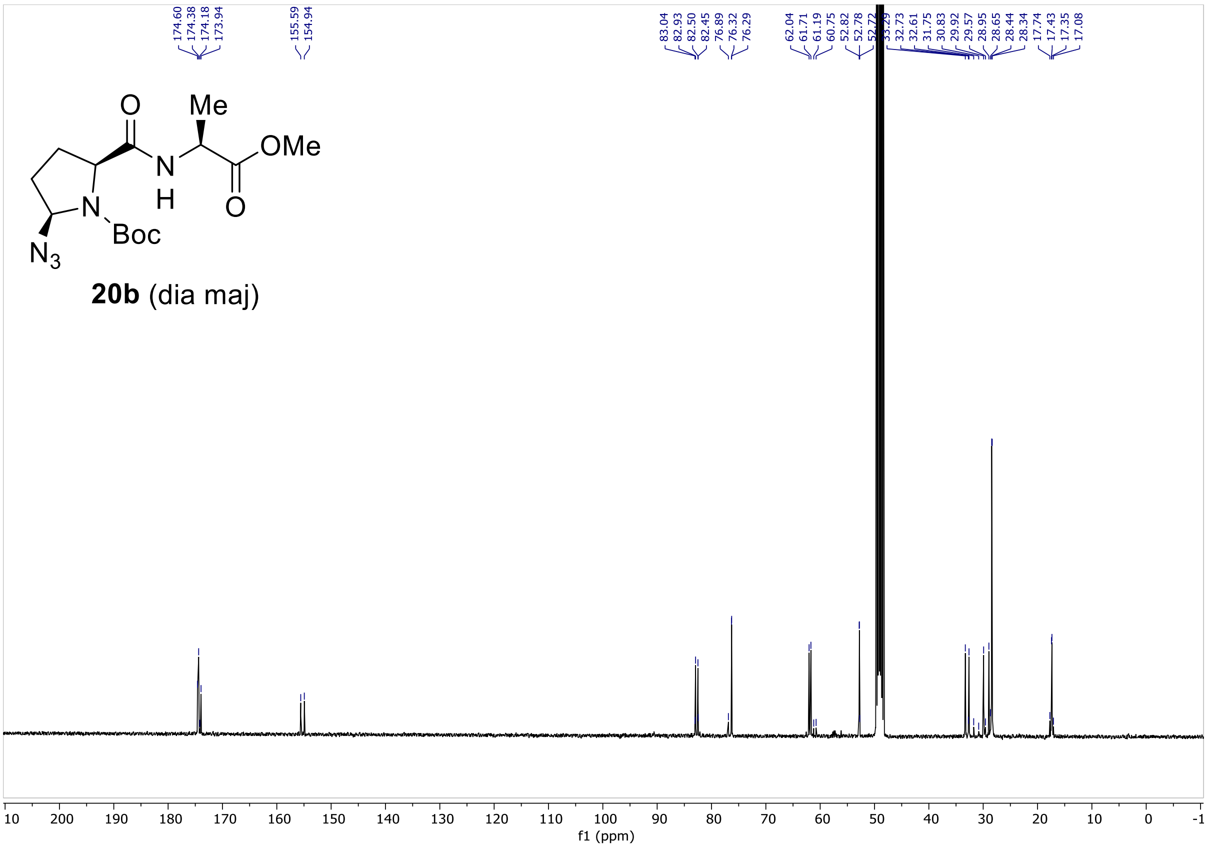

HSQC (MeOD-d_4, 278.2 K, complex mixture of rotamers) of compound **20b**

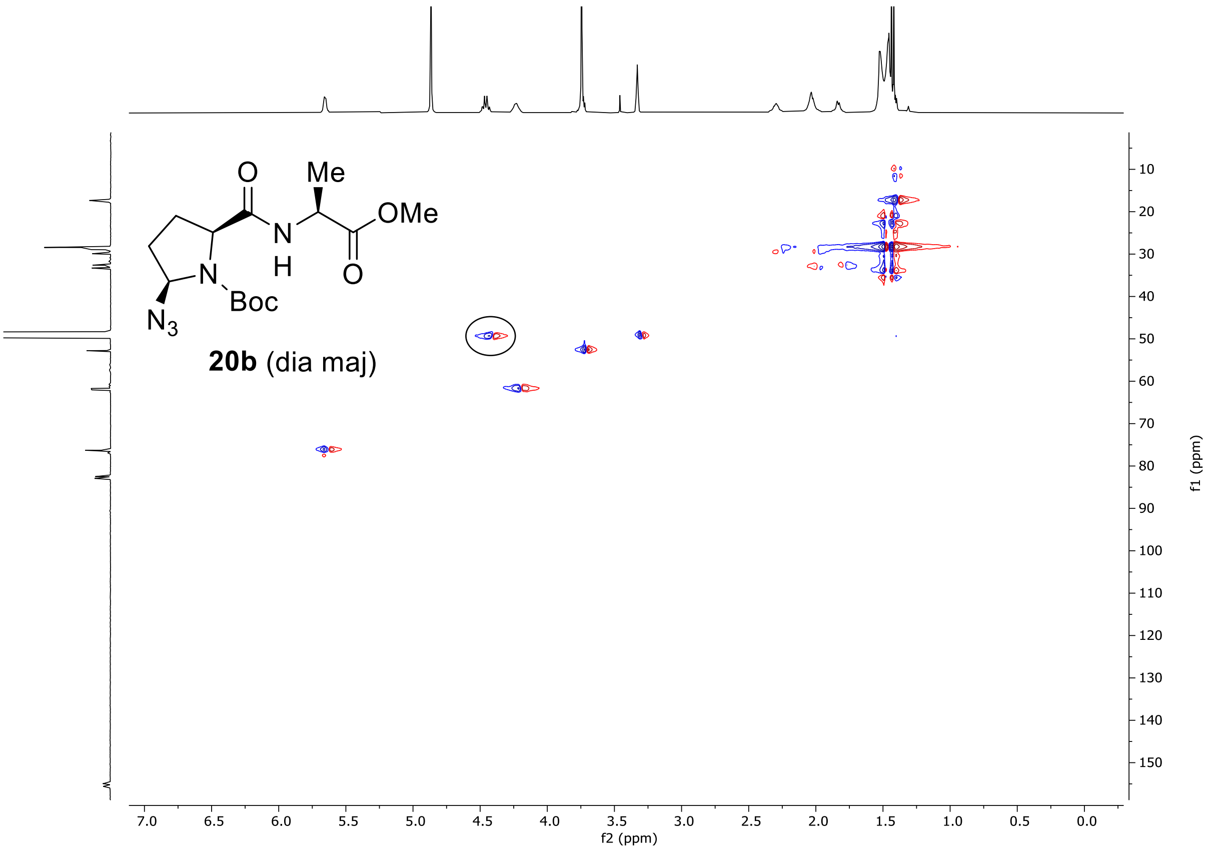

DEPT-135 (101 MHz, MeOD-d_4, 278.2 K, complex mixture of rotamers) of compound **20b**

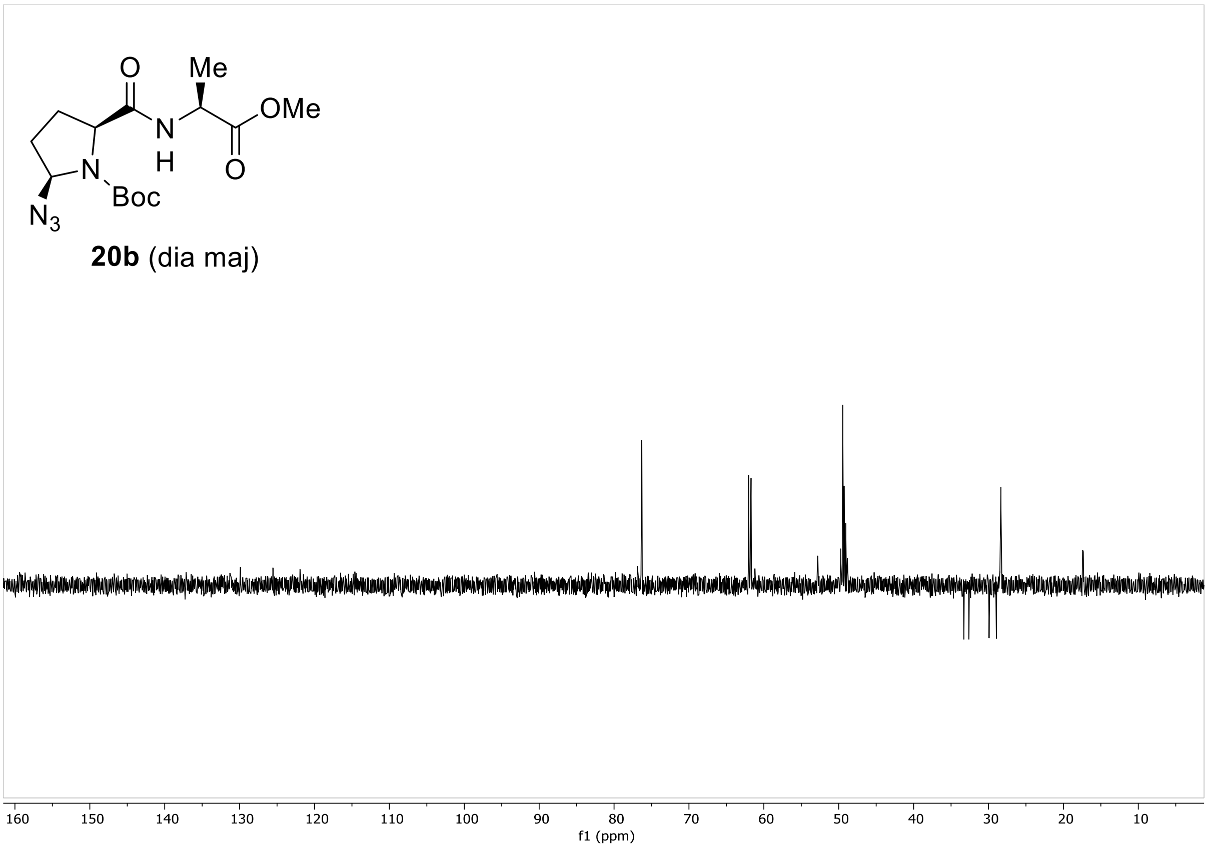

NOESY (400 MHz, MeOD-d_4, 278.2 K, complex mixture of rotamers) of compound **20b**

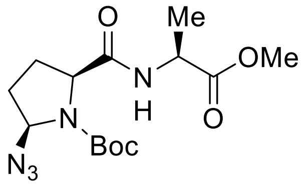

20b (dia maj)

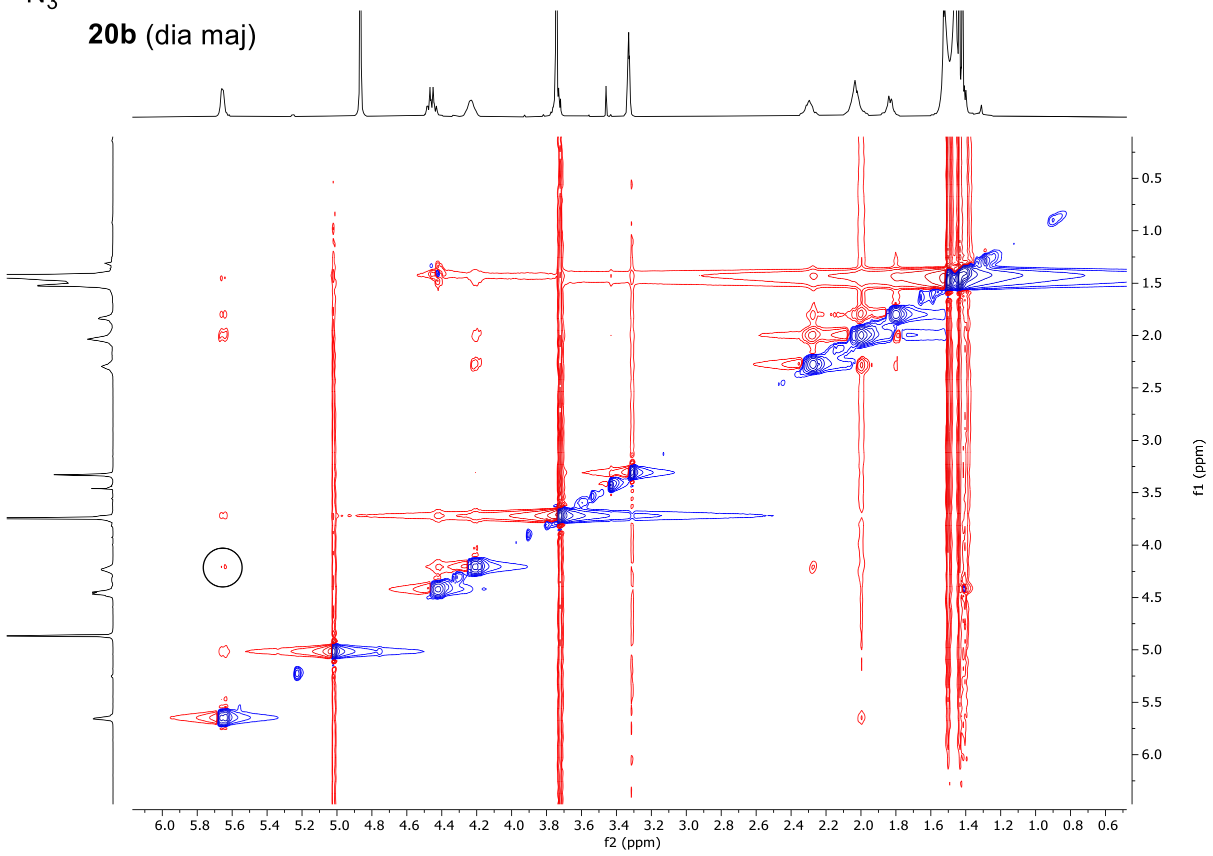

^{1}H NMR (400 MHz, MeOD-*d₄*, 298 K, complex mixture of rotamers) of compound **21a**

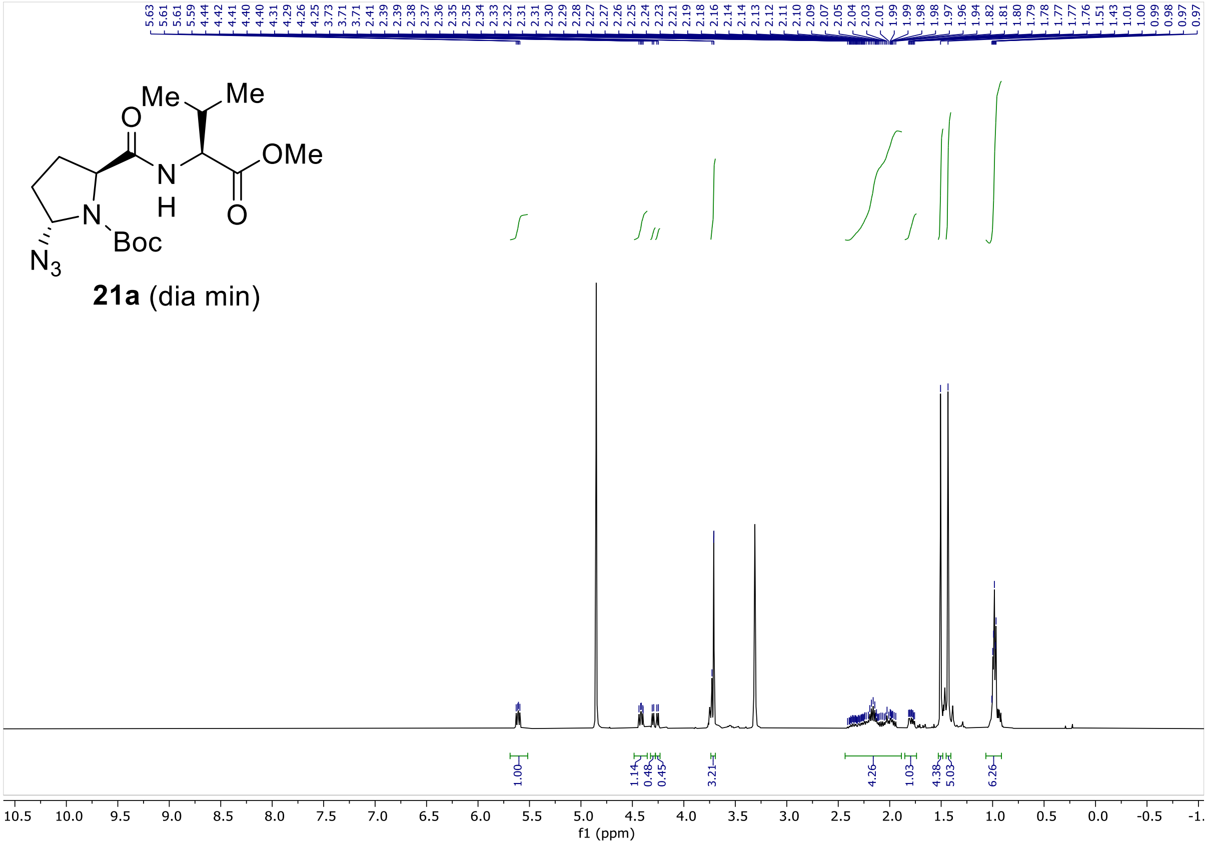

^{13}C NMR (101 MHz, MeOD-*d₄*, 278.2 K, complex mixture of rotamers) of compound **21a**

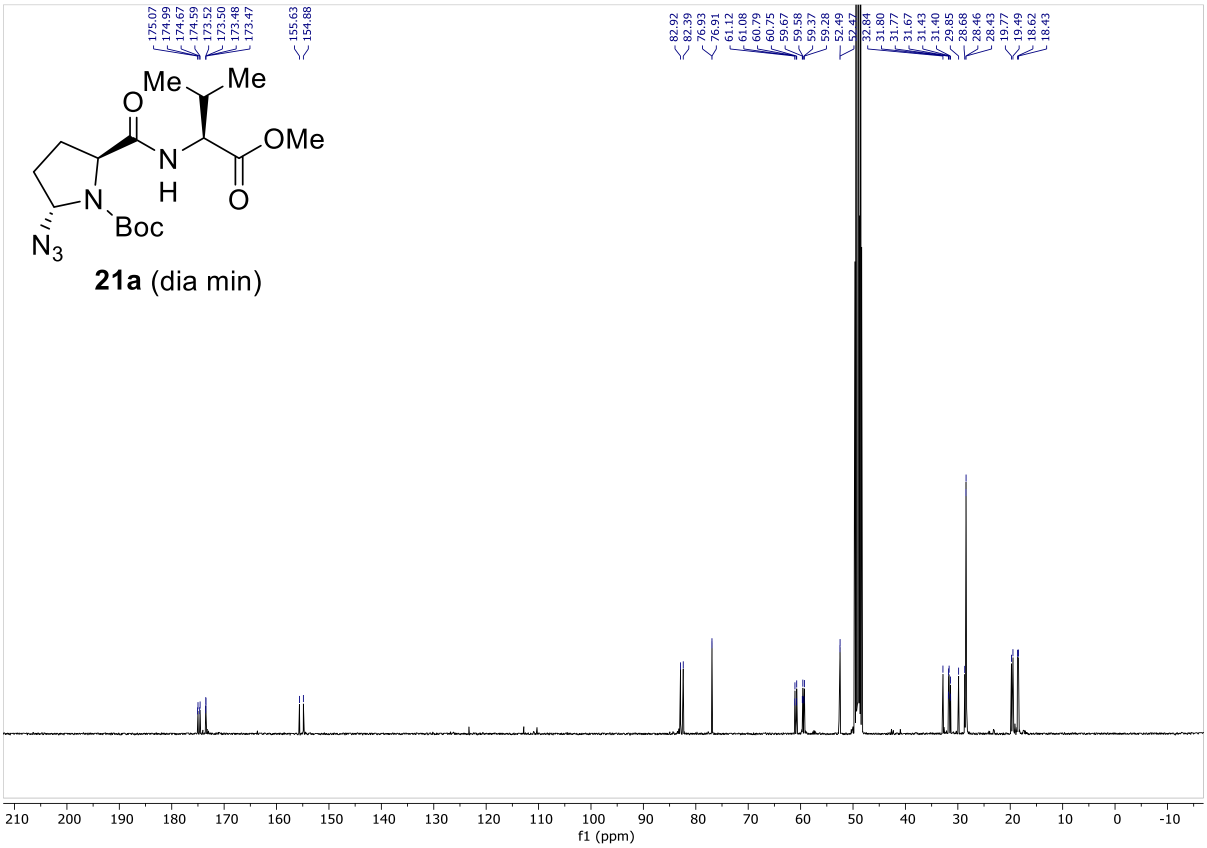

DEPT-135 (101 MHz, MeOD-d_4, 278.2 K, complex mixture of rotamers) of compound **21a**

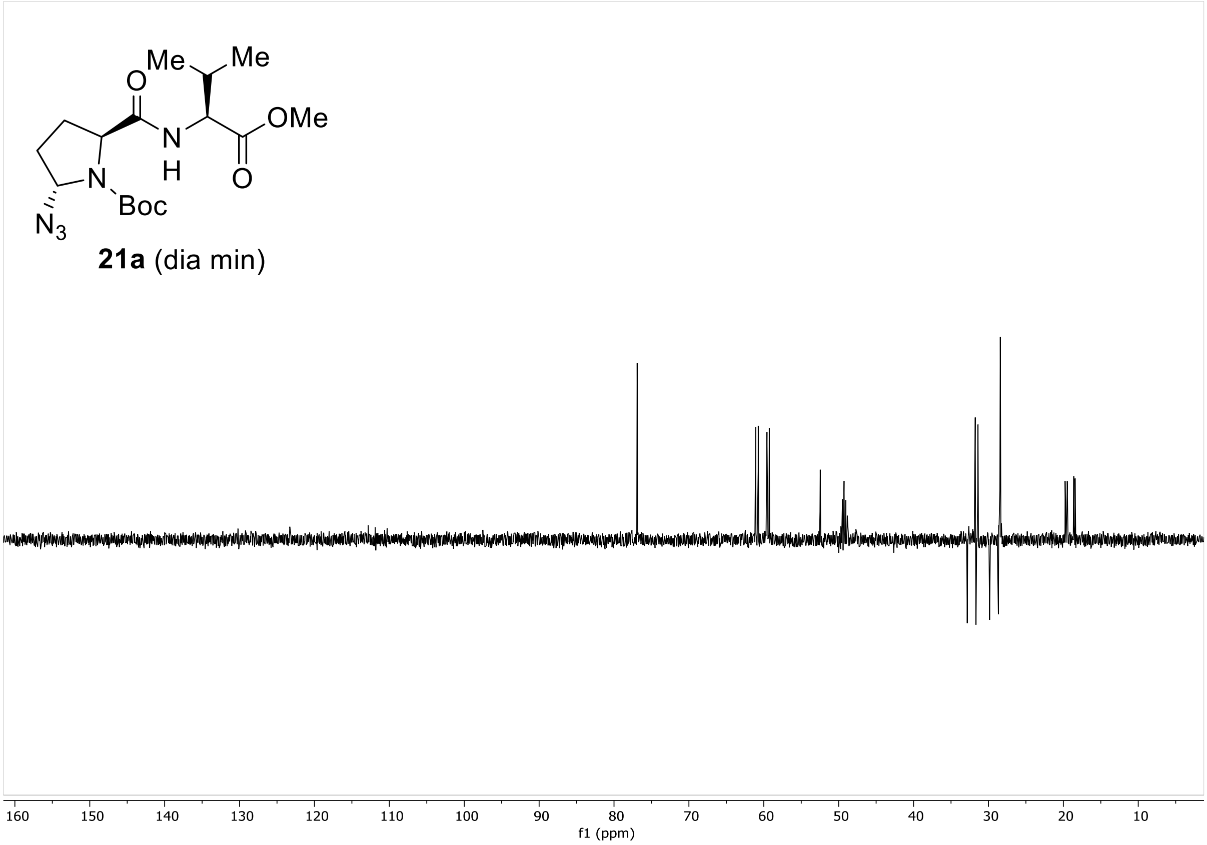

[1]**H NMR** (400 MHz, MeOD-*d₄*, 298 K, complex mixture of rotamers) of compound **21b**

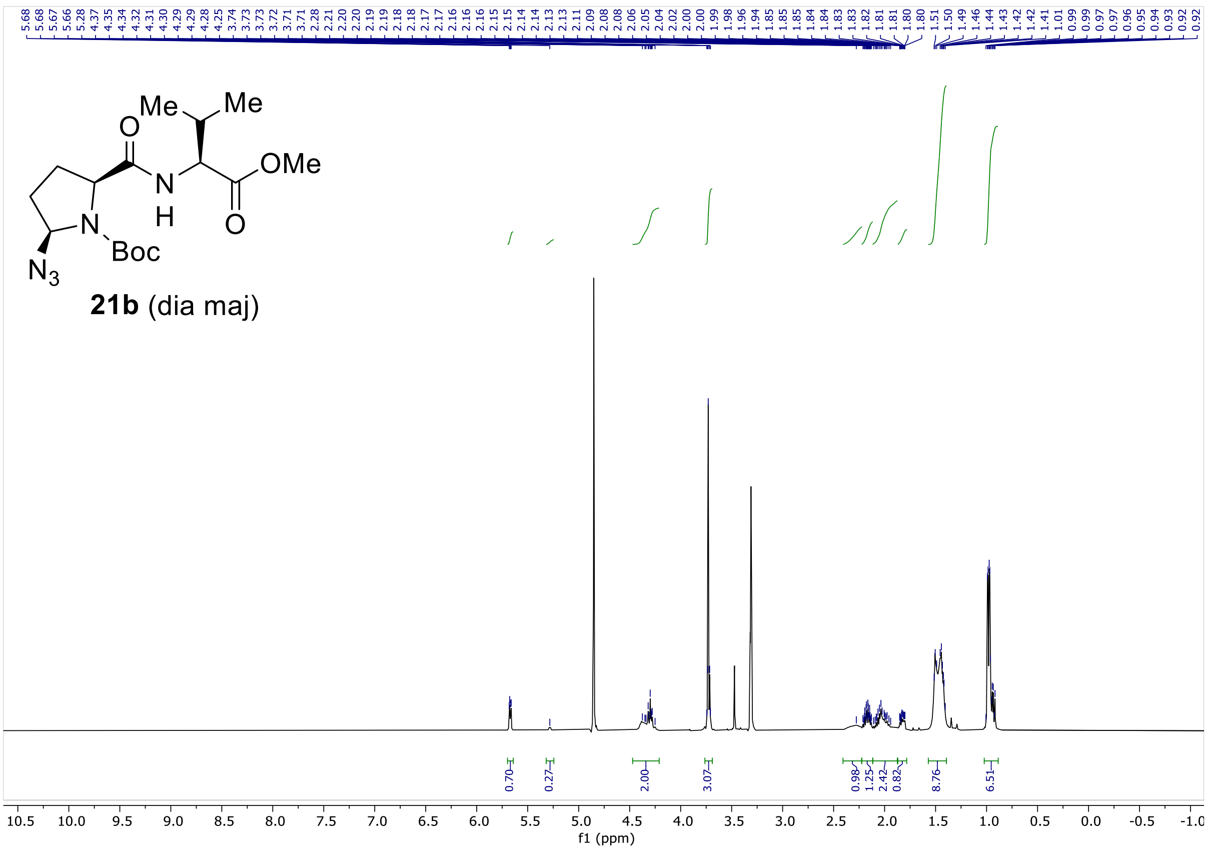

[13]**C NMR** (101 MHz, MeOD-*d₄*, 278.2 K, complex mixture of rotamers) of compound **21b**

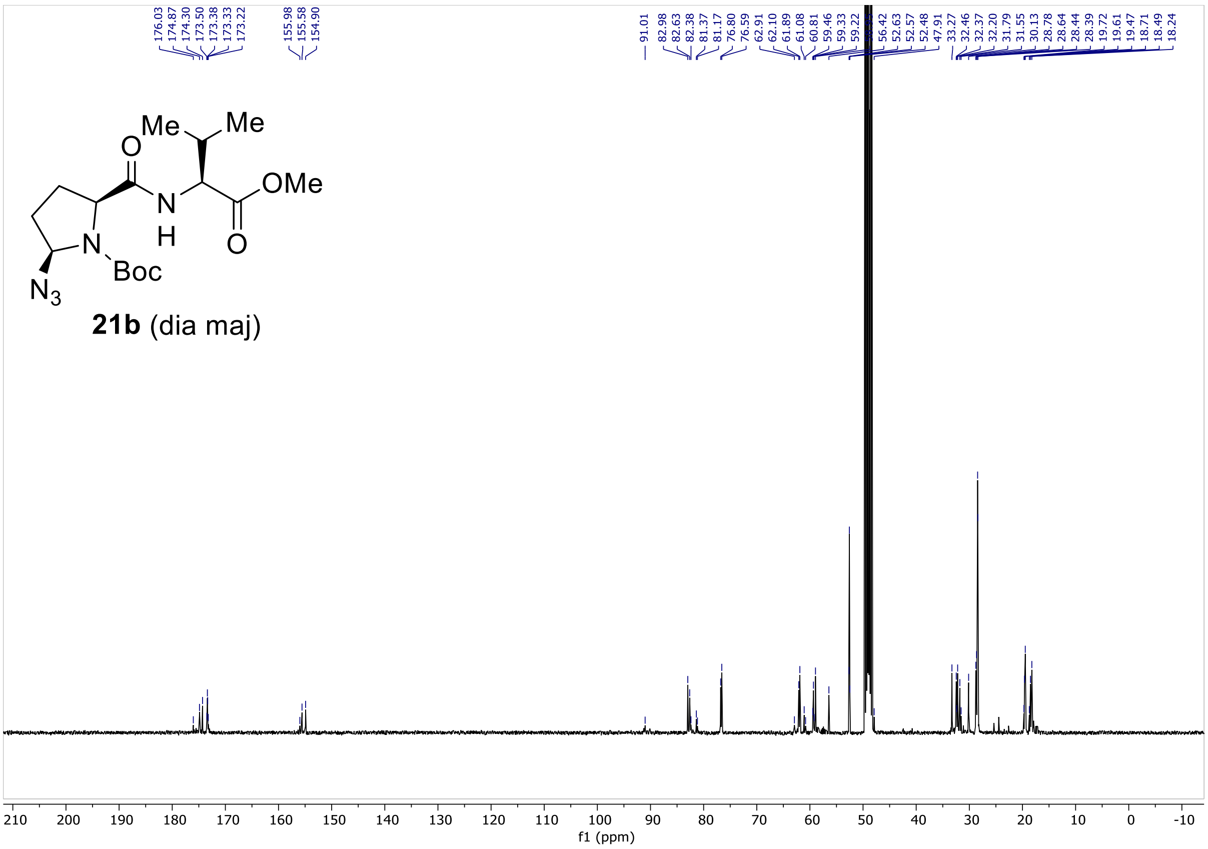

DEPT-135 (101 MHz, MeOD-d_4, 278.2 K, complex mixture of rotamers) of compound **21b**

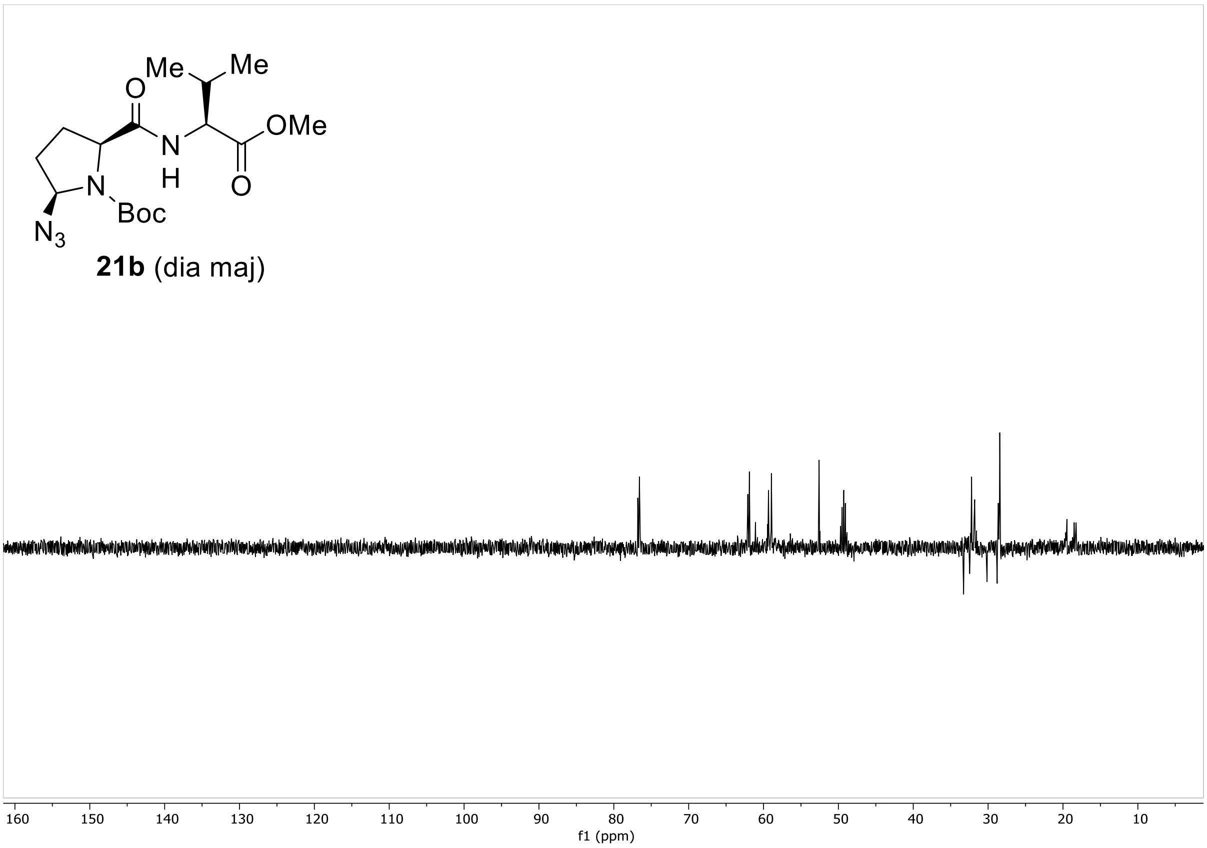

NOESY (400 MHz, MeOD-d_4, 298 K, complex mixture of rotamers) of compound **21b**

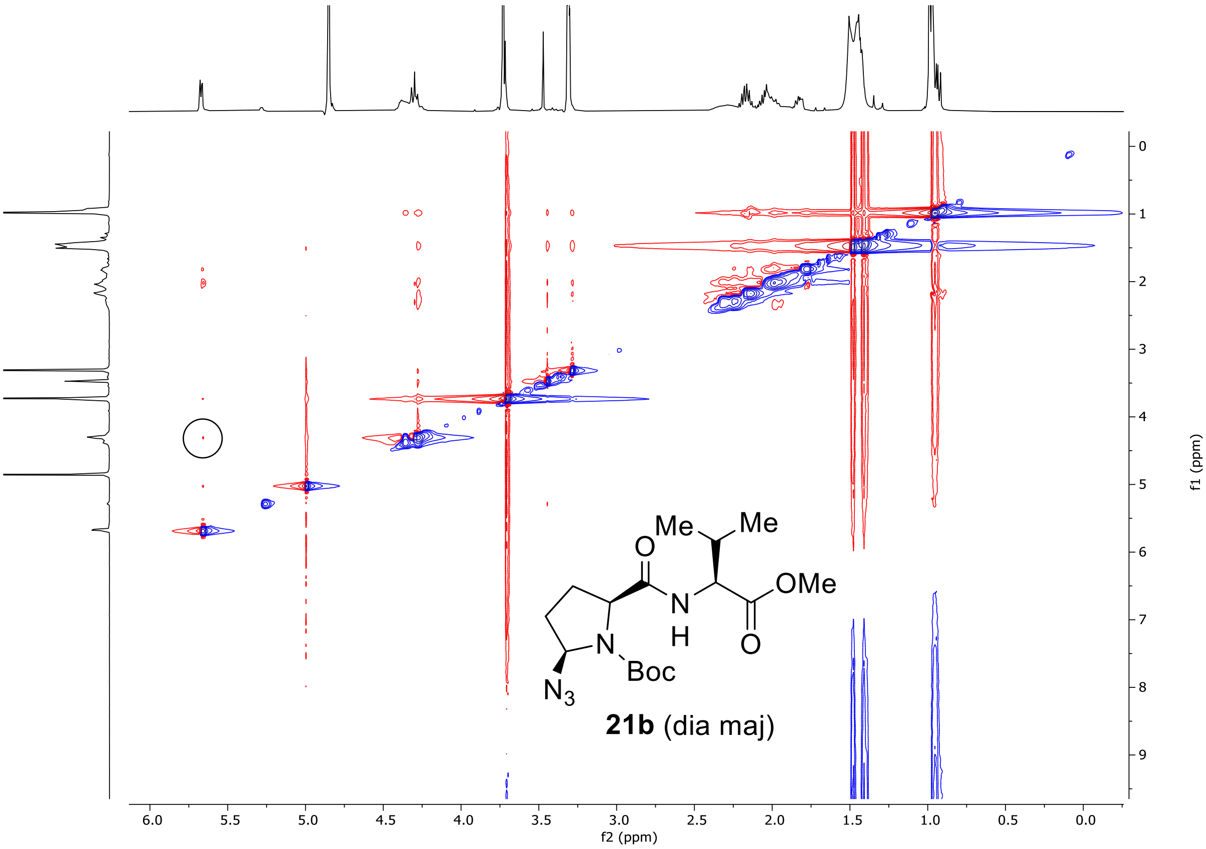

^{1}H NMR (400 MHz, MeOD-d_4, 298 K, complex mixture of rotamers) of compound **22a**

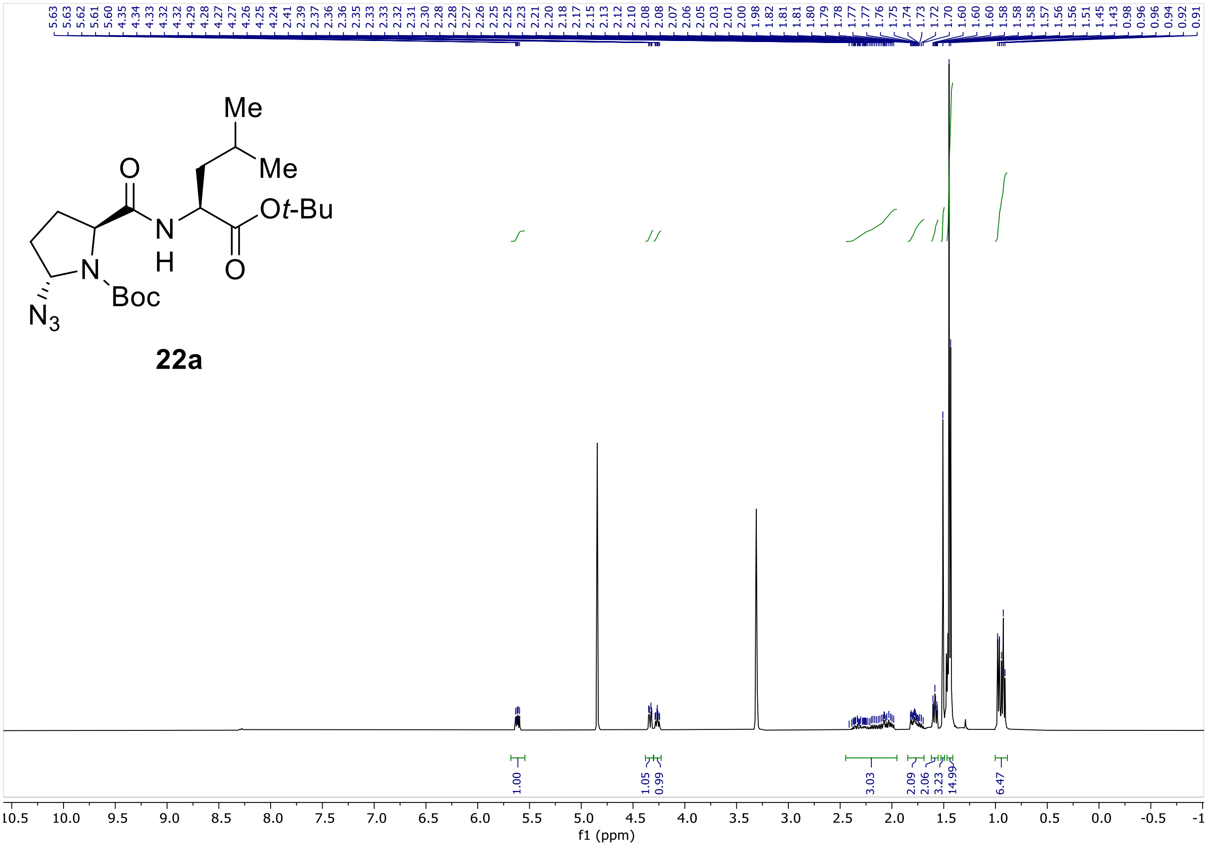

^{13}C NMR (101 MHz, MeOD-d_4, 298 K, complex mixture of rotamers) of compound **22a**

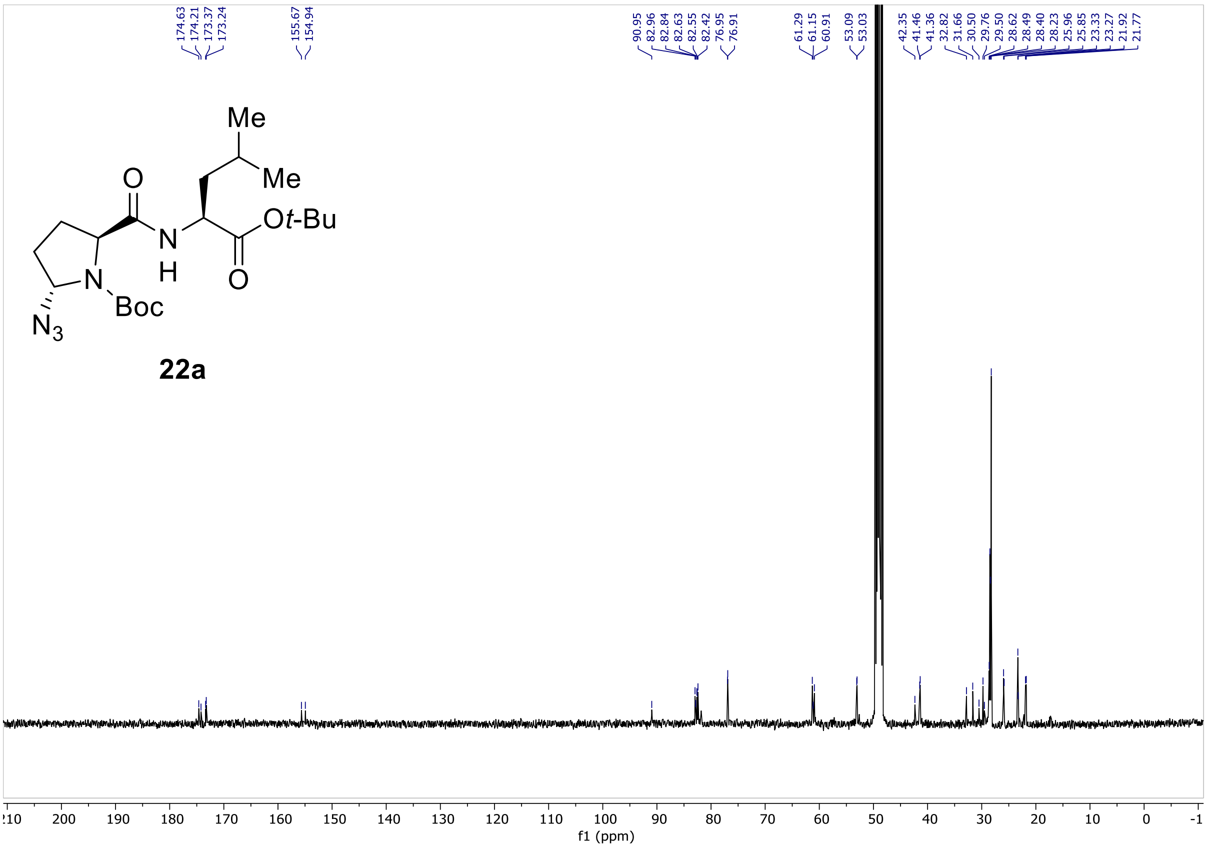

DEPT-135 (101 MHz, MeOD-d_4, 298 K, complex mixture of rotamers) of compound **22a**

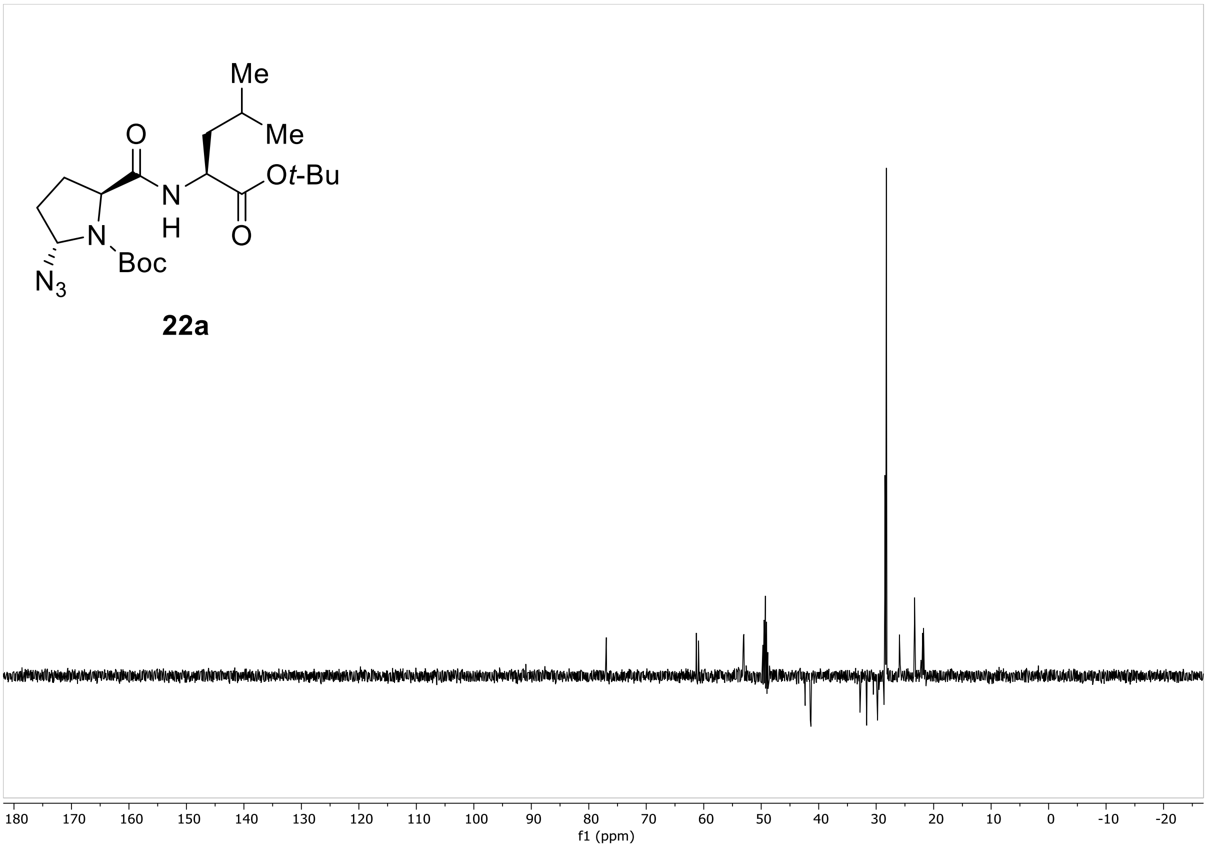

^{1}H NMR (400 MHz, MeOD-d_4, 298 K, complex mixture of rotamers) of compound **22b**

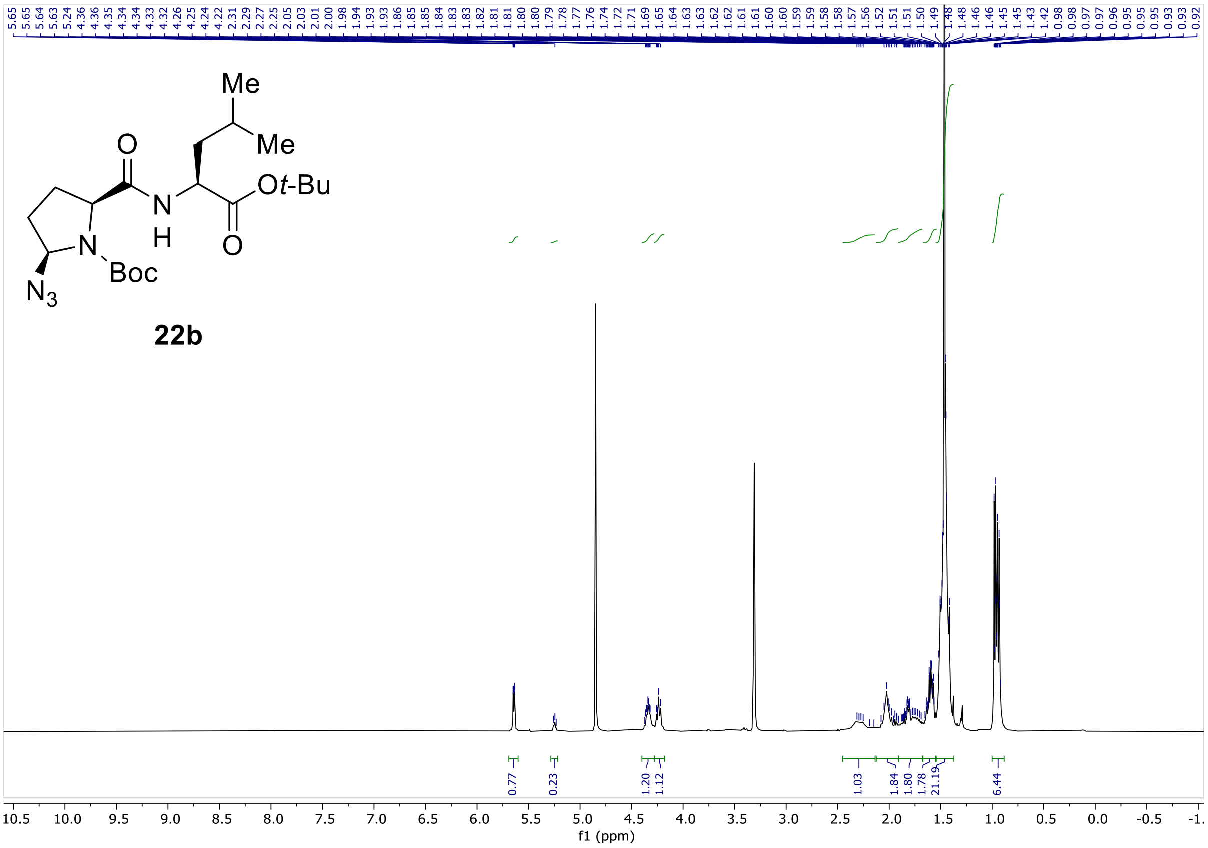

^{13}C NMR (101 MHz, MeOD-d_4, 278.2 K, complex mixture of rotamers) of compound **22b**

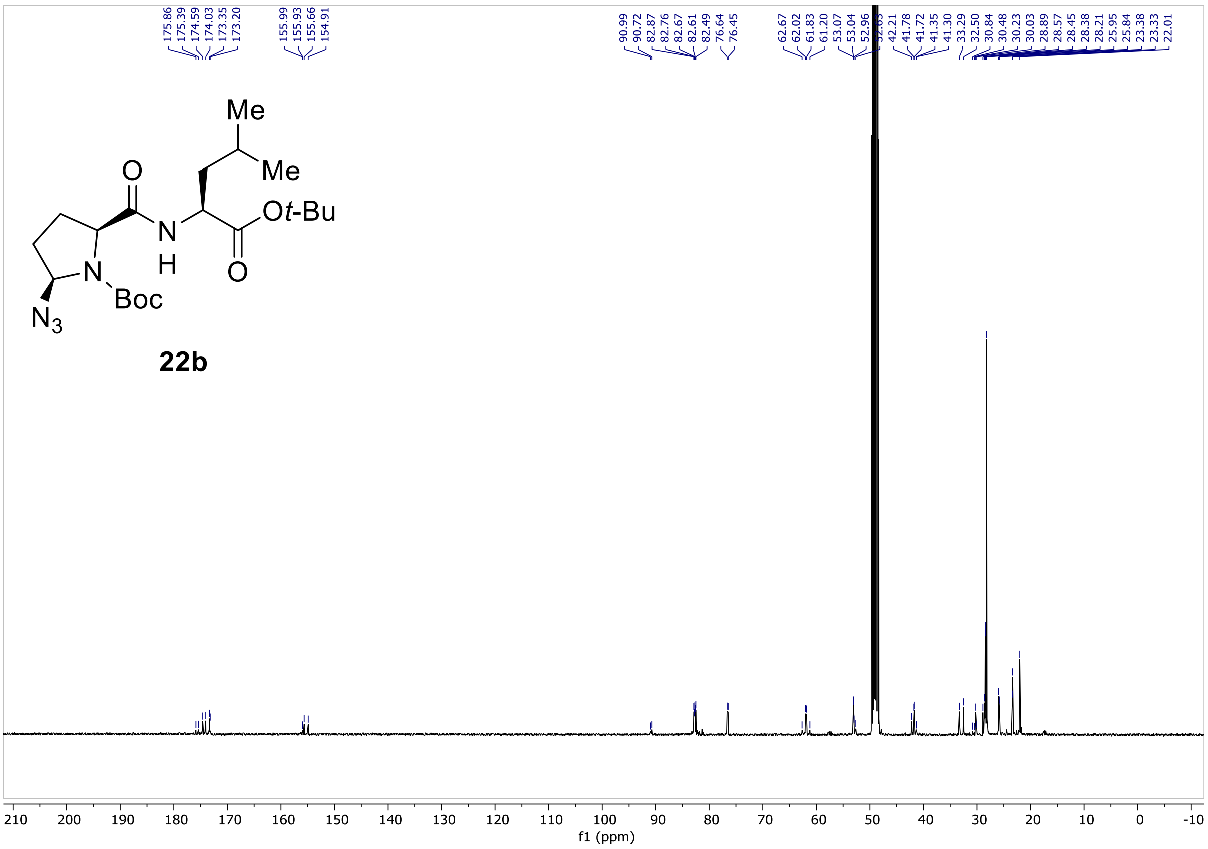

DEPT-135 (101 MHz, MeOD-d_4, 278.2 K, complex mixture of rotamers) of compound **22b**

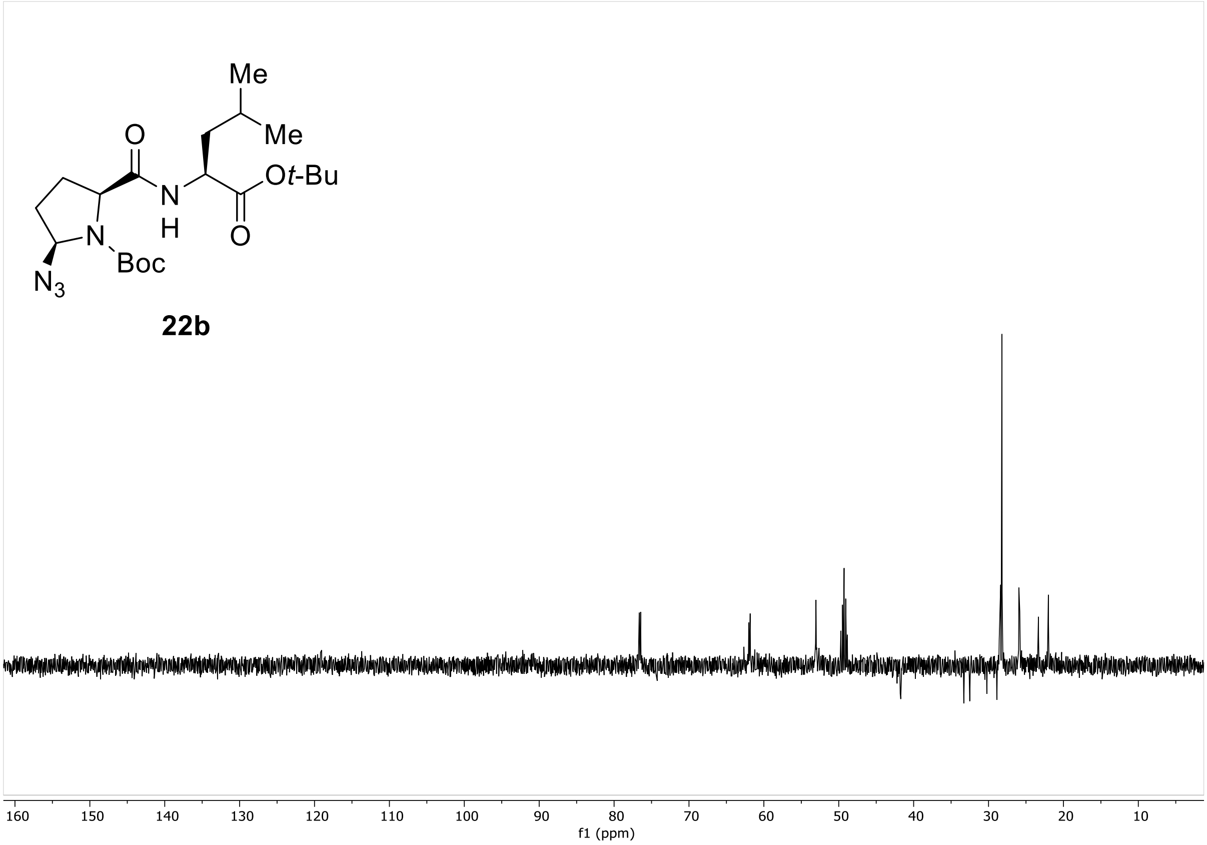

NOESY (400 MHz, MeOD-d_4, 298 K, complex mixture of rotamers) of compound **22b**

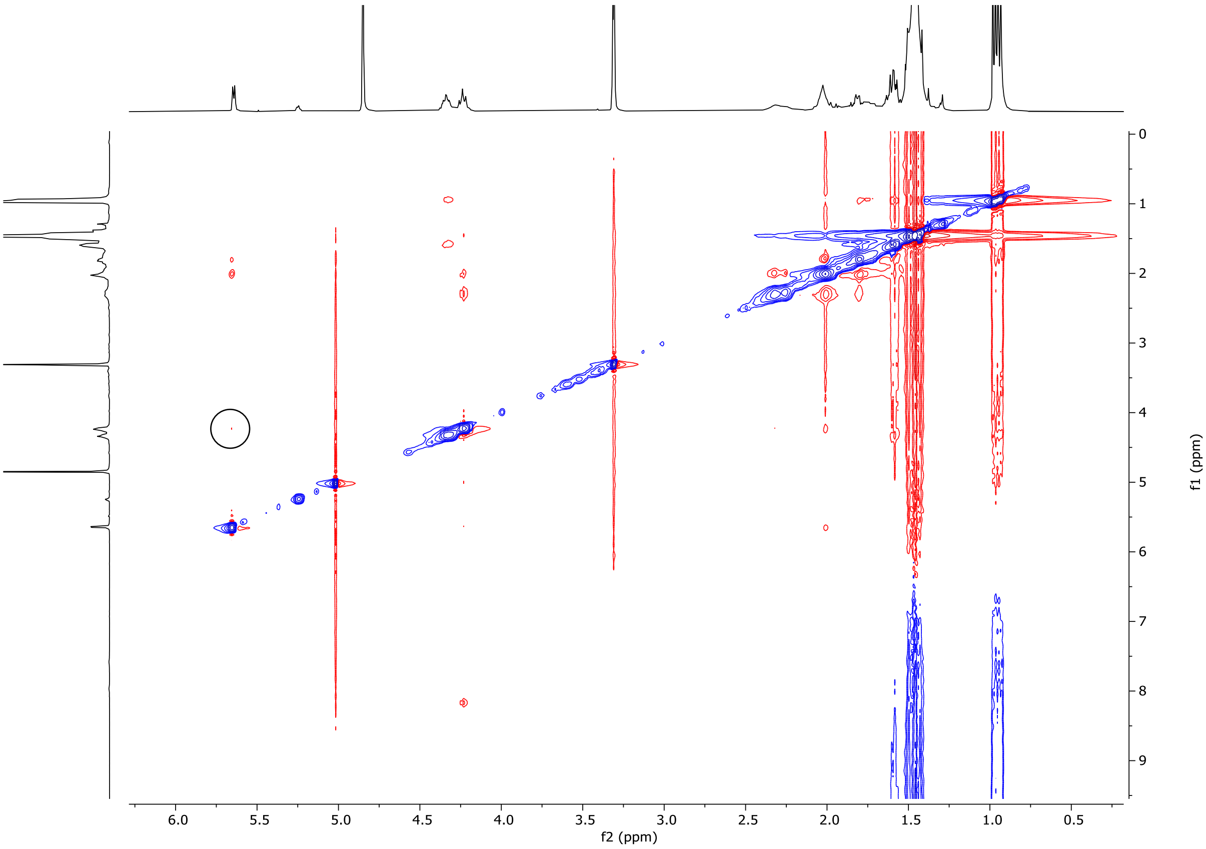

^{1}H NMR (400 MHz, MeOD-d_4, 298 K, mixture of two rotamers) of compound **23a**

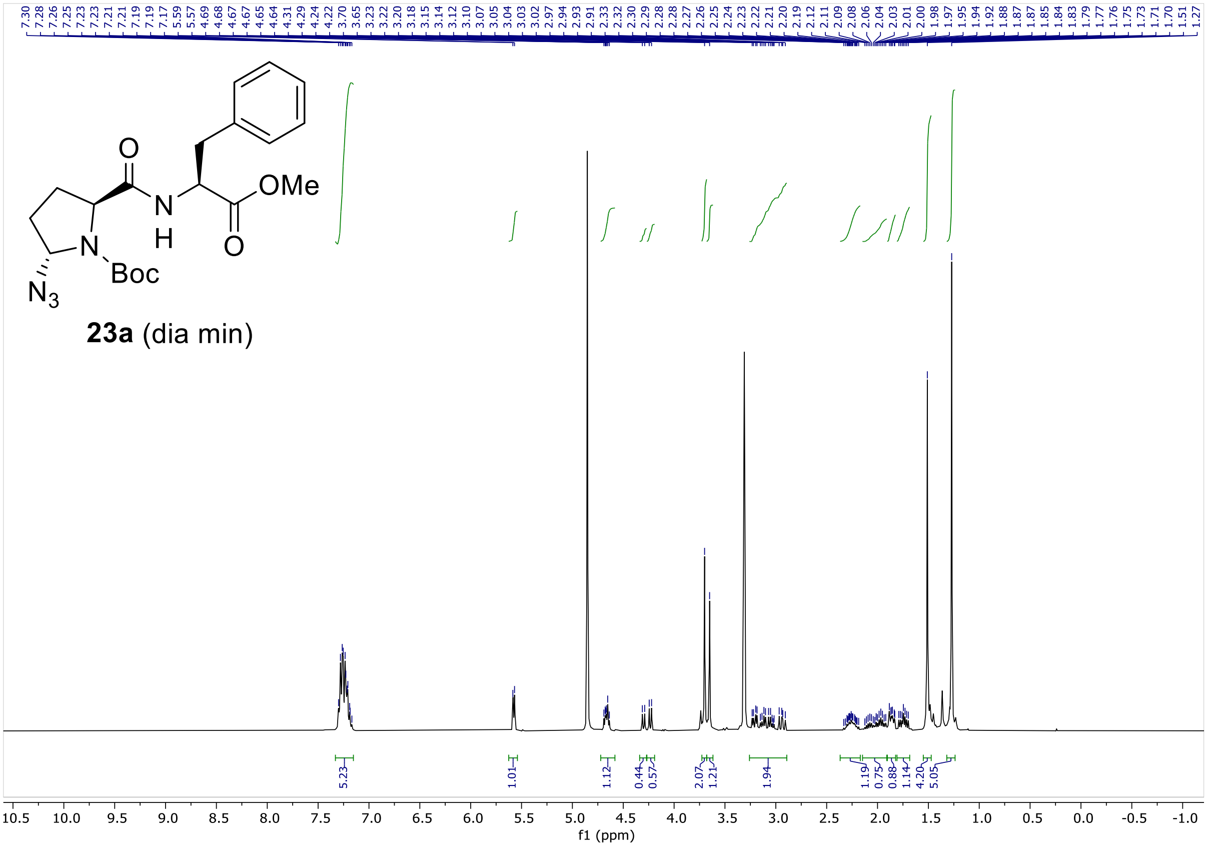

^{13}C NMR (101 MHz, MeOD-d_4, 298 K, mixture of two rotamers) of compound **23a**

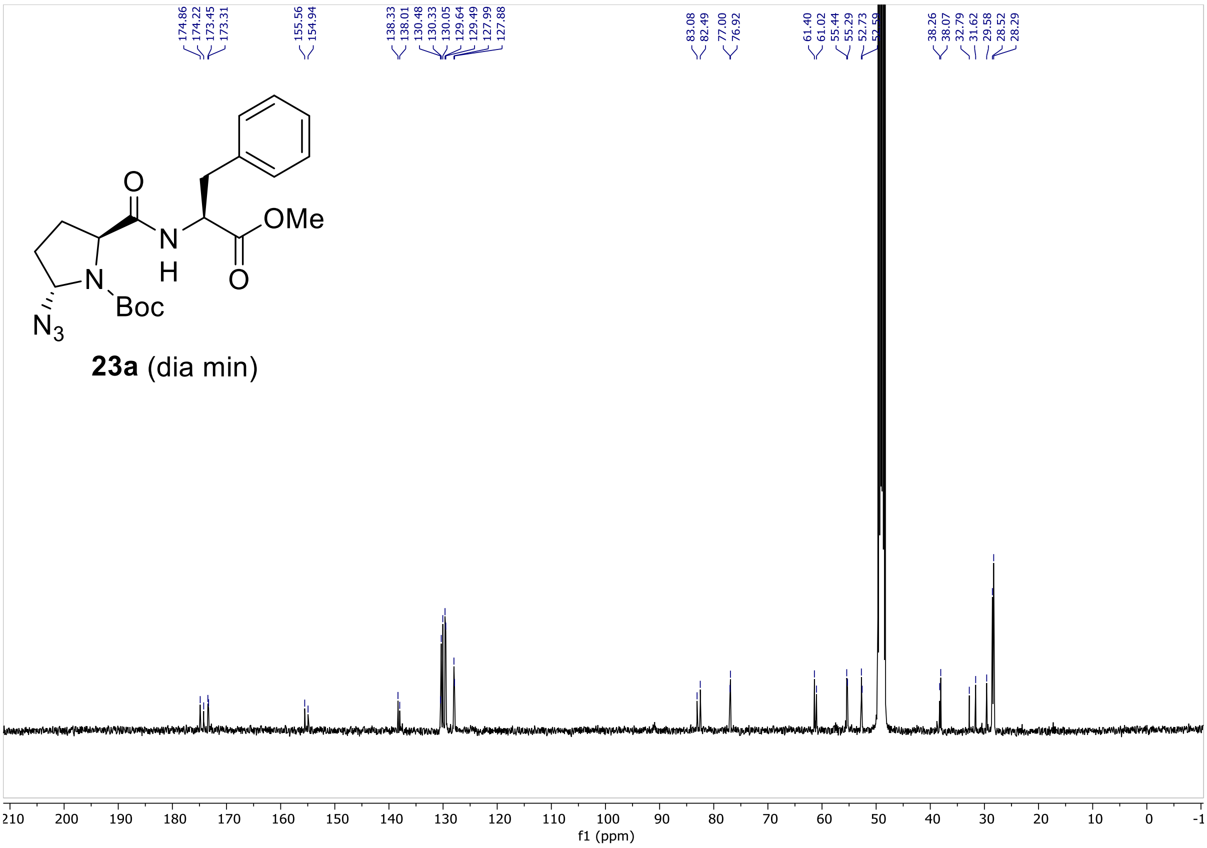

DEPT-135 (101 MHz, MeOD-d_4, 298 K, mixture of two rotamers) of compound **23a**

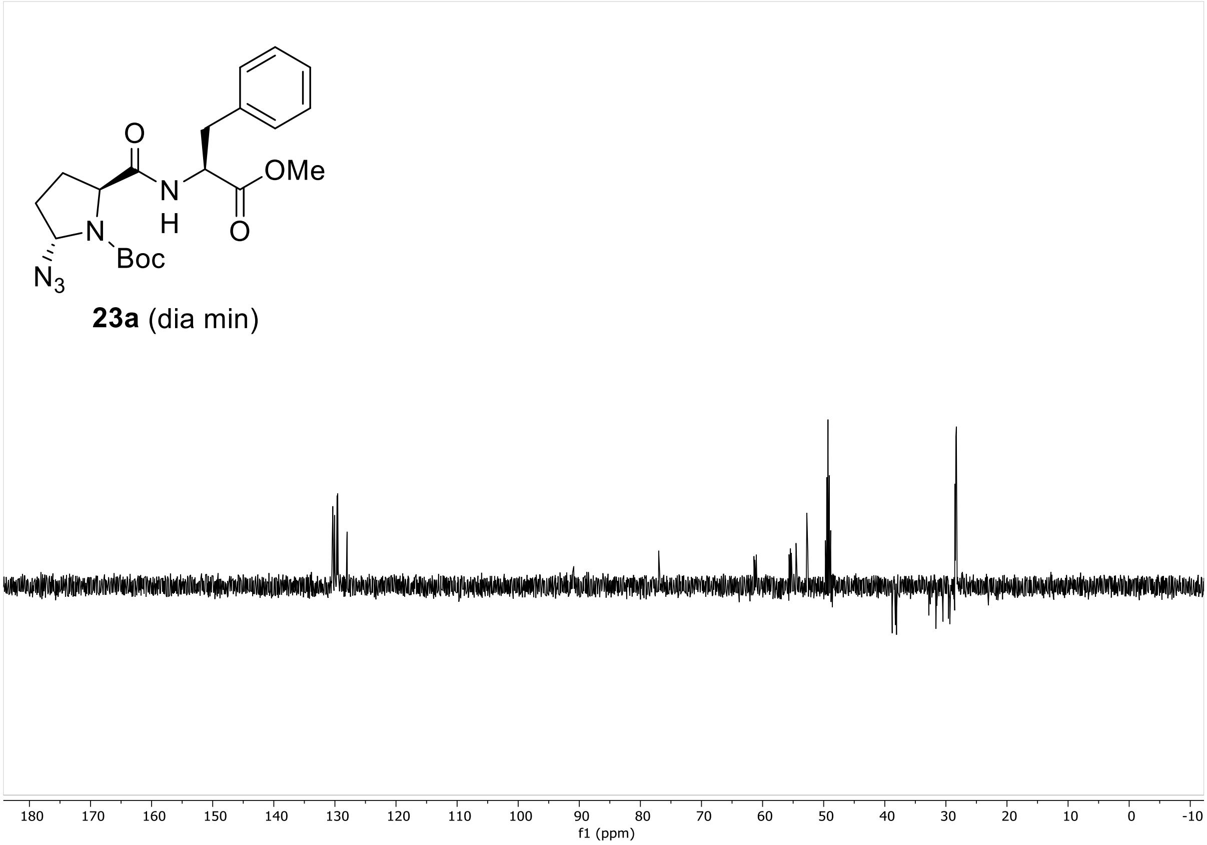

^{1}H NMR (400 MHz, MeOD-*d$_4$*, 298 K, complex mixture of rotamers) of compound **23b**

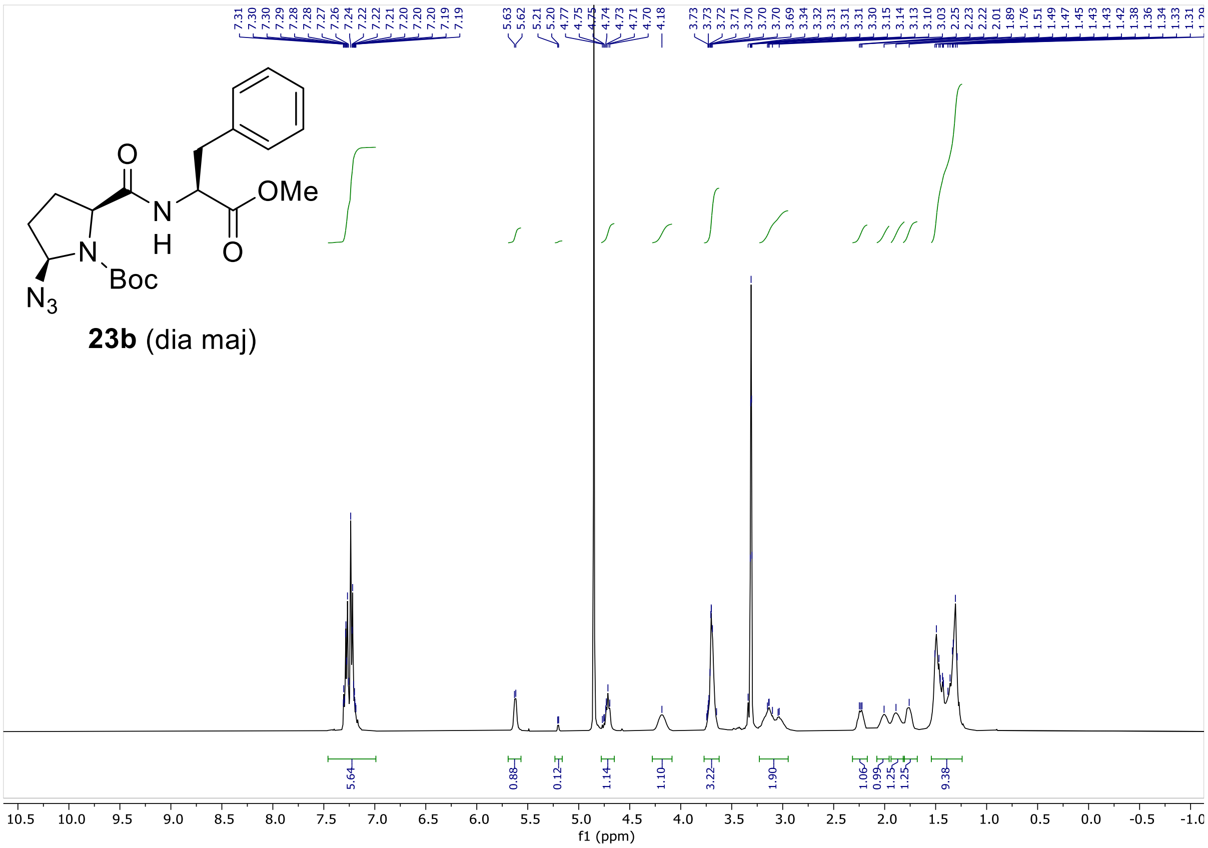

^{13}C NMR (101 MHz, MeOD-*d$_4$*, 278.2 K, complex mixture of rotamers) of compound **23b**

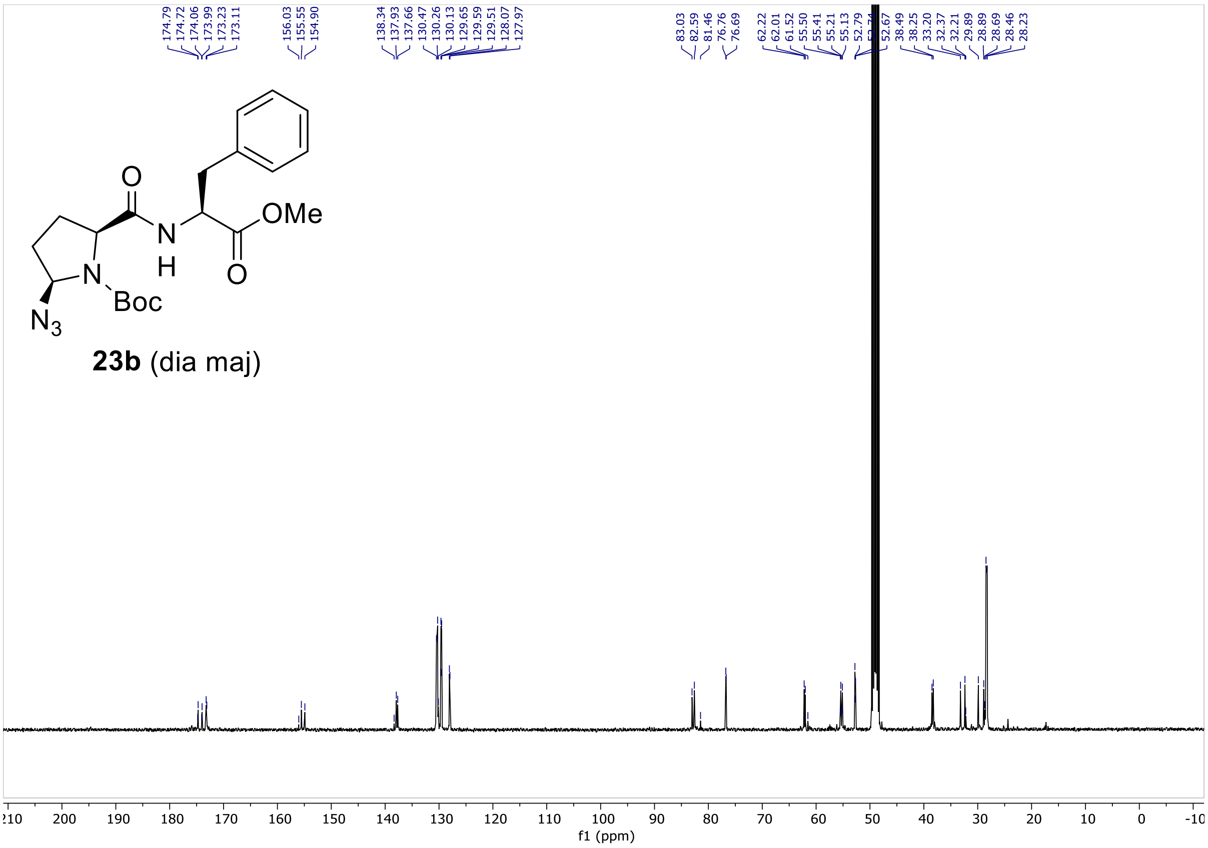

DEPT-135 (101 MHz, MeOD-*d₄*, 278.2 K, complex mixture of rotamers) of compound **23b**

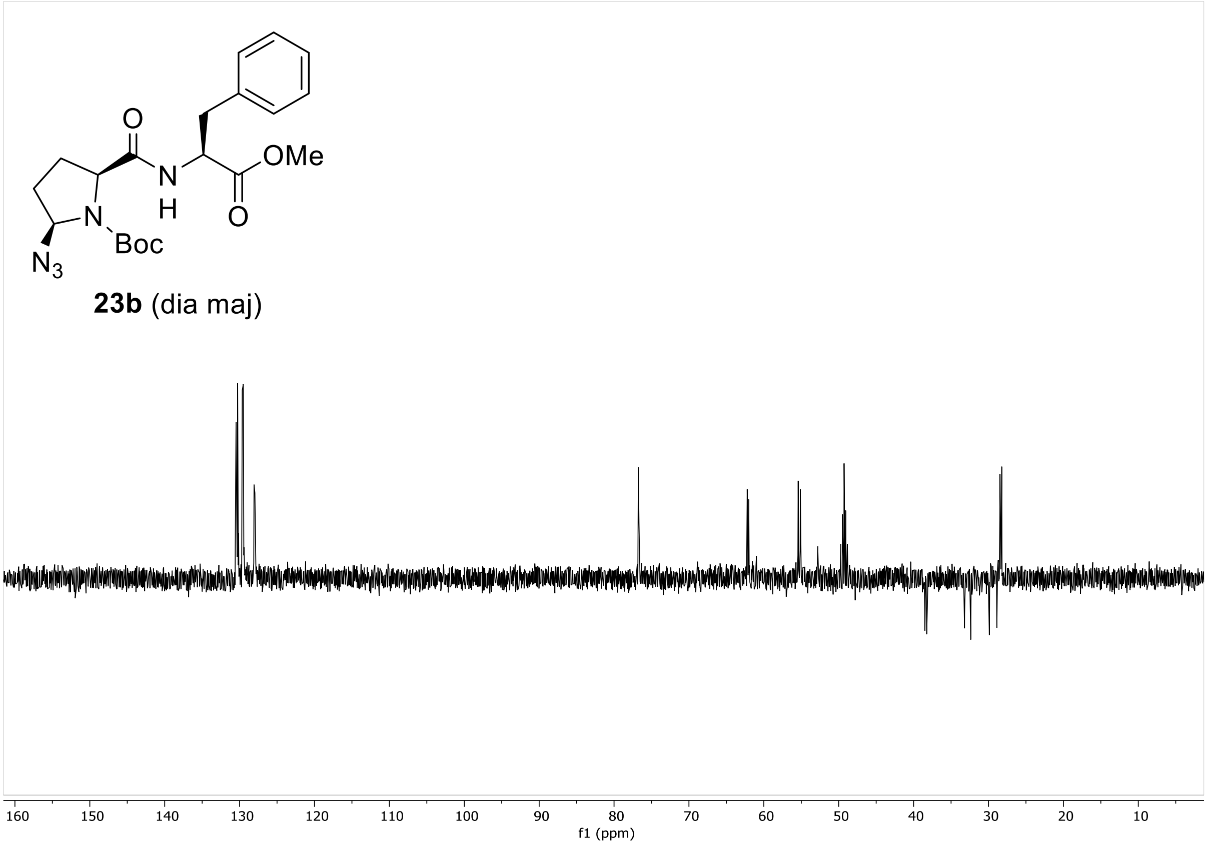

NOESY (400 MHz, MeOD-*d₄*, 298 K, complex mixture of rotamers) of compound **23b**

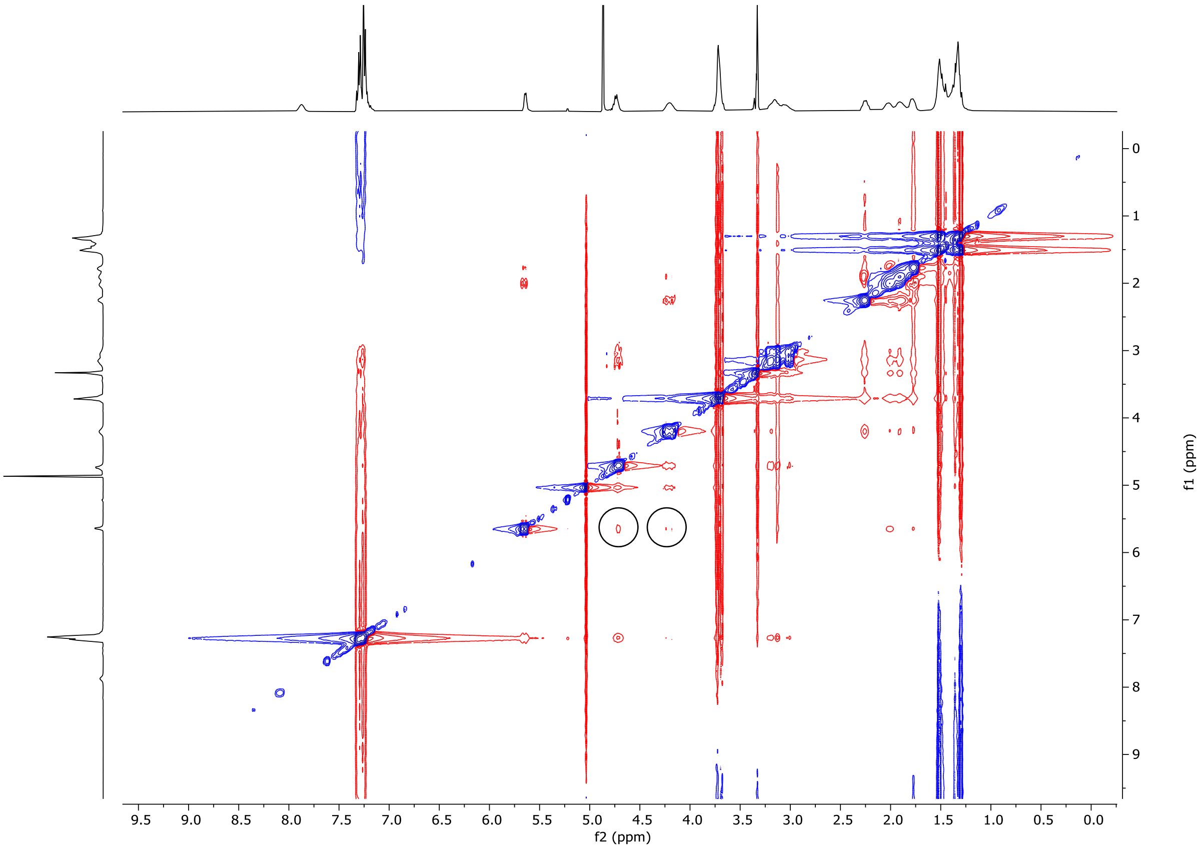

^{1}H NMR (400 MHz, MeOD-*d$_4$*, 298 K, mixture of two rotamers) of compound **24a**

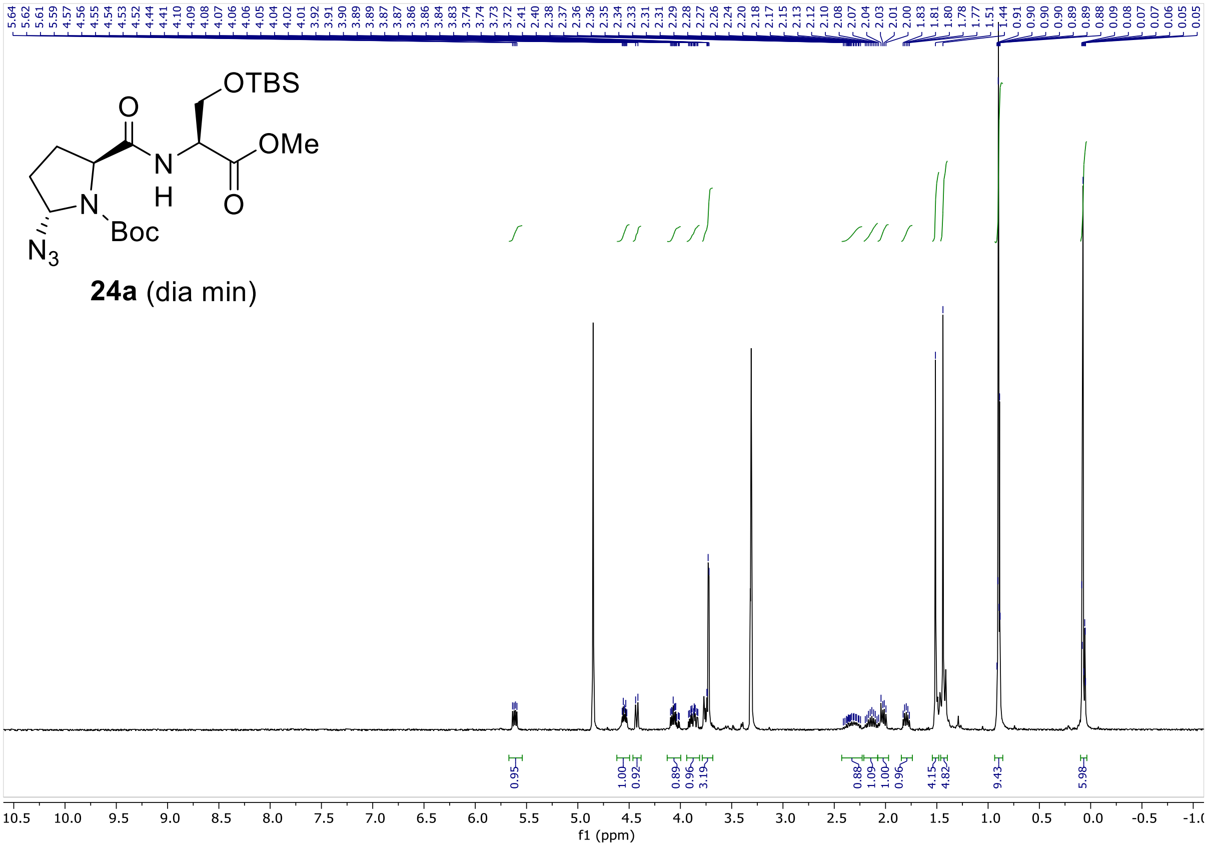

^{13}C NMR (101 MHz, MeOD-*d$_4$*, 278.2 K, mixture of two rotamers) of compound **24a**

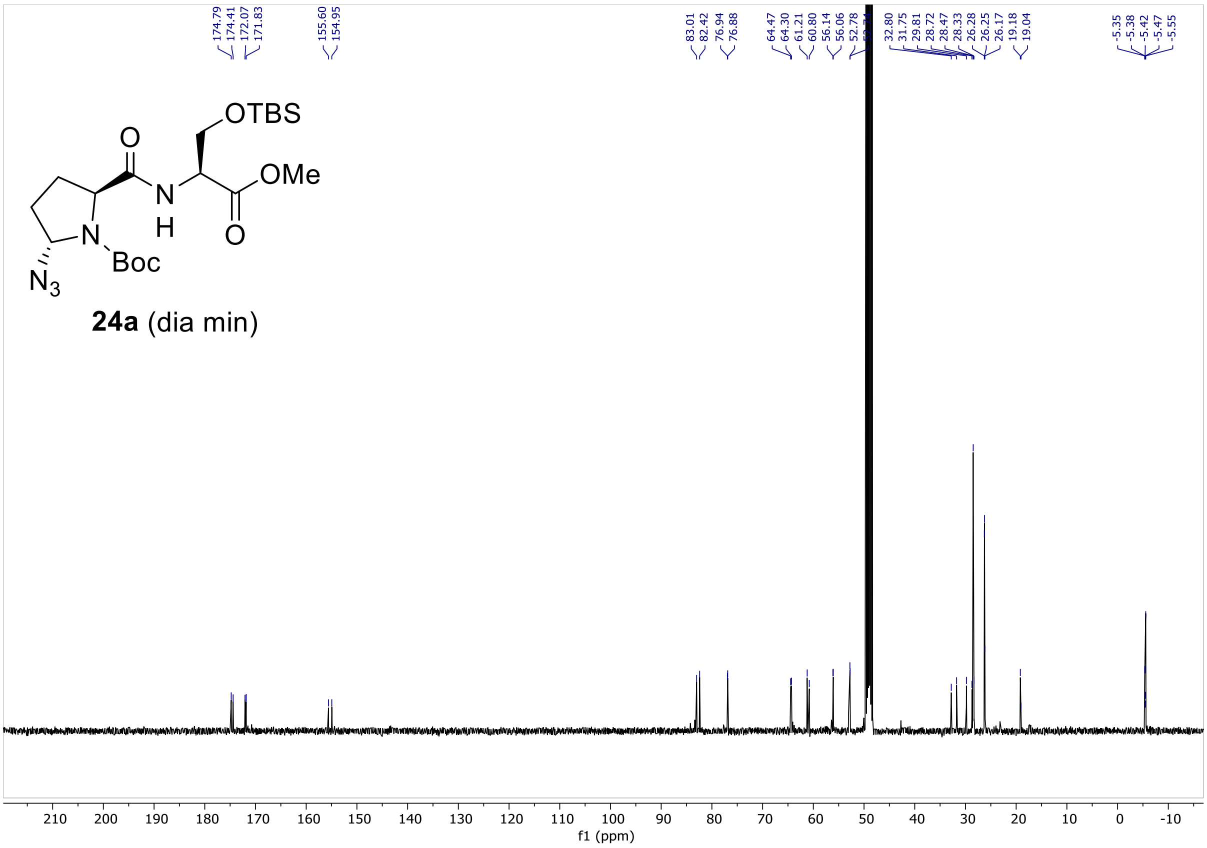

HSQC (MeOD-*d*$_4$, 278.2 K, mixture of two rotamers) of compound **24a**

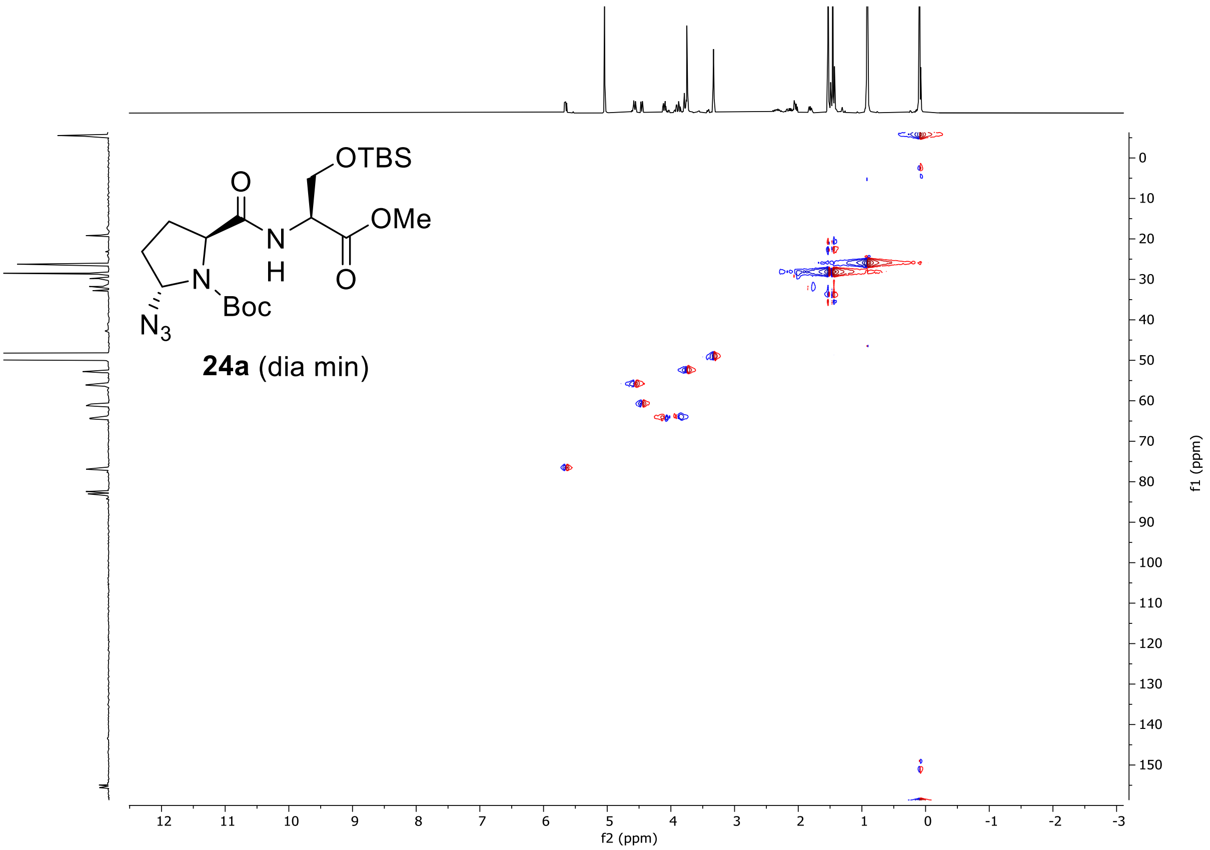

DEPT-135 (101 MHz, MeOD-*d*$_4$, 278.2 K, mixture of two rotamers) of compound **24a**

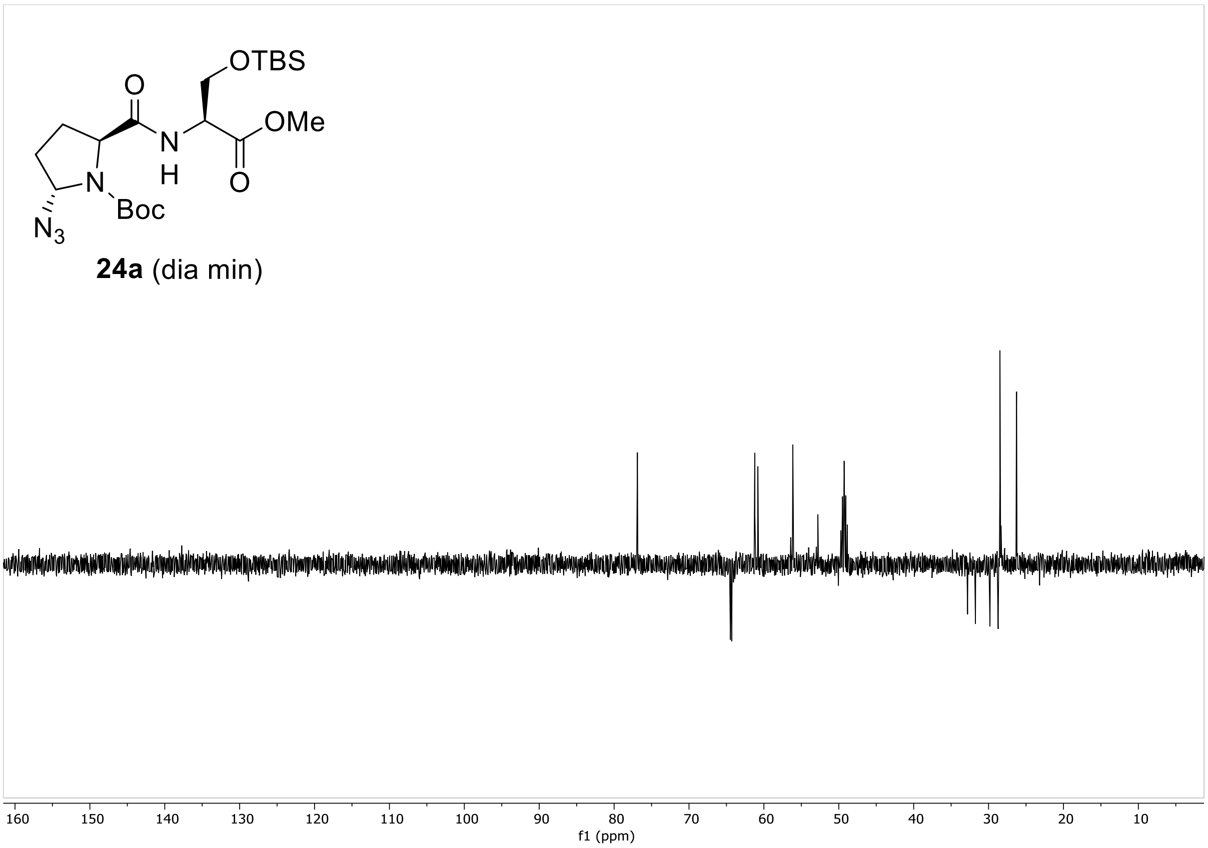

^{1}H NMR (400 MHz, MeOD-*d*$_4$, 298 K, complex mixture of rotamers) of compound **24b**

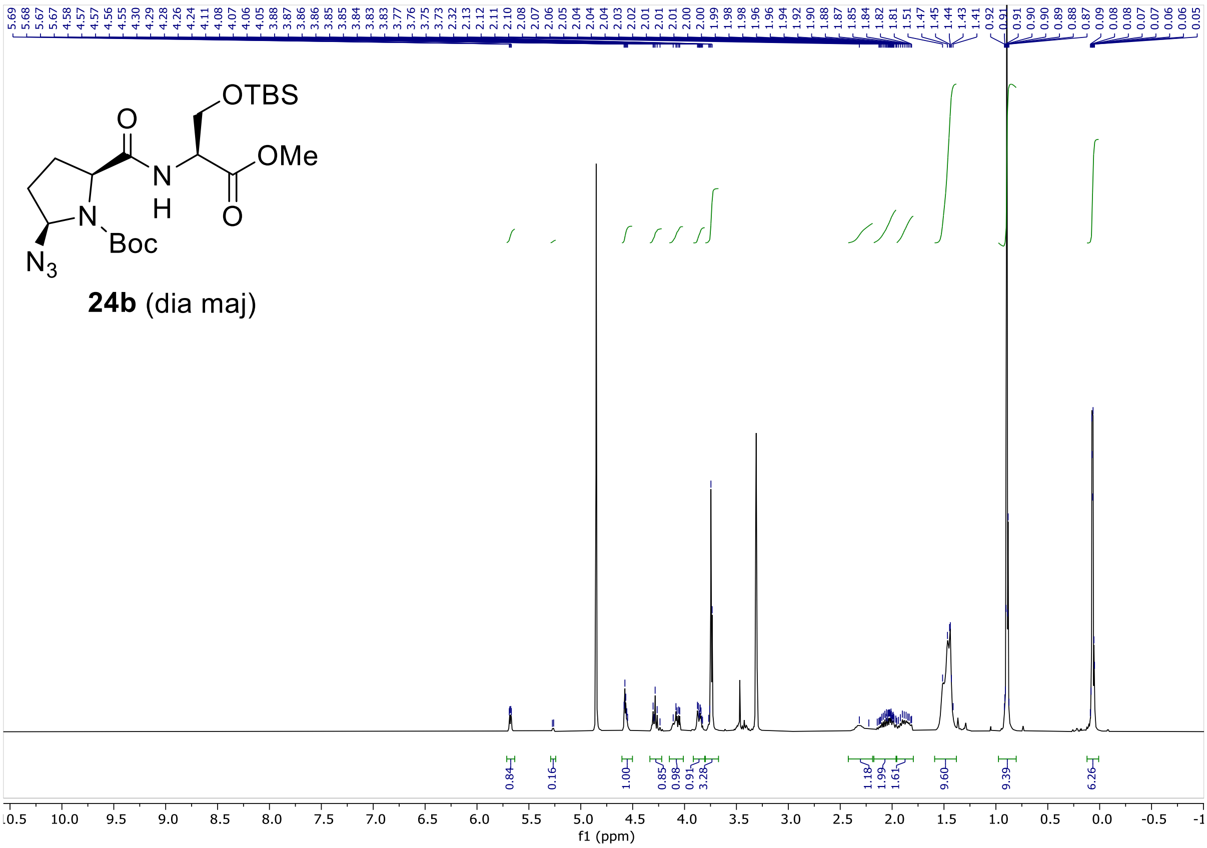

^{13}C NMR (101 MHz, MeOD-*d*$_4$, 278.2 K, complex mixture of rotamers) of compound **24b**

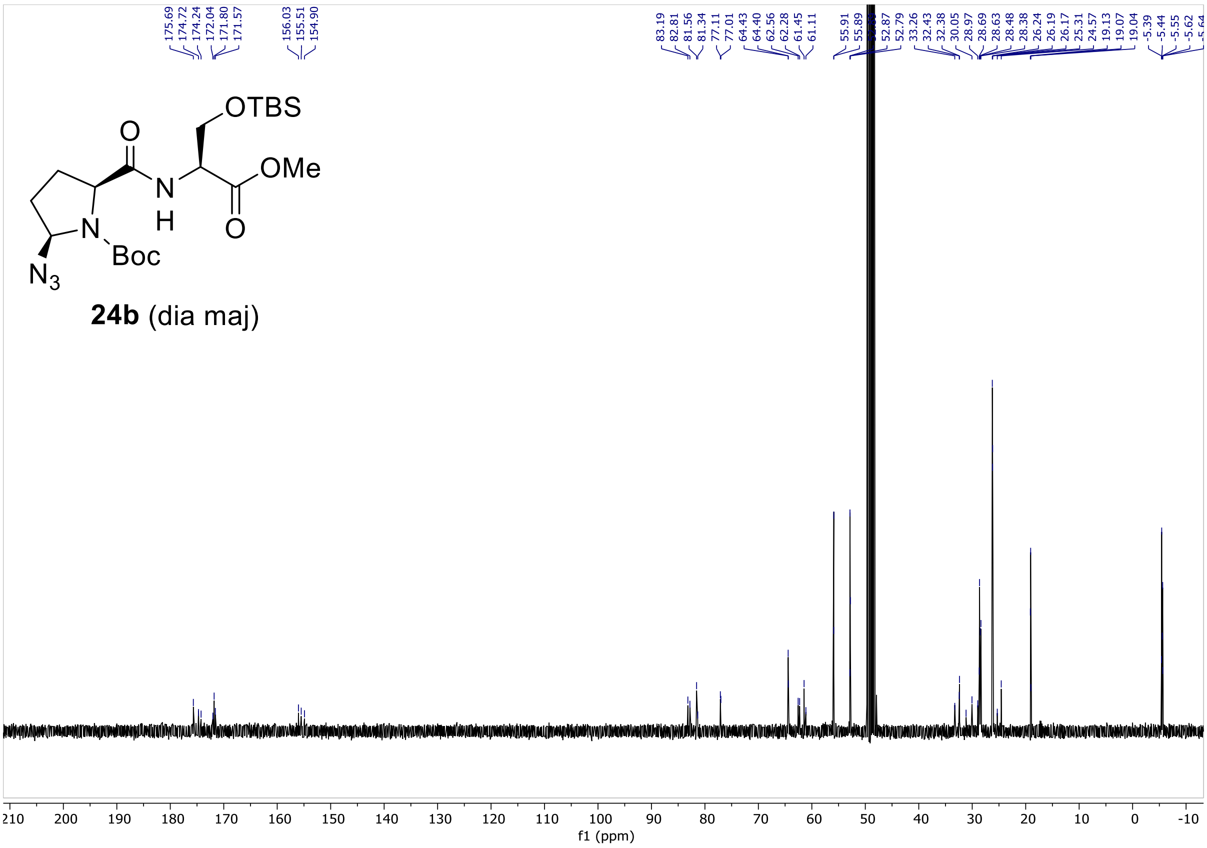

HSQC (MeOD-d_4, 278.2 K, complex mixture of rotamers) of compound **24b**

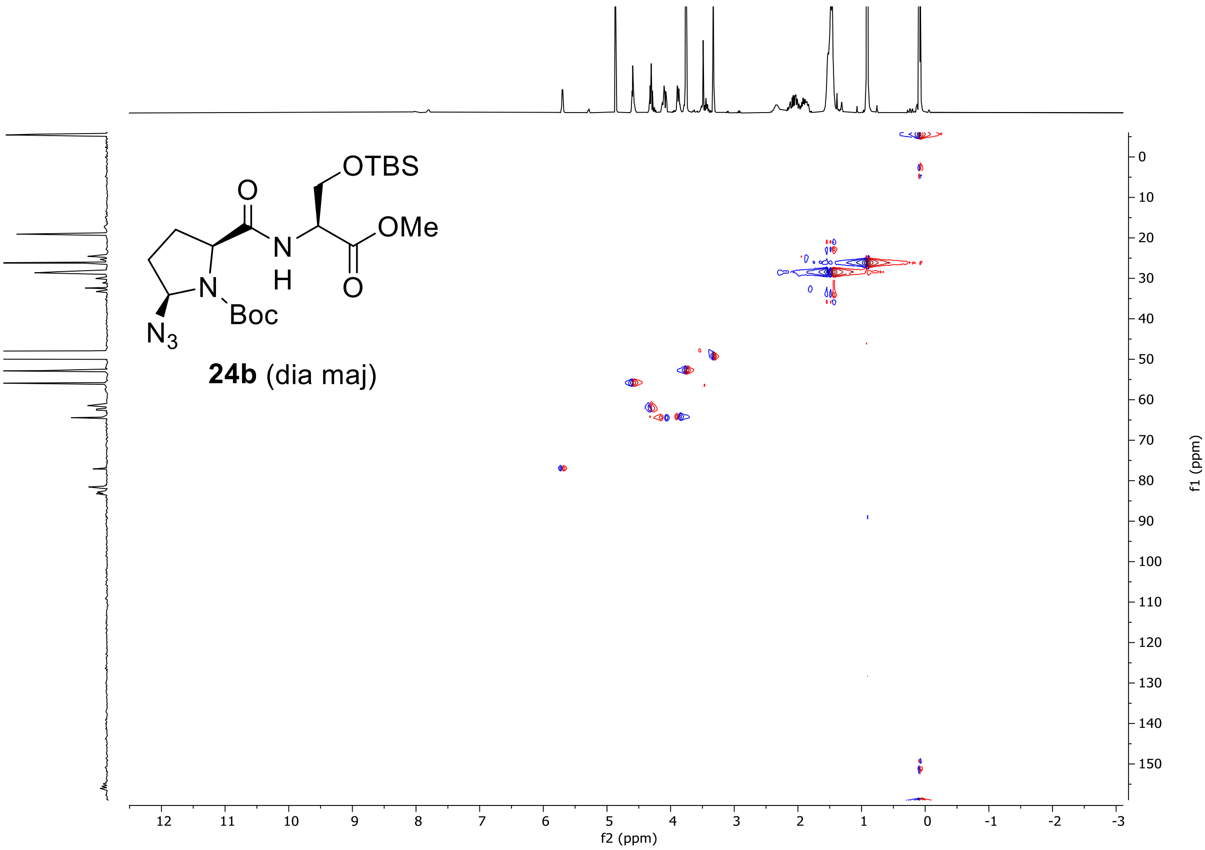

DEPT-135 (101 MHz, MeOD-d_4, 278.2 K, complex mixture of rotamers) of compound **24b**

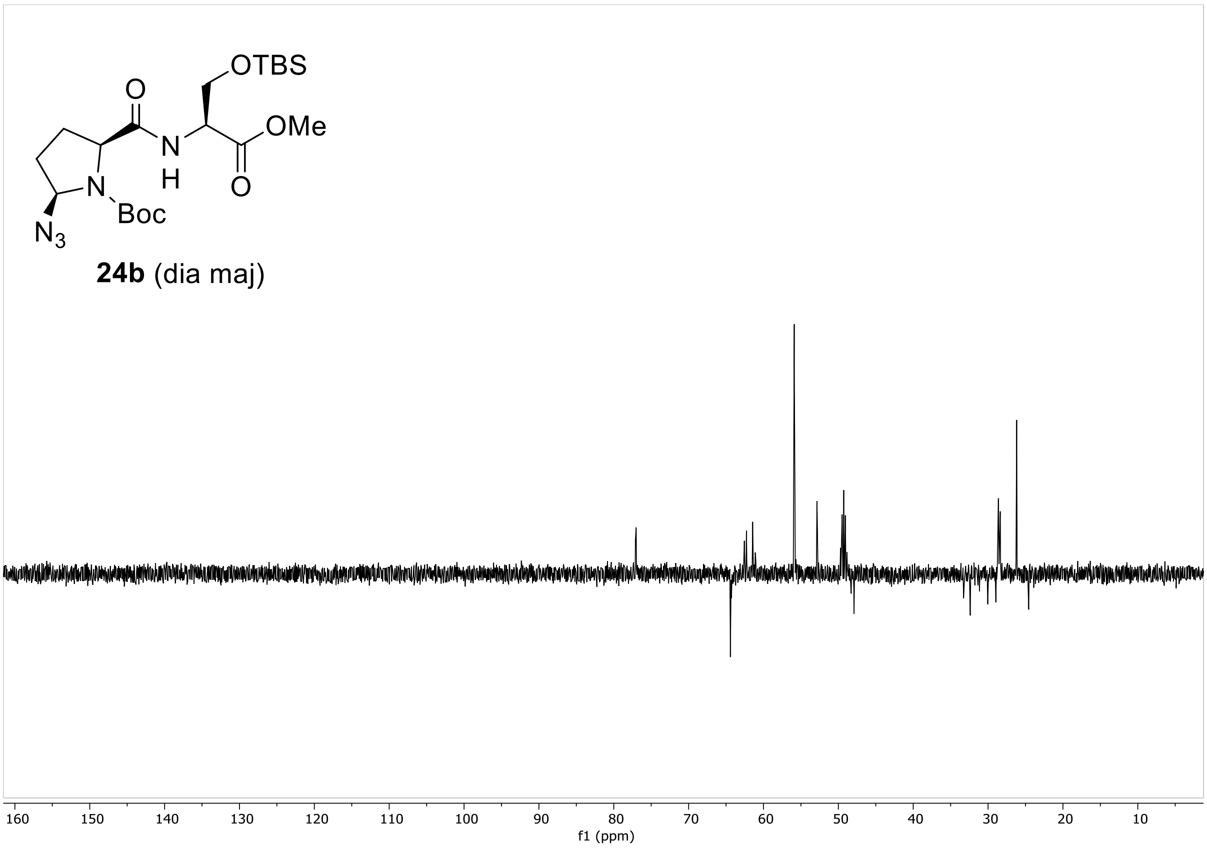

NOESY (400 MHz, MeOD-d_4, 298 K, complex mixture of rotamers) of compound **24b**

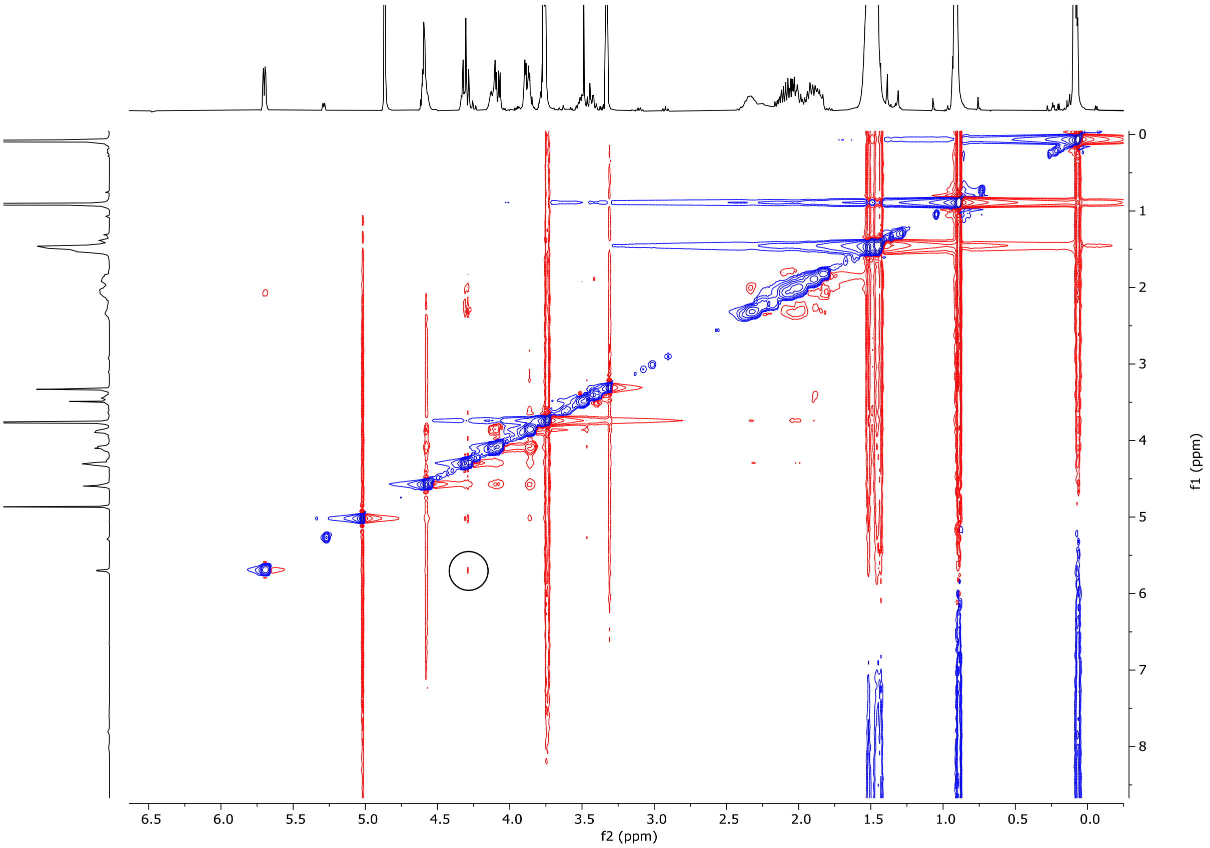

^{1}H NMR (400 MHz, MeOD-d_4, 278.2 K, mixture of two rotamers) of compound **25a**

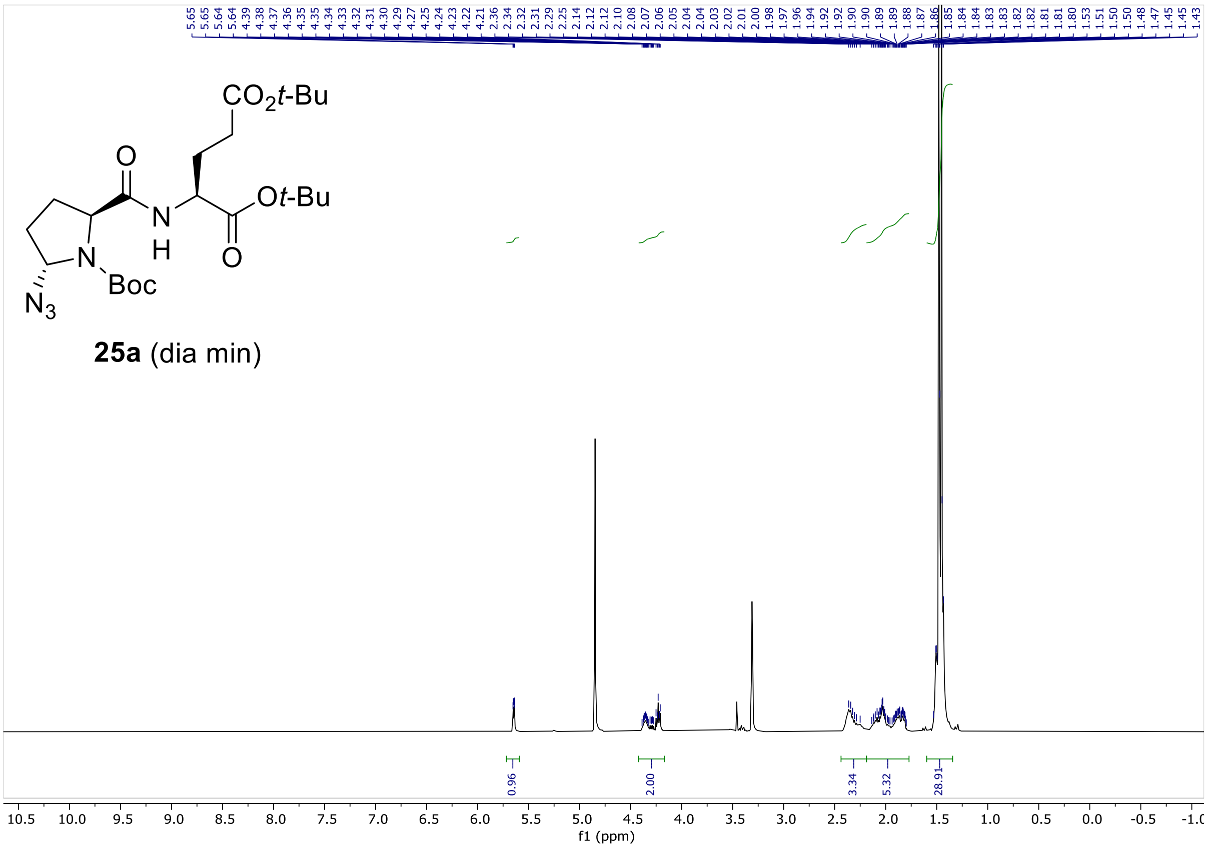

^{13}C NMR (101 MHz, MeOD-d_4, 278.2 K, mixture of two rotamers) of compound **25a**

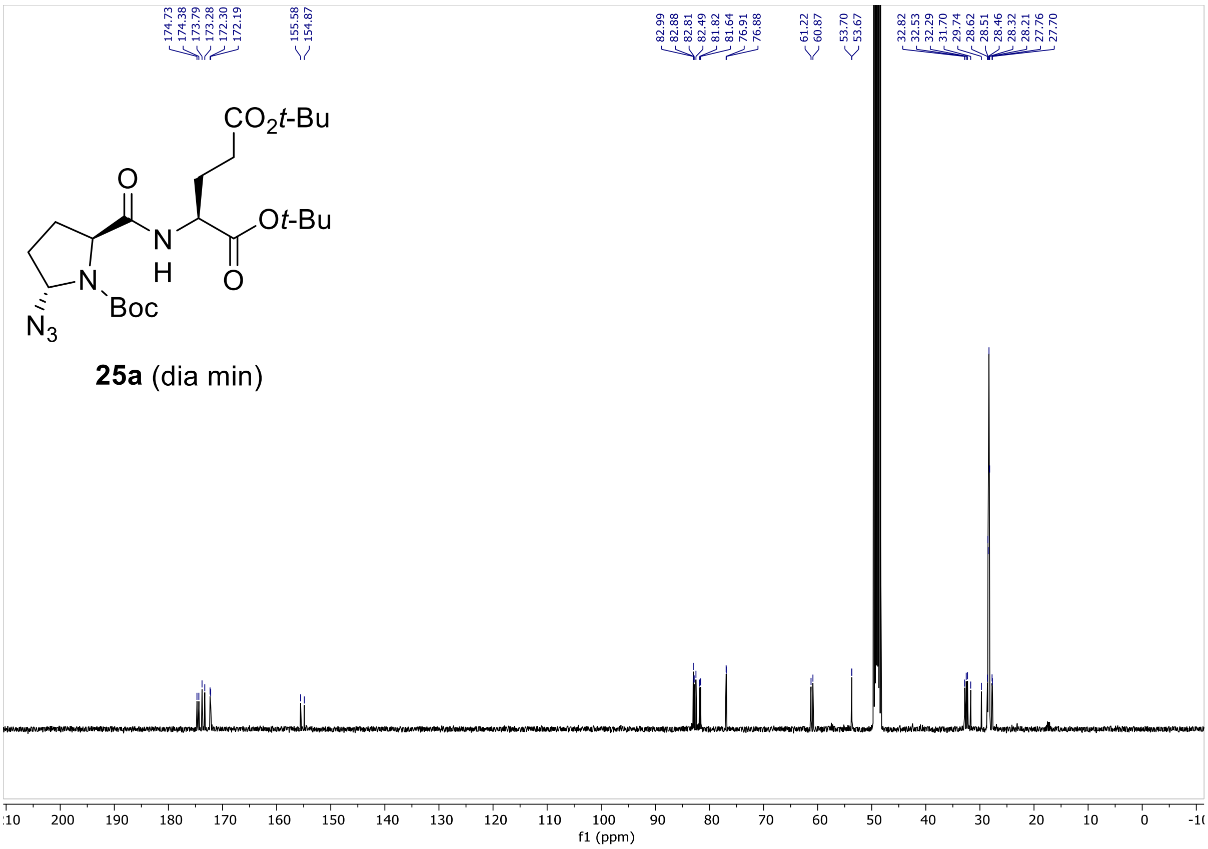

HSQC (MeOD-d_4, 278.2 K, mixture of two rotamers) of compound **25a**

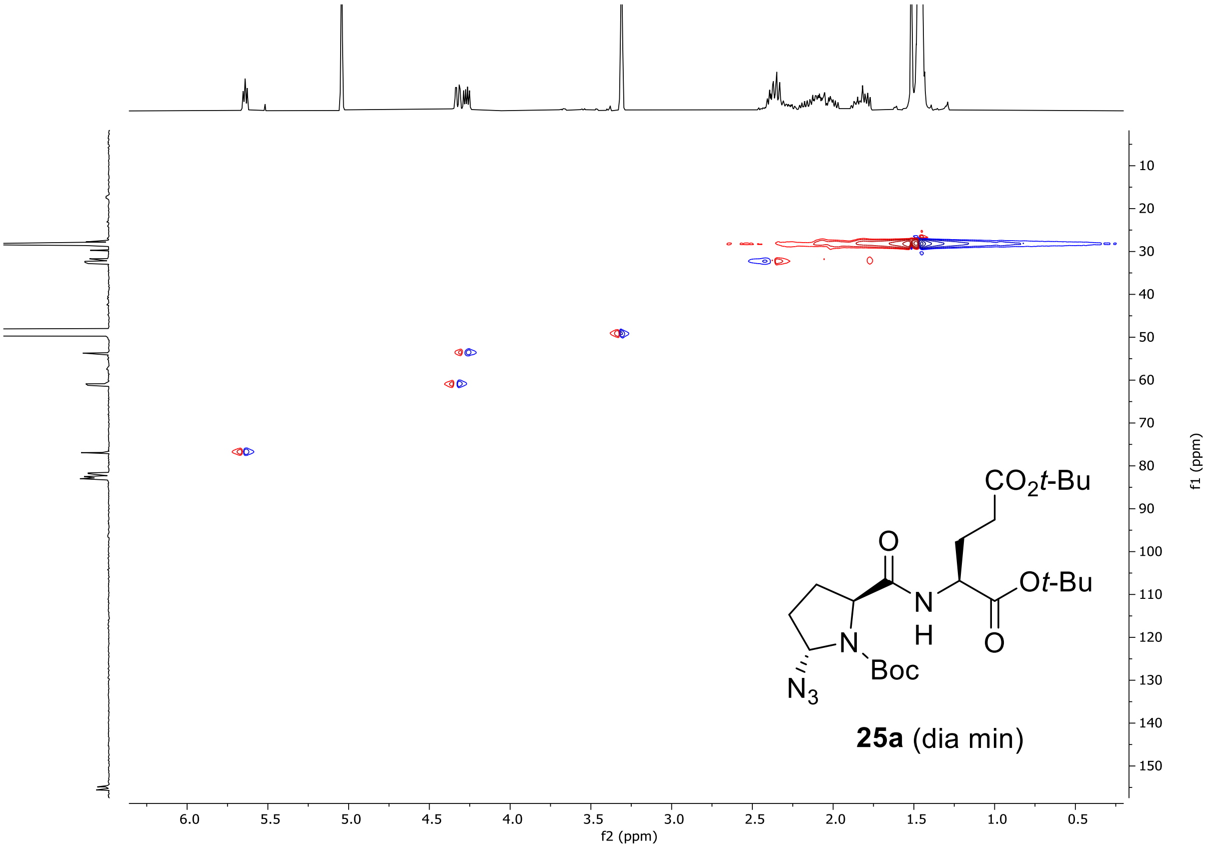

^{1}H NMR (400 MHz, MeOD-d_4, 278.2 K, complex mixture of rotamers) of compound **25b**

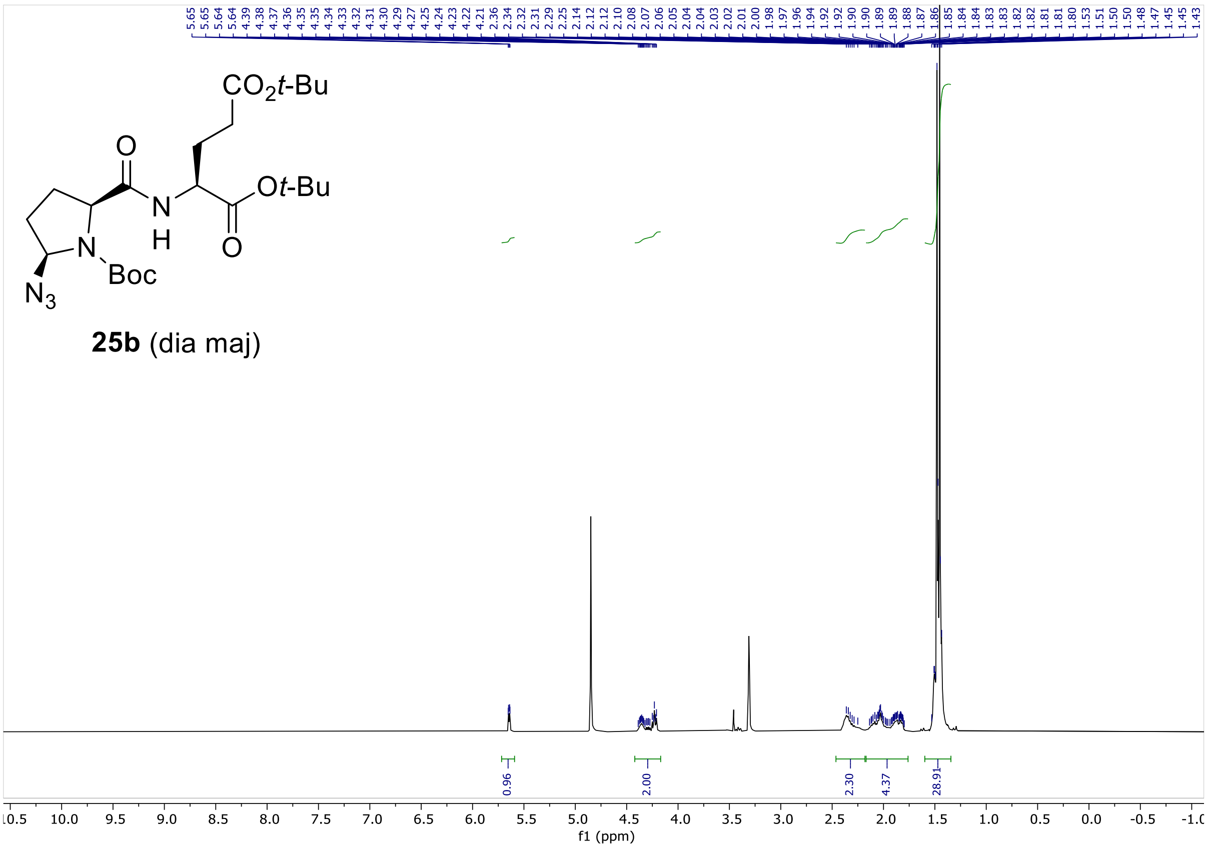

^{13}C NMR (101 MHz, MeOD-d_4, 278.2 K, complex mixture of rotamers) of compound **25b**

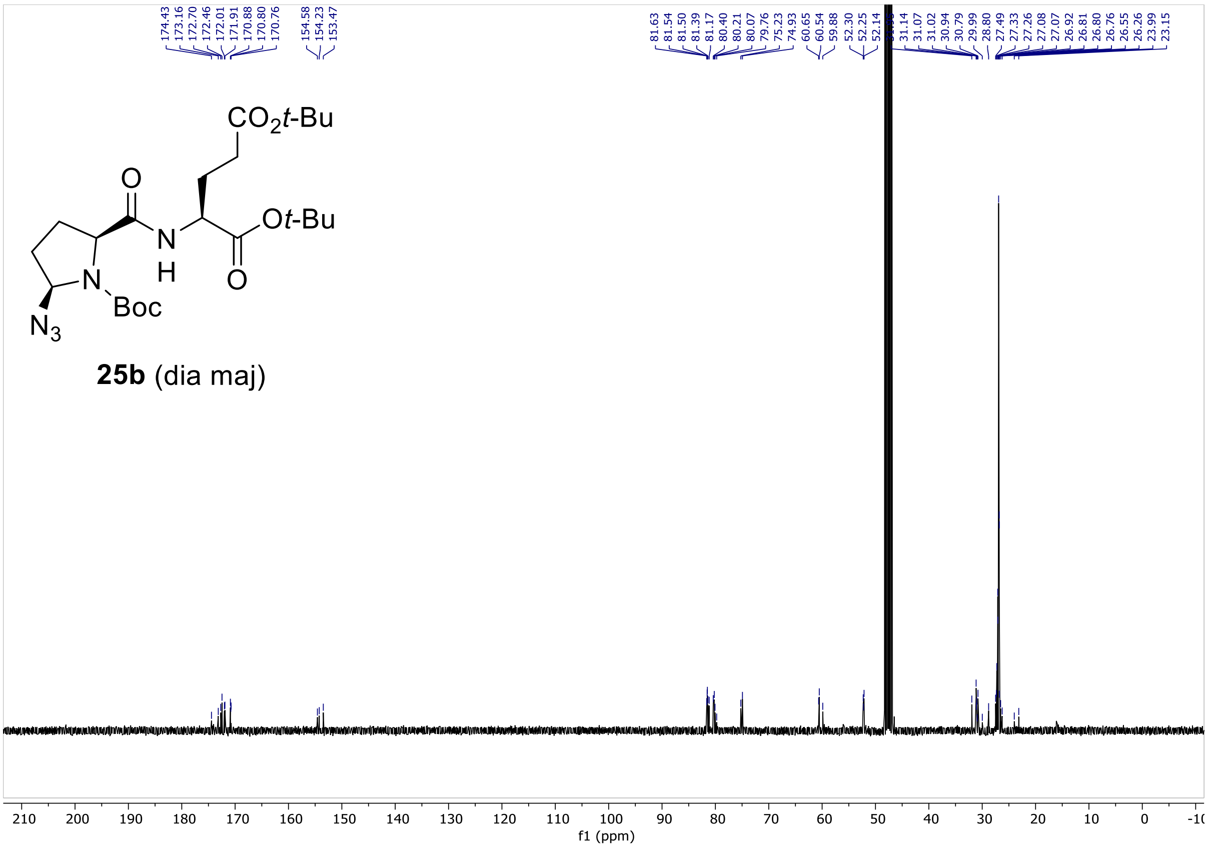

HSQC (MeOD-*d*₄, 278.2 K, complex mixture of rotamers) of compound **25b**

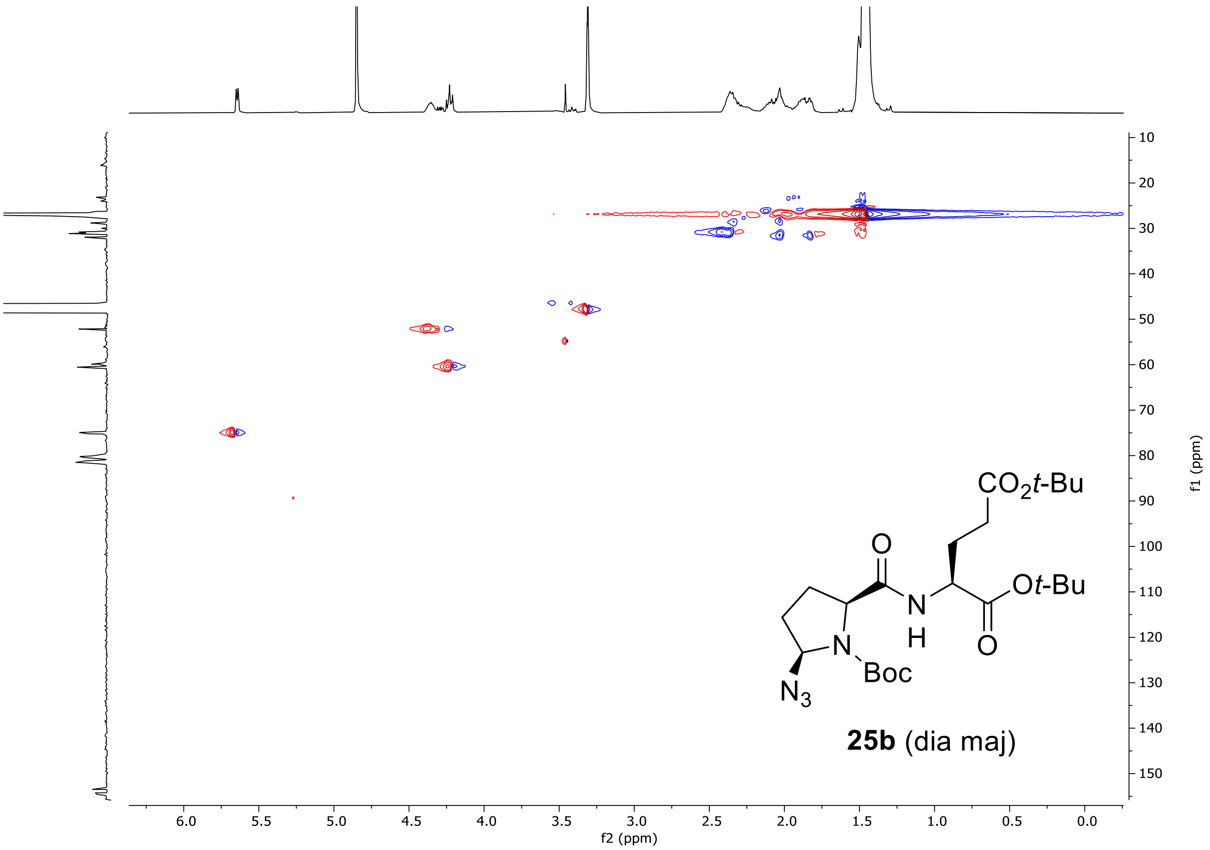

NOESY (400 MHz, MeOD-*d*₄, 278.2 K, complex mixture rotamers) of compound **25b**

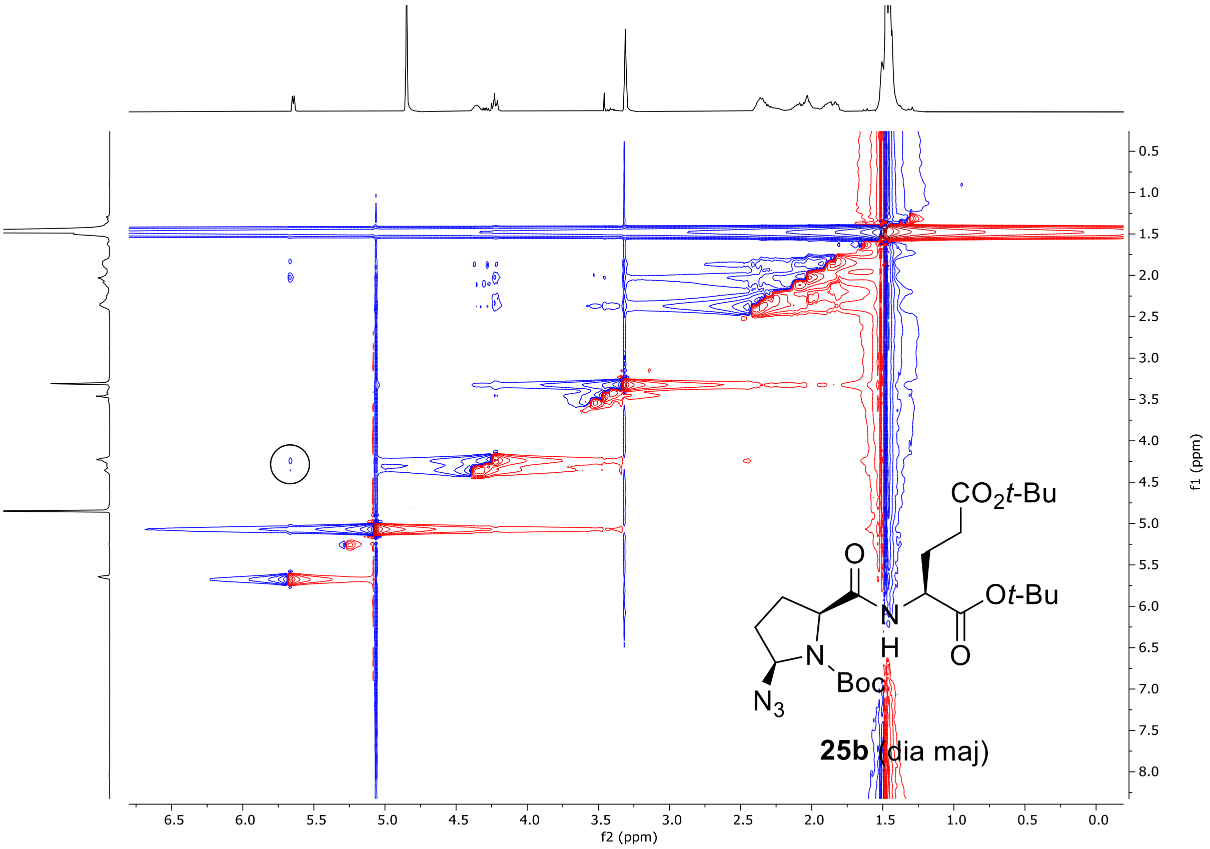

^{1}H NMR (400 MHz, MeOD-d_4, 298 K, complex mixture of diastereoisomers and rotamers) of **26**

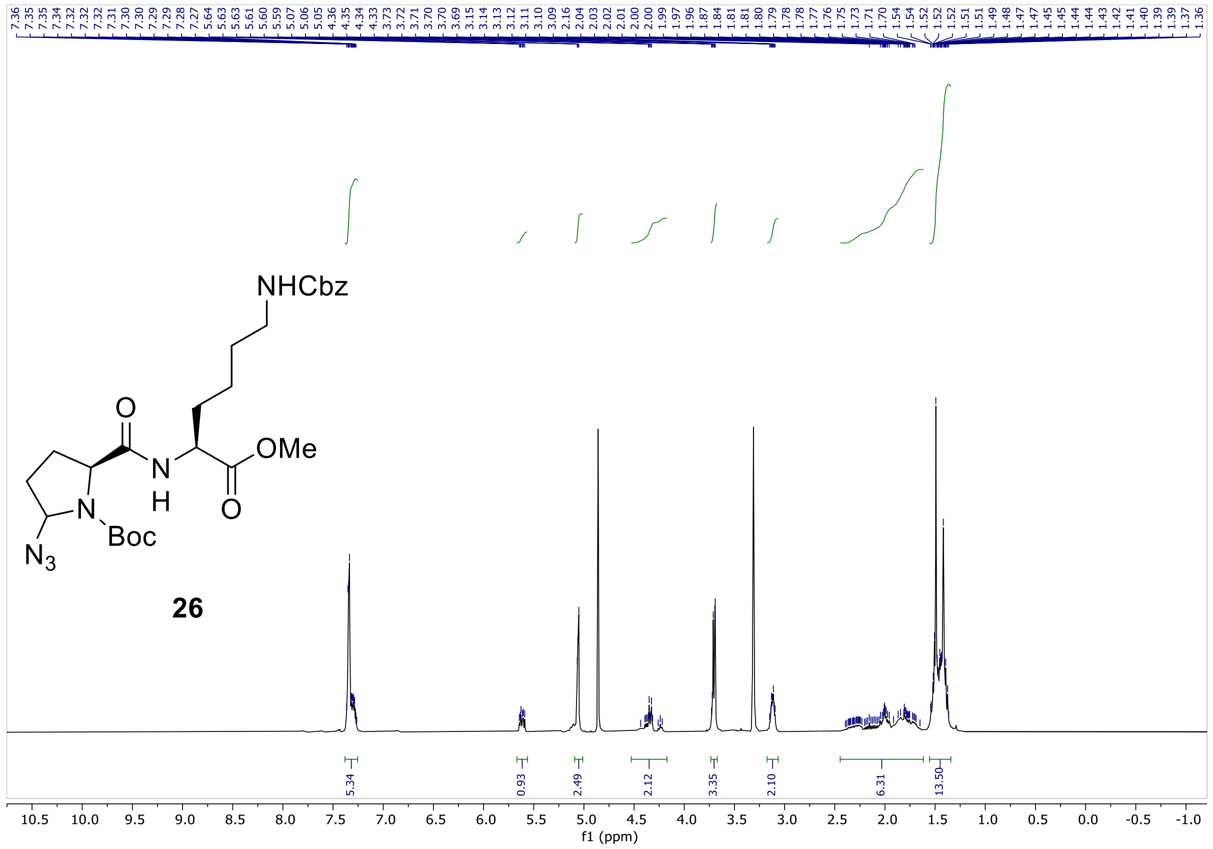

^{13}C NMR (101 MHz, MeOD-d_4, 278.2 K, complex mixture of diastereoisomers and rotamers) of **26**

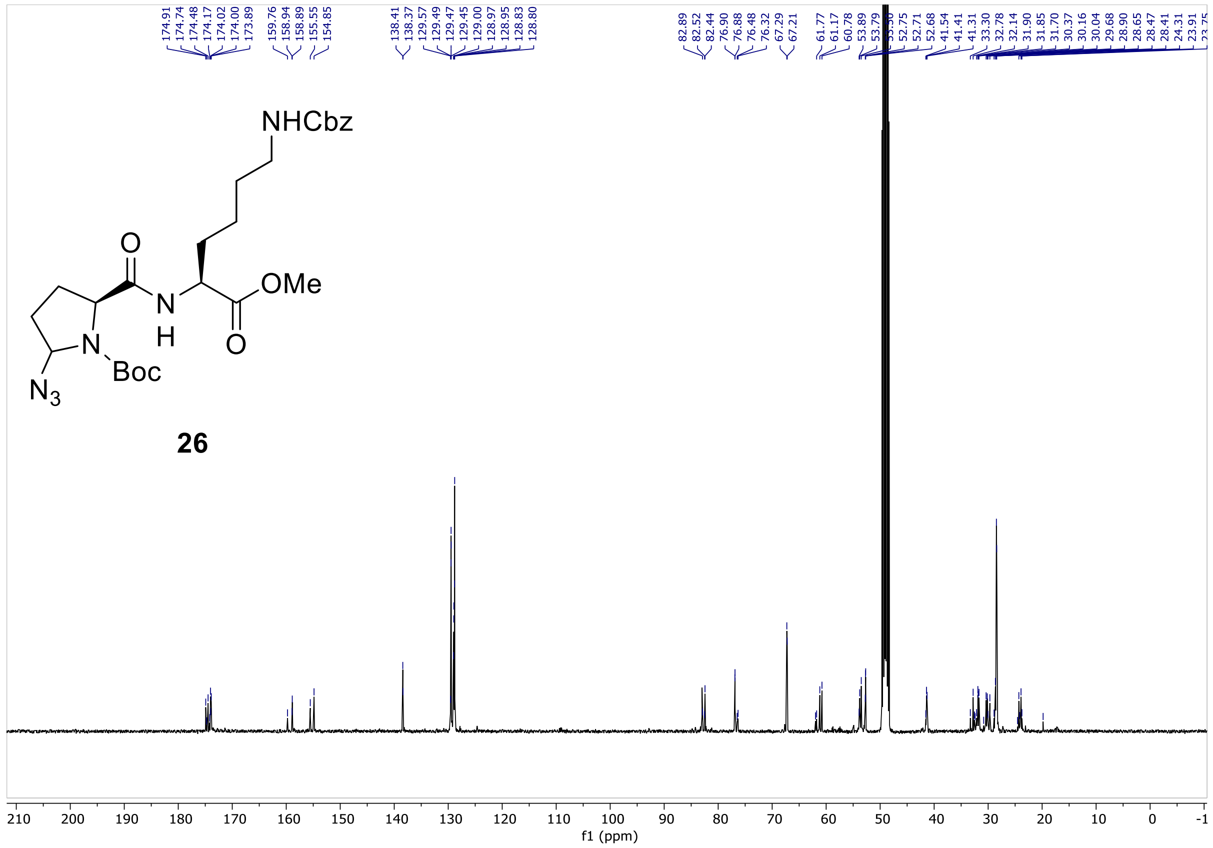

DEPT-135 (101 MHz, MeOD-d_4, 278.2 K, complex mixture of diastereoisomers and rotamers) of **26**

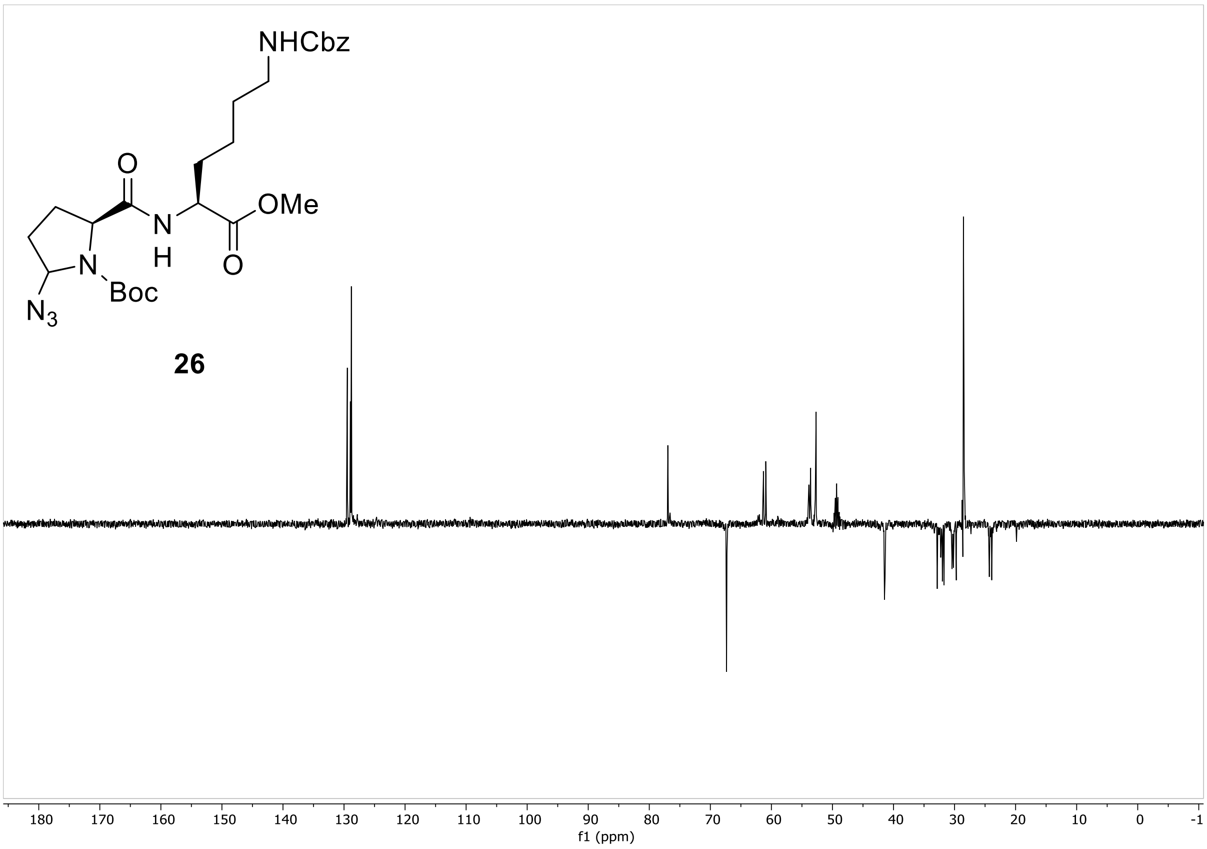

^{1}H NMR (400 MHz, MeOD-d_4, 298 K, complex mixture of diastereoisomers and rotamers) of **27**

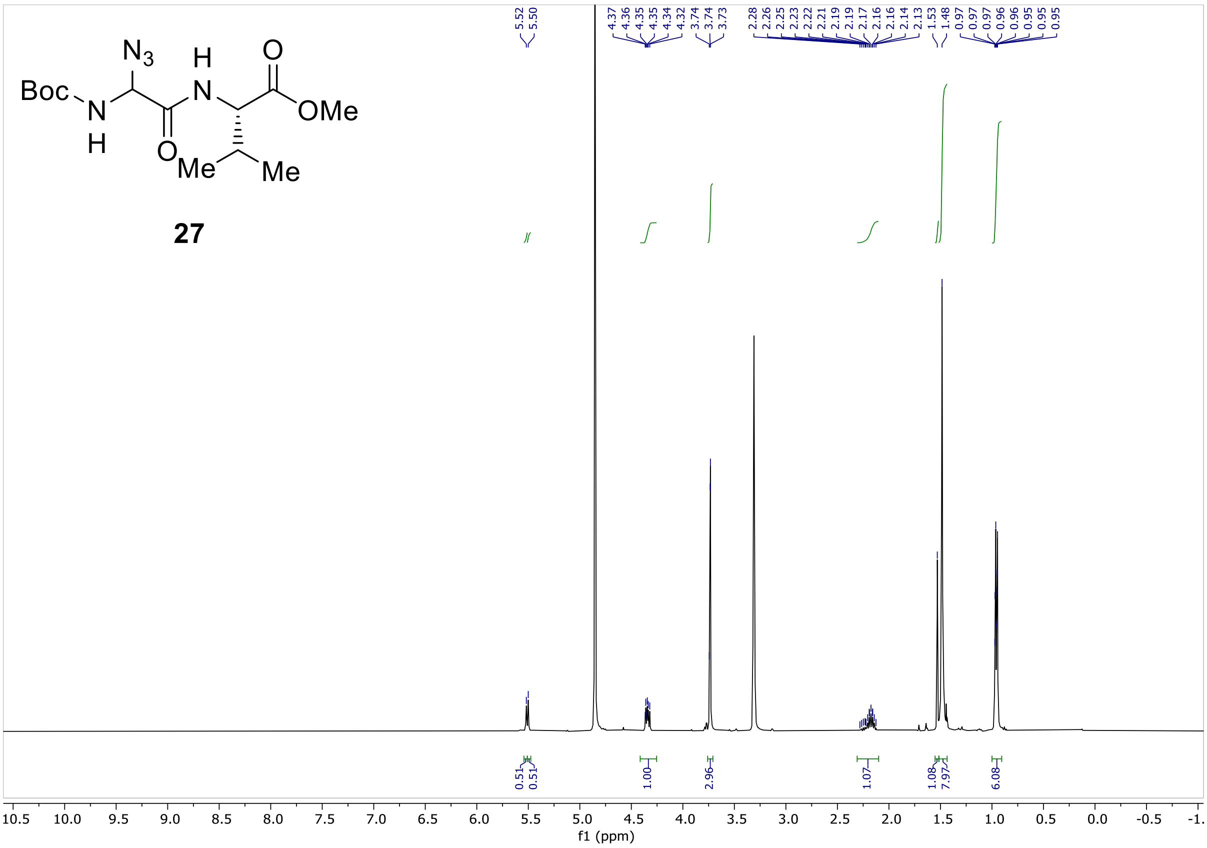

^{13}C NMR (101 MHz, $CDCl_3$, 298 K, complex mixture of diastereoisomers and rotamers) of **27**

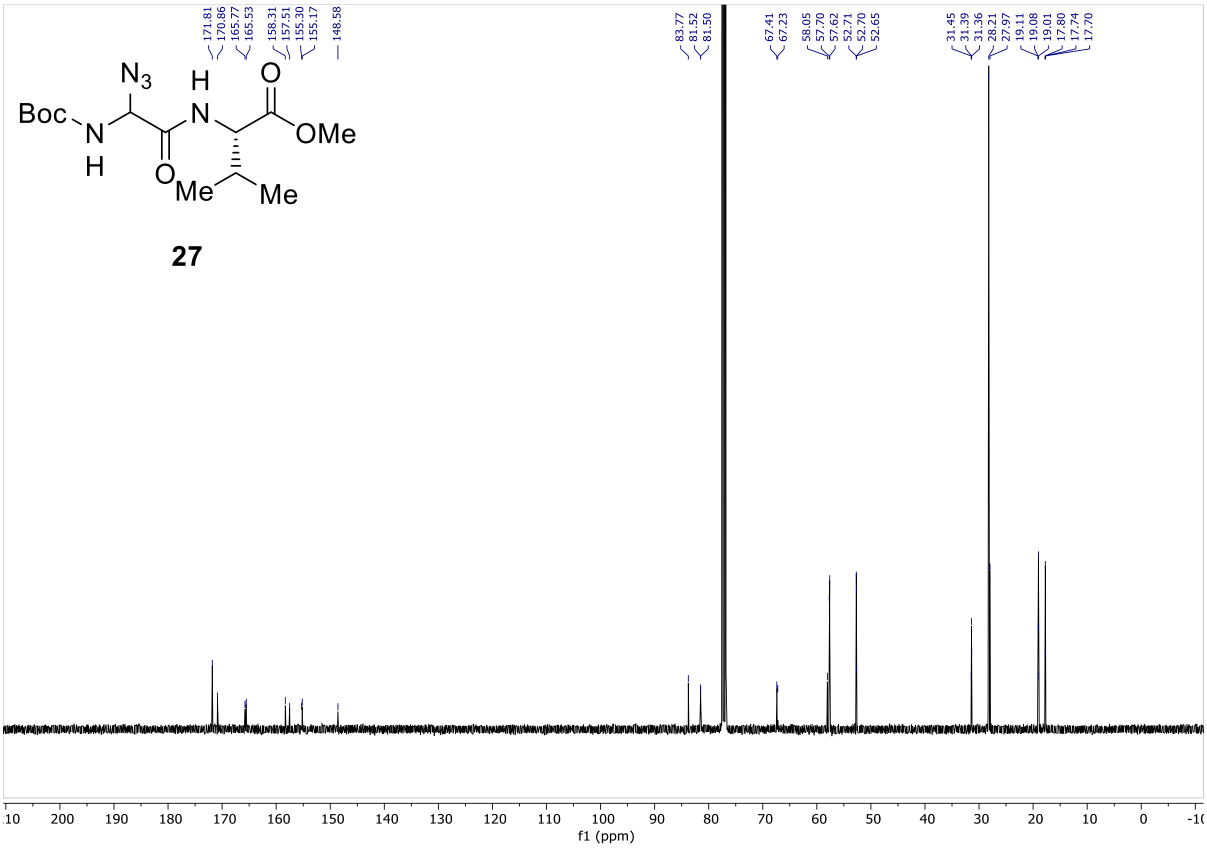

DEPT-135 (101 MHz, $CDCl_3$, 298 K, complex mixture of diastereoisomers and rotamers) of **27**

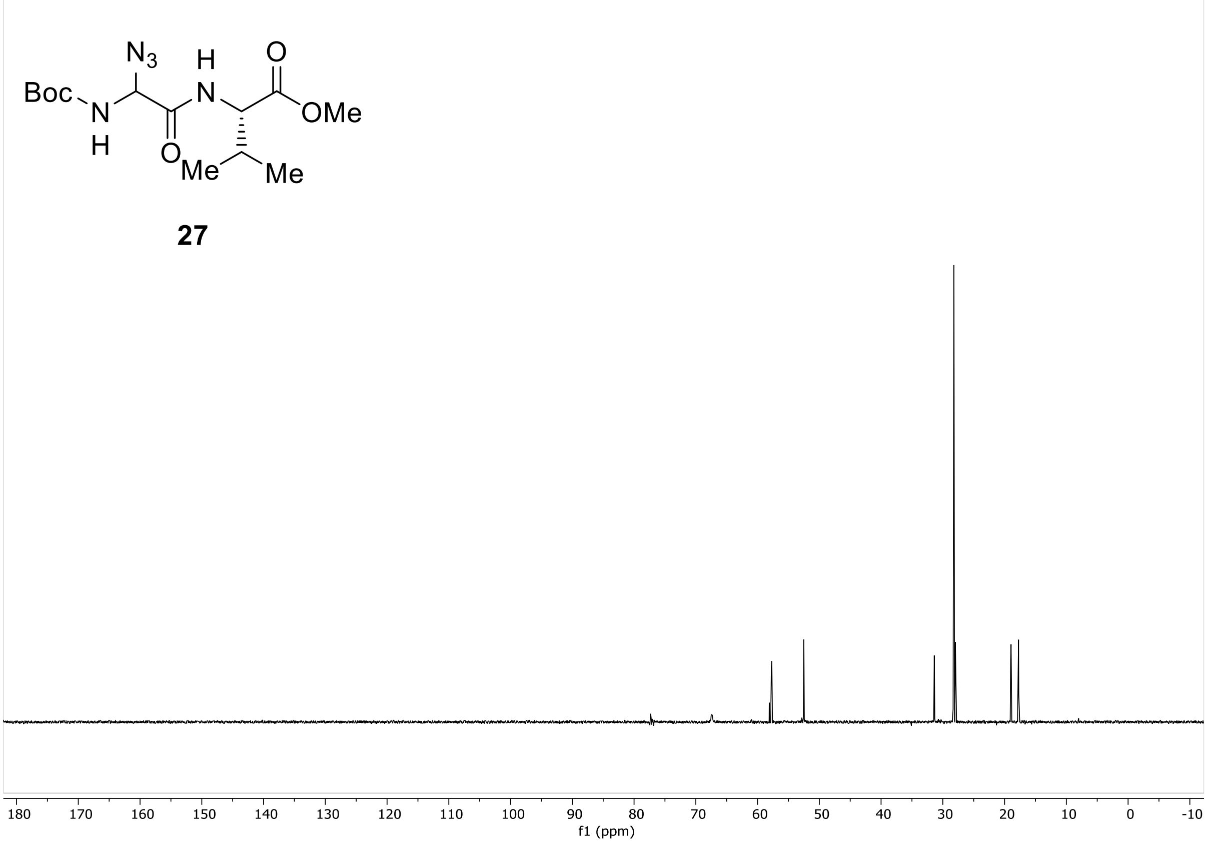

^{1}H NMR (400 MHz, MeOD-d_4, 298 K, complex mixture of diastereoisomers and rotamers) of **28**

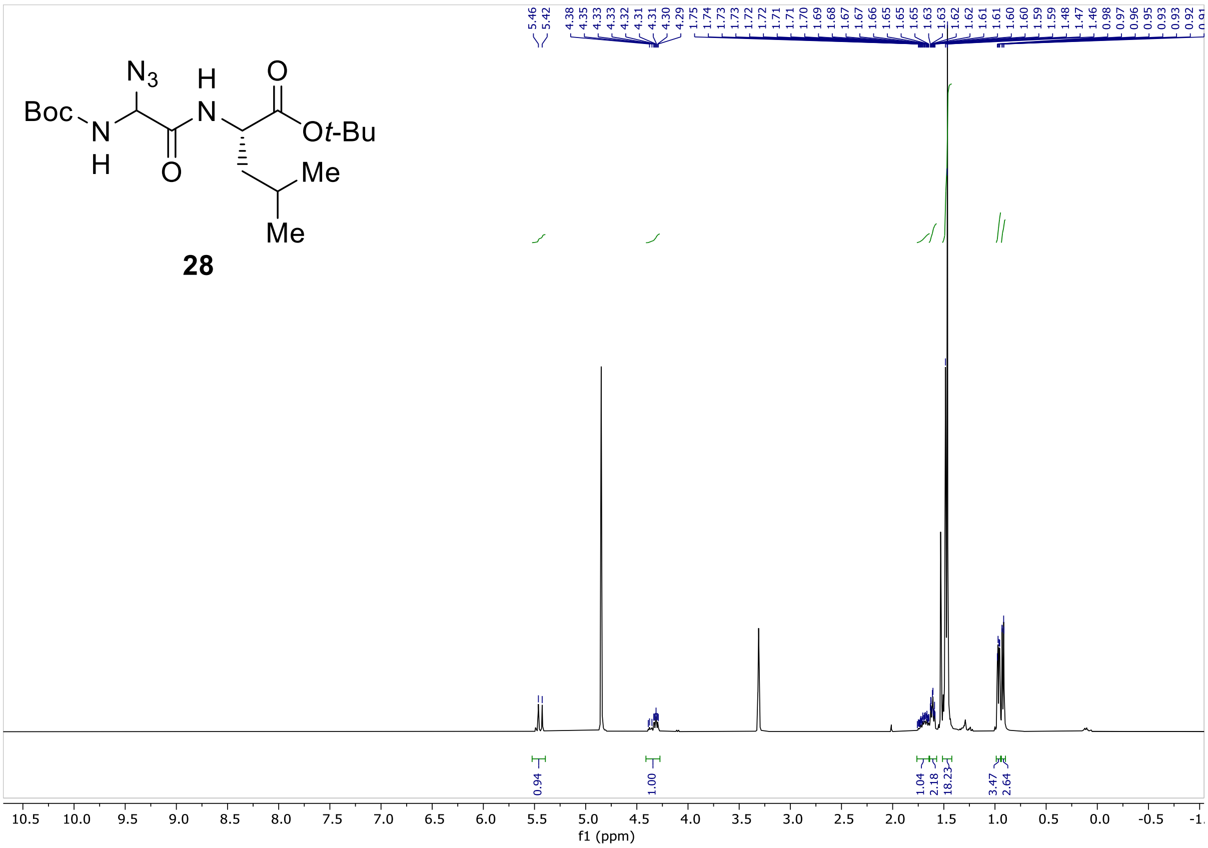

^{13}C NMR (101 MHz, $CDCl_3$, 298 K, complex mixture of diastereoisomers and rotamers) of **28**

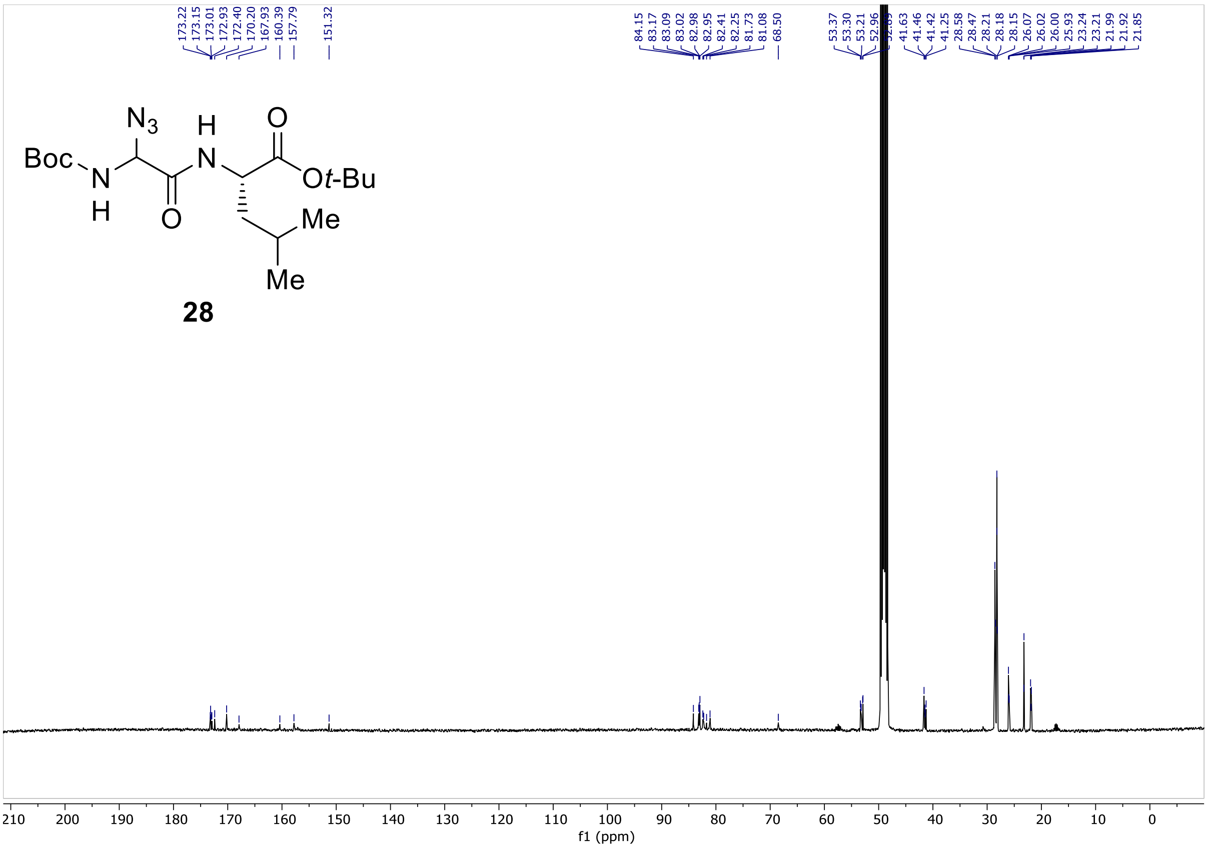

DEPT-135 (101 MHz, $CDCl_3$, 298 K, complex mixture of diastereoisomers and rotamers) of **28**

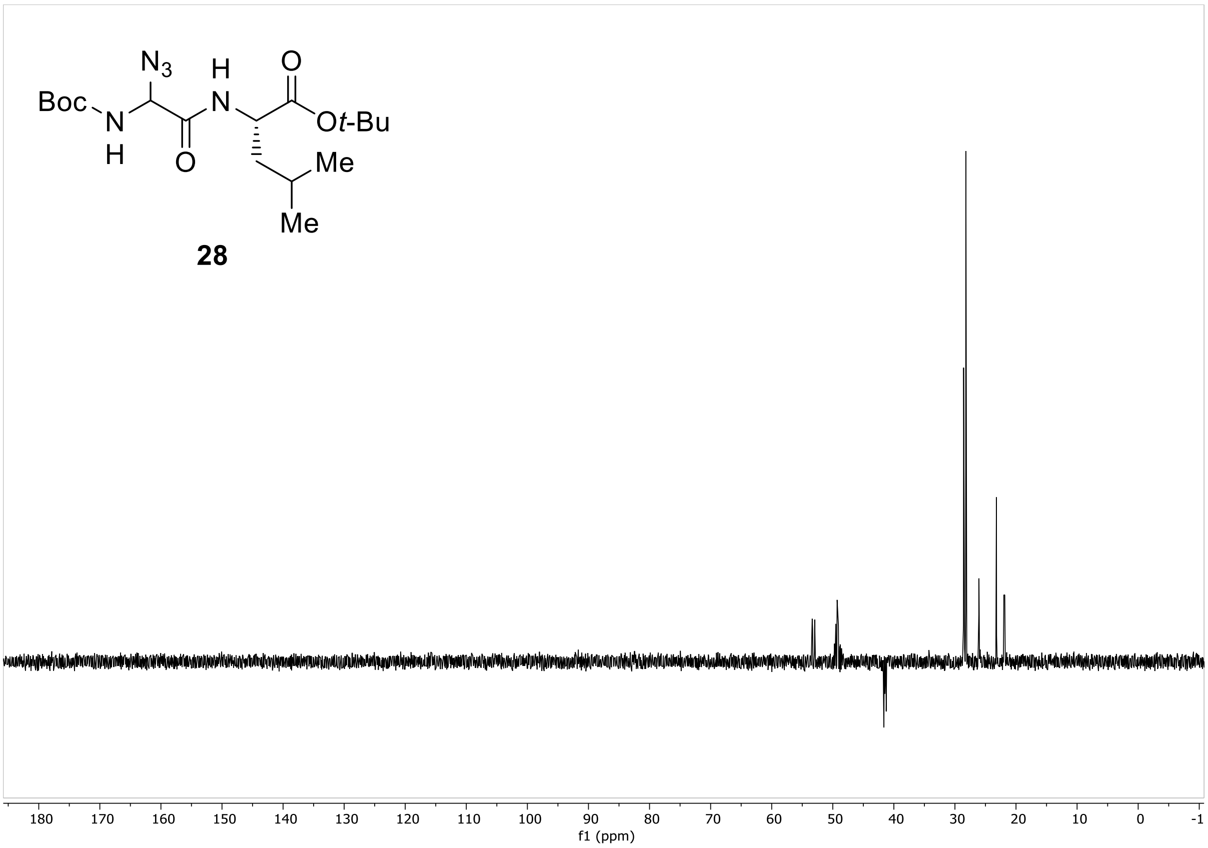

^{1}H NMR (400 MHz, MeOD-d_4, 278.2 K, complex mixture of diastereoisomers and rotamers) of compound **29**

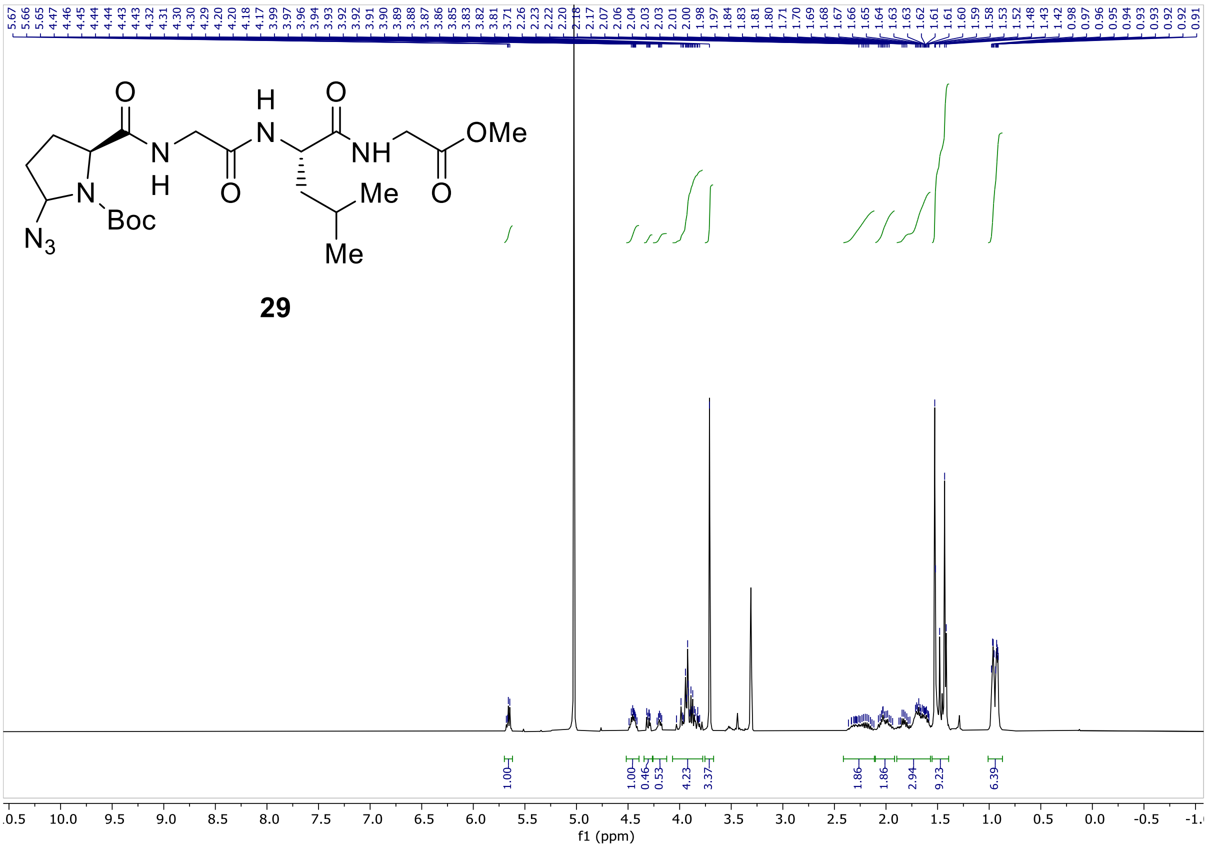

^{13}C NMR (101 MHz, MeOD-d_4, 278.2 K, complex mixture of diastereoisomers and rotamers) of compound **29**

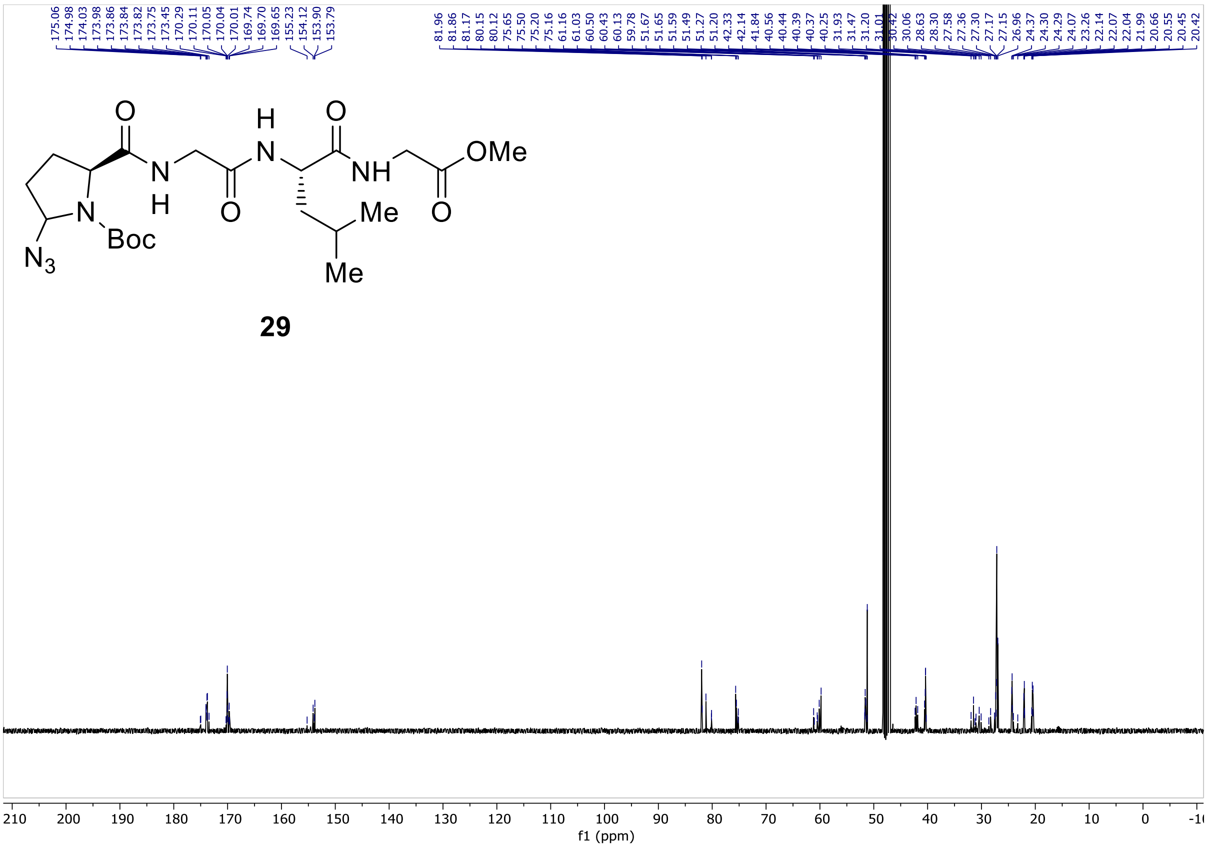

DEPT-135 (101 MHz, MeOD-d_4, 278.2 K, complex mixture of diastereoisomers and rotamers) of compound **29**

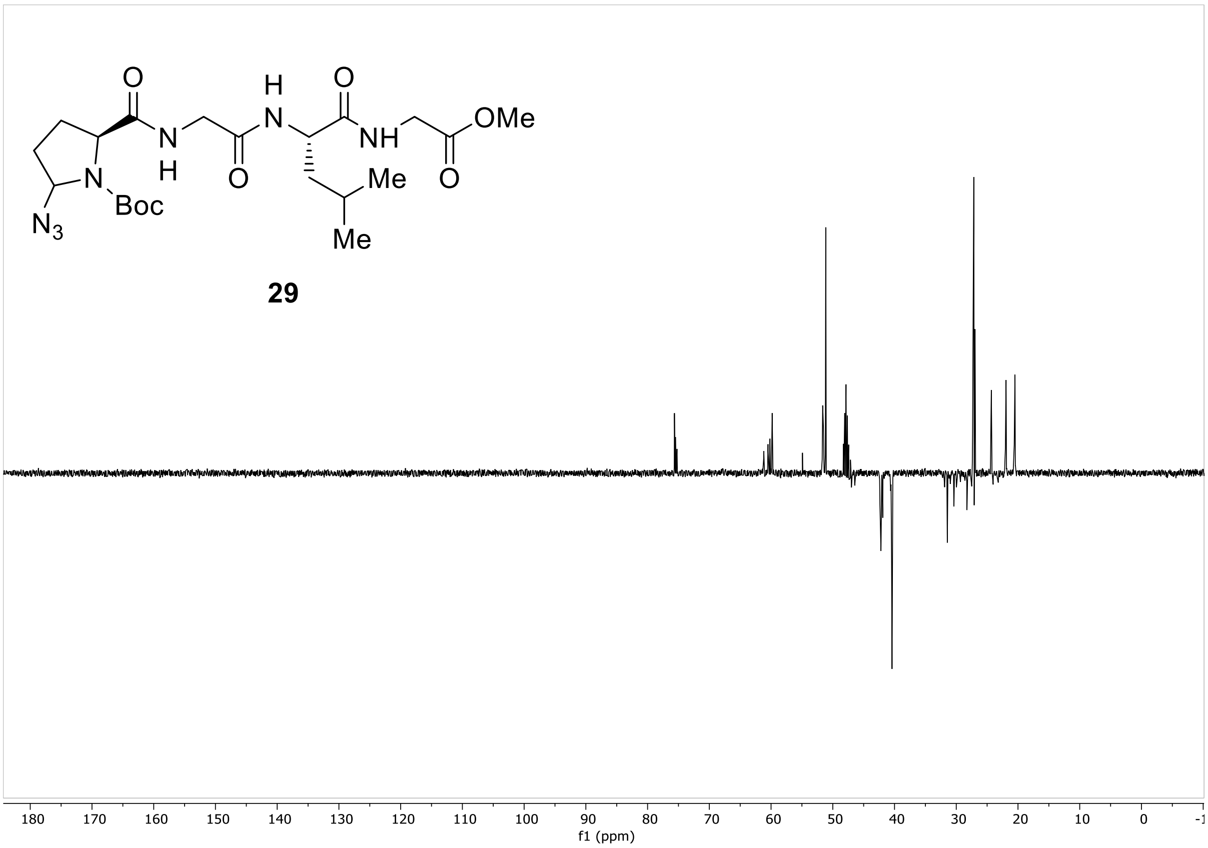

^{1}H NMR (400 MHz, MeOD-d_4, 278.2 K, mixture of two rotamers) of compound **34a**

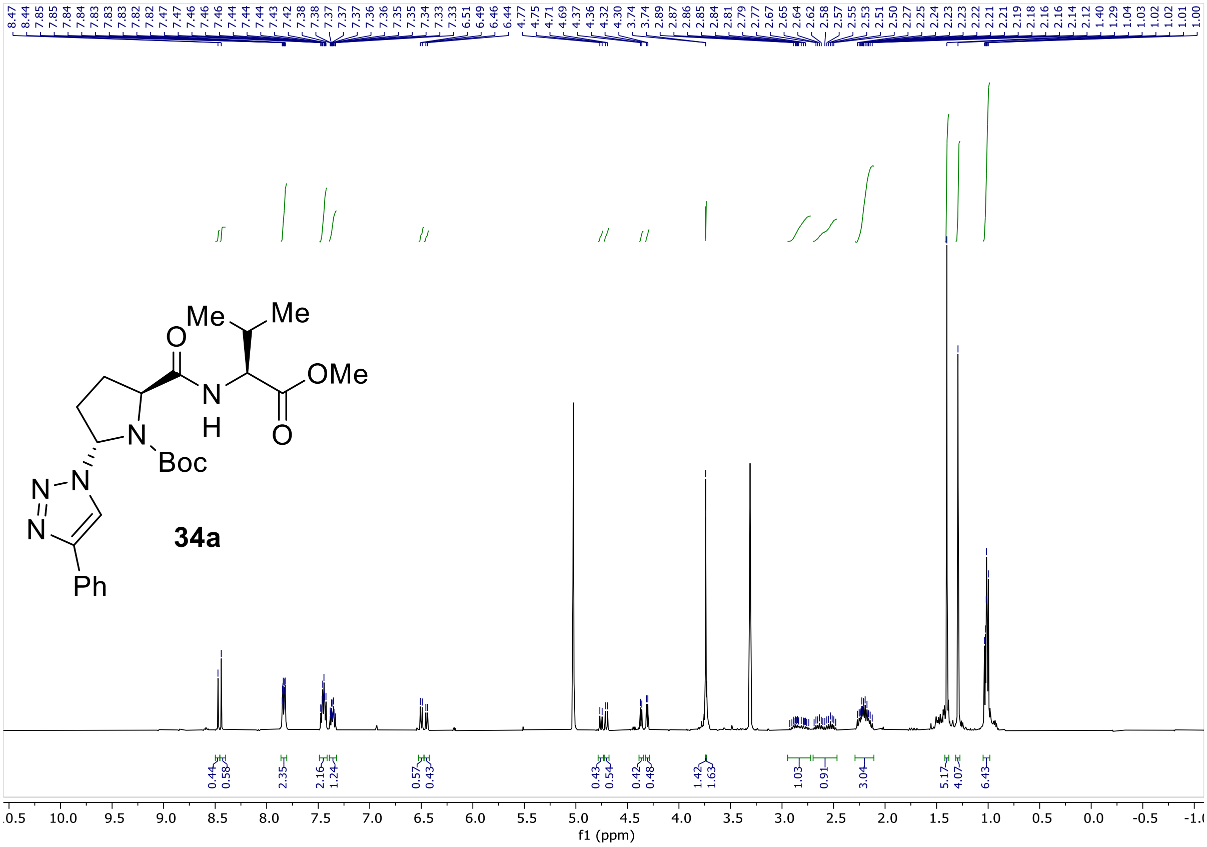

^{13}C NMR (101 MHz, MeOD-d_4, 278.2 K, mixture of two rotamers) of compound **34a**

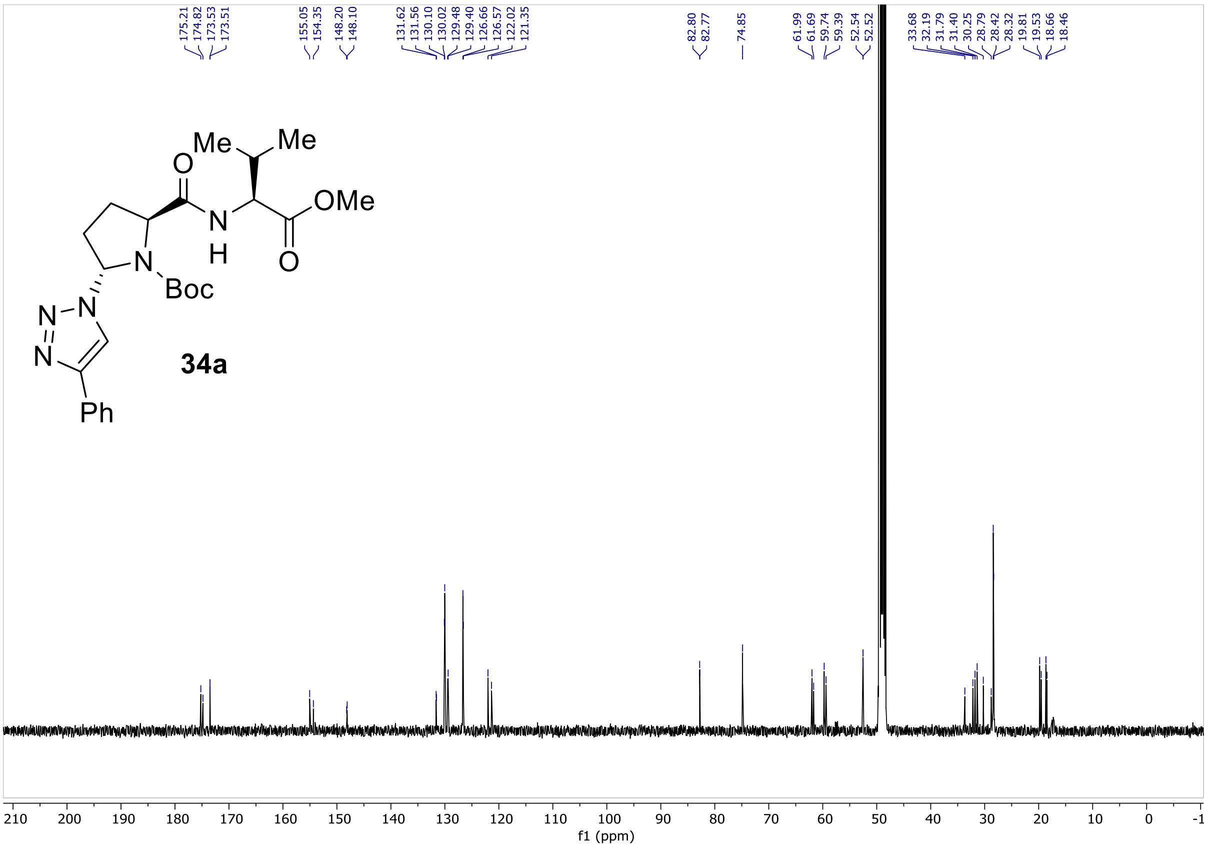

DEPT (101 MHz, MeOD-*d*$_4$, 278.2 K, mixture of two rotamers) of compound **34a**

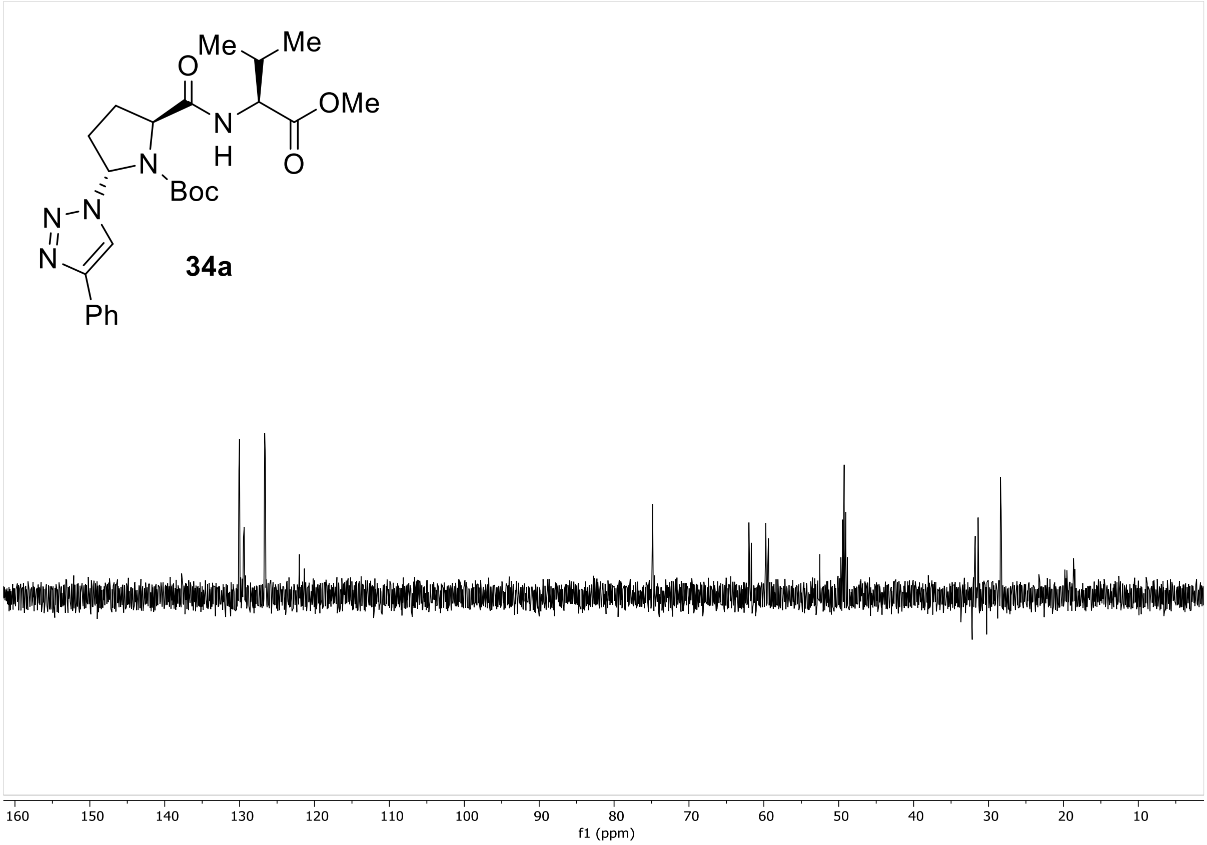

^{1}H NMR (400 MHz, MeOD-*d*$_4$, 298 K, mixture of two rotamers) of compound **34b**

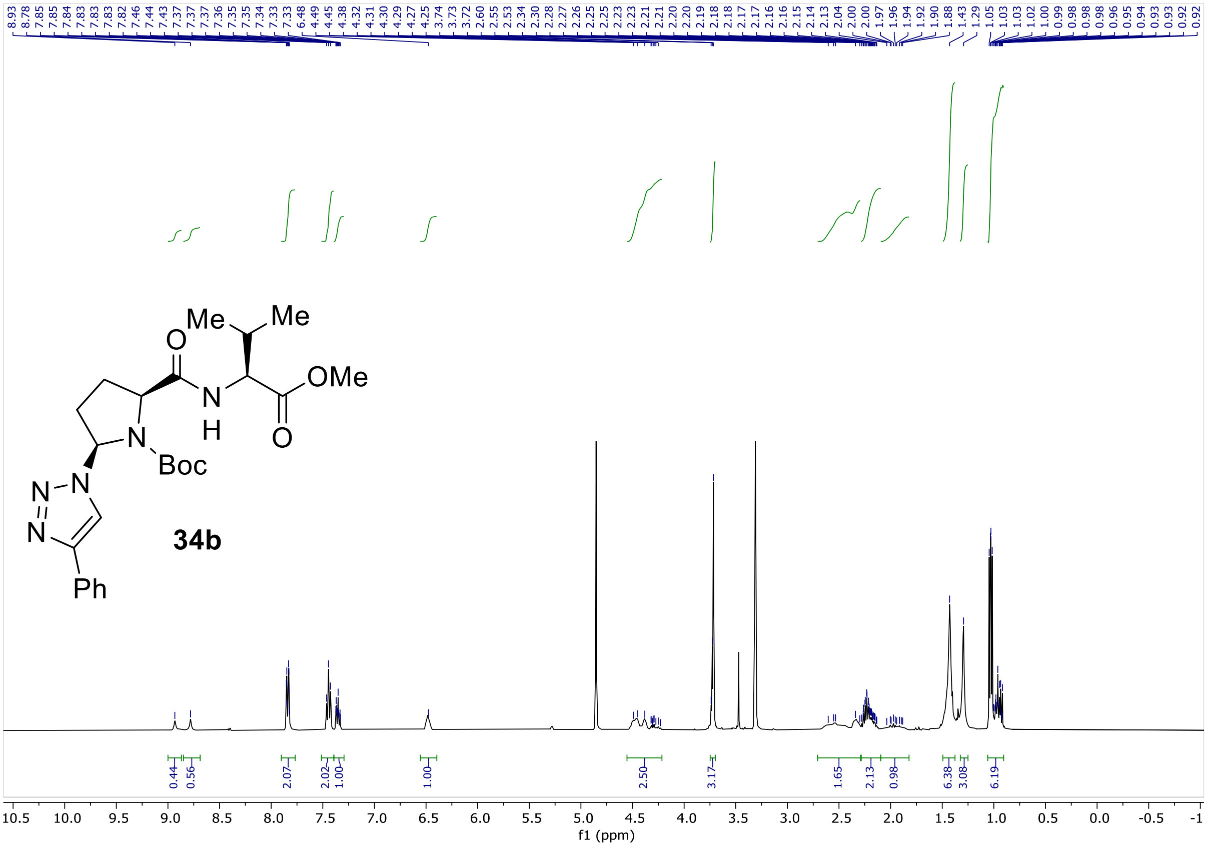

^{13}C NMR (101 MHz, MeOD-*d*$_4$, 278.2 K, mixture of two rotamers) of compound **34b**

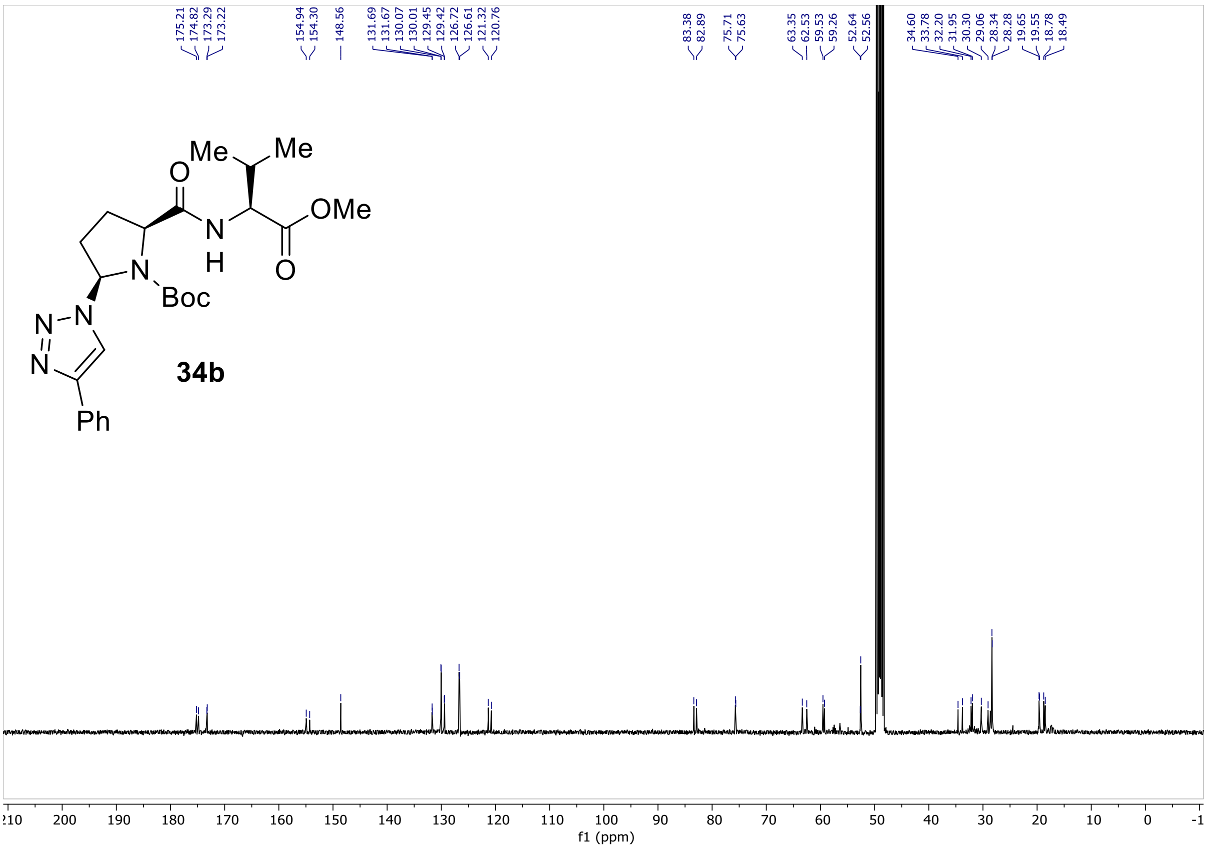

HSQC (MeOD-d_4, 278.2 K, mixture of two rotamers) of compound **34b**

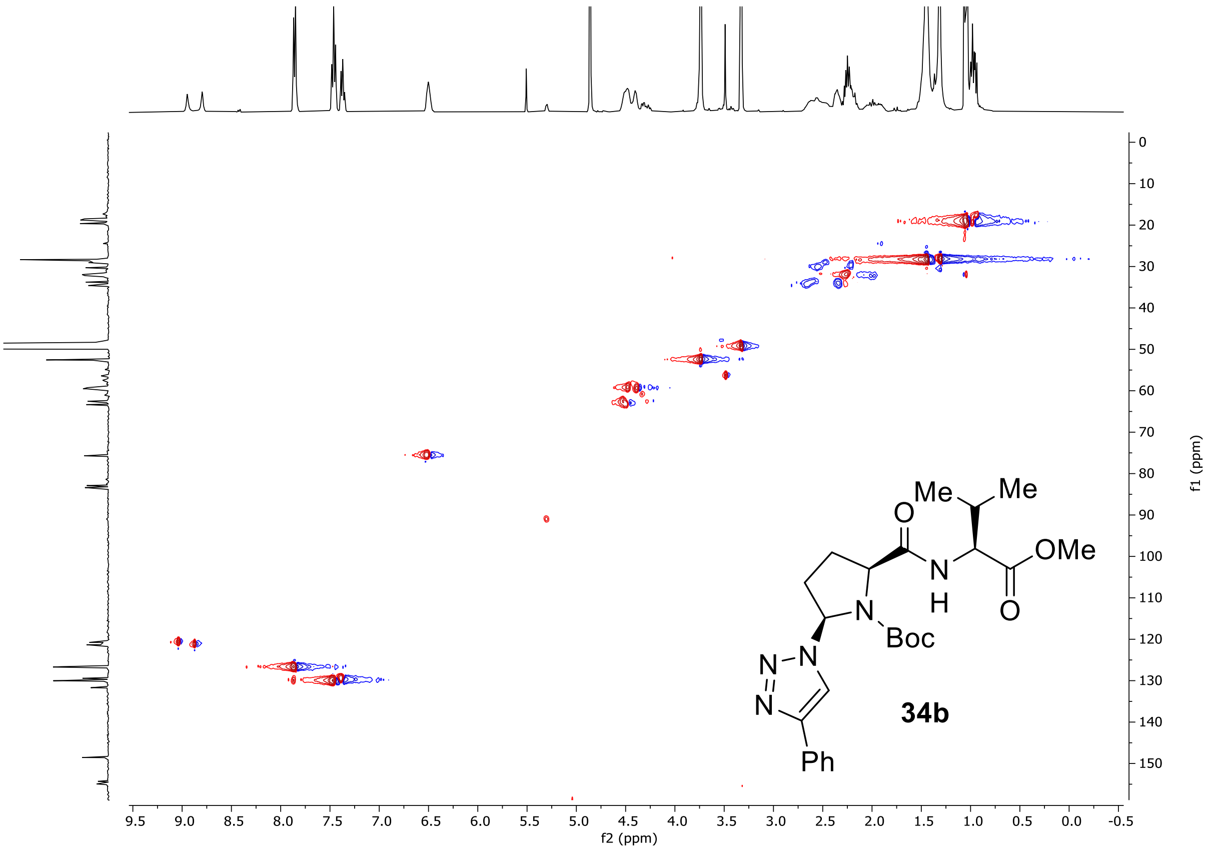

^{1}H NMR (400 MHz, MeOD-*d₄*, 298 K, mixture of two diastereoisomers) of compound **35**

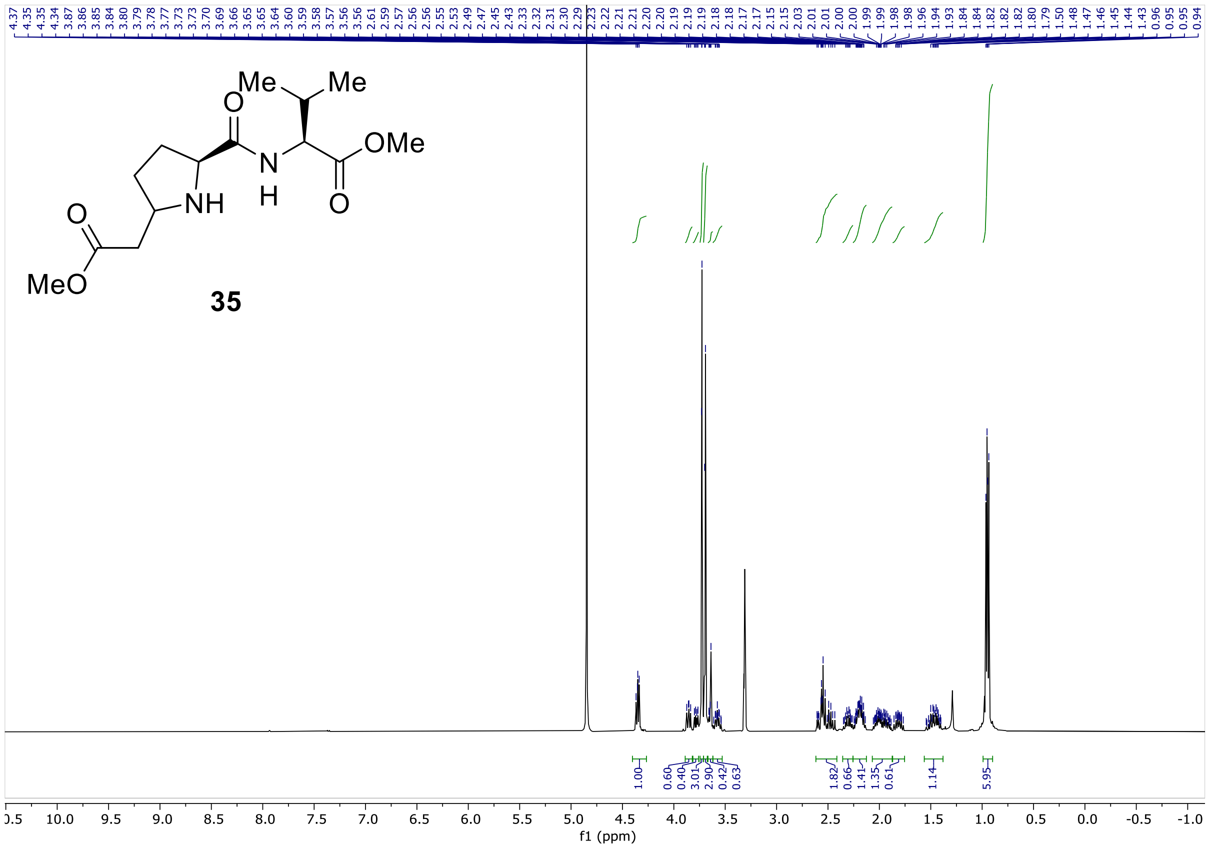

^{13}C NMR (101 MHz, MeOD-*d₄*, 298 K, mixture of two diastereoisomers) of compound **35**

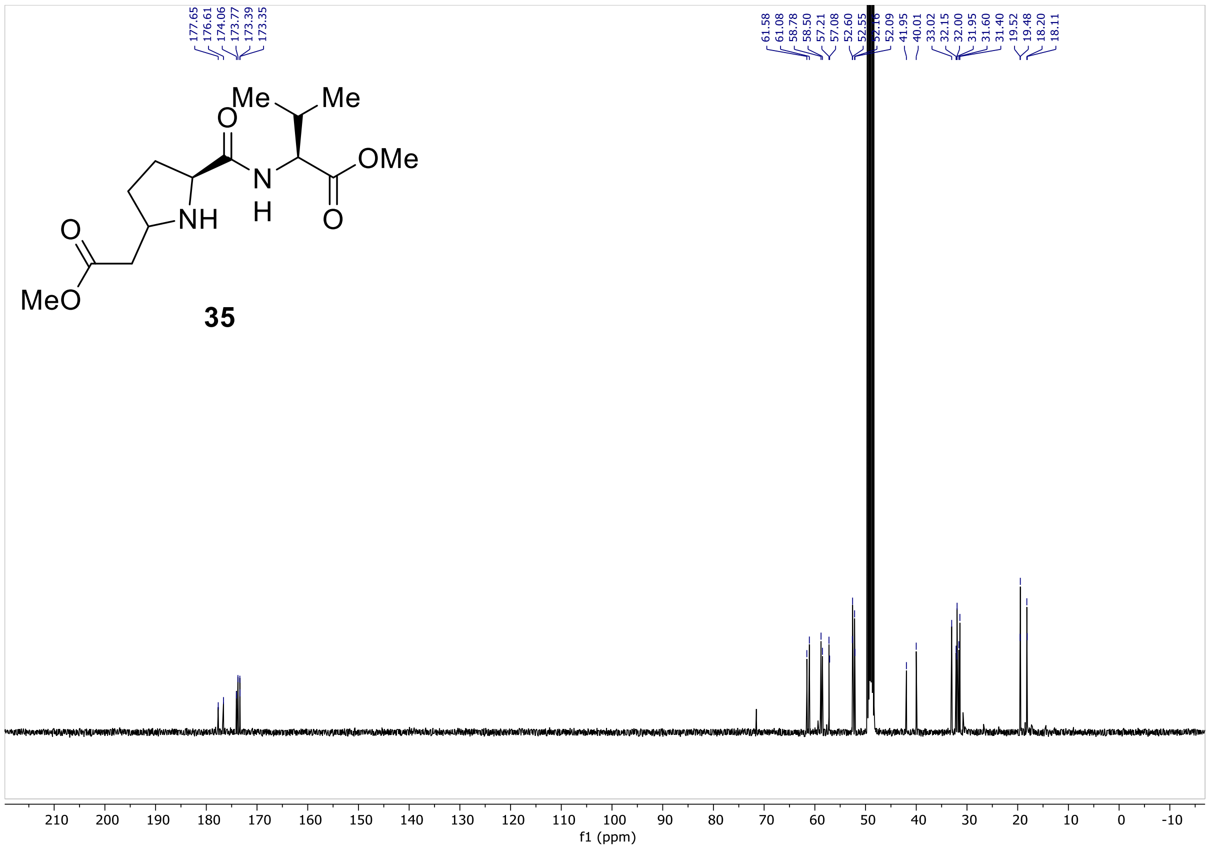

DEPT-135 (101 MHz, MeOD-d_4, 298 K, mixture of two diastereoisomers) of compound **35**

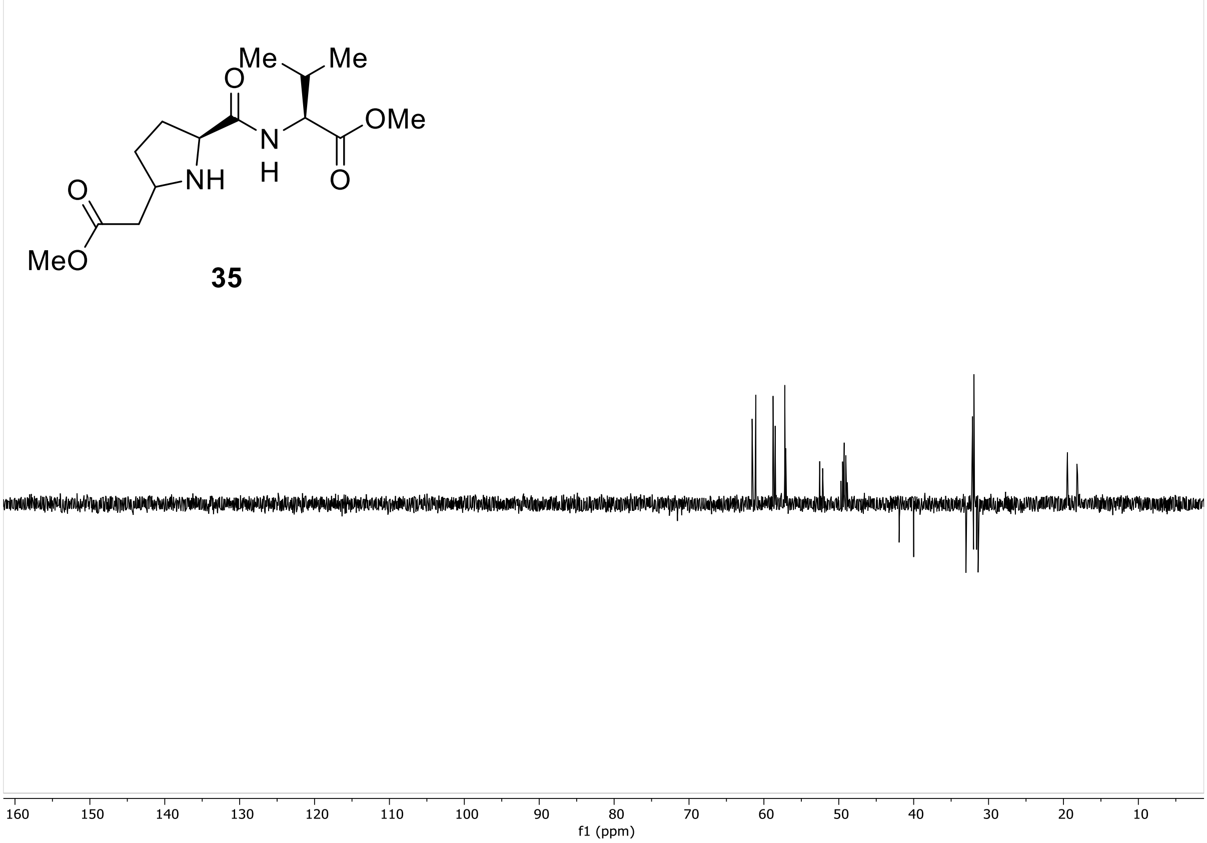

^{1}H NMR (400 MHz, MeOD-*d*$_4$, 298 K, mixture of two diastereoisomers) of **36**

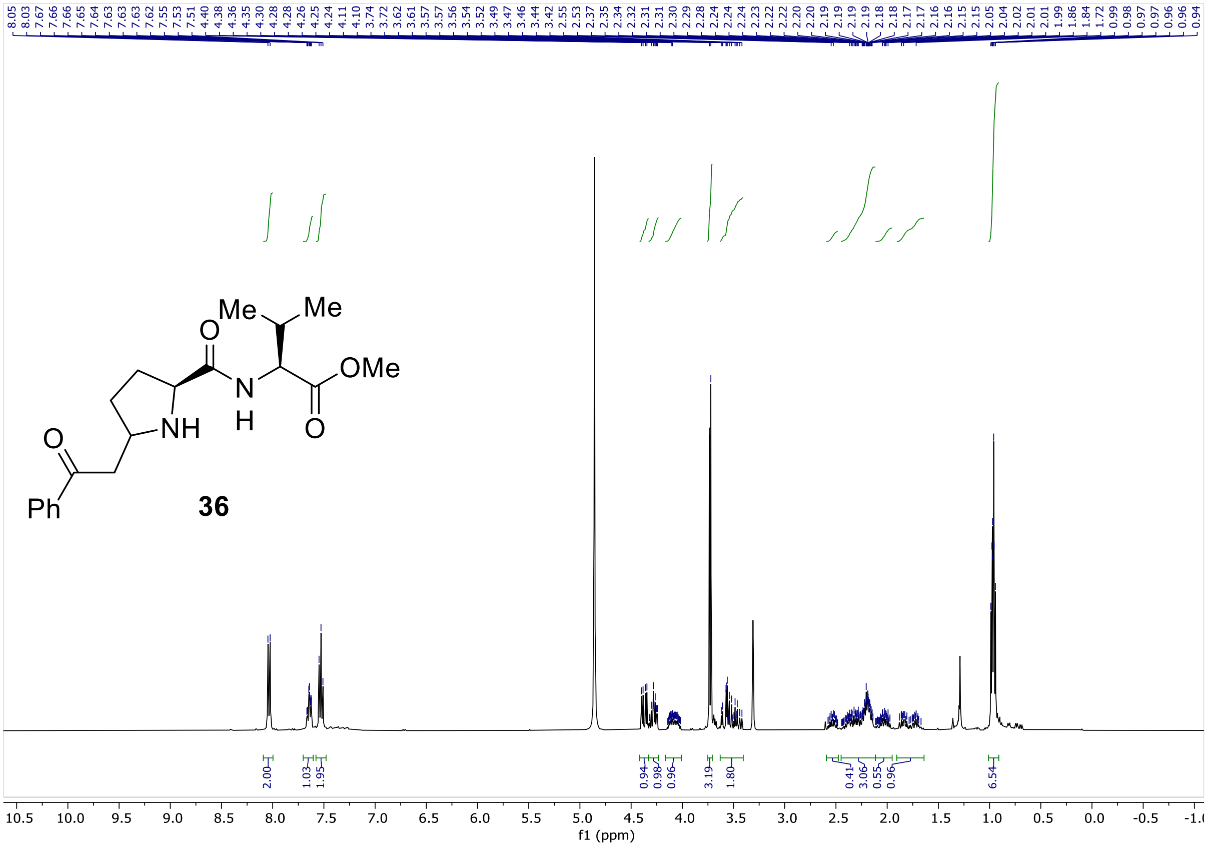

^{13}C NMR (101 MHz, MeOD-*d*$_4$, 298 K, mixture of two diastereoisomers) of **36**

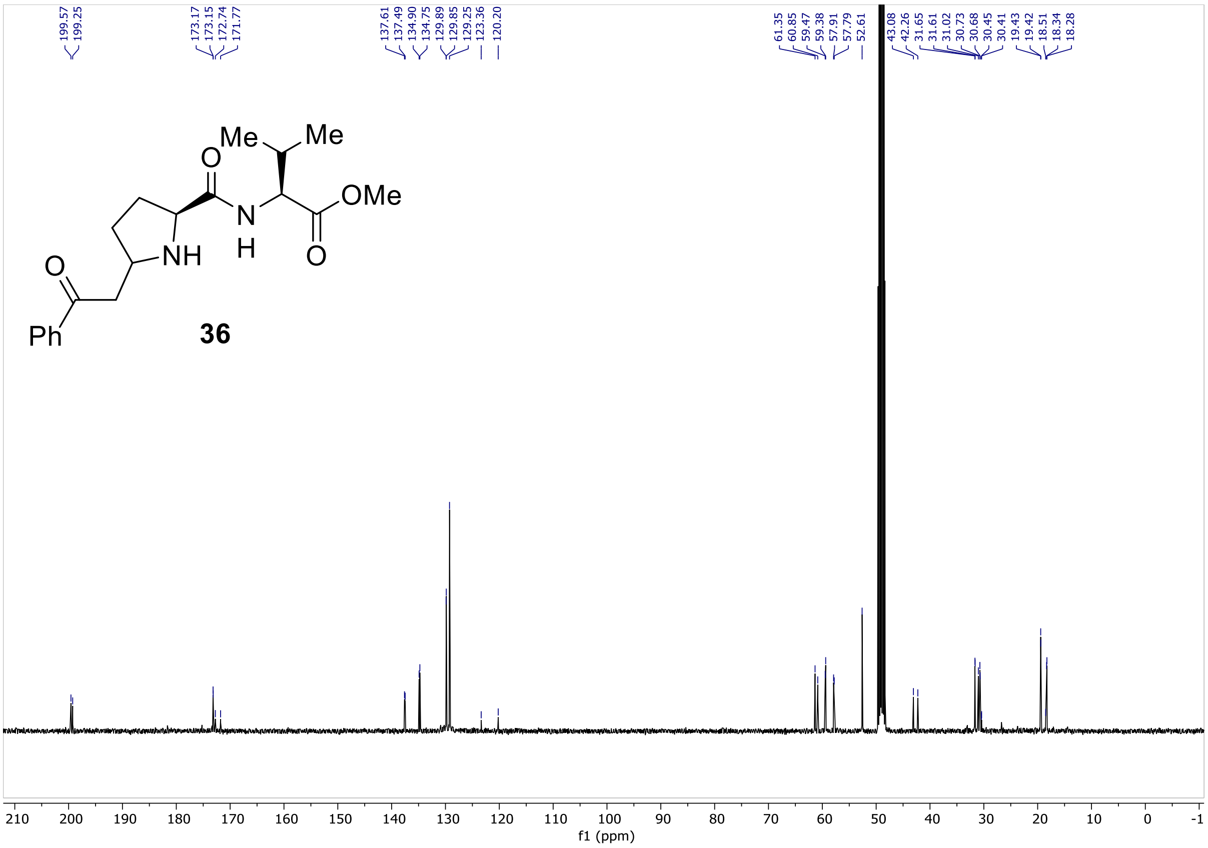

DEPT-135 (101 MHz, MeOD-d_4, 298 K, mixture of two diastereoisomers) of **36**

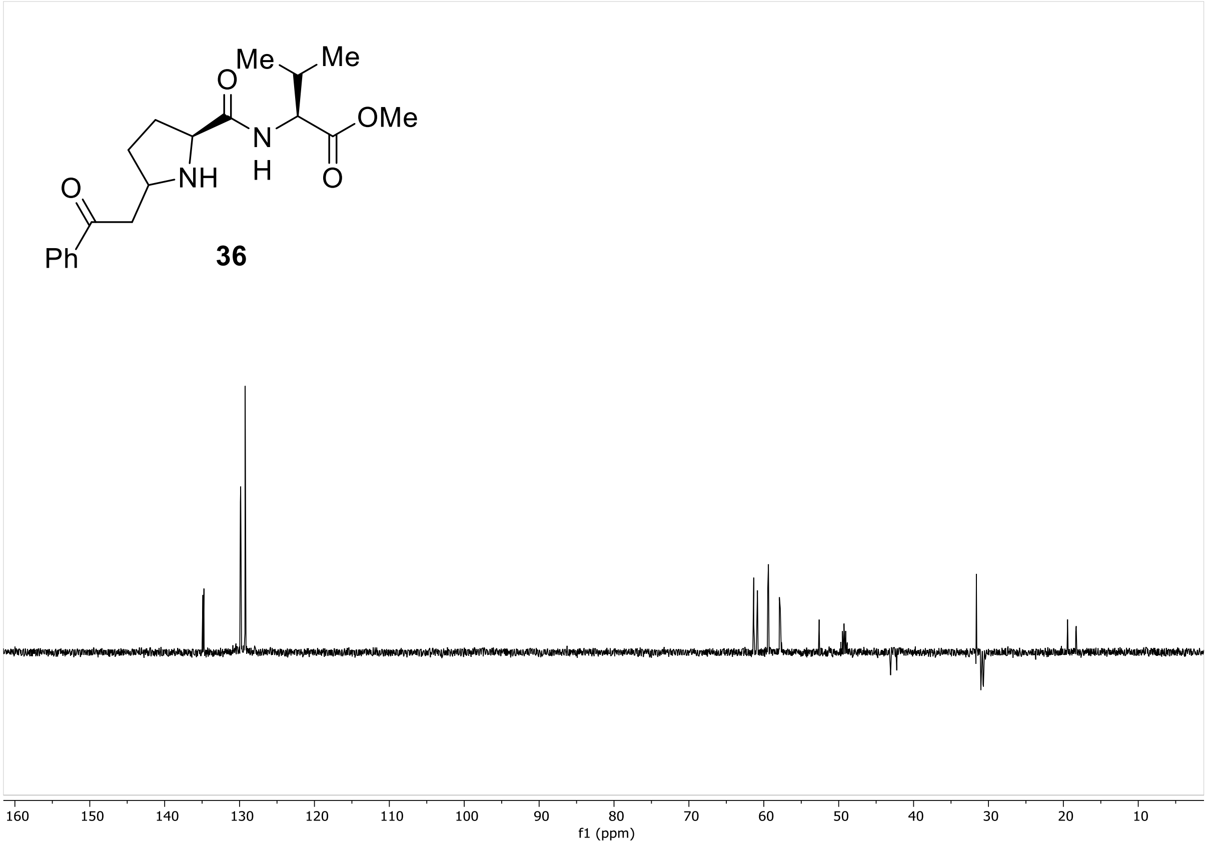

^{1}H NMR (400 MHz, MeOD-d_4, 298 K, mixture of two diastereoisomers) of compound **37**

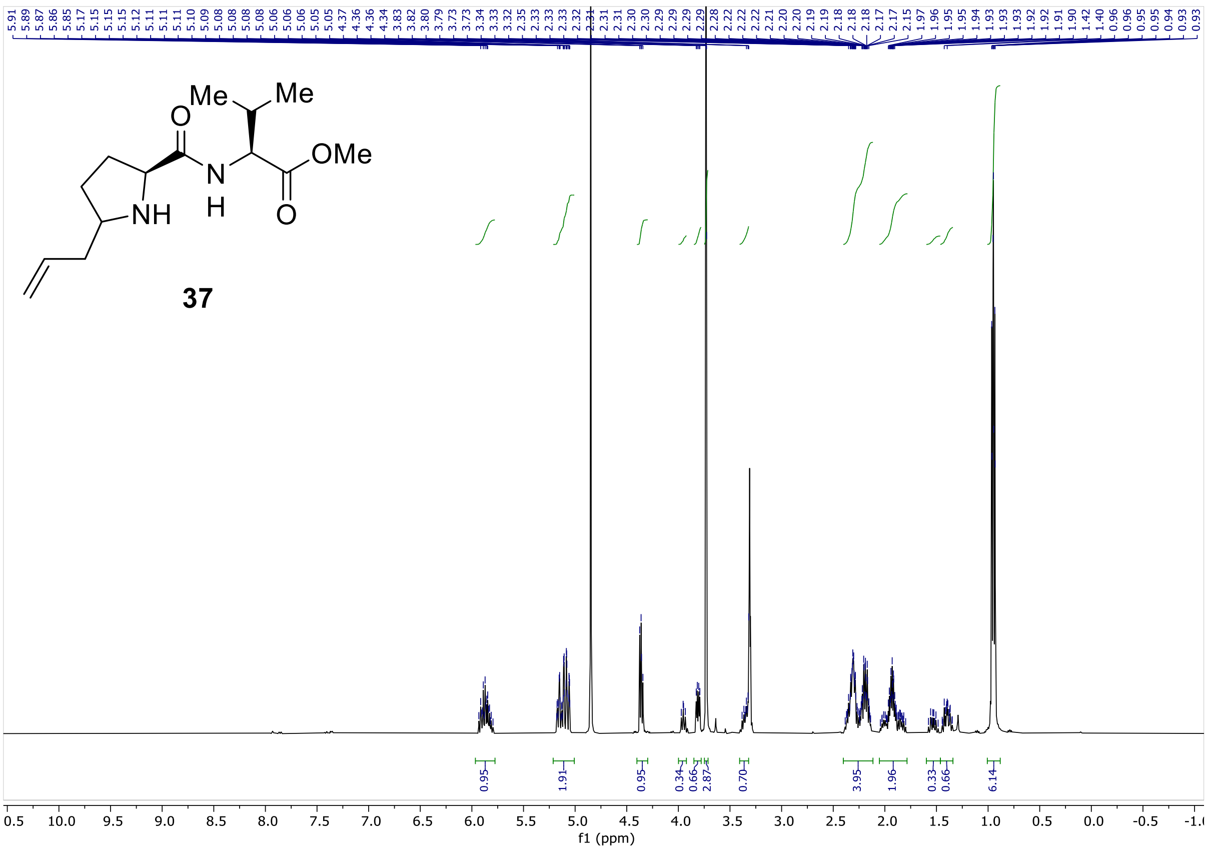

^{13}C NMR (101 MHz, MeOD-d_4, 298 K, mixture of two diastereoisomers) of compound **37**

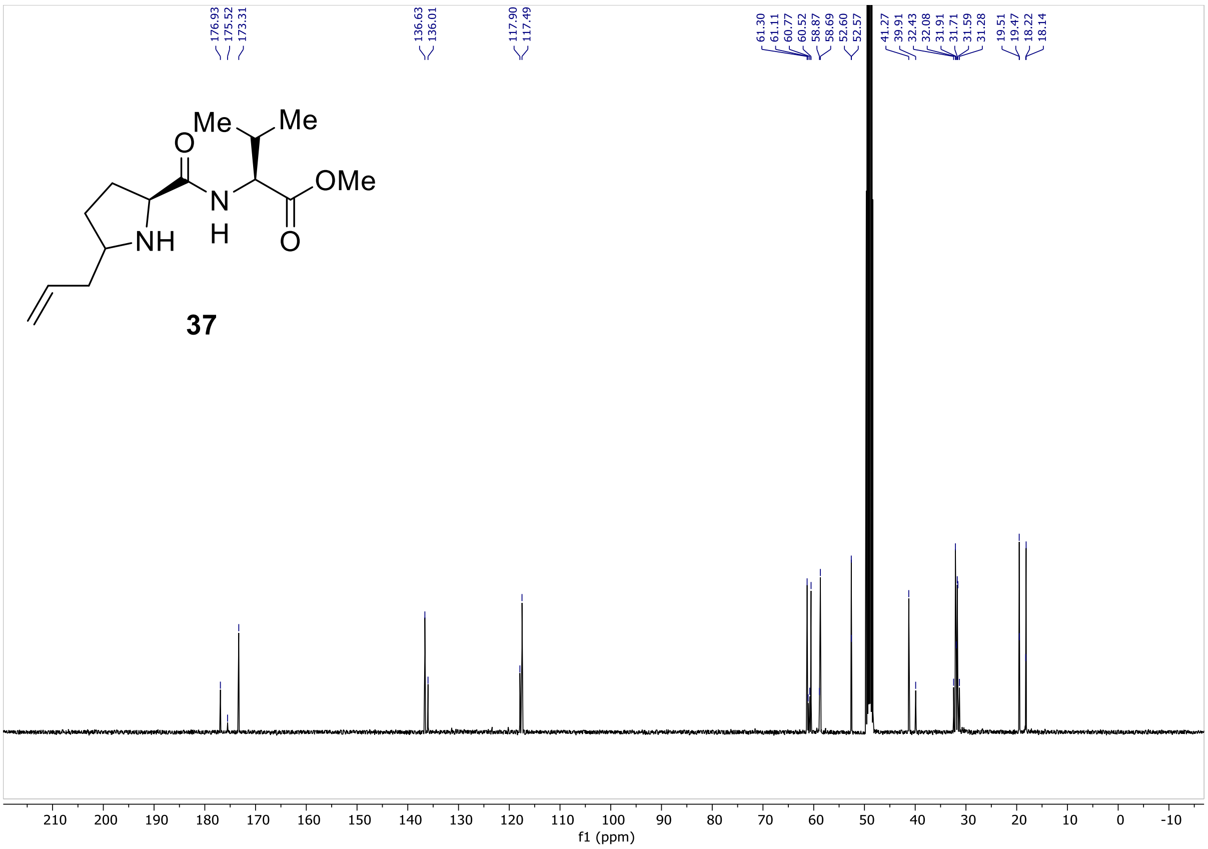

DEPT-135 (101 MHz, MeOD-d_4, 298 K, mixture of two diastereoisomers) of compound **37**

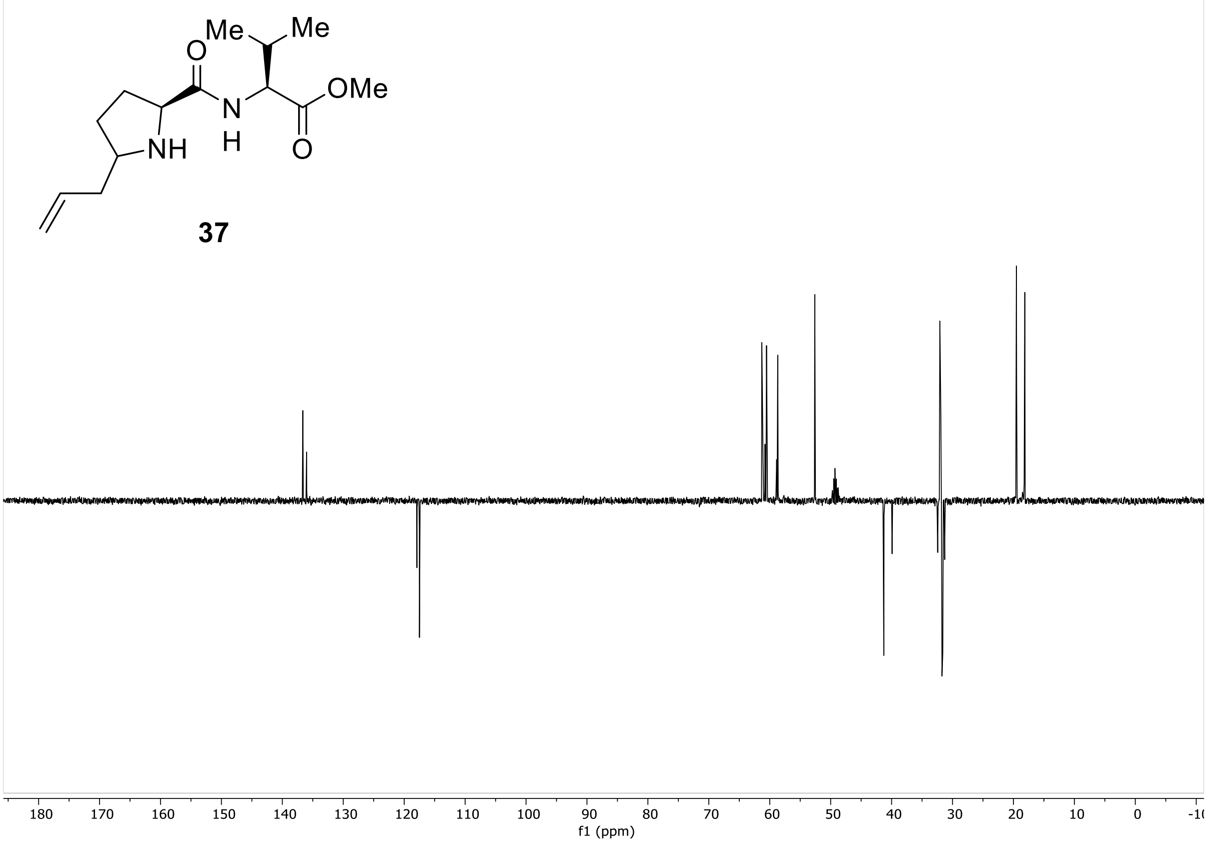

^{1}H NMR (400 MHz, MeOD-d_4, 298 K, mixture of two diastereoisomers) of **38**

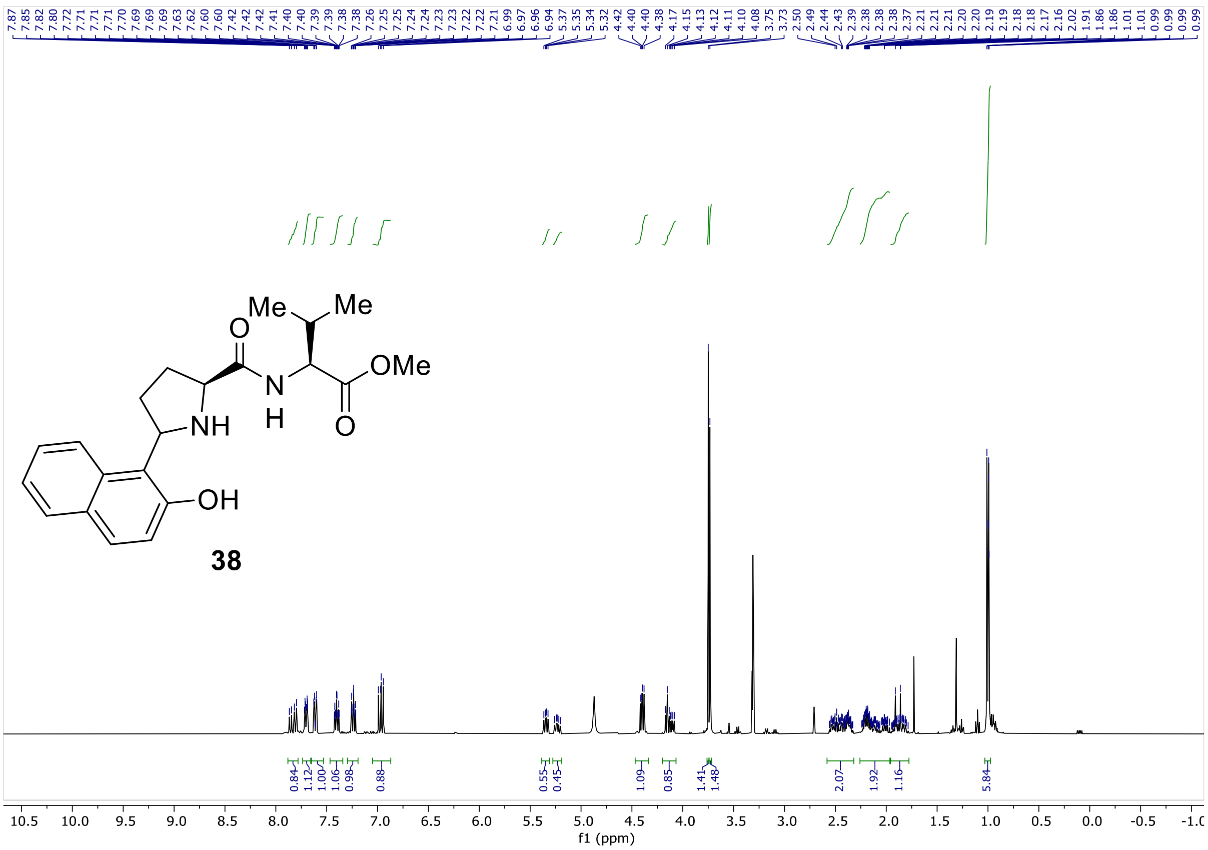

^{13}C NMR (101 MHz, MeOD-d_4, 298 K, mixture of two diastereoisomers) of **38**

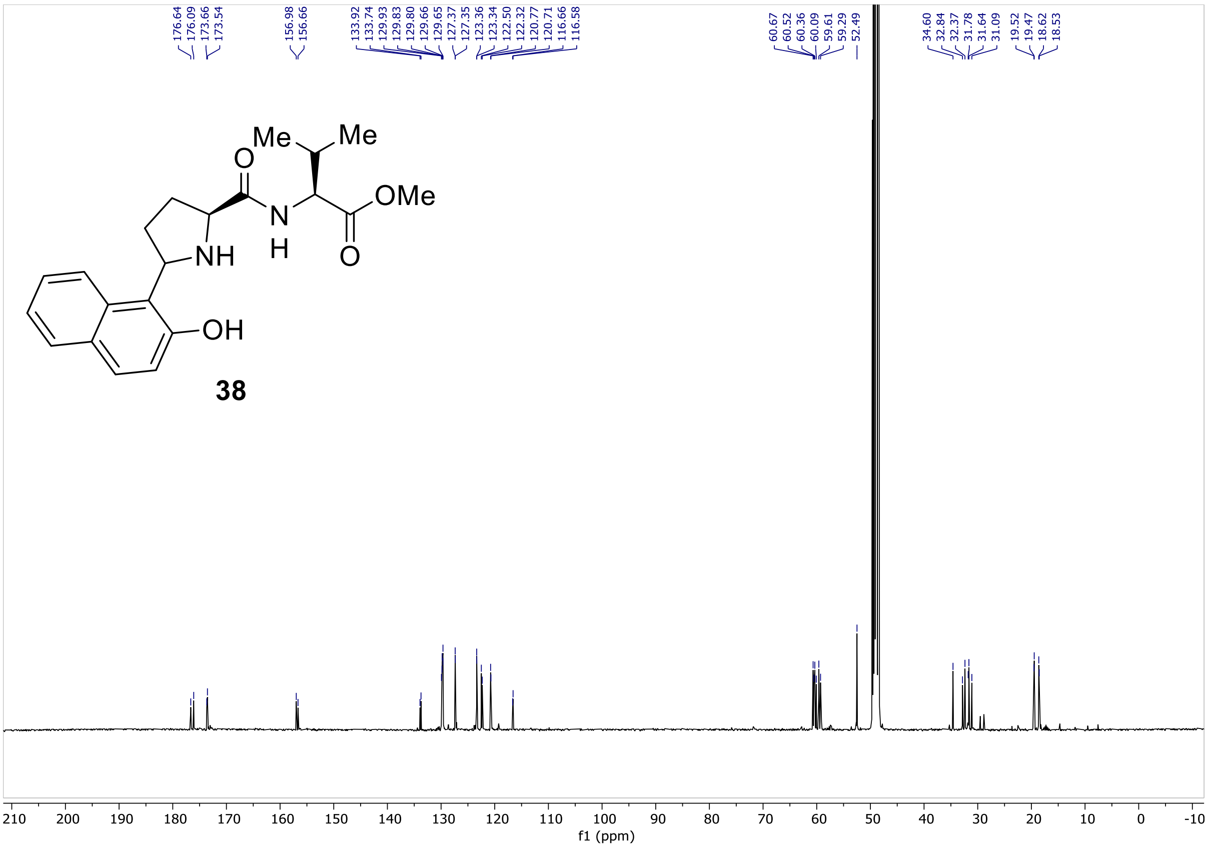

HSQC (MeOD-d_4, 298 K, mixture of two diastereoisomers) of **38**

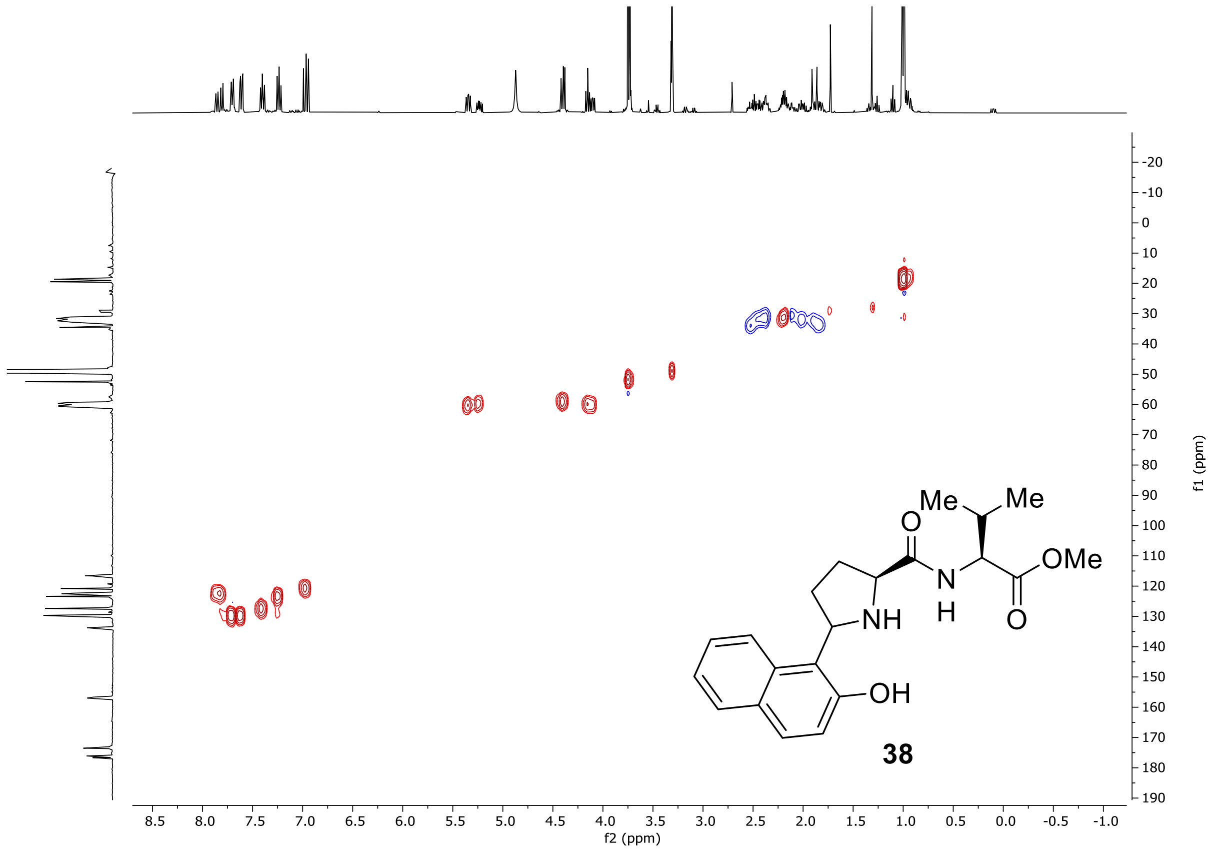

^{1}H NMR (400 MHz, MeOD-d_4, 298 K, mixture of two diastereoisomers) of **39**

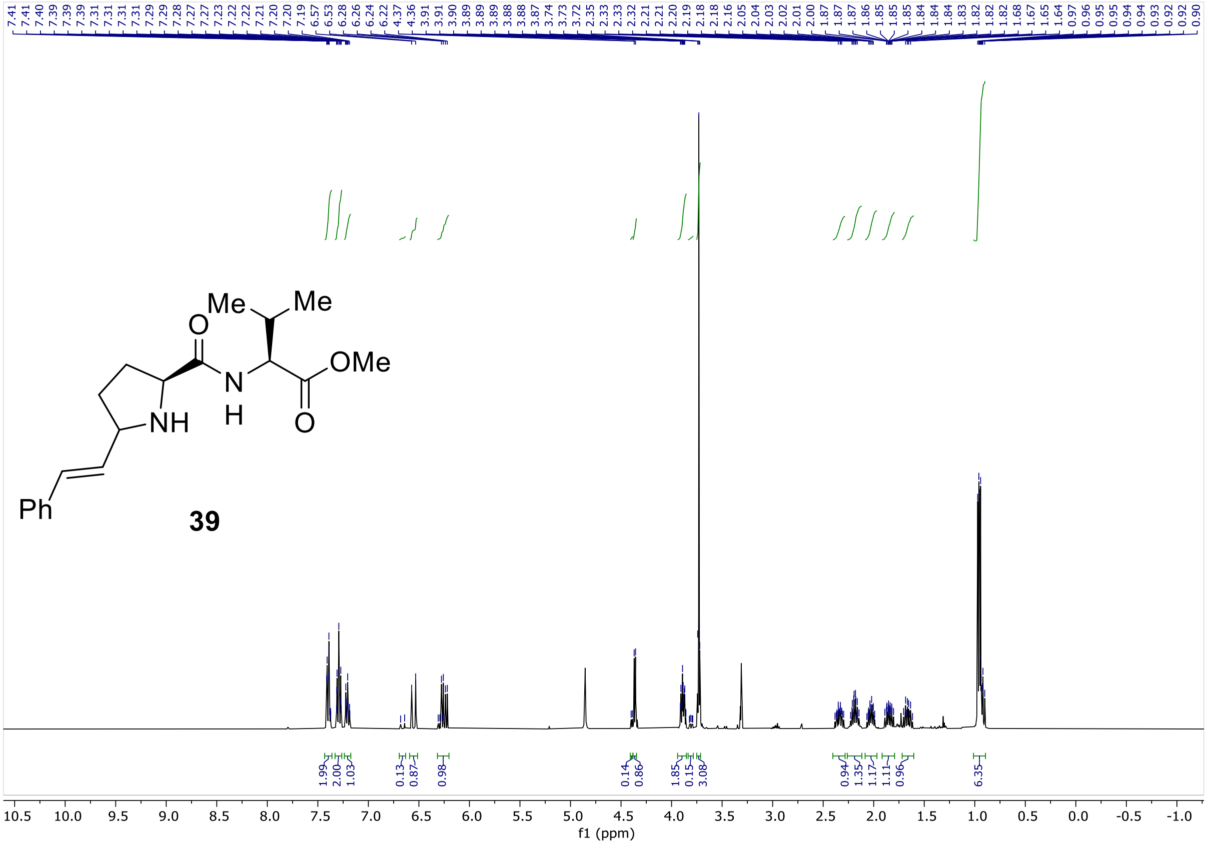

^{13}C NMR (101 MHz, MeOD-d_4, 298 K, mixture of two diastereoisomers) of **39**

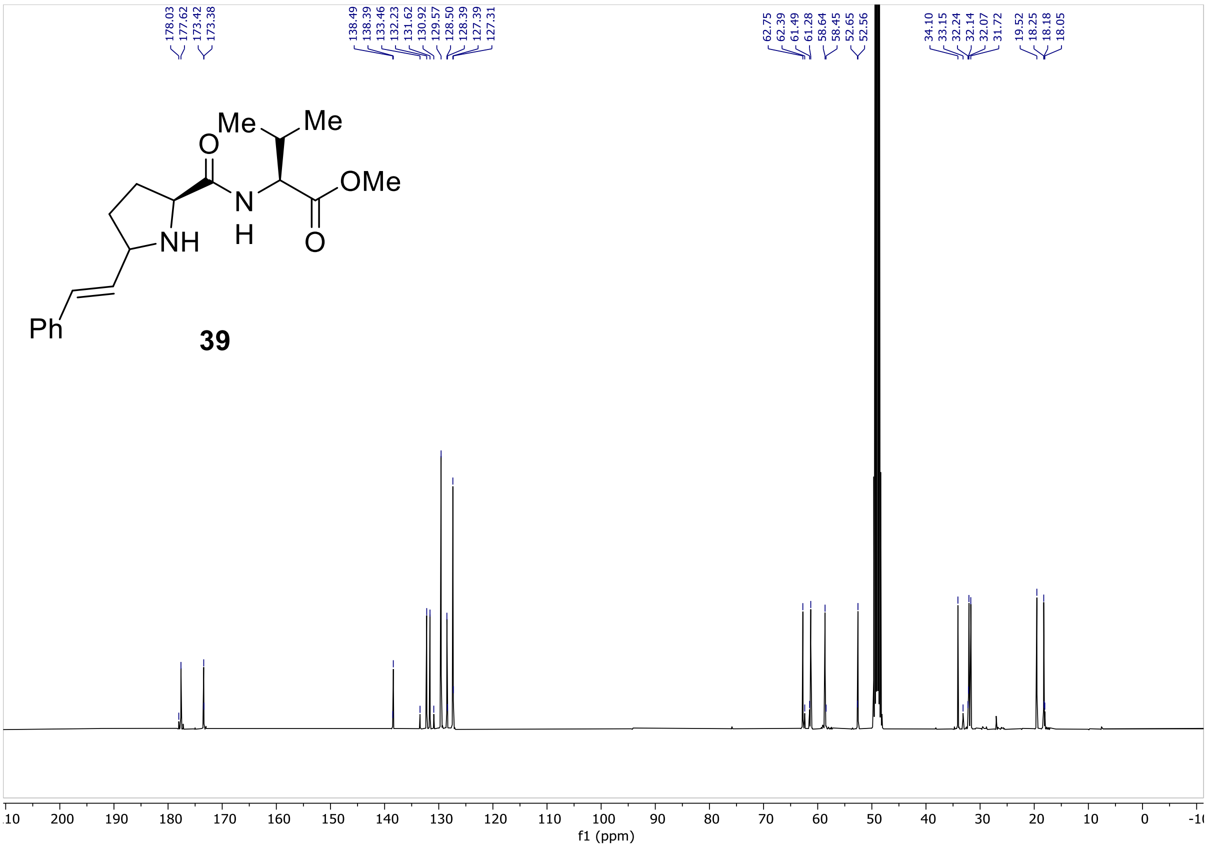

DEPT-135 (101 MHz, MeOD-d_4, 298 K, mixture of two diastereoisomers) of **39**

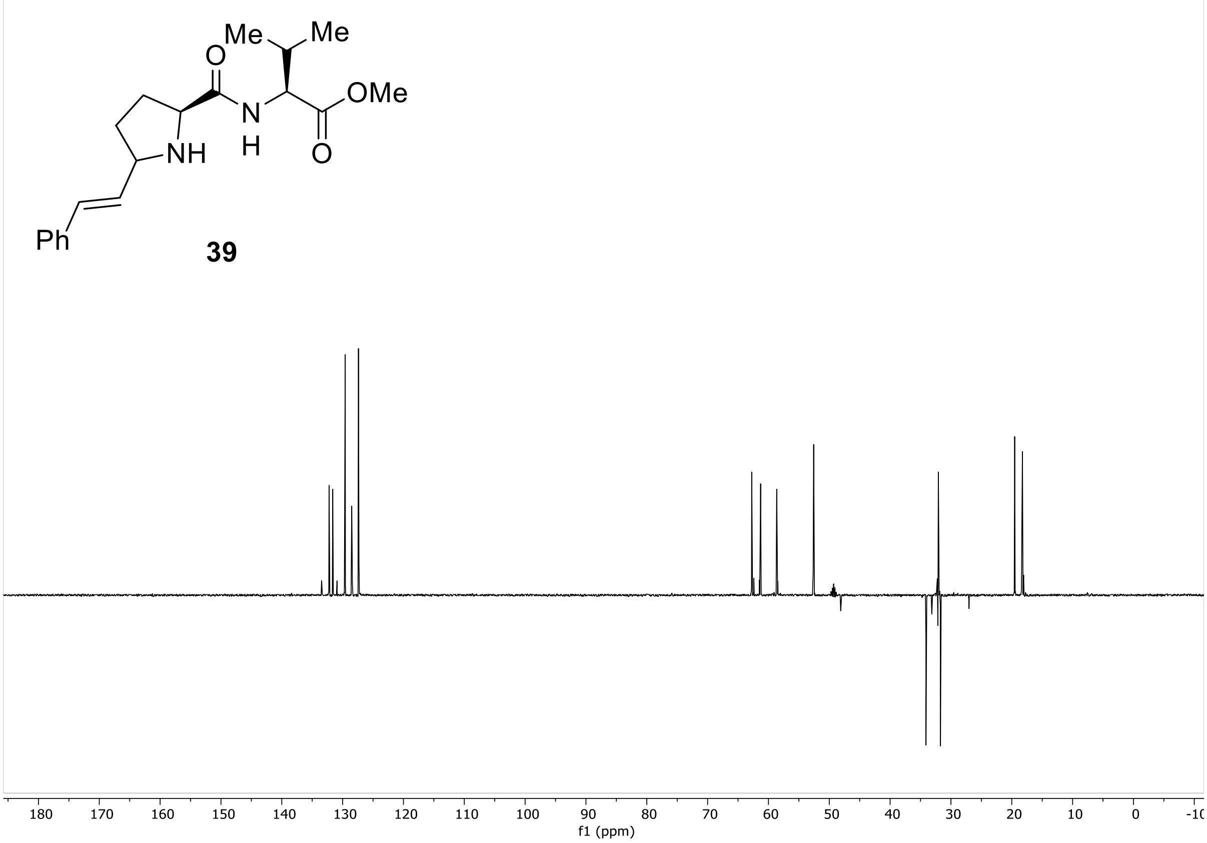

Supplement: Supplementary file 1 — Supporting Information [file CHEM-28-0-s001.pdf]
